# A *TNIP1*-driven systemic autoimmune disorder with elevated IgG4

**DOI:** 10.1038/s41590-024-01902-0

**Published:** 2024-07-26

**Authors:** Arti Medhavy, Vicki Athanasopoulos, Katharine Bassett, Yuke He, Maurice Stanley, Daniel Enosi Tuipulotu, Jean Cappello, Grant J. Brown, Paula Gonzalez-Figueroa, Cynthia Turnbull, Somasundhari Shanmuganandam, Padmaja Tummala, Gemma Hart, Tom Lea-Henry, Hao Wang, Sonia Nambadan, Qian Shen, Jonathan A. Roco, Gaetan Burgio, Phil Wu, Eun Cho, T. Daniel Andrews, Matt A. Field, Xiaoqian Wu, Huihua Ding, Qiang Guo, Nan Shen, Si Ming Man, Simon H. Jiang, Matthew C. Cook, Carola G. Vinuesa

**Affiliations:** 1 Division of Immunology and Infectious Disease, John Curtin School of Medical Research, Australian National University, Canberra, Australian Capital Territory Australia; 2 China Australia Center for Personalized Immunology, Shanghai Renji Hospital, Shanghai Jiaotong University, Shanghai, China; 3 Francis Crick Institute, London, UK; 4 Center for Tropical Bioinformatics and Molecular Biology, Australian Institute of Tropical Health and Medicine, James Cook University, Cairns, Queensland Australia; 5 Department of Rheumatology, Renji Hospital, Shanghai Jiao Tong University, School of Medicine, Shanghai, China; 6 Department of Medicine, University of Cambridge, Cambridge, UK

**Keywords:** Autoimmunity, Autoimmune diseases

## Abstract

Whole-exome sequencing of two unrelated kindreds with systemic autoimmune disease featuring antinuclear antibodies with IgG4 elevation uncovered an identical ultrarare heterozygous *TNIP1*^*Q333P*^ variant segregating with disease. Mice with the orthologous Q346P variant developed antinuclear autoantibodies, salivary gland inflammation, elevated IgG2c, spontaneous germinal centers and expansion of age-associated B cells, plasma cells and follicular and extrafollicular helper T cells. B cell phenotypes were cell-autonomous and rescued by ablation of Toll-like receptor 7 (TLR7) or MyD88. The variant increased interferon-β without altering nuclear factor kappa-light-chain-enhancer of activated B cells signaling, and impaired MyD88 and IRAK1 recruitment to autophagosomes. Additionally, the Q333P variant impaired TNIP1 localization to damaged mitochondria and mitophagosome formation. Damaged mitochondria were abundant in the salivary epithelial cells of *Tnip1*^*Q346P*^ mice. These findings suggest that TNIP1-mediated autoimmunity may be a consequence of increased TLR7 signaling due to impaired recruitment of downstream signaling molecules and damaged mitochondria to autophagosomes and may thus respond to TLR7-targeted therapeutics.

## Abstract (web summary)

Vinuesa et al. identify patients with systemic autoimmunity and a *TNIP1* variant that result in dysregulated B cell function. Mice with the orthologous *Tnip1* mutation develop spontaneous autoimmunity associated with impaired mitophagy and autophagic silencing of proteins downstream of Toll-like receptor 7 signaling.

## Main

The validation of rare and new pathogenic variants identified through next-generation sequencing has provided critical insights into the mechanisms driving human systemic autoimmunity^1–3^. While common variants in TNFAIP3 interacting protein 1 (*TNIP1*) have been associated with human autoimmunity through genome-wide association studies (GWAS)^4–6^, to date no cases have been attributed to *TNIP1* variants.

TNIP1, or A20-binding inhibitor of NF-kappa-B activation 1 (ABIN-1), suppresses nuclear factor kappa-light-chain-enhancer of activated B cells (NF-κB) activity in response to Toll-like receptor (TLR), tumor necrosis factor (TNF), interleukin-1 (IL-1) and CD40 activation. The inhibitory activity of TNIP1 on NF-κB signaling depends on its capacity to bind polyubiquitin moieties on signal transducers. Mice harboring a loss-of-function (D485N) mutation within the protein’s ubiquitin-binding domain develop lupus-like disease comparable to phenotypes observed in *Tnip1* knockout mice^7,8^. *Tnip1*^*D485N*^ mice exhibit dysregulated NF-κB, c-Jun N-terminal kinase and p38α mitogen-activated protein kinase proinflammatory signaling^7^ with autoimmunity ameliorated by Toll-like receptor 7 (TLR7) and MyD88 deficiency or interleukin-1 receptor-associated kinase 1 (IRAK1) and interleukin-1 receptor-associated kinase 4 (IRAK4)-inactivating mutations^7,9,10^. By contrast, pathogenesis in *Tnip1* knockout mice was attributed to CCAAT/enhancer-binding protein beta (CEBPβ) target gene upregulation^8^, suggesting that domain-specific *Tnip1* mutations may impact distinct immunological networks.

Recent studies identified TNIP1 as a selective autophagy receptor^11^ and regulator of mitophagy^12^. A link between TNIP1’s role in autophagosomes and mitophagosomes, and disease, has yet to be described. We report the first human pathogenic variant in *TNIP1* in two unrelated kindreds. We show that the *TNIP1* variant increases type I interferon (IFN1), prevents silencing of MyD88 and IRAK1 signaling in autophagosomes and impairs mitophagy, with all cellular phenotypes being TLR7-dependent.

## Results

### *TNIP1*^*Q333P*^ in autoimmune patients with increased IgG4

**Fig. 1:**
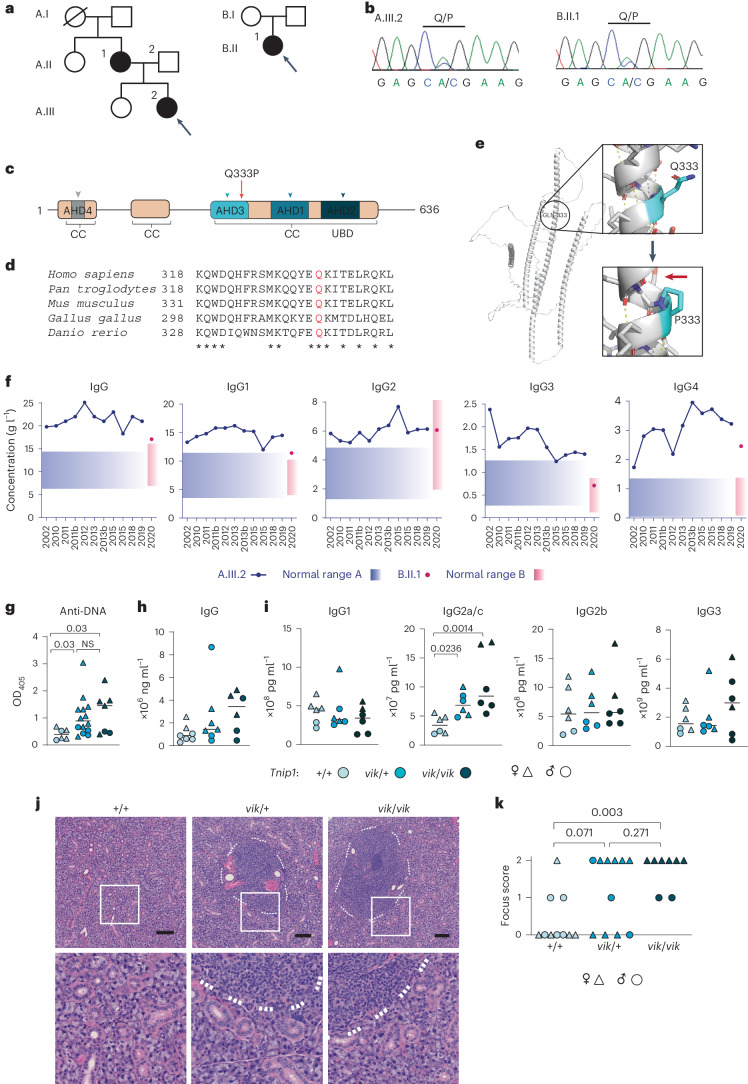
Orthologous *TNIP1* missense variant in patients and mice with systemic autoimmunity. **a**,**b**, *TNIP1*^*Q333P*^ variant in families (**a**) and results from Sanger sequencing (**b**). **c**, Position of the Q333P variant within the TNIP1 protein domains. **d**, Conservation across species. **e**, AlphaFold2-generated structure of canonical TNIP1 (AF-Q15025-F1) as modeled in PyMOL. Residue 333 is indicated in blue. The red arrow indicates the P333-disrupted hydrogen bonding in the alpha-helix 4 backbone **f**, Patient immunoglobulin IgG concentrations compared to the ‘normal’ population ranges (shaded boxes) as defined by the Canberra Hospital (Australia) and Renji Hospital (Shanghai, China) pathology laboratories, respectively. **g**, Serum antibodies to DNA from 20–28-week-old male (*n* = 8) and female (*n* = 19) *vikala* mice; *Tnip1*^+/+^ (*n* = 6), *Tnip1*^*vik*/+^ (*n* = 14) and *Tnip1*^*vik*/*vik*^ (*n* = 7) mice by enzyme-linked immunosorbent assay (ELISA). Optical density at 405 nm (OD_405_). **h**, Total serum IgG in 12-week-old male (*n* = 10) and female (*n* = 10) *vikala* mice by ELISA; *Tnip1*^+/+^ (*n* = 7), *Tnip1*^*vik*/+^ (*n* = 7) and *Tnip1*^*vik*/*vik*^ (*n* = 6). Each point represents a mouse (biological replicate). **i**, Meso Scale Discovery readout for IgG subclasses (pg ml^−1^) in 20–22-week-old male (*n* = 9) and female (*n* = 9) *vikala* mice; *Tnip1*^+/+^ (*n* = *6*), *Tnip1*^*vik*/+^ (*n* = 6) and *Tnip1*^*vik*/*vik*^ (*n* = 6); each point represents a mouse (biological replicate). **j**, H&E staining showing lymphocytic infiltrates in the submandibular salivary glands of 20–28-week-old *vikala* mice; *Tnip1*^+/+^ (*n* = 10), *Tnip1*^*vik*/+^ (*n* = 11) and *Tnip1*^vik/*vik*^ (*n* = 8). Scale bar, 100 μm. **k**, Focus score indicating the severity of lymphocytic infiltration per tissue section; each dot represents a mouse (biological replicate). Infiltrates are defined as foci consisting of 50 or more mononuclear cells. Scores: 0, no infiltrates; 1, one focal infiltrate; 2, multiple focal infiltrates. The medians are indicated by the black line, the mouse sex is indicated by the symbol type (**g**–**i**,**k**). In **g**,**h**,**j**, data are representative of *n* = 2 experiments. Data in **i** was from an experiment performed once; scoring in **k** was performed once. Statistical significance was calculated using a one-way analysis of variance (ANOVA) with multiple comparisons using a Tukey test after log-transformation of the data. Exact *P* values are shown. NS, not significant. Source data

**Extended Data Fig. 1:**
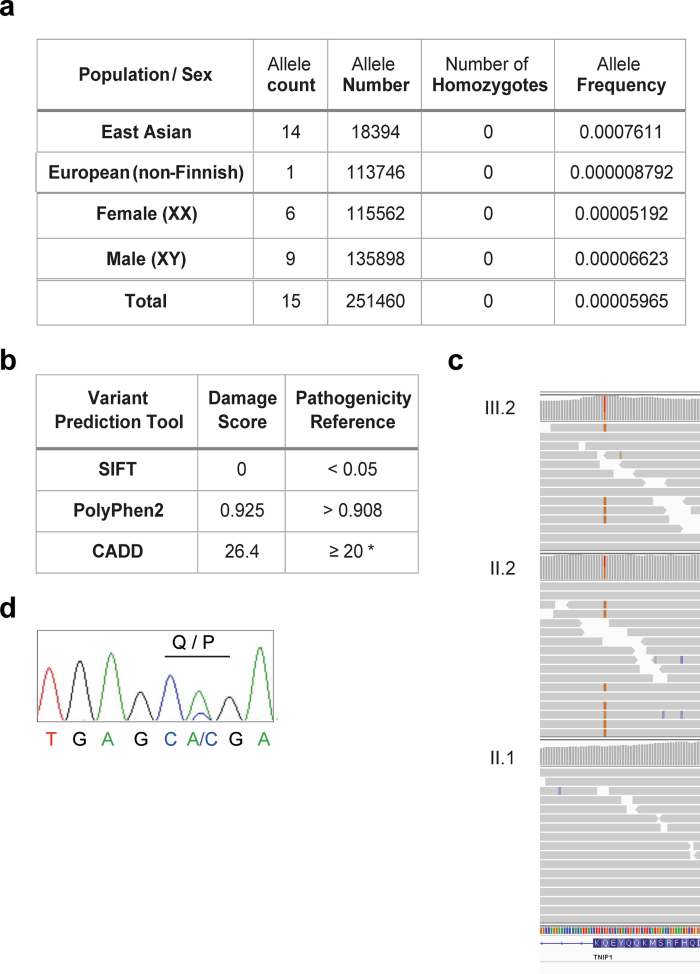
Ultrarare *TNIP1*^*Q333P*^ variant absent in unaffected family members and predicted to be damaging to TNIP1 function. **a**, GnomAD global frequencies of *TNIP1* variant in different populations and sexes. **b**, Variant pathogenicity prediction outputs for TNIP1^Q333P^. **c**, Integrative Genomics Viewer (IGV) image of the *TNIP1*^*Q333P*^ missense variant. **d**, Sequencing chromatogram validating *Tnip1*^*Q346P*^ mutation in the CRISPR/Cas9 generated *vikala* founder mouse.

We identified an ultrarare (Genome Aggregation Database minor allele frequency (MAF) = 0.00005965; Extended Data Fig. 1a) missense variant in *TNIP1* p.Gln333Pro (Q333P) present in two unrelated patients (Fig. 1a,b) with autoimmunity. The variant lies within the conserved ABIN-homology domain 3 (AHD3) (Fig. 1c,d); the proline was predicted to be structurally and functionally damaging based on AlphaFold2, PyMOL (Fig. 1e), SIFT, PolyPhen-2 and CADD (Extended Data Fig. 1b).

A.III.2 was an 8-year-old White Australian girl who presented with sicca symptoms, mouth ulcers, malar rash, fatigue and arthralgia (Fig. 1a and Supplementary Table 1). She developed chronic sterile cystitis, gastroparesis and neutropenia, which responded to immune-modulating treatment (Supplementary Table 1). Serological investigations revealed evidence of autoimmune hypothyroidism (antithyroglobulin antibody-positive) and low titer antinuclear antibody (ANA) (1:80). She had polyclonal hypergammaglobulinemia at presentation (20 g l^−1^; approximately 25% above the upper limits of normal 6.2–14.4 g l^−1^) that persisted throughout her illness. Her hypergammaglobulinemia was partially accounted for by a mean elevation of IgG4 to 3.07 g l^−1^ (twice the upper limit of normal (0.04–1.36 g l^−1^)) (Fig. 1f) and a mean IgG4:IgG ratio of approximately 0.08–0.10, which was sustained despite treatment. The proband’s mother (A.II.1; Fig. 1a) was antithyroglobulin antibody-positive and under investigation for CREST syndrome after autoimmune serology revealed a high titer of centromere antibodies (1:5,120). The proband’s father (A.II.2) was unaffected and lacked the variant (Extended Data Fig. 1c). B.II.1 was a 47-year-old Chinese Han female diagnosed with systemic lupus erythematosus (SLE) featuring ANAs, anti-double-stranded DNA, anti-Ro (Sjögren's disease (SjD)-related antigen A (SSA)) and antiphospholipid antibodies, arthritis, proteinuria (urinary protein more than 500 g per 24 h), and hypergammaglobulinemia of IgG, IgG1, IgG4 and IgE, with an IgG4:IgG ratio of 0.14 (Fig. 1f and Supplementary Table 1). Additional rare variants in known lupus-causing genes or genes causing monogenic autoimmunity were not identified.

### Autoimmunity in mice with the orthologous *Tnip1*^*Q346P*^ variant

**Extended Data Fig. 2:**
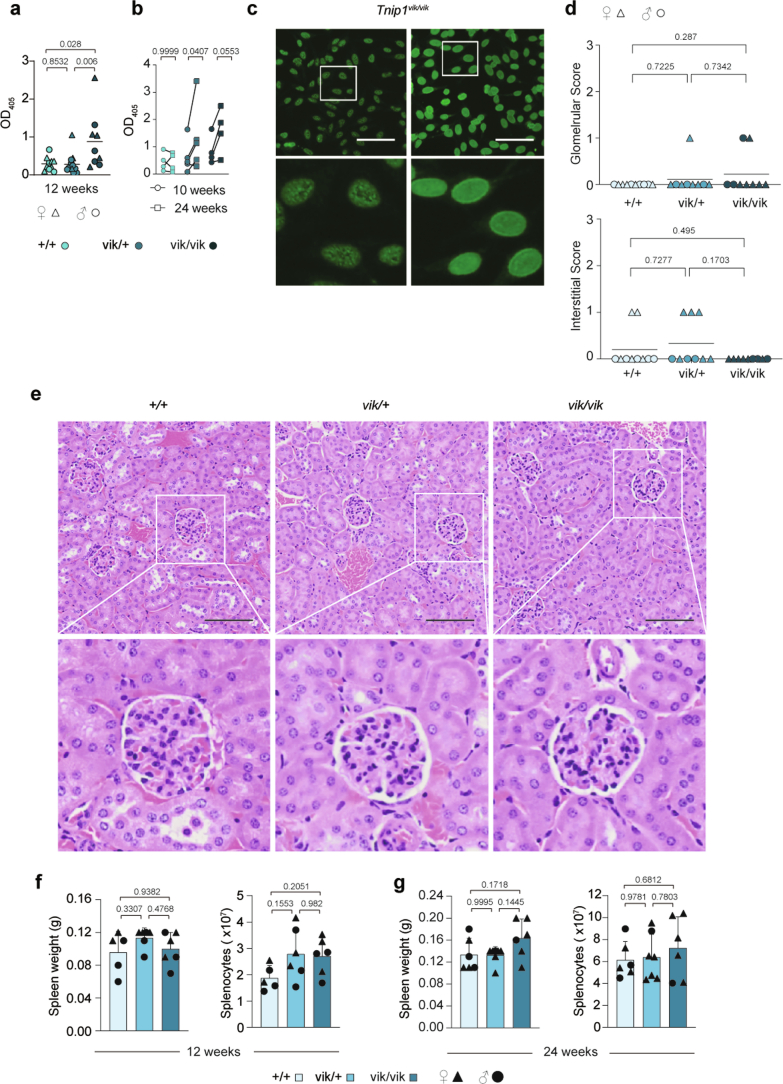
Anti-DNA antibodies increase with age but do not cause glomerulonephritis or splenomegaly in *vikala* mice. **a**, **b**, Serum antibodies to DNA from 12 week-old *vikala* mice. OD405, optical density at 405 nm, bars represent the mean values and each point represents a mouse (**a**) and comparison of relative optical densities at 10 and 24 week-old mice (**b**). OD405, optical density at 405 nm. (b). **c**, Hep-2 immunofluorescence showing ANA staining patterns in 12-week-old *Tnip1*^*vik*/*vik*^ mice. Scale bars represent 50 μm. **d**, Glomerular and interstitial damage scores assessed from hematoxylin and eosin (H&E) stained kidney sections from *vikala* mice; *Tnip1*^*+/+*^ *(n* = *10), Tnip1*^*vik/+*^*(n* = *9), Tnip1*^*vik/vik*^ *(n* = *9)* at 20–28 weeks of age. Means indicated by black line, each point represents a mouse. **e**, Representative H&E stained *vikala* kidney tissue scored in panel d; upper panel shows one representative image of kidney tissue from *Tnip1*^*+/+*^ *(n* = *10), Tnip1*^*vik/+*^*(n* = *9), Tnip1*^*vik/vik*^ *(n* = *9)*; lower panel shows an expanded view of the region enclosed in white, featuring glomeruli. Scale bars represent 50 μm, original magnification 20x. **f**, **g**, mean spleen weight and total cellularity of 12 week-old (*Tnip1*^*+/+*^ *(n* = *5), Tnip1*^*vik/+*^*(n* = *6), Tnip1*^*vik/vik*^ *(n* = *6)* (**f**), and 24 week-old mice *Tnip1*^*+/+*^ *(n* = *6), Tnip1*^*vik/+*^*(n* = *7), Tnip1*^*vik/vik*^ *(n* = *6)* (**g**), respectively; each dot represents a mouse (biological replicate) and error bars indicate SD. Sexes are indicated by symbol type. Data representative of *n* = *2* (**a, f-g**). Data in panel (**b-e**) are from experiments performed once. Statistical significance was performed using one-way ANOVA with Tukey multiple-comparison test following log-transformation of data (**a, d,f-g**) and two-way ANOVA with Sidak’s multiple comparison test (**b**). Exact *P* values are shown. Source data

To investigate the impact of *TNIP1*^*Q333P*^ on autoimmunity, we generated CRISPR–Cas9-edited C57BL/6 mice carrying the orthologous variant (Q346P (*Tnip1*^*Q346P*^); Extended Data Fig. 1d), referred to as *vikala* mice and the ‘*vik’* allele. Anti-DNA autoantibodies were detectable in homozygous (*Tnip1*^*vik*/*vik*^)mice at 12 weeks, but not in heterozygous (*Tnip1*^*vik*/+^) mice (Extended Data Fig. 2a). By 20–28 weeks, *Tnip1*^*vik*/+^ mice had also developed anti-DNA antibodies (Fig. 1g and Extended Data Fig. 2b), with levels being either slightly lower or comparable to those in *Tnip1*^*vik/vik*^ mice (Fig. 1g).

While total IgG levels were not significantly elevated in *vikala* mice, 20-week-old *vikala* mice had elevated IgG2c antibodies (Fig. 1h,i). Homogenous nuclear and speckled antinuclear antibodies were detected in *Tnip1*^*vik/vik*^ mice (Extended Data Fig. 2c). Hematoxylin and eosin (H&E) staining showed multifocal lymphocytic sialadenitis in *Tnip1*^*vik/+*^ and *Tnip*1^*vik/vik*^ mice (Fig. 1j,k). *Vikala* mice did not develop glomerular damage or splenomegaly (Extended Data Fig. 2d–g). Exocrinopathy has not been reported in *Tnip1*^*D485N*^ mice, which instead develop glomerulonephritis^7^, indicating that the *Tnip1*^*Q346P*^ variant is pathogenic and acts differently to the previously described D485N allele.

### Cell-intrinsic expansion of B cell subsets in *vikala* mice

**Fig. 2:**
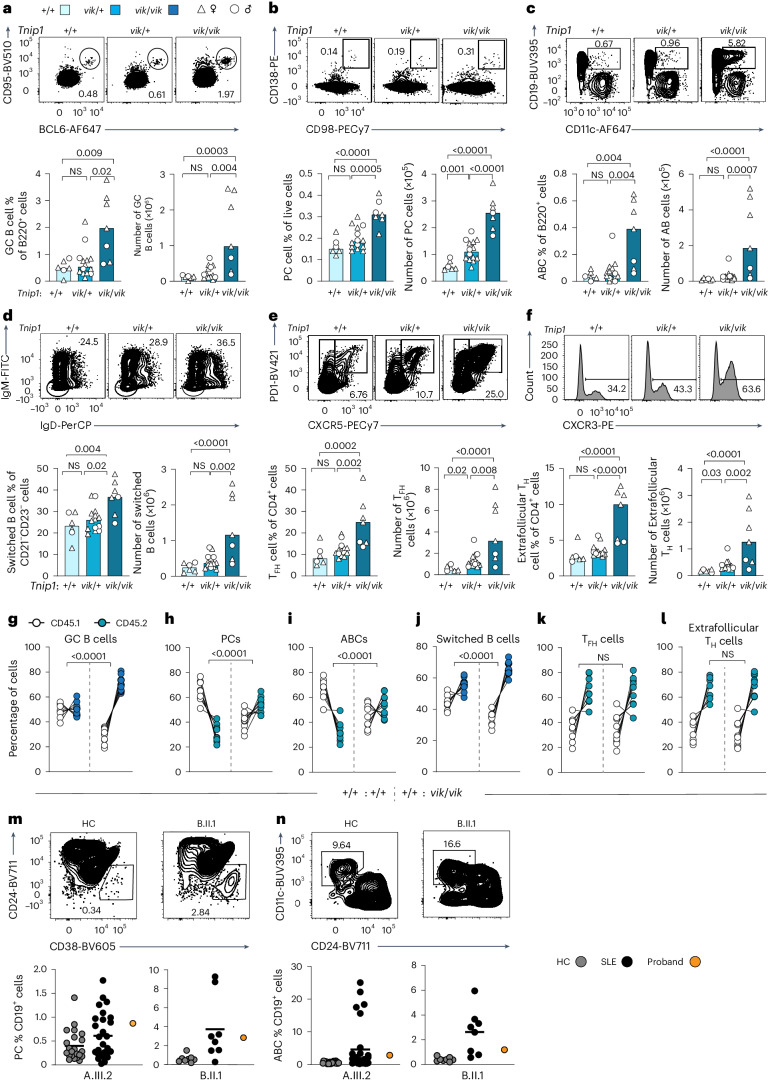
*Vikala* mice develop cell-intrinsic expansion of activated B and T immune cell subsets. **a**–**f**, Flow cytometry plots and quantification of splenocytes from 22–28-weeks-old male (*n* = 8) and female (*n* = 19) *vikala* mice; *Tnip1*^+/+^ (*n* = 6), *Tnip1*^*vik*/+^ (*n* = 14) and *Tnip1*^*vik*/*vik*^ (*n* = 7): GC B cells (CD19^+^CD95^+^BCL6^+^) (**a**); PCs (CD138^+^CD98^+^) (**b**); ABCs (B220^+^CD21^−^CD23^−^CD19^high^CD11c^+^) (**c**); switched B cells (B220^+^CD21^−^CD23^−^IgD^−^IgM^−^) (**d**); T_FH_ cells (CD4^+^CXCR5^+^PD1^high^) (**e**); and extrafollicular T_H_ cells (CD4^+^CXCR5^−^PD1^+^CXCR3^+^) (**f**). **g**–**l**, Splenic phenotypes of 26–30-week-old chimeric mice 16 weeks after reconstitution with a 1:1 ratio of control *Tnip1*^+/+^ CD45.1:*Tnip1*^+/+^ CD45.2 (*n* = 7) or *Tnip1*^+/+^ CD45.1:*Tnip1*^*vik*/*vik*^ CD45.2 (*n* = 8) BM cells. All irradiated *Rag*^*-/-*^ recipient mice were female. **g**, GC B cells. **h**, PCs. **i**, ABCs. **j**, Switched B cells. **k**, T_FH_ cells. **l**, Extrafollicular T_H_ cells. **m**,**n**, Representative flow cytometry plots and quantification of the PC (**m**) and ABC (**n**) phenotype in PBMCs from A.III.2 or B.II.1, healthy controls (*n* = 23, *n* = 8) and individuals with SLE (*n* = *27*, *n* = 8), respectively. The bars in **a**–**f** or the black lines in **m**,**n** represent the median values; each dot represents an individual mouse or human blood donor (biological replicate). In **a**–**f**, data are representative of *n* = 2 experiments. In **g**–**n**, data are from experiments performed once. Statistical analysis was performed using a one-way ANOVA with Tukey’s correction for multiple comparisons (**a**–**f**) and a two-way ANOVA (**g**–**l**). Exact *P* values are shown. Source data

**Extended Data Fig. 3:**
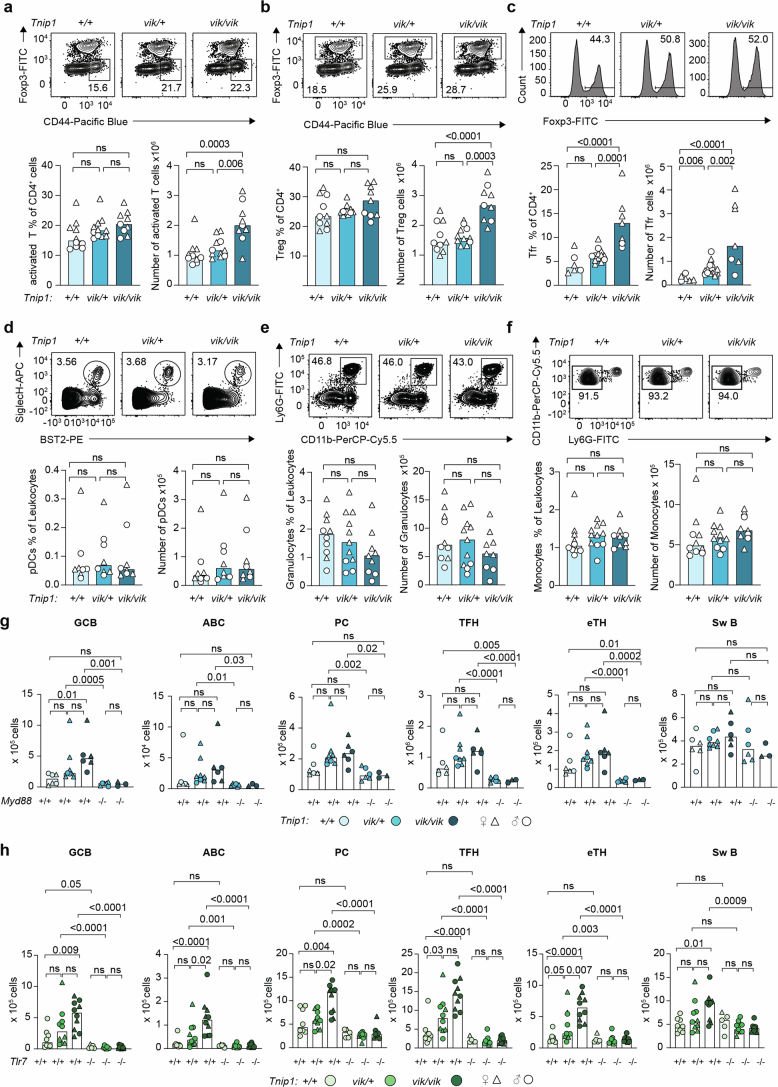
*Myd88* and *Tlr7* deficiency rescues abnormal total splenocyte phenotypes in *vikala* mice. **a-f**, Flow cytometric quantification of proportion and total numbers of activated CD4 T cells (CD3^+^CD4^+^CD44^+^Foxp3^-^) (**a**); and T regulatory cells (Treg; CD3^+^CD4^+^CD44^+^Foxp3^+^) (**b**) from 20–28 week-old *vikala* mice *Tnip1*^*+/+*^ *(n* = *10), Tnip1*^*vik/+*^*(n* = *11), Tnip1*^*vik/vik*^ *(n* = *9)*; T follicular regulatory cells (Tfr; CD4^+^PD1^+^CXCR5^+^Foxp3^+^) (**c**) from 22–28 week-old *vikala* mice; *Tnip1*^*+/+*^*(n* = *6), Tnip1*^*vik/+*^*(n* = *14)* and *Tnip1*^*vik/vik*^ *(n* = *7)*; plasmacytoid dendritic cells (pDCs; CD3^−^CD19^−^CD8^−^CD11c^+^CD11b^-^SiglecH^+^BST2^+^) (**d**) from 20 week-old *vikala* mice (*Tnip1*^*+/+*^ *(n* = *8), Tnip1*^*vik/+*^*(n* = *8), Tnip1*^*vik/vik*^ *(n* = *8)*; and granulocytes (Ly6C^+^ Ly6G^+^CD11b^+^) and monocytes (CD11c^-^CD11b^+^Ly6G^-^) from 20–28 week-old *vikala* mice (*Tnip1*^*+/+*^ *(n* = *10), Tnip1*^*vik/+*^*(n* = *11), Tnip1*^*vik/vik*^ *(n* = *9)* (**e-f)**. **g-h**, Flow cytometric quantification of splenic cell subsets (total number) in 16–20 or 20–30 week-old *vikala* mice either competent or deficient in *MyD88* (**g**) and *Tlr7* (**h**), respectively. Subsets from left to right; germinal center B cells (GCs; CD19^+^CD95^+^BCL6^+^); age associated B cells (ABC; B220^+^CD21^−^CD23^−^CD19^high^CD11c^+^), plasma cells (PC; CD138^+^CD98^+^), T follicular helper cells (Tfh; CD4^+^CXCR5^+^PD1^high^), extrafollicular helper T cells (eT_H_; CD4^+^CXCR5^−^PD1^+^CXCR3^+^), and switched B cells (B220^+^CD21^−^CD23^−^IgD^−^IgM^−^). Bars represent the median values, and each dot represents an individual mouse (biological replicate) with males and females represented by circles and triangles, respectively (**a-h**). Data presented in **a-b** and **e-f** are all from the same experimental cohort of 20–28 week old *vikala* mice. Data presented in **c** and **d** are each from separate experimental cohorts of 20-week-old and 22–28-week-old *vikala* mice, respectively. **a-h**, Results are representative n = 2 experiments. *P* values were determined by one-way ANOVA with Tukey's test for three-way comparisons following log-transformation of data. ns, not significant. Source data

Abnormalities in immune cell compartments were assessed in splenocytes from unchallenged *vikala* mice (Fig. 2a–f and gating in Supplementary Fig. 1). *Tnip1*^*vik/vik*^ mice had increased proportions and numbers of spontaneous germinal centers (GCs), plasma cells (PCs), age-associated B cells (ABCs) and CD21^−^CD23^−^ switched B cells (Fig. 2a–d). Total T follicular helper cells (T_FH_) and CXCR3^+^ extrafollicular T_H_ cells were increased in both *Tnip1*^*vik/+*^ and *Tnip1*^*vik/vik*^ mice compared to wild-type (WT) littermates (Fig. 2e,f). *Tnip1*^*vik/vik*^ *vikala* mice also had significantly increased activated T cells, T regulatory and T follicular regulatory (T_FR_) cell numbers but plasmacytoid dendritic cells (pDCs), granulocytes and monocytes were normal (Extended Data Fig. 3a–f).

To determine whether cellular phenotypes were cell-intrinsic, we generated mixed bone marrow (BM) chimeras; 1:1 mixes of BM from WT CD45.1 with *Tnip1*^*vik/vik*^ *vikala* CD45.2 or *Tnip1*^*+/+*^ CD45.2 BM were transferred into sublethally irradiated *Rag1*^−/−^ mice. Analysis of splenic immune cell populations 16 weeks after reconstitution identified a cell-autonomous expansion of GC B cells, PCs, ABCs and switched B cells (Fig. 2g–j). By contrast, the T cell changes were cell-extrinsic (Fig. 2k,l).

The composition of peripheral blood mononuclear cells (PBMCs) from the probands was compared to the healthy and SLE-affected cohorts. A.III.2 and B.II.1 had increased ABCs and PCs compared to non-SLE healthy controls (HCs), a finding also seen in other patients with SLE (Fig. 2m,n and gating in Supplementary Fig. 2). Together these findings indicate that TNIP1^Q333P^ signals in B cells to increase the subsets associated with systemic autoimmunity.

### Cellular phenotypes in *vikala* mice are MyD88-TLR7 dependent

**Fig. 3:**
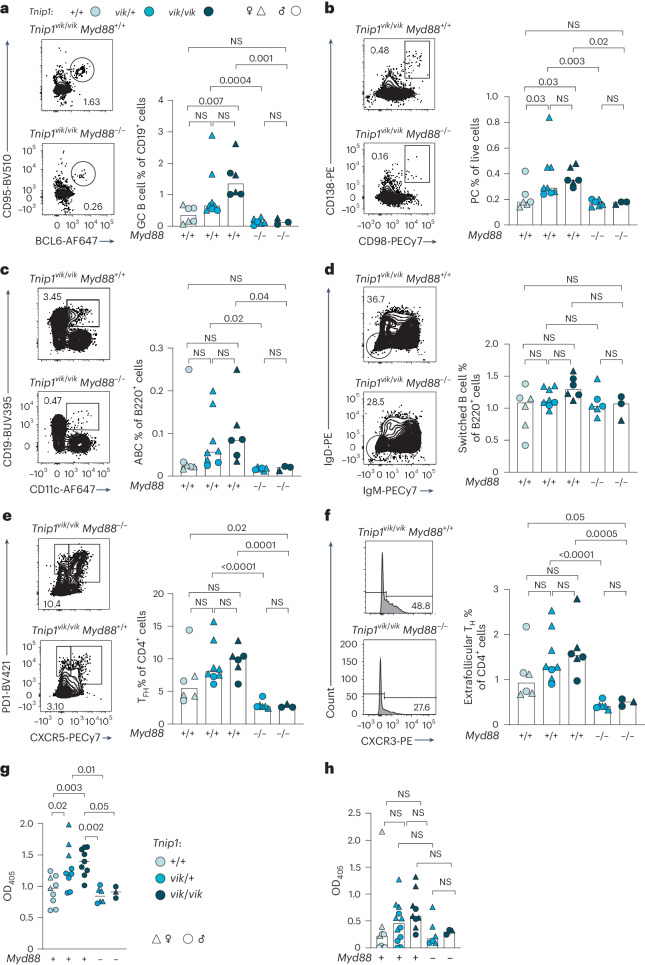
TNIP1-driven cellular phenotypes are dependent on MyD88 signaling. **a–f**, Representative flow cytometry plots and proportions of splenocytes from male (*n* = 12, dots) and female (*n* = 17, triangles) *vikala* mice aged 16–20 weeks: GC B cells (CD19^+^CD95^+^BCL6^+^) (**a**); PCs (CD138^+^CD98^+^) (**b**); ABCs (B220^+^CD21^−^CD23^−^CD19^high^CD11c^+^) (**c**); switched B cells (B220^+^CD21^−^CD23^−^IgD^−^IgM^−^) (**d**); T_FH_ cells (CD4^+^CXCR5^+^PD1^high^) (**e**); and extrafollicular T_H_ cells (CD4^+^CXCR5^−^PD1^+^CXCR3^+^) (**f**), either competent or deficient in *Myd88*. *Tnip1*^+/+^ (*n* = 6), *Tnip1*^*vik*/+^ (*n* = 8), *Tnip1*^*vik*/*vik*^ (*n* = 6), *Tnip1*^*vik*/+^ *Myd88*^−/−^ (*n* = 6) and *Tnip1*^*vik*/*vik*^ *Myd88*^−/−^ (*n* = *3*). The bars represent the median values; each point represents an individual mouse (biological replicate). **g**, serum IgG2c antibodies in 16–20-week-old *vikala* mice either competent or deficient in *Myd88*; *Tnip1*^+/+^ (*n* = 9), *Tnip1*^*vik*/+^ (*n* = 9), *Tnip1*^*vik*/*vik*^ (*n* = 9), *Tnip1*^*vik*/+^ *Myd88*^−/−^ (*n* = 6) and *Tnip1*^*vik*/*vik*^ *Myd88*^−/−^ (*n* = 3). The black lines represents the median; each point represents an individual mouse (biological replicate); sex is indicated by the respective symbol. **h**, Serum antibodies to DNA (ANAs) in 16–20-week-old *vikala* mice either competent or deficient in *Myd88;* *Tnip1*^+/+^ (*n* = 8), *Tnip1*^*vik*/+^ (*n* = 12), *Tnip1*^*vik*/*vik*^ (*n* = 9), *Tnip1*^*vik*/+^ *Myd88*^−/−^(*n* = 6) and *Tnip1*^*vik*/*vik*^ *Myd88*^−/−^ (*n* = 3). The bars represent the median; each point represents an individual mouse (biological replicate). In **a**–**f**, data are representative of *n* = 2 independent experiments. In **g**,**h**, data are from experiments performed once. Statistical significance was performed using a one-way ANOVA with Tukey’s correction for multiple comparison after log-transformation of data. Exact *P* values are shown. Source data

**Fig. 4:**
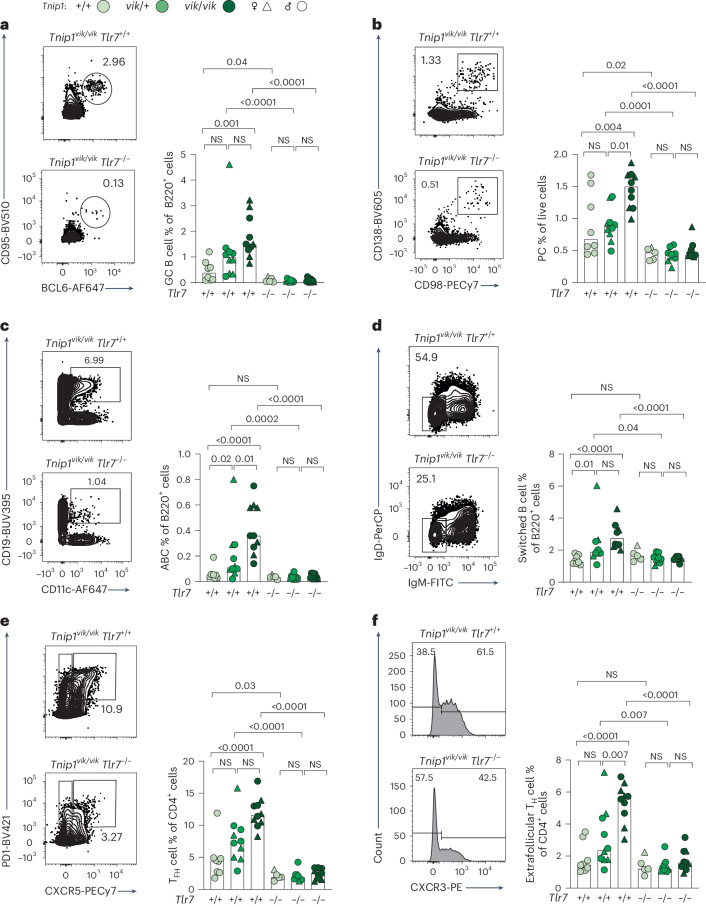
TNIP1-driven cellular phenotypes are dependent on TLR7 signaling. **a**–**f**, Representative flow cytometry plots and proportions of splenocytes from male (*n* = 34, dots) and female (*n* = 17, triangles) *vikala* mice aged 20–30 weeks: GC B cells (CD19^+^CD95^+^BCL6^+^) (**a**); PCs (CD138^+^CD98^+^) (**b**); ABCs (B220^+^CD21^−^CD23^−^CD19^high^CD11c^+^) (**c**); switched B cells (B220^+^CD21^−^CD23^−^IgD^−^IgM^−^) (**d**); T_FH_ cells (CD4^+^CXCR5^+^PD1^high^) (**e**); and extrafollicular T_H_ cells (CD4^+^CXCR5^−^PD1^+^CXCR3^+^) (**f**) either competent or deficient in *Tlr7*; *Tnip1*^+/+^ (*n* = 8), *Tnip1*^*vik*/+^ (*n* = 10), *Tnip1*^*vik*/*vik*^ (*n* = 10), *Tnip1*^*vik*/+^ *Tlr7*^−/−^ (*n* = 9), *Tnip1*^*vik*/*vik*^ *Tlr7*^−/−^ (*n* = 9) and *Tnip1*^+/+^ *Tlr7*^−/−^ (*n* = 5). The bars represent the median values; each symbol represents an individual mouse (biological replicate). Data are representative of *n* = 2 independent experiments. Statistical significance was performed using a one-way ANOVA with Tukey’s correction for multiple comparisons after log-transformation of the data. Exact *P* values are shown. Source data

TLR7 signaling via its adapter MyD88 is important in the development of SLE^2,13^. As TNIP1 is recruited to activated MyD88 signaling complexes^8^, and *Tnip1*^*D485N*^-mediated autoimmunity is MyD88-dependent^7^, we investigated a pathogenic role for this pathway by crossing *vikala* mice to mice lacking MyD88 or TLR7. Increases in total GC B cells and frequencies of PCs and ABCs were rescued with MyD88 ablation, however reductions in CD21^−^CD23^−^ switched B cells were not statistically significant (Fig. 3a–d and Extended Data Fig. 3g). T_FH_ and extrafollicular T_H_ cells were reduced with MyD88 ablation (Fig. 3e–f and Extended Data Fig. 3g). IgG2c antibodies were also significantly reduced (Fig. 3g). While the same reduction trend was seen for anti-DNA antibodies, it failed to reach statistical significance (Fig. 3h). Crosses to mice lacking TLR7 rescued all cellular phenotypes (Fig. 4a–f and Extended Data Fig. 3h). Our data show a role for the TLR7-MyD88 signaling pathway in the autoimmune cellular phenotypes of *vikala* mice.

### TNIP1^Q333P^ is impaired in repressing interferon-β signaling

**Fig. 5:**
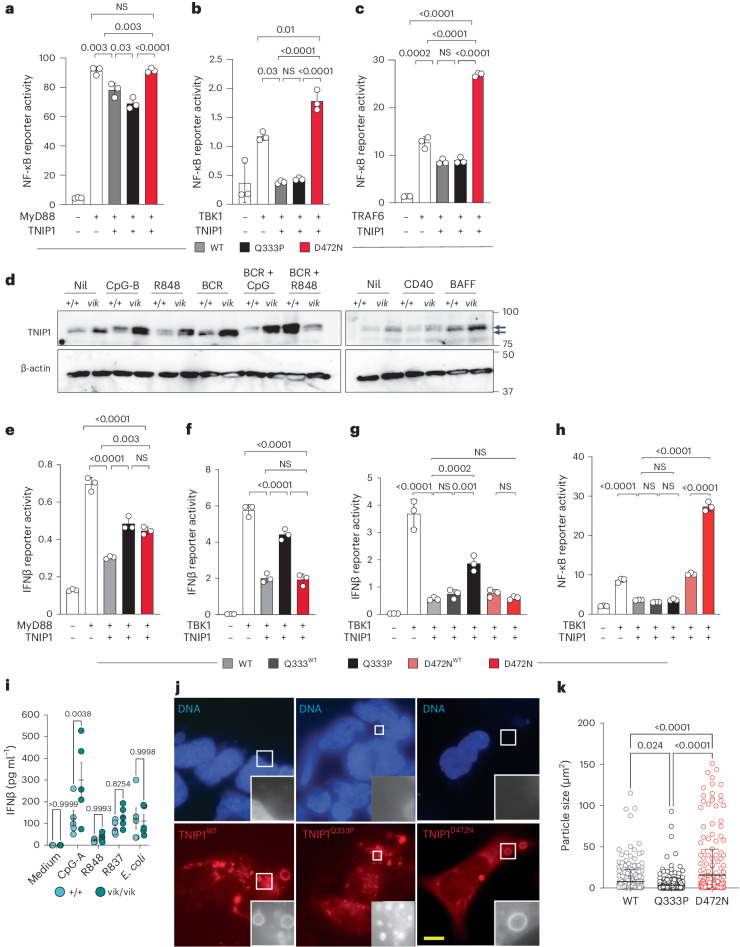
TNIP1^Q333P^ regulates NF-κB signaling but not IFNβ and alters the size of TNIP1 puncta. **a**–**c**, NF-κB activity (ratio of NF-κB firefly to *Renilla* luciferase in relative light units) 24 h after lipofectamine transfection of human TNIP1 plasmids (WT, Q333P and D472N) into HEK 293 cells cotransfected with MyD88 (**a**), TBK1 (**b**) and TRAF6 (**c**). Data are shown as the mean and s.d. of *n* = 3 biological replicates or transfections, shown as individual dots and representative of three independent experiments (**a**–**c**). **d**, Immunoblots (IBs) of splenic B cell lysates from 20-week-old WT (+/+) and *vikala* homozygote (*vik*/*vik*) mice probed for TNIP1 and actin proteins after stimulation with CpG-B, R848, BCR (αIgM), BCR and CpG-B, BCR and R848, CD40 and BAFF. Data are representative of two independent experiments. **e**–**h**, IFNβ (**e**–**g**) and NF-κB (**h**) activity (ratio of IFNβ or NF-κB firefly to *Renilla* luciferase in relative light units) 24 h after lipofectamine transfection with human TNIP1 plasmids (WT, Q333P and D472N) into HEK 293 cells cotransfected with MyD88 (**e**) or TBK1 (**f**–**h**). Data are shown as the mean and s.d. of *n* = 3 biological replicates or transfections shown as individual dots and representative of three independent experiments (**e**–**h**). Statistical significance was calculated using a one-way ANOVA with Tukey’s correction for multiple comparisons (**a**–**c**,**e**–**h**). Exact *P* values are shown. **i**, ELISA-determined serum levels of IFNβ protein from *vikala* BM-pDCs; *Tnip1*^+/+^ (*n* = 5) and *Tnip1*^*vik*/*vik*^ (*n* = 5), untreated or stimulated with CpG-A, R848, R837 and *Escherichia coli*. Data are representative of three experiments. The error bars indicate the mean with the s.e.m.; each dot represents a single mouse (biological replicate). **j**, Immunofluorescence staining of HEK 293 cells expressing TNIP1^WT^, TNIP1^Q333P^ or TNIP1^D472N^. **k**, Quantification of WT (*n* = 301), Q333P (*n* = 305) and TNIP1^D472N^ (*n* = 268) mean particle size. Scale bar, 10 μm. Data are representative of *n* = 3 independent experiments. Statistical significance was performed using a two-way ANOVA with Šidák’s correction for multiple comparisons (**i**) and a one-way ANOVA with Tukey’s correction for multiple comparisons (**k**). The error bars indicate the mean with the s.d. Exact *P* values are shown. Source data

**Extended Data Fig. 4:**
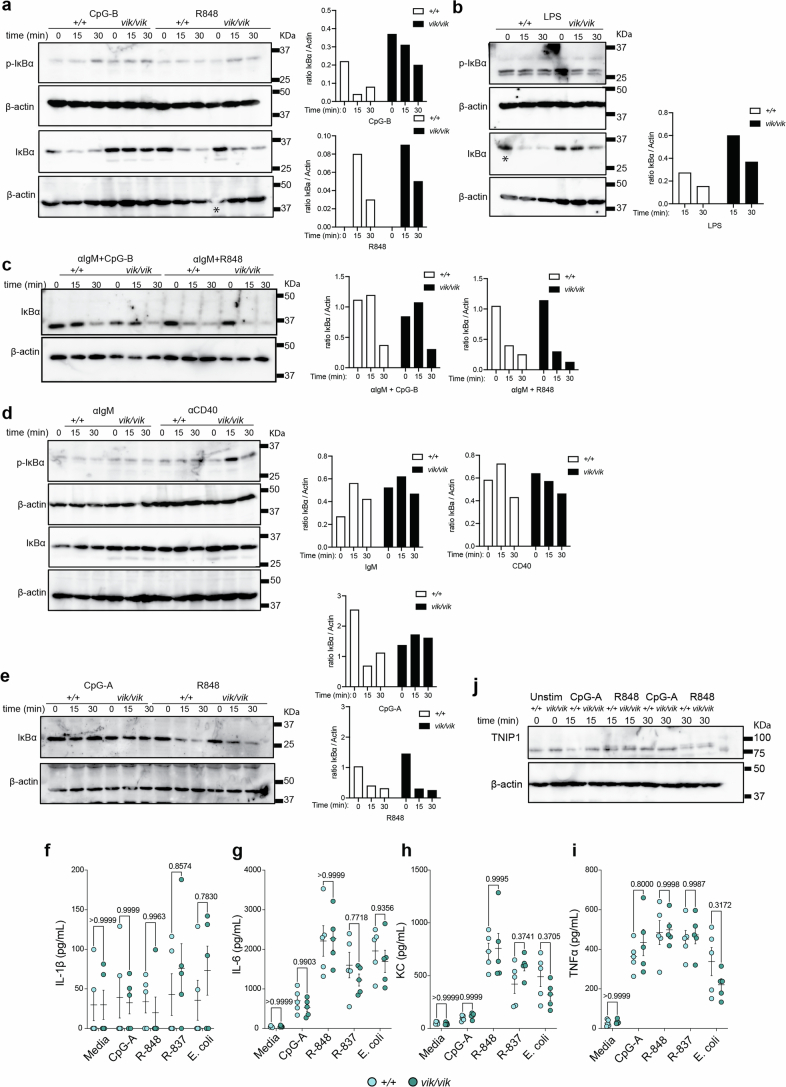
NF-κB-dependent signaling and cytokine secretion from splenocytes and bone marrow derived pDCs is not increased *in vikala* mice. **a-d**, Immunoblots of splenic B cell lysates from *Tnip1*^*+/+*^ *(n* *=* *1)* and *Tnip1*^*vik/vik*^ *(n* *=* *1)* mice probed for TNIP1, IκBα and Actin proteins after stimulation with CpG-B, R848 (**a**), LPS (**b**), BCR+CpG-B, BCR+R848 (**c**), BCR (αIgM) and CD40 (**d**). B cells from 40-week (**a, d**), 10–12-week (**b**), and 28–29-week old mice (**c**). (Densitometric ratio of IκBα to Actin loading control in stimulated wildtype (*+/+*) and *vikala* homozygote (*vik/vik*) B cells shown (right) (**a-d**). Asterisks indicate poor quality bands that were prohibitive to accurate quantification. **e**, **j**, Immunoblots of bone marrow derived pDCs from 16–20 week-old *Tnip1*^*+/+*^ *(n* *=* *1)* and *Tnip1*^*vik/vik*^ *(n* *=* *1)* mice probed for IκBα (**e**), TNIP1 (**j**) and Actin proteins after stimulation with CpG-A and R848. Densitometric ratio of IκBα to Actin loading control shown (**e**). **f**-**i**, Bone marrow derived pDCs from 16–20 week-old *Tnip1*^*+/+*^ *(n* *=* *1)* and *Tnip1*^*vik/vik*^ *(n* *=* *1)* mice were seeded in 96 well plates and treated with either CpG-A, R848, R837 or infected with E.coli. Supernatants were harvested for quantification by ELISA at 6 h (**f-i**) post treatment. Bars represent means with standard error of the mean (SEM) and each dot a single mouse (biological replicate). Two-way ANOVA with Sidak’s multiple comparisons; ns, not significant. Data in panels (**a-e, j**) are representative of experiments performed twice and (**f-i**) from experiments performed three times. Source data

Overactive NF-κB signaling contributed to the increased proinflammatory milieu central to the pathogenesis reported in the *Tnip1*^D485N^ model^7^. Therefore, we explored whether TNIP1^Q333P^ drove excess NF-κB signaling using a luciferase reporter assay and coexpressing either MyD88, TRAF6 or TBK1 to activate signaling in HEK 293 cells. Surprisingly, unlike TNIP1^D472N^ (the D485N ortholog in human TNIP1), the Q333P variant did not impair TNIP1’s inhibition of NF-κB signaling, behaving comparably to TNIP1^WT^ (Fig. 5a–c). This was corroborated by measuring phosphorylated or total IκBα—an NF-κB-dependent kinase—by immunoblotting of purified *vikala* B cells. *Vikala* B cells and BM-derived pDCs (BM-pDCs) were also treated with the NF-κB-inducing TLR ligands CpG-B, R848 and lipopolysaccharide (LPS) alone (Extended Data Fig. 4a,b), or together with anti-IgM (αIgM) (Extended Data Fig. 4c), or with αIgM or αCD40 alone (Extended Data Fig. 4d). The kinetics of NF-κB activation based on the degradation (on phosphorylation) of IκBα were similar for WT and *vikala* B cells and BM-pDCs under almost all conditions, with no evidence of decreased total IκBα in *vikala* cells (Extended Data Fig. 4e). Furthermore, NF-κB-driven cytokines (interleukin-1β, interleukin-6, TNF and CXCL1/KC) from stimulated BM-pDCs were comparable in *Tnip1*^*+/+*^ and *Tnip1*^*vik/vik*^ mice (Extended Data Fig. 4f–i). TLR signaling triggered by CpG-B and R848, but not B cell receptor (BCR) and CD40, activated TNIP1, which was detected as a higher molecular weight band^11^ in B cell lysates from mice (Fig. 5d). In BM-pDCs, R848 but not CpG-A induced TNIP1 activation (Extended Data Fig. 4j).

TNIP1 inhibits interferon-β (IFNβ) promoter activity in response to viral infection or poly(I:C) transfection^14^. As TNIP1 associates with the activated MyD88 signaling complexes^8^ and *Myd88* ablation rescued the *vikala* B cell phenotypes, we asked whether TNIP1 represses the MyD88-dependent IFN1 pathway that induces IFNβ production. As expected, TNIP1^WT^ effectively repressed IFNβ luciferase activity (Fig. 5e). By contrast, coexpression of MyD88 with the TNIP1^Q333P^ and TNIP1^D472N^ constructs impaired repression (Fig. 5e). Strikingly, the TNIP1^Q333P^ variant also impaired repression of TBK1-induced IFNβ activity while the TNIP1^D472N^ variant retained repressive capacity comparable to that of the TNIP1^WT^ construct (Fig. 5f,g).

To address whether the TNIP1^Q333P^ protein exerts a dominant-negative effect, TBK1 was coexpressed with 50:50 ratios of *TNIP1* alleles in both the IFNβ and NF-κB luciferase reporter assays. The D472N and Q333P variants resulted in TNIP1 haploinsufficiency because TNIP1^WT^ largely restored the repressive activity of both mutant proteins in our reporter assays (Fig. 5g,h). IFNβ protein was also increased in CpG-A-stimulated *Tnip1*^*vik/vik*^ BM-pDCs compared to *Tnip1*^*+/+*^, with only a trend observed for R848 or R837 stimulation (Fig. 5i). In summary, our data show that TNIP1^Q333P^ selectively impacts IFN1 signaling.

### TNIP1^Q333P^ fails to localize to autophagosomes

**Extended Data Fig. 5:**
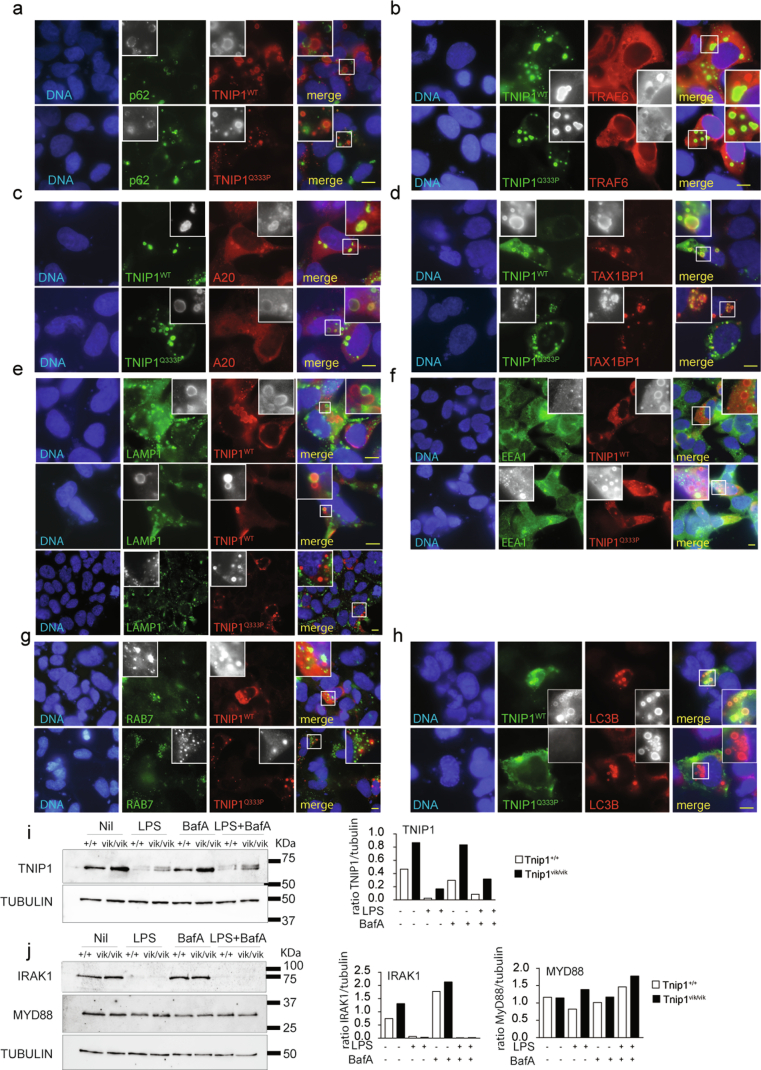
TNIP1Q333P localization with signaling partners and Myddosome component turnover. **a–h**, Immunofluorescence on HEK293 cells for TNIP1 and p62 (green; **a**), TRAF6 (red; **b**), A20 (red; **c**), TAX1BP1 (red, **d**), LAMP1 (green; **e**) EEA1 (green; **f**), RAB7 (green; **g**) and LC3B (red; **h**). Scale bars represent 10 μm. Images are representative of *n* = *3* (**b**), *n* = *2* (**a, c-f, h**) and *n* = *1* **(g)** independent experiments. **i-j**, Immunoblotting of TNIP1 (**i**), IRAK1 and MYD88 (**j**) protein in *Tnip1*^*+/+*^ and *Tnip1*^*vik/vik*^ BMDMs treated with LPS (100 ng/mL) and Bafilomycin A1 (100 nM) for 2 h. Densitometric plots showing levels of each protein relative to the loading control are shown (right). Data representative of n = 2 (**i-j**) experiments. Source data

We next sought to understand the mechanism by which TNIP1^Q333P^ selectively alters MyD88 signaling. First, we investigated whether the variant altered subcellular localization by overexpressing TNIP1 in HEK 293 cells. As previously noted^11,15,16^, TNIP1^WT^ localized as cytoplasmic puncta, but these were significantly smaller in cells expressing TNIP1^Q333P^ (Fig. 5j,k). By comparison, TNIP1^D472N^ showed larger puncta (Fig. 5j). The selective autophagy receptor p62/sequestosome-1, which captures TNIP1’s binding partner A20 within autophagosomes forms similar cytoplasmic puncta^17^. Costaining cells expressing TNIP1 with a p62 antibody revealed minimal colocalization with a few puncta (Extended Data Fig. 5a), suggesting that most TNIP1 puncta are not bona fide sequestosomes. Consistent with this, TRAF6, which colocalizes to p62^+^ sequestosomes^18^ was recruited to only some of the TNIP1 puncta (Extended Data Fig. 5b).

As expected, TNIP1 showed colocalization with A20 and its partner Tax1-binding protein 1 (TAX1BP1) (Extended Data Fig. 5c,d), but minimal costaining with LAMP1^+^ lysosomal compartments under basal conditions (Extended Data Fig. 5e) and no colocalization with the endosomal markers EEA1 and RAB7 (Extended Data Fig. 5f,g).

**Fig. 6:**
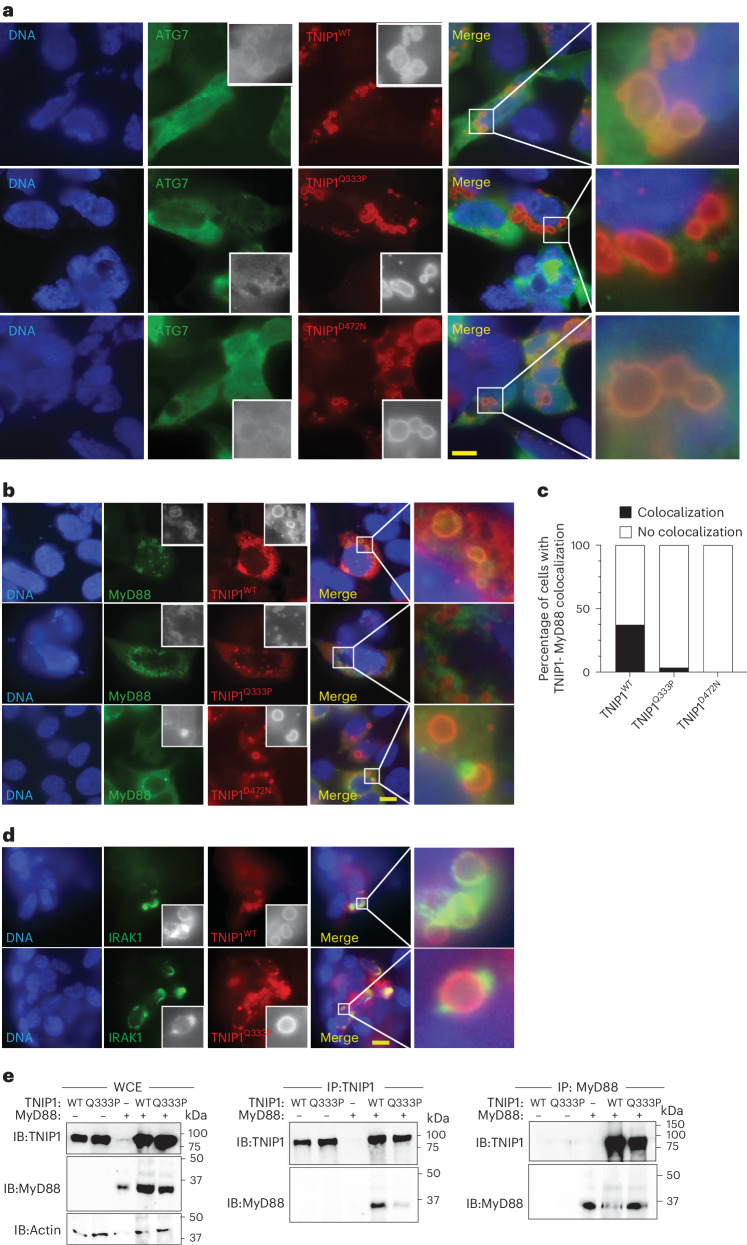
TNIP1^Q333P^ has impaired colocalization with the autophagosome markers ATG7, MyD88 and IRAK1. **a**,**b**,**d**, Immunofluorescence staining of HEK 293 cells expressing TNIP1^WT^, TNIP1^Q333P^ or TNIP1^D472N^ (red) and ATG7 (green) (**a**), or MyD88 (**b**) or IRAK1 (**d**) (green). DNA was stained with 4′,6-diamidino-2-phenylindole (DAPI). Scale bar, 10 μm. Data are representative of *n* = 3 (**a**,**b**) or *n* = 2 (**d**) independent experiments. Scale bar, 10 μm. **c**, Quantification of the percentage of TNIP1^WT^, TNIP1^Q333P^ and TNIP1^D472N^-expressing cells showing localization (black bar) or lack of localization (white bar) with MyD88 within puncta. Data are from one experiment of *n* = 21 (WT), *n* = 32 (Q333P) and *n* = 35 (D472N). TNIP1-expressing cells were imaged. In **c**, data are representative of *n* = 3 independent experiments. **e**, Anti-TNIP1 or anti-MyD88 IBs of overexpressed and immunoprecipitated TNIP1^WT^, TNIP1^Q333P^ or MyD88 in HEK 293T cells. In **e**, data are representative of n = 2 independent experiments. WCE, whole-cell extract. Source data

Phosphorylated TNIP1 colocalizes with the autophagosome marker LC3B and target ubiquinated Myddosome proteins for autophagic degradation^11^. Staining for the ATG7 and LC3B autophagosome markers revealed that the larger TNIP1 puncta were indeed autophagosomes (Fig. 6a and Extended Data Fig. 5h); TNIP1^Q333P^ had impaired localization to both ATG7^+^ and LC3B^+^ autophagosomes (Fig. 6a and Extended Data Fig. 5h). Additionally, TNIP1^D472N^ was unimpaired in its colocalization with ATG7^+^ autophagosomes (Fig. 6a), aligning with our observed lack of reduction of puncta size compared with TNIP1^WT^ (Fig. 5j,k).

Immunoblotting of *vikala* BM-derived macrophage (BMDM) lysates revealed increased levels compared to BMDMs from *Tnip*^+/+^ mice at steady state. LPS stimulation diminished the expression of both mutant and WT TNIP1 consistent with degradation of activated phosphorylated TNIP1 (ref. ^11^). Blocking autophagolysosomal fusion with bafilomycin A1 (BafA) alone did not have a marked effect on basal TNIP1 levels (Extended Data Fig. 5i). However, *vikala* cells treated with both LPS and BafA showed a subtle increase in TNIP1 expression compared to LPS treatment alone (Extended Data Fig. 5i). The higher amount of TNIP1^Q346P^ protein in BMDM lysates, in conjunction with diminished colocalization to LC3B^+^ and ATG7^+^ autophagosomes, suggests that the variant may interfere with TNIP1’s function as a selective autophagy receptor.

### Reduced MyD88 interaction and autophagosome localization

Colocalization of MyD88 with ATG5^+^ autophagosomes exerts an immunomodulatory effect on MyD88 signaling complexes via a nondegradative mechanism^19^. Notably, we did not observe differences in MyD88 levels in *vikala* and WT BMDMs in the presence or absence of BafA, suggesting that this protein is not degraded by autophagy (Extended Data Fig. 5j). Given our demonstration of loss of MyD88 regulation by TNIP1^Q333P^, we investigated whether TNIP1 localized with MyD88 in autophagosomes and whether this could be altered by the variant. TNIP1^WT^ showed strong colocalization with MyD88 in autophagosomes, whereas TNIP1^Q333P^ demonstrated reduced colocalization (Fig. 6b,c). Given its reduced interaction with MyD88, unsurprisingly TNIP1^D472N^^11^ had impaired colocalization with the MyD88 puncta (Fig. 6b,c). We also observed enhanced localization of ectopically expressed IRAK1 with TNIP1^WT^ in HEK 293 cells compared with TNIP1^Q333P^ (Fig. 6d), and increased basal expression of IRAK1 in *Tnip1*^*vik/vik*^ BMDMs compared to *Tnip1*^+/+^ (Extended Data Fig. 5j). BafA treatment further increased IRAK1 levels in both *Tnip1*^*+/+*^ and *Tnip1*^*vik/vik*^ BMDMs, suggesting that the steady-state levels of IRAK1 protein are turned over by autophagy (Extended Data Fig. 5j), as noted previously^11^. Finally, coimmunoprecipitation (co-IP) of overexpressed TNIP1 and MyD88, in HEK 293T cells, revealed diminished interaction of TNIP1^Q333P^ variant with MyD88 compared to TNIP1^WT^ (Fig. 6e). Together, these data indicate that the TNIP1 variant impairs recruitment of MyD88 and Myddosome components to autophagosomes, resulting in sustained TLR7 signaling.

### Effects of Toll-like receptor 9–ligand complexes on *vikala* B cell survival

**Fig. 7:**
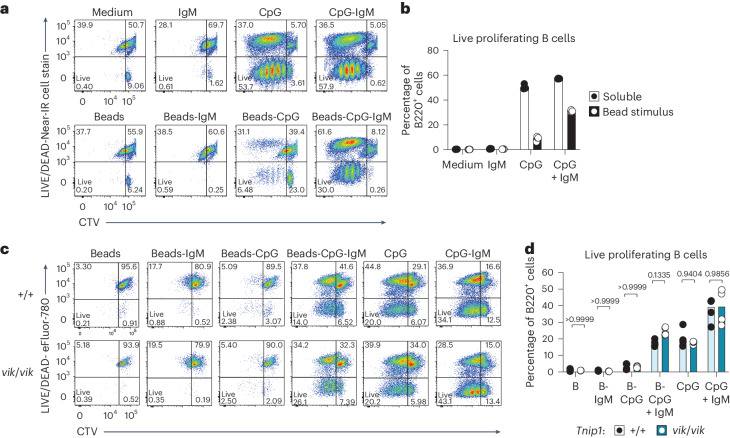
*Vikala* B cells do not have a statistically significant survival advantage to synergistic BCR–TLR9 stimulation. **a**–**b**, Representative flow cytometry plots (**a**) and proportions (**b**) of live purified WT B220^+^ B cells cultured from 8-week-old *Tnip1*^+/+^ (*n* = 1) mice with soluble or bead-conjugated IgM or CpG (ODN 1826) for 72 h; each dot represents a technical replicate. **c**,**d**, Representative flow cytometry plots (**c**) and proportions (**d**) of live B220^+^ B cells from 6–12-week-old *Tnip1*^+/+^ (*n* = 4) and *Tnip1*^*vik*/*vik*^ (*n* = 4) mice cultured with bead-conjugated IgM or CpG (ODN 1826) for 72 h; each dot represents an individual mouse (biological replicate). Statistical significance was calculated using a two-way ANOVA with Šidák’s crrection for multiple comparisons. Exact *P* values are shown. In **a**–**d**, data are representative of *n* = 2 experiments. Source data

We tested the impact of the *TNIP1*^*Q346P*^ variant on Toll-like receptor 9 (TLR9) signaling and B cell survival. Studies^20–22^ have shown a role for immune complexes consisting of TLR9 agonists cross-linked to BCR ligands colocalizing within autophagosome-like structures^23^, leading to postproliferative cell death, thus limiting the emergence of autoreactive B cells. As expected, BCR stimulation (αIgM) induced limited proliferation and induced the death of most B cells, whereas soluble CpG promoted proliferation and survival of divided B cells, as did soluble CpG with IgM (Fig. 7a,b and gating in Supplementary Fig. 3). Stimulation with IgM- and CpG-conjugated beads (mimicking DNA-containing immune complexes) reduced B cell survival by approximately 50%, compared with unconjugated soluble CpG (Fig. 7a,b). When stimulated with beads conjugated to IgM and CpG, *Tnip1*^*vik/vik*^ B cells showed overall similar rates of survival to those of *Tnip1*^*+/+*^ B cells (Fig. 7c,d). These results suggest that the breach in B cell tolerance exerted by TNIP1^Q346P^ is not a consequence of reduced cell death triggered by BCR–TLR9 cross-linking.

### Q333P impairs the localization of TNIP1 to mitophagosomes

**Fig. 8:**
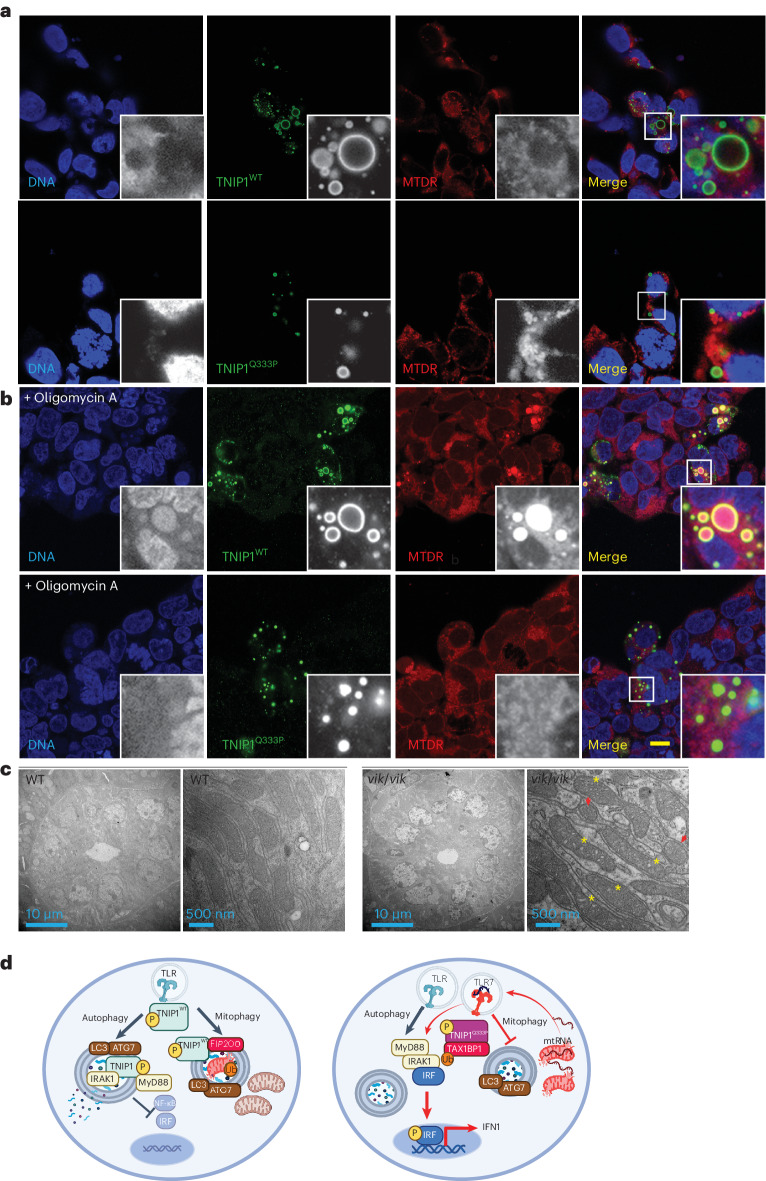
*TNIP1*^Q333P^ mitophagosome recruitment of TNIP1 and mitochondria. **a**,**b**, Immunofluorescence staining of HEK 293 cells overexpressing TNIP1^WT^ or TNIP1^Q333P^ (green) stained with Mitotracker Deep Red without treatment (**a**) or treated with 10 μM oligomycin A for 2 h (**b**) (red). Cells treated with oligomycin A for 2 h are labeled. Scale bar, 10 μm. Data are representative of *n* = 2 experiments. **c**, Representative electron micrographs of submandibular salivary gland ultrathin sections from *n* = 1 WT and *n* = 2 homozygous 16-week-old *vikala* female mice. The red arrowheads indicate swollen mitochondria and the yellow asterisks disorganized cristae. **d**, Schematic of the proposed model of the molecular impact of Q333P on TNIP1 function. On TLR ligation, TNIP1^WT^ is activated and recruited to ATG7-LC3B^+^ autophagosomes where it recruits the Myddosome leading to degradation of some of the components and dampening of the signal. Q333P impairs recruitment to autophagosomes and to MyD88. In addition, mitophagy stimulation activates recruitment of TNIP1^WT^ and selective autophagy receptors, such as TAX1BP1, to sequester damaged and ubiquitinylated mitochondrial components. The interaction probably occurs via the ADH3 domain of TNIP1. Q333P impairs mitophagosome recruitment of TNIP1 and may impact clearance of damaged mitochondria, leading to cytosolic release of mitochondrial damage-associated molecular patterns, mitochondrial RNA or DNA, and activation of the innate TLR7 or TLR9 receptors. The schematic was created with BioRender.com. mtRNA, mitochondrial RNA. Source data

**Extended Data Fig. 6:**
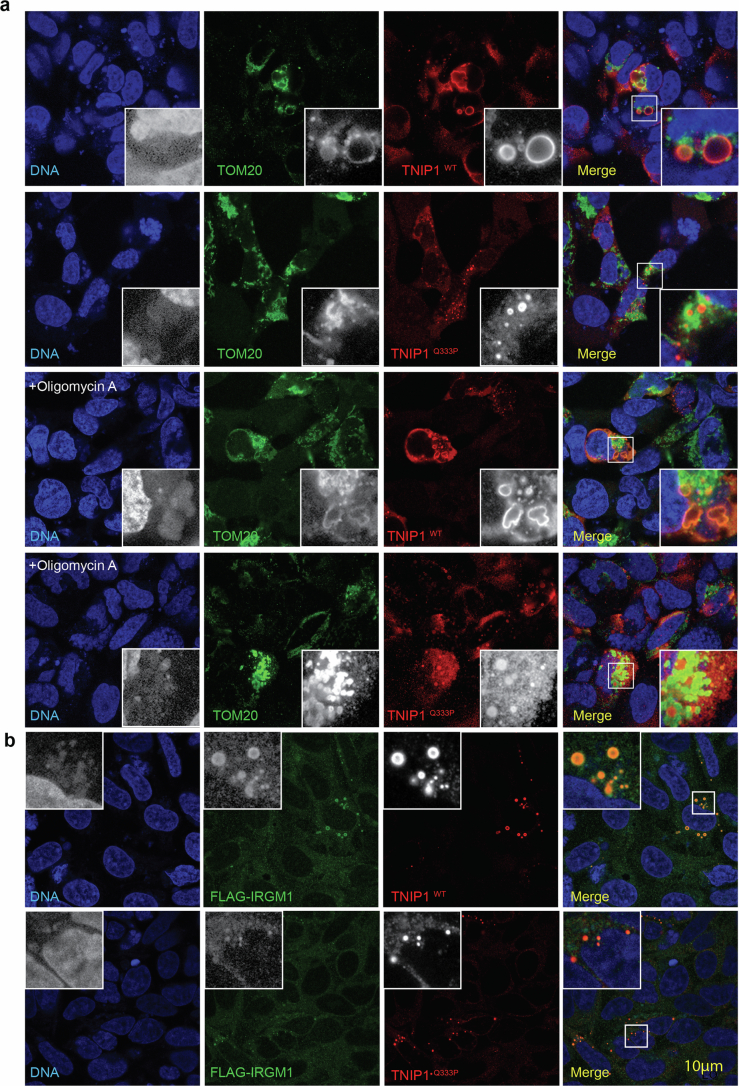
Q333P impairs mitophagosome recruitment of TNIP1 with TOM20+ mitochondria. **a**, Immunofluorescence staining of HEK293 cells overexpressing TAX1BP1, TOM20 (green) and TNIP1 (red). **b**, Immunofluorescence staining of HEK293 cells overexpressing TNIP1 (red) and IRGM1 (green). DNA is stained with DAPI in blue. Panels showing cells treated with 10 μM oligomycin A for 2 h are indicated **(a)**. Scale bars represent 10 μm. Images are representative of *n* = *3* (**a)** and *n* = *2* **(b**) independent experiments with >20 transfected cells per experiment for (**a**) and 10–15 transfected cells per experiment for (**b**). Source data

TNIP1 is a selective mitophagy receptor^15^ with both the LIR and AHD3 domains having roles in regulating the rate of autophagic clearance of damaged mitochondria^12^. To determine whether AHD3-residing Q333P impacts mitophagy, we looked at localization of TNIP1^WT^ and TNIP1^Q333P^ in HEK 293 cells stained with Mitotracker Deep Red (MTDR) to label respiring mitochondria. Under basal conditions, TNIP1 showed little mitochondrial colocalization (Fig. 8a). However, low mitophagy induction using oligomycin A, which blocks the mitochondrial F1/F0 ATP synthase^24^, resulted in costaining of large TNIP1 puncta with MTDR. Strikingly, this was enhanced for TNIP1^WT^ compared with TNIP1^Q333P^ (Fig. 8b). Unlike TAX1BP1, when expressed alone TNIP1 does not localize to mitochondria even under mitophagic conditions^12^. Therefore, we coexpressed the ATP translocase of the outer mitochondrial membrane (TOM20) together with TAX1BP1 and TNIP1 in HEK 293 cells and examined TNIP1 localization with TOM20 using immunofluorescence staining. Coexpression of TAX1BP1 led to TOM20 recruitment to enlarged TNIP1^+^ puncta as observed previously^12^, even in the absence of oligomycin A treatment. The smaller TNIP1^Q333P^ puncta were impaired in this colocalization (Extended Data Fig. 6a).

The GTPase IRGM1, which has been linked to SjD-like autoimmunity, has a critical role in efficient clearance of damaged mitochondria by supporting autophagosome–lysosome fusion and preventing TLR7-dependent IFN1 induction from mitochondrial 16s RNA release^25^. Given the SjD-like infiltration of lymphocytes in the salivary glands of *vikala* mice, we looked at whether IRGM1 colocalizes with TNIP1^+^ puncta. In HEK 293 cells, IRGM1 overexpression markedly reduced TNIP1 puncta size; however, both WT and Q333P TNIP1 puncta colocalized with IRGM1, albeit at somewhat reduced levels for Q333P (Extended Data Fig. 6b).

**Extended Data Fig. 7:**
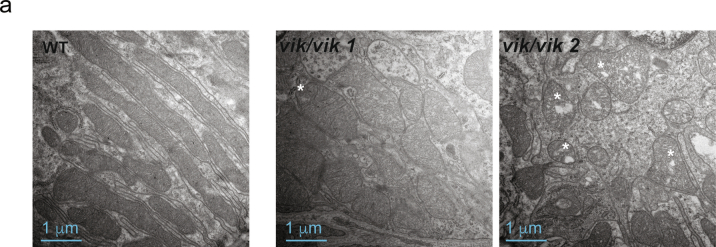
Salivary gland epithelial cells from homozygous *vikala* mice develop damaged mitochondria. **a**, Representative electron micrographs of submandibular salivary gland ultrathin sections from *Tnip1*^*+/+*^ *(n* = *1)* and *Tnip1*^*vik/vik*^ *(n* = *2)* 16-week-old *vikala* female mice. White asterisks indicate clearings indicative of mitochondrial membrane damage. Scale bars represent 1 μm. Source data

Finally, we used electron microscopy to visualize mitochondrial morphology in the salivary gland epithelial cells of *vikala* mice, given abnormalities in these cells in patients with SjD^26^. The salivary gland mitochondria of *Tnip1*^*vik/vik*^ mice showed morphological alterations with swelling (Fig. 8c, red arrowheads; Extended Data Fig. 7a) and disorganized cristae (Fig. 8c, yellow asterisks; Extended Data Fig. 7a). Together, our data show that TNIP1^Q333P^ is impaired in its localization to mitophagosomes, probably impacting clearance of damaged mitochondria (Fig. 8d).

## Discussion

Our study established a causative role for *TNIP1* variants in human autoimmunity. Edited *vikala* mice harboring an ortholog to a rare human *TNIP1*^Q333P^ variant found in two unrelated probands, developed B cell-mediated autoimmunity featuring elevated IgG2c and immune cell infiltrates in salivary glands that are consistent with features of the probands’ clinical phenotype. Although *vikala* mice share phenotypic features with previously reported TNIP1 loss-of-function (LOF) mice, our study revealed that TNIP1^Q333P^ does not cause autoimmunity by enhancing NF-κB activation, but rather enhances IFN1 signaling and impairs Myddosome^11^ and mitochondrial recruitment to autophagosomes.

It is intriguing that the *TNIP1*^Q333P^ variant retains repressive capacity over NF-κB signaling. LOF mutations in the polyubiquitin-binding domain, such as the *TNIP1*^*D472N*^ variant prevent TNIP1-mediated repression of NF-κB activity; overactive NF-κB signaling was reported to drive lupus-like disease in *Tnip1*^*D485N*^ mice^7^. Our findings are consistent with a study in which a somatic *TNIP1*^Q333P^ mutation was assessed alongside other human *TNIP1* variants causing ocular adnexal mucosa-associated lymphoid tissue lymphoma and it repressed NF-κB normally^27^. Thus, discrepancies between *vikala* and polyubiquitin-binding defective *Tnip1*^*D485N*^ mice, such as the presence of glomerular injury in *Tnip1*^*D485N*^ mice but not in *vikala* mice, may be explained by a lack of NF-κB-inducible proinflammatory mediators in the latter^28^. This may indicate that nephritis requires activation of both IFN1 and NF-κB pathways, and thus occurs with lesions upstream of TNIP1, as seen in our demonstration that TLR7 gain of function causes lupus nephritis^2^.

The limited effect of TNIP1^Q333P^ on NF-κB regulation may be explained by the protein domain harboring the mutation. TNIP1^Q333P^ occurs in the AHD3 domain, located upstream of the ubiquitin-binding region that is required for NF-κB repression^29^. AHD3 is essential for the interaction with TAX1BP1 and for regulation of mitophagy^12^. TAX1BP1–TNIP1 complexes inhibit IFNβ antiviral signaling by disrupting TBK1 polyubiquitination^14^. MyD88 can induce IFN1 production via its ability to bind TBK1 (refs. ^30,31^). Activated TBK1 can phosphorylate IFN-regulatory factor-inducing IFN1 production^8^. This aligns with our finding that TNIP1^Q333P^ profoundly impairs regulation of TBK1-induced IFNβ activity.

A key finding providing additional mechanistic insights is that cells expressing TNIP1^Q333P^, MyD88 and IRAK1 have impaired localization to autophagosomes and there is weakened interaction between MyD88 and TNIP1. Recent findings that TNIP1 is a selective autophagy receptor^11^, and that MyD88 signaling is repressed by the autophagosome protein ATG5 (ref. ^19^), suggests that TNIP1 acts in autophagosomes to inactivate MyD88 signaling and this is disrupted by the Q333P variant. Furthermore, TBK1 promotes autophagic degradation of activated TNIP1 via phosphorylation of TNIP1’s LIR motif^32^. Such autophagic turnover of TNIP1 is impaired by the Q346P mutation (orthologous to the human Q333P variant), as seen by the increase in TNIP1 protein levels in *vikala* BMDMs and B cells. By contrast, and as expected, MyD88 levels did not change. Importantly, these findings point to a defect in TBK1-dependent recruitment of signaling-competent MyD88 to autophagosomes, rather than a generalized defect in autophagy, which is in fact reported to be augmented in lupus-prone mice and human SLE B cells^33^.

TNIP1–TAX1BP1 complexes also prevent TAX1BP1 binding to ubiquitinated cargo on damaged mitochondria^12^, thereby inhibiting mitophagy. Thus, Q333P may impair interactions with autophagosome–mitophagosome regulatory proteins, thereby impairing the localization of TNIP1 to mitophagosomes, as we have demonstrated. We speculate that impaired mitophagy in mice expressing TNIP1^Q346P^ leads to mitochondrial RNA spillage, which has been shown to increase TLR7 signaling^25^.

*Vikala* mice developed salivary gland infiltration and mitochondrial morphological defects within salivary epithelial cells, which is consistent with the sicca symptoms in proband A.III.2. Given the expression of TNIP1 in the salivary gland^34^, it is tempting to speculate that abnormal salivary gland mitochondria occur because of impaired mitophagosome localization of TNIP1^Q333P^. However, we cannot discount that mitochondrial damage may be secondary to inflammation in this organ. Immune cell infiltrates, including CXCR3^+^ effector T cells, are seen within the glandular tissue of patients with SjD^35^. We also observed salivary gland inflammation in *vikala* mice, which has not been reported in other TNIP1 LOF models.

ABCs and PCs were expanded in *vikala* mice. Patients with SLE harbor circulating ABCs and accumulate extrafollicular T_H_ cells, features aligned with increased TLR7 signaling. Indeed, increased TLR7 signaling within B cells drives ABC and PC accumulation^2^. ABCs undergo immunoglobulin class switching and produce IgG2c after TLR stimulation^36^. It is probable that ABCs produce the pathogenic IgG2c antibodies in *vikala* mice, given the role of Tbet in inducing class switching to IgG2c in mice in a B cell-intrinsic manner^37,38^. Consistent with this, ablation of *Tlr7* or *Myd88* reduced ABCs and IgG2c levels in *vikala* mice.

The contribution of the rare *TNIP1*^Q333P^ variant to autoimmune pathogenesis is demonstrated by the shared phenotypes observed in two unrelated families and the *vikala* mice providing a bona fide model of human disease. The one notable difference is the predominance of elevated IgG4 in the patients and IgG2c in mice. Interleukin-4 drives class switching to IgG4 in humans and IgG1 in mice, whereas IFNγ drives IgG2c in mice. Nevertheless, human and mouse IgG subclasses lack perfect functional correlates across species^39^ and IFNγ is also known to have a role in human SLE-associated ABC formation^40^. IgG4 and mouse IgG2c, elevated in *vikala* mice, may share features important for their association with autoimmune conditions: elevated IgG4 is observed in patients with IgG4-related disease and in patients with SjD^41^. Both diseases present with salivary gland inflammation and are associated with expansion of T_FH_ cells that also characterize *vikala* mice^42,43^. The Australian proband has some clinical features of SjD and IgG4-related disease, although her manifestations have not evolved toward either pathology despite observation for more than 15 years. Lack of salivary gland biopsies also precluded reaching a diagnosis of IgG4-related disease and both probands exhibited other features more typically associated with systemic autoimmunity. Thus, we do not propose *TNIP1*^*Q333P*^ as a Mendelian cause of IgG4-related disease.

Our findings nevertheless suggest that TNIP1 and localization of MyD88, IRAK1 and mitochondria to autophagosomes should be investigated further in other autoimmune and inflammatory disorders, particularly when elevated IgG4 is observed. Finally, in patients with ambiguous diagnoses of human autoimmunity featuring elevated IgG4 and sicca symptoms, TNIP1-mediated disease may be present and would pave the way for pathway-targeted treatments such as TLR7 and TBK1 inhibitors.

## Methods

### Informed consent and ethics approval

Written informed consent was obtained from the study participants as part of the Australian and China Center for Personalized Immunology Programs. The study was approved by and complies with all relevant ethical regulations of the Australian National University (ANU) and ACT Health Human Ethics Committees (ACT Health ETH.1.15.015, ANU 2015/079, ETH.1.16.011 2016/071, ETH.10.3.435 2010/409) or by the Renji Hospital Ethics Committee of Shanghai Jiaotong University School of Medicine.

### Whole-exome sequencing

Participant DNA samples were enriched using the Human SureSelect XT2 All Exon V4 Kit and sequenced using the Illumina HiSeq 2000 system. Bioinformatics analysis was performed at the John Curtin School of Medical Research (JCSMR), ANU. Briefly, raw sequence reads were aligned to the human reference genome (Hg19) and single-nucleotide variants and small insertions and deletions called using the Genome Analysis Toolkit. Results were scored based on rarity as reported according to MAF, deleteriousness based on PolyPhen-2, SIFT and CADD score, expression in immune tissues and reported mouse phenotypes, as described previously^18^. The variant was confirmed using Sanger sequencing: *TNIP1* forward 5′-TTTCGAGAGCTGAGGGATGG-3′**;** *TNIP1* reverse, 5′-ACTCCCCAAGGTTCAAAGCTG-3′.

### Human PBMC preparation

Human PBMCs were isolated using Ficoll-Paque (GE Healthcare Life Sciences) gradient centrifugation and frozen in FCS (Gibco) with 10% dimethyl sulfoxide (Sigma-Aldrich).

### Mice

C57BL/6 mice were housed and bred under specific pathogen-free conditions. All mouse procedures were conducted according to regulations approved by the ANU Animal Experimentation Ethics Committee (A2022/18, A2018/38, A2021/29) under the National Health and Medical Research Council Australian code of practice. Mice used in the cellular phenotyping experiments were aged 16–20 weeks or 20–30 weeks. Both male and female mice were used. Experimental mice were randomly distributed across cages.

### CRISPR–Cas9-mediated genome editing of mouse zygotes

C57BL/6Ncrl mice were housed under specific pathogen-free conditions. All mouse procedures were approved by the ANU Animal Experimentation Ethics Committee (AEEC A2014/058 and A2014/016) under the National Health and Medical Research Council Australian code of practice. TNIP1 guide RNA 5′-GAAGCAGCAGTATGAGCCAGA-3′, single-stranded oligonucleotide 5′- TTCTAACCCCAGTACCTGTCTGCCCACAGCTGCTGGAAGTGAACAAGCAGTGGGACCAGCATTTCCGGTCCATGAAGCAGCAGTATGAGCCGAAGGTGATGGAGTTCCCGGGAGCTGAGCCGAGGACGGCTCGGGGAGGCGGGCTGAGAGGCTTGTGACCTGGCTGAGATGGGGACGGTGG-3′ and Cas9 protein were obtained from Integrated DNA Technologies. The procedure for CRISPR editing of mouse zygotes, pronuclear injection and mouse genotyping has been described previously^2^. The primers used for genotyping and strain validation were: *Tnip1*^*Q346P*^ forward, 5′-TCACAGTAACTCTCCAGGCC-3′; *Tnip1*^*Q346P*^reverse, 5′-TCCACACTTGCCTCTTCCAT-3′.

### Histology

Histological analysis and scoring of microscopy imaging was carried out blinded and with deidentified genotypes. Murine salivary glands and kidneys were fixed in 10% neutral buffer formalin solution, embedded in paraffin and stained with H&E. Focal lymphocytic infiltrates in salivary gland tissue sections, defined as foci comprising 50 or more mononuclear cells, were enumerated and scored based on the following scale: 0, no infiltrates; 1, one focal infiltrate; 2, multiple focal infiltrates.

### Antibodies

Antibodies for immunoblotting and the co-IP studies were as follows: mouse anti-HA (clone HA-7, cat. no. H3663, Sigma-Aldrich); rabbit anti-HA (cat. no. H6908, Sigma-Aldrich); mouse anti-FLAG M2 (cat. no. F1804, Sigma-Aldrich); mouse anti-Myc (Ab-1, clone 9E10, cat. no. OP10-200UG, Sigma-Aldrich); rabbit anti-TNIP1 (cat. no. HPA037893, Sigma-Aldrich); mouse anti-actin (clone JLA20, Developmental Studies Hybridoma Bank, University of Iowa); mouse anti-alpha-tubulin (B-5-1-2, cat. no. 32-2500, Thermo Fisher Scientific); rabbit anti-IκBα (cat. no. 9242, Cell Signaling Technology); rabbit anti-phospho IκBα (Ser32) (clone 14D4, cat. no. 2859, Cell Signaling Technology); mouse anti-SQSTM1 (cat. no. ab56416, Abcam); rabbit anti-IRAK1 (D51G7, cat. no. 4504, Cell Signaling Technology); rabbit anti-MyD88 (cat. no. 4283, Cell Signaling Technology); mouse anti-EEA1 (clone N19, cat. no. E7659, Sigma-Aldrich); rabbit anti-LAMP1 (cat. no. ab24170, Abcam); and rabbit anti-RAB7 (C-19, cat. no. sc-6563, Santa Cruz Biotechnology). Secondary antibodies were conjugated to horseradish peroxidase (HRP) (mouse anti-rabbit IgG peroxidase conjugated, light chain specific, cat. no. 211-032-171, Jackson ImmunoResearch) used at 1:2,500 dilution, goat anti-mouse IgG peroxidase conjugated, light chain specific (cat. no. 115-035-174, Jackson ImmunoResearch) used at 1:2,500 dilution, goat anti-mouse IgG, HRP-conjugated (cat. no. 62-6520, Thermo Fisher Scientific) used at 1:5,000 dilution, goat anti-rabbit IgG, HRP-conjugated (cat. no. 65-6120, Thermo Fisher Scientific) and Alexa Fluor 568 or Alexa Fluor 488 (Invitrogen Molecular Probes). For IP, 2 μg of primary antibodies were used. Antibodies used for immunofluorescence imaging included: mouse anti-HA used at 1:300 dilution; rabbit anti-HA used at 1:300 dilution; mouse anti-FLAG M2 used at 1:200 dilution; mouse anti-myc (Ab-1) used at 1:150 dilution; rabbit anti-TNIP1 used at 1:100 dilution; mouse anti-SQSTM1 used at 1:100 dilution; rabbit anti-IRAK1 used at 1:100 dilution; rabbit anti-MyD88 used at 1:100 dilution; mouse anti-EEA1 used at 1:100 dilution; rabbit anti-LAMP1 used at 1:100 dilution; and goat anti-RAB7 used at 1:100 dilution. Secondary antibodies conjugated to Alexa Fluor 568, 594 or 488 were all used at 1:500 dilution (donkey anti-goat IgG Alexa Fluor 488, cat. no. A-11055, Invitrogen; donkey anti-rabbit IgG, Alexa Fluor 488, cat. no. A-21206, Invitrogen; donkey anti-mouse IgG Alexa Fluor 488, cat. no. A21202, Invitrogen; Alexa Fluor 568 donkey anti-mouse IgG, cat. no. A10037, Invitrogen; donkey anti-rabbit IgG, Alexa Fluor 594, cat. no. A-21207). The antibodies and dyes used for staining mouse tissues for flow cytometry include: annexin V-FITC (1:100 dilution, cat. no. 560931, BD Pharmingen); B220-Alexa Fluor 647 (1:400 dilution, clone RA3-6B2, cat. no. 557683, BD Pharmingen); B220-BUV395 (1:200 dilution, clone RA3-6B2, cat. no. 563793, BD Horizon); B220-BUV737 (1:200 dilution, clone RA3-6B2, cat. no. 612838, BD Horizon); BCL6-A467 (1:40 dilution, clone K112-91, cat. no. 561525, BD Pharmingen); BST2-PE (1:400 dilution, clone 927, cat. no. 127010, BioLegend); CCR7-PerCP-Cy5.5 (1:50 dilution, clone 4B12, cat. no. 120116, BioLegend); CD3 Alexa Fluor 700 (1:200 dilution, clone 17A2, cat. no. 100216, BioLegend); CD4 Alexa Fluor 647 (1:400 dilution, clone RM4-5, cat. no. 100530, BioLegend); CD4 BUV395 (1:200 dilution, clone 6K1.5, cat. no. 563552, BD Horizon); CD4 PerCP-Cy5.5 (1:400 dilution, clone RM4-5, cat. no. 116012, BioLegend); CD8-BUV805 (1:200 dilution, clone 53-6.7, cat. no. 612898, BD Horizon); CD11b-PerCP-Cy5.5 (1:400 dilution, clone M1/70, cat. no. 101228, BioLegend); CD11c-Alexa Fluor 647 (1:200 dilution, clone N418, cat. no. 117312, BioLegend); CD11c-BV510 (1:400 dilution, clone N418, cat. no. 117353, BioLegend); CD11c-FITC (1:800 dilution, clone N418, cat. no. 117305, BioLegend); CD19 Alexa Fluor 700 (1:200 dilution, clone eBio1D3, cat. no. 56-0193-82, Invitrogen); CD19-BV605 (1:400 dilution, clone 6D5, cat. no. 115540, BioLegend); CD19-BUV395 (1:200 dilution, clone 1D3, cat. no. 563557, BD Horizon); CD21/35-BV605 (clone 7G6, 1:400 dilution, BD Horizon); CD23-BV421 (1:400 dilution, clone B3B4, BioLegend); CD25-APC (1:200 dilution, clone PC61, cat. no. 102012, BioLegend); CD25-PE (1:100 dilution, clone PC62, BioLegend); CD44-FITC (1:50 dilution, clone IM7, cat. no. 563176, BD Pharmingen); CD44-Pacific Blue (1:400 dilution, clone IM7, cat. no. 103020, BioLegend); CD45.2-PerCP-Cy5.5 (1:200 dilution, clone 104, cat. no. 552950, BD Biosciences); CD45.1-BV605 (1:100 dilution, clone A20, cat. no. 110737, BioLegend); CD45.1-BV711 (1:200 dilution, clone A20, cat. no. 110739, BioLegend); CD95 (FAS)-BV510 (1:200 dilution, clone Jo2, cat. no. 563646, BD Horizon); CD98-PE-Cy7 (1:200 dilution, clone RI.388, cat. no. 128214, BioLegend); CD138-PE (1:400 dilution, clone 281-2, cat. no. 561070, BD Pharmingen); CXCR3-PE (1:400 dilution, clone CXCR3-173, cat. no. 126506, BioLegend); CXCR5-Biotin (1:40 dilution, clone 2G8, cat. no. 551960, BD Biosciences); FOXP3-FITC (1:200 dilution, clone FJK-16s, cat. no. 11-5773-82, eBioscience); FOXP3-PE-Cy7 (1:400 dilution, clone FJK-16s, cat. no. 25-5773-82, eBioscience); IA/IE-BV421 (1:800 dilution, clone M5/114.15.2, cat. no. 107631, BioLegend); IgD-PerCP-Cy5.5 (1:400 dilution, clone 11-26c.2a, cat. no. 564273, BD Pharmingen); IgD-PE (1:800 dilution, clone 11-26c.2a, cat. no. 405705, BioLegend); IgM-FITC (1:200 dilution, clone II/41, cat. no. 553437, BD Pharmingen); IgM-PE-Cy7 (1:400 dilution, clone II/41, cat. no. 25-5790-82, Invitrogen); PD1-BV421 (1:200 dilution, clone 29F.1A12, cat. no. 135217, BioLegend); Ly6C-Biotin (1:200 dilution, clone AL-21, cat. no. 557359, BD Pharmingen); Ly6G-FITC (1:200 dilution, cat. no. 127606, BioLegend); SiglecH-APC (1:200 dilution, clone 551, cat. no. 129611, BioLegend); streptavidin-BV510 (1:400 dilution, cat. no. 405233, BioLegend); streptavidin-PE-Cy7 (1:400 dilution, cat. no. 25-4317-82, eBioscience); LIVE/DEAD APC-Cy7 (eFluor 780) (1:1,000 dilution, cat. no. 65-0865-18, eBioscience); LIVE/DEAD Fixable Aqua Dead Cell Stain (1:1,000 dilution, cat. no. L34957, Invitrogen); Fc Block CD16/CD32 (1:100 dilution, clone 2.462, cat. no. 553141, BD Pharmingen); and Cell Trace Violet (manufacturer’s recommendations, cat. no. C34557, Molecular Probes). Antibodies used to stain human PBMCs include: CD11c-BUV395 (1:50 dilution, clone B-ly6, cat. no. 563787, BD Biosciences); CD11c-BV510 (1:25 dilution, clone B-ly6, cat. no. 563026, BD Biosciences); CD127-BB700 (1:25 dilution, clone HIL-7R-M21, cat. no. 566398, BD Biosciences); CD19 APC-Cy7 (1:50 dilution, clone SJ25C1, cat. no. 348794, BD Biosciences); CD19-BV650 (1:50 dilution, clone HIB19, cat. no. 302238, BioLegend); CD24-BV605 (1:25 dilution, clone ML5, cat. no. 311124, BioLegend); CD24-BV711 (1:50 dilution, clone ML5, cat. no. 563401, BD Biosciences); CD25-APC-R700 (1:50 dilution, clone 2A3, cat. no. 565106, BD Biosciences); CD27-PE-Cy7(1:20 dilution, clone M-T271, cat. no. 560609, BD Biosciences); CD27-APC-eFluor780 (1:50 dilution, clone O323, cat. no. 47-0279, eBiosciences); CD38-APC (1:20 dilution, clone HB-7, cat. no. 345807, BD Biosciences); CD38-BV605 (1:25 dilution, clone HIT2, cat. no. 303532, BioLegend); CD3-BV786 (1:200 dilution, clone SK7, cat. no. 563799, BD Biosciences); CD3-FITC (1:50 dilution, clone UCHT1, cat. no. 300406, BioLegend); CD45RA-PE-Cy7 (1:50 dilution, clone HI100, cat. no. 25-0458-73, eBioscience); CD45RA-Pacific Blue (1:100 dilution, clone HI100, cat. no. 304123, BioLegend); CD4-BUV496 (1:100 dilution, clone SK3, cat. no. 564651, BD Biosciences); CD56-BUV737 (1:400 dilution, clone NCAM16.2, cat. no. 564447, BD Pharmingen); CD8-BV421 (1:100 dilution, clone RPA-T8, cat. no. 562428, BD Biosciences); CXCR3-PE (1:50 dilution, clone G025H7, cat. no. 353706, BioLegend); CXCR5-Alexa Fluor 647 (1:50 dilution, clone RF8B2, cat. no. 558113, BD Biosciences); IgD-BV421 (1:50 dilution, clone IA6-2, cat. no. 562518, BD Biosciences); IgD-BV510 (1:30 dilution, clone IA6-2, cat. no. 348220, BioLegend); LIVE/DEAD Fixable Blue Dead Cell Stain (1:1,000 dilution, cat. no. L23105, Invitrogen); LIVE/DEAD Stain Kit Green Fluorescent (1:1,000 dilution, cat. no. L23101, Invitrogen); and PD1-PE-CF594 (1:50 dilution, clone EH12.2H7, cat. no. 329940, BioLegend). All fluorescence-activated cell sorting and microscopy work was carried out at the Microscopy and Cytometry Facility, ANU.

### Flow cytometry

Murine spleens were isolated as single-cell suspensions after red blood cell lysis. To stain for surface markers, cells were incubated for 30 min at 4 °C in antibody mixture diluted in ice-cold staining buffer (2 mM EDTA, 2% FCS in PBS). Before addition of the antibody staining cocktails, purified rat anti-mouse CD16/CD32 (Mouse BD Fc Block, BD Biosciences) was used to block Fc receptors; Zombie aqua dye (BioLegend) or anti-Fixable Viability Dye-eFluor780 (eBioscience) was used to stain dead cells. To stain intracellular markers, the Foxp3 Transcription Factor Staining Buffer Set (eBioscience) was used according to the manufacturer’s instructions. A Fortessa or LSRFortessa X-20 cytometer with FACSDiva software (BD Biosciences) were used for flow cytometry acquisition; data were analyzed using FlowJo (FlowJo LLC)

### Generation of bone marrow chimeras

Rag1^−/−^ recipient mice were irradiated (500 cGy) and intravenously injected with equal numbers of BM cells (2 × 10^6^) from either WT or *vikala* CD45.2 and WT CD45.1 donor mice. CD45.1 donors were C57BL/6-Ptprc^a^ mice. Bactrim was administered to the drinking water provided to mice 48 h prior to injection and for 6 weeks following injection. 16 weeks post-reconstitution mice were euthanised and spleens harvested for flow cytometric phenotyping.

### Generation of particulate immune complexes and B cell stimulations

Particulate immune complexes of anti-BCR (Biotin-SP AffiniPure Fab Fragment Goat Anti-Mouse IgM, μ-chain-specific, cat. no. 115-067-02015-067-020, Jackson ImmunoResearch), CpG-B (ODN 1826 Biotin, cat. no. tlrl-1826b, Invivogen) and anti-BCR-CpG for enhanced in vitro stimulation of splenocytes were generated by conjugation of ligands to streptavidin-coated 0.196-μm microspheres (cat. no. CP01001, Bangs Laboratories) as described previously^44^. Splenocytes were negatively magnetic-activated cell-sorted for B cells and stained with Cell Trace Violet (cat. no. C34557, Molecular Probes) at a concentration of 10 μM in PBS warmed to 37 °C for 10 min. Samples were diluted with 10 ml cold complete Roswell Park Memorial Institute (RPMI) 1640 medium and rested on ice for 5 min before centrifugation and resuspension of the pellet. A total of 2 × 10^5^ of these B cells were incubated with 5,000× conjugated microspheres for each of the stimulation conditions for 72 h in complete RPMI 1640 medium before staining with fluorochrome-conjugated antibodies for analysis using flow cytometry.

### Cytokine analysis

Analysis of the cytokines secreted by pDCs were determined using a multiplex ELISA for interleukin-1β, TNFα, KC and interleukin-6 (MCYTOMAG-70K, Merck Millipore), and an IFNβ ELISA (MECY2MAG-73K, Merck Millipore) according to the manufacturer’s instructions. Cytokine quantification was performed on a MAGPIX (Luminex) analyzer and data were collected using xPONENT v.4.2.

### Mesoscale

Mesoscale was performed according to the manufacturer’s guidelines using the Mouse Isotyping Panel 1 Kit (cat. no. K15183B, Meso Scale Discovery) for IgA, IgG1, IgG2a, IgG2b, IgG3 and IgM. Serum was diluted 1:100,000 in 1% FCS in PBS; 0.05% PBS-Tween 20 was used for all wash steps.

### BMDM and B cell cultures

Primary BMDMs were cultured for 5 days in DMEM (Gibco) supplemented with 1% nonessential amino acids (Gibco), 10% FCS, 30% medium conditioned by L929 mouse fibroblasts and 1% penicillin-streptomycin. BMDMs were seeded onto 12-well plates at a density of 1 × 10^6^ cells per well, followed by overnight incubation. Cells were treated with LPS (100 ng μl^−1^) alone or in combination with bafilomycin A1 (100 nM) (Selleckchem) for 2 h after treatment with TLR agonists; cells were collected and either lysed in 100 μL radioimmunoprecipitation assay buffer for immunoblotting or 1 ml TRIzol for quantitative PCR.

Splenocytes from mice were MACS-sorted using a Pan B Cell Isolation Kit II (cat. no. 130-104-443, Miltenyi Biotec); 1 × 10^6^ cells per well were plated before stimulation for the indicated time points with CpG-B (100 nM, cat. no. tlr1-1826-1, InvivoGen), R848 (2 μg ml^−1^, cat. no. tlrl-r848, InvivoGen), AffiniPure F(ab')_2_ Fragment Goat Anti-mouse IgM, μ-chain-specific (10 μg ml^−1^, cat. no. 115-006-075, Jackson ImmunoResearch), recombinant mouse BAFF (25 ng ml^−1^, cat. no. 591202, BioLegend) and CD40 monoclonal antibody (1C10) (20 μg ml^−1^, cat. no. 16-0401-82, Thermo Fisher Scientific).

### BM-derived pDC culture

BM cells isolated from mice femur and tibia were cultured in T25 flasks for 10 days (37 °C in 5% CO_2_) in RPMI 1640 (cat. no. 11875093, Thermo Fisher Scientific) supplemented with 10% FCS (cat. no. F8192, Sigma-Aldrich), 2 mM L-glutamine (cat. no. 25030081, Thermo Fisher Scientific), 100 U ml^−1^ penicillin-streptomycin (cat. no. 15140163, Thermo Fisher Scientific), 2.5 mM, pH 7.2–7.5, pKa 7.3 at 37 °C (cat. no. 15630130, Thermo Fisher Scientific), 1 mM sodium pyruvate (cat. no. 11360070, Thermo Fisher Scientific) and 300 ng ml^−1^ of mouse Flt3 ligand (cat. no. 130-097-372, Miltenyi Biotec) to promote pDC differentiation. pDCs were seeded onto 96-well plates (150,000 cells per well) and rested for 2 h before stimulation with CpG-A (1 µM, cat. no. tlr-1585, InvivoGen), R837 (1 µg ml^−1^, cat. no. tlr-imq, InvivoGen) or R848 (1 µg ml^−1^, cat. no. tlrl-r848, InvivoGen) or infection with *E. coli* at a multiplicity of infection of 50 for 6–12 h. After stimulation, the 96-well plates were centrifuged at 1,000*g* for 5 min to pellet the pDCs. The supernatant was collected for cytokine analysis. For the in vitro experiments, 2–4 cell replicates were randomly allocated to the wells of tissue culture plates and treated with the indicated experimental conditions or stimuli.

### HEp-2 immunofluorescence

Antinuclear antibodies were determined using HEp-2 slides (NOVA Lite). Serum was diluted 1:40 before addition to the HEp-2 slides and stained using donkey anti-mouse IgG Alexa Fluor 488 secondary antibody at 1:5,000 dilution. Imaging of the slides was performed using an Olympus IX71 inverted fluorescence microscope with an Olympus UPlanSApo 20× objective.

### ELISAs for anti-DNA antibody detection

Ninety-six-well plates (SANTSC-204463) were coated with poly-l-lysine (cat. no. P8920, Sigma-Aldrich) and incubated at 22–24 °C for 5 h before the addition of 50 ng of calf thymus DNA (cat. no. D7290, Sigma-Aldrich) diluted in ELISA coating buffer (0.05 M sodium carbonate anhydrous/sodium hydrogen carbonate, pH 9.6) overnight. Plates were blocked in PBS and 1% BSA blocking buffer for 2 h at 22–24 °C. Mouse serum was diluted 1:40 with blocking buffer and incubated overnight at 4 °C. The plates were washed (PBS and 0.05% Tween 20), and goat anti-mouse IgG-AP antibodies (cat. no. 1030-04, Southern Biotech) were added for 1 h at 37 °C. Phosphatase substrate (cat. no. S0942, Sigma-Aldrich) diluted (1 mg ml^−1^) in ELISA developing buffer (0.1 M glycine, 0.1 mM ZnCl_2_, 1.0 M MgCl_2_·6H_2_O, pH 10.4) was added to the plate. The absorbance of the samples was measured at 405 nm and normalized to background absorbance at 605 nm using the Infinite 200 PRO Tecan Microplate Reader (Tecan).

### ELISAs for murine IgG and IgG2c quantification

Ninety-six-well plates were coated with goat anti-mouse kappa (Southern Biotech cat. no. 1050-01) in ELISA coating buffer, and incubated at 22–24 °C overnight at 4 °C. Plates were blocked in PBS and 3% BSA blocking buffer for 1 h at 37 °C. The plates were washed (PBS and 0.05% Tween 20). Mouse serum was diluted 1:500 (IgG assay) or fourfold from 1:100 to 1:409,6001 (IgG2c assay) in 3% BSA blocking buffer, added to the plate and incubated for 1 h at 37 °C. The plates were washed and goat anti-mouse IgG-AP or goat anti-mouse IgG2c-AP (cat. no. 1079-04, Southern Biotech) was added; the plates were incubated for 1 h at 37 °C. The plates were washed and phosphatase substrate diluted (1 mg ml^−1^) in ELISA developing buffer was added and incubated for 1 h or up to 3 h at 37 °C. The absorbance of samples was measured at 405 nm and normalized to background absorbance at 630 nm using the Infinite 200 PRO Tecan Microplate Reader. The total serum IgG concentration was determined using a standard curve generated using optical density readouts from a serially diluted IgG1 isotype control (cat. no. 02-6100, Thermo Fisher Scientific).

### Microbial culture

*E. coli* (strain no. 11775, American Type Culture Collection) containing the relevant plasmid constructs was grown in Luria-Bertani medium (cat. no. 244620, BD) overnight under aerobic conditions at 37 °C. Overnight cultures were used to extract plasmid DNA using mini or midi plasmid extraction kits (QIAGEN).

### Expression vectors and mutagenesis

The following expression vectors were obtained: untagged TBK1 (cat. no. SC11125, Origene Technologies); HA-TNIP1 (cat. no. HG14942-NY, Sino Biological); untagged IRF5 (cat. no. SC104269, OriGene Technologies), untagged MyD88 (cat. no. OHu21475, GenScript); pNIFTY (NF-κB luciferase vector, InvivoGen); IFNβ luciferase (a gift from J. P.-Y. Ting, Department of Microbiology-Immunology, Lineberger Comprehensive Cancer Center, University of North Carolina); pRL-CMV (Promega Corporation); ATG7 (pCMV-myc-Atg7, plasmid no. 24921, Addgene); IRAK1 (cat. no. OHu18911C, GenScript);, TAX1BP1 (a gift from M. Cook, Department of Medicine, University of Cambridge); FLAG-TRAF6 (plasmid no. 66929, Addgene); and A20 (cat. no. HG12089-NF, Sino Biological). Mutagenesis was performed using the Quikchange I and II site-directed mutagenesis protocols (Agilent Technologies). The mutagenesis primer sequences used to generate the *TNIP1* variants were: 5′-GAAGCAGCAGTATGAGCCGAAGATCACTGAGCTGC-3′ (Q333P); and 5′-GAAGATCTTCGAGGAGAACTTCCAGAGGGAGCG-3′ (D472N). Sanger sequencing of the samples was performed using the following primers 5′-GAAGATGCTGGAGCAGCAGC-3′ and 5′-GACAGCAGAGGCCAAGGAGC-3′ for Q333P and D472N, respectively, by the Australian Cancer Research Foundation Biomolecular Resource Facility, JCSMR and ANU.

### Transfection, IP and immunoblotting

HEK 293 or HEK 293T cells (cat. no. CRL-3216, ATCC, Invitro Technologies) were transfected (Lipofectamine 2000, Thermo Fisher Scientific) with the relevant plasmids according to the manufacturer’s recommendations. Whole-cell extracts were prepared from HEK 293 or stimulated B cells or pDCs were lysed in radioimmunoprecipitation assay buffer (1% Triton X-100, 50 mM Tris-HCl, pH 7.4, 150 mM NaCl, 0.5% sodium deoxycholate, 0.1% SDS) and centrifuged. The indicated proteins were immunoprecipitated with specific antibody using Protein G Agarose (cat. no. 16-266, Merck Millipore). Immunoprecipitants or whole-cell extracts were resuspended in SDS buffer and boiled before electrophoresis on denaturing SDS–polyacrylamide gel electrophoresis gels. Gels were transferred to nitrocellulose membranes (cat. no. 1620097, Bio-Rad Laboratories), blocked overnight (tris-buffered saline with 01% Tween 20 and skimmed milk) and probed with the relevant primary and secondary antibodies; rabbit anti-TNIP1 (cat. no. HPA037891, Sigma-Aldrich) used at 1:1000 dilution; mouse anti-actin used at 1:5,000 dilution; rabbit anti-IRAK1 used at 1:1,000 dilution; rabbit anti-MyD88 used at 1:1,000 dilution; mouse anti-alpha-tubulin used at 1:5,000 dilution; rabbit anti-IκBα used at 1:1,000 dilution; rabbit anti-phospho IκBα (Ser32) used at 1:1,000 dilution; mouse anti-rabbit IgG peroxidase conjugated, light chain specific used at 1:2,500 dilution; goat anti-mouse IgG peroxidase conjugated, light chain specific used at 1:2,500 dilution; goat anti-mouse IgG (H+L), HRP-conjugated used at 1:5,000 dilution; and goat anti-rabbit IgG (H+L) HRP-conjugated. Membranes were developed with enhanced chemiluminescence developer (Clarity Western ECL Substrate, cat. no. 170-5061, Bio-Rad Laboratories).

### Dual luciferase assays

HEK 293 cells (sourced from ATCC, cat. no. CRL-1573, Invitro Technologies; *Mycoplasma*-tested using the PlasmoTest (cat. no. rep-pt1, InvivoGen) were transfected with either an IFNβ reporter (145 ng) (a gift from J. P.-Y. Ting) containing a 130-bp region from the *IFNB* gene or the pNIFTY (45 ng) reporter plasmid comprising five NF-κB repeated transcription factor binding sites upstream of an ELAM proximal promoter to drive the expression of a firefly luciferase reporter gene. pRL-CMV (5 ng) constitutively expressing *Renilla*, was included as a transfection control with pcDNA3.1 and the indicated vectors. Cells were lysed 24 h after transfection and dual luciferase assays were performed in accordance with the manufacturer’s guidelines (Luc-Pair Duo-Luciferase HS Assay Kit, GeneCopoeia).

### Immunofluorescence staining and microscopy

Cells that adhered to coverslips were transfected with tagged constructs, fixed in 3.7% formaldehyde, then permeabilized and blocked with 5% BSA/0.1% Triton X-100 for 1 h. Staining with specific primary antibodies was carried out in blocking buffer for 1 h. Antibodies used include: mouse anti-HA used at a 1:300 dilution; rabbit anti-HA used at a 1:300 dilution; mouse anti-FLAG M2 used at a 1:200 dilution; mouse anti-myc (Ab-1) used at a 1:150 dilution; rabbit anti-TNIP1 used at a 1:100 dilution; mouse anti-SQSTM1 used at a 1:100 dilution; rabbit anti-IRAK1 used at a 1:100 dilution; rabbit anti-MyD88 used at 1:100 dilution; mouse anti-EEA1 used at a 1:100 dilution; rabbit anti-LAMP1 used at a 1:100 dilution; and goat anti-RAB7 used at a 1:100 dilution. Secondary antibodies conjugated to Alexa Fluor 568, 594 or 488 were all used at a 1:500 dilution (donkey anti-goat IgG Alexa Fluor 488; donkey anti-rabbit IgG, Alexa Fluor 488; donkey anti-mouse IgG Alexa Fluor 488; Alexa Fluor 568 donkey anti-mouse IgG; and donkey anti-rabbit IgG, Alexa Fluor 594). Cells were washed in PBS with 0.1% Tween 20 and stained with appropriate Alexa Fluor-labeled secondary antibodies before mounting in VECTASHIELD (Vector Laboratories) with DAPI. Images were captured on an Olympus IX71 inverted microscope using an Olympus PlanApo N 60× oil objective (1.42 numerical aperture/0.17 working distance) or PlanApo N 100× oil objective (1.40 numerical aperture/0.17 working distance). Transfected cells were stained with MTDR FM by diluting the stock solution in growth medium and adding to cells growing on coverslips to a final concentration of 500 nM. After incubation for 30 min at 37 °C, cells were fixed in 3.7% formaldehyde and mounted in VECTASHIELD with DAPI. Images were captured on a Leica SP5 confocal microscope with a pin hole of 95.5 μm and a HCX PL APO lambda blue 63× 1.4 oil objective, and compiled using Adobe Photoshop. Particle sizes were quantified using Fiji^45^.

### Structural modeling

As no crystal structure was available for residues 292–389, a computer model of the location of the variant within the TNIP1 structure was designed using AlphaFold predictions (AF-Q15025-F1) and modeled in PyMOL. Residues 292–389, including the TNIP1^Q333P^ variant, were submitted to ColabFold v.1.5.2 and AlphaFold2 using MMseqs2 (ref. ^46^). The highest confidence structure produced was represented in PyMOL.

### Statistical analysis

All statistical analyses were carried out using Prism v.9 (GraphPad Software). Statistically significant differences are indicated as *P* ≤ 0.05. Statistical significance was assessed on log-transformed cellular phenotyping, ELISA and Meso Scale datasets using a one-way ANOVA with post-hoc Tukey test to compare multiple treatments. A two-way ANOVA was used for the BM chimera cellular phenotyping dataset; a two-way ANOVA with a Šidák multiple-comparisons test was used for the BM-derived pDC cytokine analysis. All data were filed using Microsoft Excel and graphed using Prism. No animals or data points were excluded from the analyses. Data distribution was assumed to be normal but this was not formally tested. Aside from the histological analysis of salivary gland and kidney sections, data collection and analysis were not performed blind to the conditions of the experiments. No statistical methods were used to predetermine sample sizes but our sample sizes are similar to those reported in previous publications^2^.

### Reporting summary

Further information on research design is available in the Nature Portfolio Reporting Summary linked to this article.

## Online content

Any methods, additional references, Nature Portfolio reporting summaries, source data, extended data, supplementary information, acknowledgements, peer review information; details of author contributions and competing interests; and statements of data and code availability are available at 10.1038/s41590-024-01902-0.

## Supplementary information

Supplementary Information Supplementary Table 1, Case notes and Figs. 1–3.

Reporting Summary

## Source data

Source Data Fig. 1 Salivary gland images for Fig. 1j.

Source Data Fig. 1 Statistical source data.

Source Data Fig. 2 Statistical source data.

Source Data Fig. 3 Statistical source data.

Source Data Fig. 4 Statistical source data.

Source Data Fig. 5 Image files Fig. 5j.

Source Data Unprocessed immunoblots for Figs. 5d and 6e, and Extended Data Figs. 4a–e,j and 5i,j.

Source Data Fig. 5 Statistical source data.

Source Data Fig. 6 Statistical source data.

Source Data Fig. 6 Image files for Fig. 6a,b,d.

Source Data Fig. 7 Statistical source data.

Source Data Fig. 8 Image files for Fig. 8a–c.

Source Data Statistical source data for Extended Data Figs. 2–5.

Source Data Extended Data Fig. 2 Image files for Extended Data Fig. 2c,e.

Source Data Extended Data Fig. 5 Image files for Extended Data Fig. 5a–h.

Source Data Extended Data Fig. 6 Image files Extended Data Fig. 6a,b.

Source Data Extended Data Fig. 7 Image files Extended Data Fig. 7a.

## Data Availability

The genomic data relating to the families in Fig. 1 and Supplementary Table 1 have been submitted to the Sequence Read Archive under accession nos. SAMN33490700 (A.III.2(CPI119)), SAMN33490701 (A.II.1(CPI122)) and SAMN33490702 (A.II.2(CPI123)). Source data are provided with this paper.
