# Supplementary material for: A TNIP1-driven systemic autoimmune disorder with elevated IgG4
Source: Nat Immunol. 2024 Jul 26;25(9):1678–91. doi: 10.1038/s41590-024-01902-0 (PMC11362012; doi:10.1038/s41590-024-01902-0)
Supplement: Supplementary file 1 — Supplementary Table 1, Case notes and Figs. 1–3. [file 41590_2024_1902_MOESM1_ESM.pdf]

nature immunology

Supplementary information

https://doi.org/10.1038/s41590-024-01902-0

A *TNIP1*-driven systemic autoimmune disorder with elevated IgG4

In the format provided by the authors and unedited

Supplementary Material

Supplementary Table 1. Patient clinical information

ID	Sex	Ethnicity	Age of Onset	Clinical and Laboratory Manifestation
A.III.2	Female	Australian/ Caucasian	8	Presented with dry eyes, mouth ulcers, malar rash, fatigue, abdominal pain, diarrhoea and arthralgia. Serological investigations revealed polyclonal hypergammaglobulinaemia of IgG from an early age (including disproportionate increase in IgG4), positive Coomb's test, anti-nuclear antibodies (ANAs) (1:80), SSB antibodies and autoimmune hypothyroidism (thyroid peroxidase, thyroglobulin antibodies). Colonoscopy and upper endoscopy revealed lymphoid hyperplasia without mucosal ulceration or inflammation. The patient's health complications include severe gastroparesis and chronic non-infective cystitis.<br><br>Medications: Alfacalcidol, Azathioprine, Hydroxychloroquine, Levofloxacin, Mycophenolate Mofetil, Prednisone
A.II.2	Female	Australian/ Caucasian	Unknown	Antithyroglobulin antibody positive and under investigation for CREST syndrome after autoimmune serology revealed a high titre of centromere antibodies (1:5120)
B.II.1	Female	Chinese Han/ East Asian	47	Diagnosed with systemic lupus erythematosus (SLE) featuring arthritis, ANAs (1:640), anti-dsDNA (99.45) and anti-SSA, beta-2 glycoprotein antibodies and lupus anticoagulant. The patient has hypergammaglobulinemia of IgG, IgG1, IgG4 and IgE, elevated urine protein of >500g/24hr and low complement proteins (low C3, low C4). In 2014 the patient had surgery for thyroid tumours.<br><br>Medications: Levofloxacin, Hydroxychloroquine Sulfate, Prednisone Acetate, Alfacalcidol

Case notes for A.III.2

Female patient presented at age 8 with abdominal pains, fatigue, diarrhoea and malar rash. She also reported arthralgia of large and small joints. She had dry eyes and mouth ulcers.

Family history revealed that the mother also had autoimmune thyroid disease (thyroglobulin antibody positive) and was under investigation for CREST syndrome after autoimmune serology revealed a high titre of centromere antibodies (1:5120). Her maternal aunt reported Raynaud's phenomenon. Maternal grandmother had been diagnosed with multisystem atrophy.

Investigations of the proband revealed evidence of autoimmune hypothyroidism (antithyroglobulin antibody positive) and she was commenced on thyroxine replacement therapy. Colonoscopy and upper endoscopy, which revealed lymphoid hyperplasia but no mucosal ulceration or inflammation.

Serology revealed low titre ANA (1:80) and tests for dsDNA antibodies, antibodies to extractable nuclear antigens, and rheumatoid factor were negative. She had marked polyclonal hypergammaglobulinaemia of IgG, initially accounted for by an increase in all four subclasses:

IgG	19.8g/L	(6.2-14.4)
IgG1	13.3	(3.42-11.50)
IgG2	5.83	(1.0-4.55)
IgG3	2.38	(0.28-1.25)
IgG4	1.73	0.04-1.36)

She was treated with hydroxychloroquine and a short course of glucocorticoids and her symptoms resolved. Shortly afterwards, her symptoms settled and she was commenced on azathioprine to good effect.

Clinical manifestations through adolescence consisted of joint pains and intermittent abdominal pains. Investigations revealed evolution of immunoglobulin abnormalities with increase in total IgG to between 20 and 25g/L, with increase of IgG to approximately 25% beyond the upper limit of normal, and persistent increase of IgG4 to approximately twice the upper limit of normal. She declined a minor salivary gland biopsy.

Azathioprine was ceased after when she was 15 years old and over the next 12 months her symptoms slowly returned, with arthralgia, fatigue and Raynaud's phenomenon. Azathioprine was recommenced and her symptoms settled. She then developed headaches. Brain imaging revealed no abnormalities.

At age 18 she developed symptoms suggesting recurrent urinary tract infections, but urine was consistently sterile. She went on to have a cystoscopy, which showed macroscopic features of lymphocytic inflammation and trigonitis.

Two years later she developed additional abdominal symptoms of pain and bloating. Radionuclide gastric emptying study showed markedly reduced gastric emptying. She received a 6 month trial of IVIg. Symptoms improved and follow up gastric emptying returned to normal.

In her 20s, symptoms became more severe again and she was changed to mycophenolate. This brought about an excellent response, with complete symptoms resolution. IgG and IgG4 remained elevated. She remains on MMF at the current time.

Supplementary Figure 1

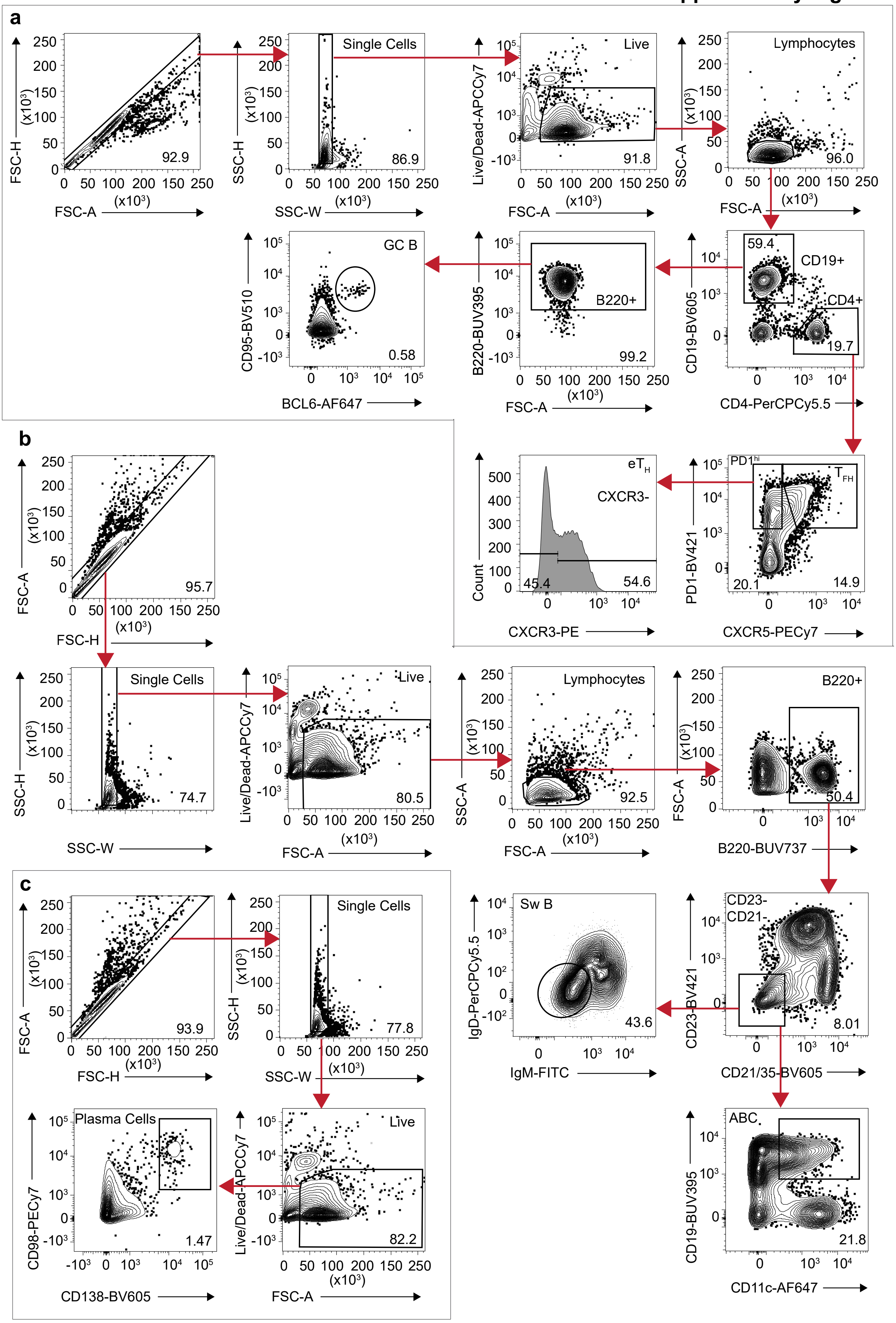

Supplementary Figure 1. Gating strategy used to characterise *vikala* immune cell populations.

a-c, Gating strategies used to define T follicular helper cells, (T_{FH}), extrafollicular helper T cells (eT_H) and germinal center (GC) B cells (**a**); switched B (Sw B) cells and age-associated B cells (ABC) (**b**); and plasma cells (PC) in *vikala* mice (**c**).

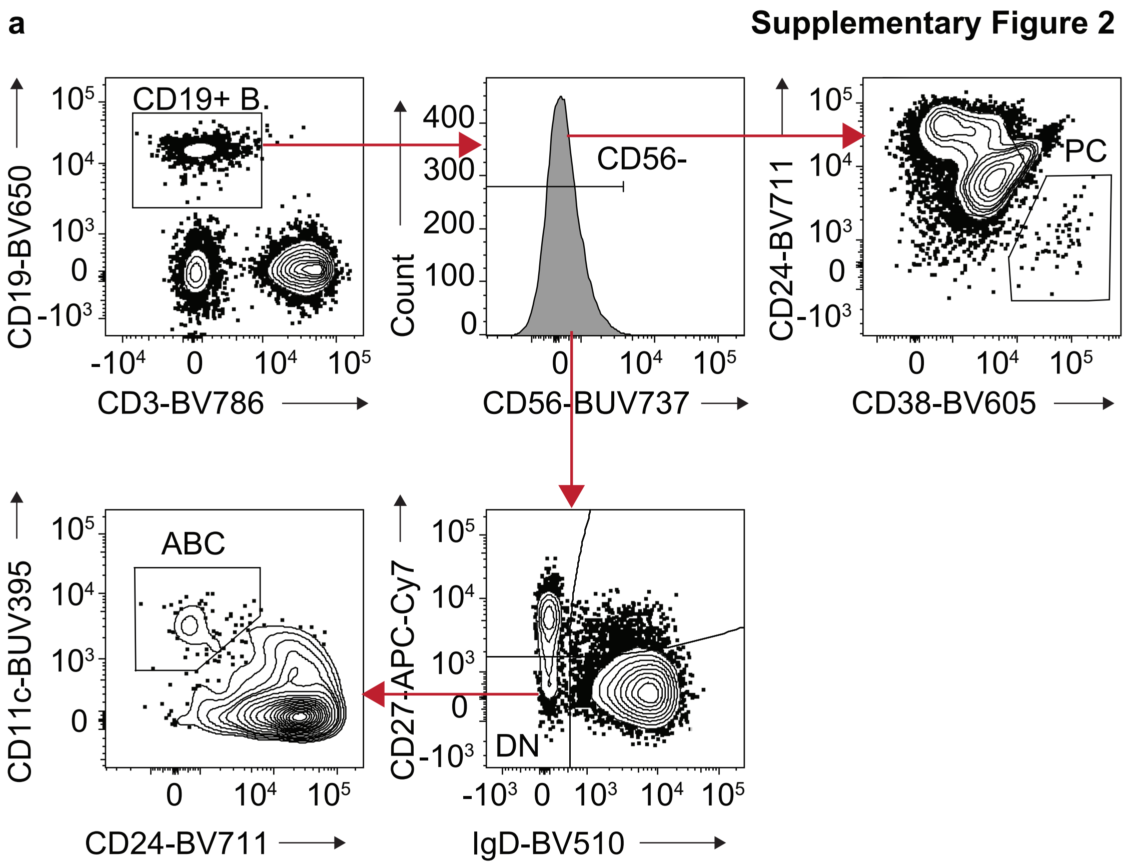

Supplementary Figure 2. Gating strategy for human PBMC B cell subsets.

a. Gating strategy used to define human plasma cells (PC) and age-associated B cells (ABC). Pre-gated on live, singlet, lymphocytes.

a **Supplementary Figure 3**

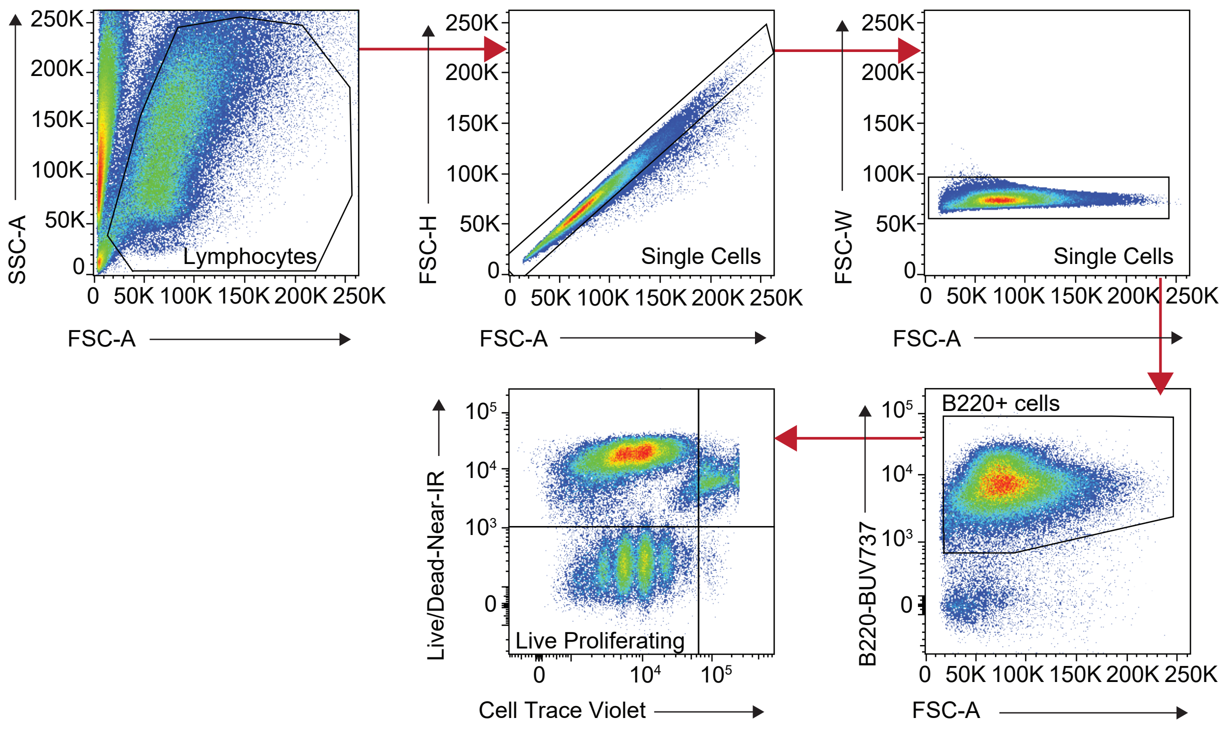

Supplementary Figure 3. Gating strategy used to quantify proliferating live B cells

a. Gating strategy to define live proliferating B cells following 72h culture with soluble or bead-conjugated CpG and IgM alone or in combination.
