# Supplementary material for: A TNIP1-driven systemic autoimmune disorder with elevated IgG4
Source: Nat Immunol. 2024 Jul 26;25(9):1678–91. doi: 10.1038/s41590-024-01902-0 (PMC11362012; doi:10.1038/s41590-024-01902-0)
Supplement: Supplementary file 2 — Reporting Summary [file 41590_2024_1902_MOESM2_ESM.pdf]

nature portfolio

Corresponding author(s): Carola G. Vinuesa

Last updated by author(s): Jun 17, 2024

Reporting Summary

Nature Portfolio wishes to improve the reproducibility of the work that we publish. This form provides structure for consistency and transparency in reporting. For further information on Nature Portfolio policies, see our Editorial Policies and the Editorial Policy Checklist.

Statistics

For all statistical analyses, confirm that the following items are present in the figure legend, table legend, main text, or Methods section.

n/a	Confirmed	
☐	☒	The exact sample size (*n*) for each experimental group/condition, given as a discrete number and unit of measurement
☐	☒	A statement on whether measurements were taken from distinct samples or whether the same sample was measured repeatedly
☐	☒	The statistical test(s) used AND whether they are one- or two-sided<br>*Only common tests should be described solely by name; describe more complex techniques in the Methods section.*
☐	☒	A description of all covariates tested
☐	☒	A description of any assumptions or corrections, such as tests of normality and adjustment for multiple comparisons
☐	☒	A full description of the statistical parameters including central tendency (e.g. means) or other basic estimates (e.g. regression coefficient) AND variation (e.g. standard deviation) or associated estimates of uncertainty (e.g. confidence intervals)
☐	☒	For null hypothesis testing, the test statistic (e.g. *F*, *t*, *r*) with confidence intervals, effect sizes, degrees of freedom and *P* value noted<br>*Give P values as exact values whenever suitable.*
☒	☐	For Bayesian analysis, information on the choice of priors and Markov chain Monte Carlo settings
☒	☐	For hierarchical and complex designs, identification of the appropriate level for tests and full reporting of outcomes
☒	☐	Estimates of effect sizes (e.g. Cohen's *d*, Pearson's *r*), indicating how they were calculated

Our web collection on statistics for biologists contains articles on many of the points above.

Software and code

Policy information about availability of computer code

Data collection	A Fortessa or Fortessa X-20 cytometer was used to collect all flow cytometric cellular phenotyping data and version 8.0 FACSDiva (BD, Biosciences) was used during data acquisition.
Data analysis	Prism 9, GraphPad Software LLC was used to perform all statistical analysis in this study. Luciferase assays, Meso Scale, qPCR, ELISA, and cellular phenotyping statistics were based on one-way ANOVA with Tukey's multiple comparison. Two-way ANOVA was used to evaluate statistical significant in bone marrow chimera phenotyping, BM-pDC and B-cell stimulation experiments. Flow cytometry data was analyzed using the FlowJo software v10 (FlowJo LLC). Data was filed in Microsoft Excel version 2405. Immunoblots and immunofluorescent images were quantified using Fiji ImageJ version 2.1.0/1.53c.

For manuscripts utilizing custom algorithms or software that are central to the research but not yet described in published literature, software must be made available to editors and reviewers. We strongly encourage code deposition in a community repository (e.g. GitHub). See the Nature Portfolio guidelines for submitting code & software for further information.

Data

Policy information about availability of data

All manuscripts must include a data availability statement. This statement should provide the following information, where applicable:
- Accession codes, unique identifiers, or web links for publicly available datasets
- A description of any restrictions on data availability
- For clinical datasets or third party data, please ensure that the statement adheres to our policy

Genomic data relating to the families in Figure. 1 and Supplementary Table. 1 have been submitted to the NCBI SRA Biosample database and are available under data accession codes: SAMN33490700 (A.III.2(CPI119)), SAMN33490701 (A.II.1(CPI122)) and SAMN33490702 (A.II.2(CPI123)).

Research involving human participants, their data, or biological material

Policy information about studies with human participants or human data. See also policy information about sex, gender (identity/presentation), and sexual orientation and race, ethnicity and racism.

Reporting on sex and gender	Human participants were selected based on harboring the genetic variant of interest which in this case was identified in two female patients and a female family member. Patient, and where applicable, family member consent was obtained prior to enrollment in this study. Sex and/or gender of probands were not relevant for inclusion in this study.
Reporting on race, ethnicity, or other socially relevant groupings	The ethnicity/race of probands included in this study are self-reported. Ethnicity/race data was obtained through the China and Australian Centers for Personalised Immunology patient recruitment process.
Population characteristics	Participants were either individuals with systemic autoimmune disease or their family members, and healthy blood donor controls. Patient medical treatments and clinical diagnosis are provided in Table S1.
Recruitment	Patients were recruited by referring medical practitioners based on meeting clinical eligibility criteria. Healthy blood donors ranged in age from 26 to 71 years of age and were predominantly female (18/22 female and 4/22 male) for the Australian cohort. The CACPI healthy control cohort ranged from 25 to 60 years and comprised an all female cohort (8).
Ethics oversight	Written informed consent was obtained from participants enrolled in the Centre for Personalised Immunology Program. This study was approved by and abides by the ethical regulations of the Australian National University and ACT Health Human Ethics Committees, or by the Renji Hospital Ethics Committee of Shanghai Jiaotong University School of Medicine.

Note that full information on the approval of the study protocol must also be provided in the manuscript.

Field-specific reporting

Please select the one below that is the best fit for your research. If you are not sure, read the appropriate sections before making your selection.

☒ Life sciences ☐ Behavioural & social sciences ☐ Ecological, evolutionary & environmental sciences

For a reference copy of the document with all sections, see nature.com/documents/nr-reporting-summary-flat.pdf

Life sciences study design

All studies must disclose on these points even when the disclosure is negative.

Sample size	Statistical methods were not used to pre-determine sample sizes for all experiments but our sample sizes are similar to those reported in previous publications (1)<br>1. Brown, G.J. et al. TLR7 gain-of-function genetic variation causes human lupus. Nature 605, 349-356 (2022).
Data exclusions	Data was not excluded.
Replication	Fig. 1a-f patient pedigrees, sequence conservation, structural modeling and patient serological data - experimental replication not feasible<br>Fig. 1g,h are representative of n=2 experiments<br>Fig. 1i Immunoglobulin mesoscale data was performed once (IgG2c subtype elevation in vik/vik compared to wildtype confirmed via ELISA). Although the entire Mesoscale Discovery Ig multiplexed screen was only undertaken once due to cost, Ig of interest were measured in separate ELISA experiments confirming the Mesoscale data.<br><br>Fig. 1j-k. histological scoring performed on salivary gland sections from n=29 mice (+/+=10, vik/+=11, and vik/vik=8 mice). For H&E histology evaluation, the important factor is to evaluate sufficient numbers of mice, provided that the H&E stain works. We evaluated n=29 mice.<br><br>Fig. 2 a-f are representative of n=2 experiments<br>Fig. 2 g-l bone marrow chimera experiment was conducted once. This experiment included 20 mice, all mice behaving in the expected trend (no outliers), which is identical to the mixed bone marrow chimera results we have previously produced for other Tlr7-dependent systemic autoimmunity models.

Fig 2. m-n human PMBC phenotyping experiments were performed once. Human PBMC phenotyping was limited by the samples available in the biobank, which were used up in their entirety for the phenotyping shown and probands could not be recalled.

Fig 3. a-f are representative of n=2 experiments
Fig 3. g-h were performed once. a-DNA ELISA, IgG2c ELISA on Myd88KO serum-serology of an independent cohort of Tnip1 mutant x MyD88 KO could not be repeated as the strain was culled during the COVID shutdown. The cross was not set up again results were identical to the Tnip1 mutant x TLR7 KO mice, which acts upstream of MyD88, and thus provided confirmation that an intact TLR7-MyD88 KO pathway is necessary for the observed serological phenotype.

Fig 4. a-f are representative of n=2 experiments

Fig. 5 a-c are representative of n=3 experiments, D472N TNIP1 positive control in Fig. 5 a, b was included in 1 of 3 repeat experiments
Fig. 5 d representative of n=2 experiments
Fig. 5 e-h are representative of n=3 experiments
Fig 5 i-j are representative of n=3 experiments
Fig 5 k (quantification of j)

Fig 6. a-b are representative n=3experiments
Fig 6. c quantification representative of n=3 experiments.
Fig 6. d-e are representative n=2 experiments

Fig 7a-b representative of n=2 experiments
Fig 7c-d representative n=3 experiments

Fig. 8a-b are representative of n=2 experiments
Fig. 8c Representative salivary gland sections from n=3 mice analysed (+/+=1, vik/vik=2)
Fig 8d (diagram - replication n/a)

Ext. 1 a-d replication not applicable

Ext. 2 a representative of n=2 experiments
Ext. 2 b-c were performed once. Anti-DNA serology comparing same mice at 10 and 24 weeks. In essence, the 24 week is a repeat of the 10 week, showing the same trend.
Ext. 2 d-e histological scoring performed once on kidney sections from n=28 mice (+/+=10, vik/+=9, and vik/vik=9 mice). For H&E histology evaluation, the important factor is to evaluate sufficient numbers of mice, provided that the H&E stain works. We evaluated n=28 mice.

Ext. 2 f-g spleen organ weights measured once for mice at 12 weeks and once at 24 weeks. Given that the 12 week was essentially a negative result (no difference in spleen mass), an independent experiment with mice culled at 24 weeks was essentially a repeat, with again no difference shown.

Ext 3. a-f experiments representative of n=2 experiments
Ext 3 g-h total cell numbers from cellular phenotyping representative of n=2

Ext. 4 a-e, j are representative of n=2 experiments
Ext. 4 f, i are representative of n=3 experiments

Ext. 5 a,c,f, h,i and j are representative of n=2 experiments
Ext. 5 b is representative of n=3 experiments
Ext. 5 g experiment was performed once since there was no colocalisation evident with rab7.

Ext. 6 a-b are representative of n=2 experiments

Ext. 7a displays salivary gland sections from one +/+ and two vik/vik mice, respectively. As for other pathology assessments, there is no possibility of technical error, the important factor is to include several mice in the assessment and show the original unedited photographs.

Randomization

For in vitro experiments, randomisation was not required given there were no relevant covariates (i.e. cells from littermate mice came from the same cage, all wells treated simultaneously using multi-channel pipettes, on the same day, in the same single plate, analysed in the same machine, handled by the same investigator). For other animal experiments mice were randomly allocated into cages and experimental cohorts.

Blinding

Blinding to allocation occurred for all experiments in which the investigator had to score data manually (i.e. intensity and pattern of ANA fluorescence, analysis of histological samples from mouse necropsies, assessment of Ig deposits in EM kidney sections). Blinding did not occur for assays in which a pre-determined order was required for loading gels plus the result would be presented raw to the reader (i.e. western blot gels) or analysed via an automated machine without input from the investigator (i.e. quantification of luciferase activity). Investigators planned mouse experiments based on genotype and grouping, but during performance of experiments mice were identified only by randomly assigned number with investigators blind to group allocation.

Reporting for specific materials, systems and methods

We require information from authors about some types of materials, experimental systems and methods used in many studies. Here, indicate whether each material, system or method listed is relevant to your study. If you are not sure if a list item applies to your research, read the appropriate section before selecting a response.

Materials & experimental systems

n/a	Involved in the study
☐	☒ Antibodies
☐	☒ Eukaryotic cell lines
☒	☐ Palaeontology and archaeology
☐	☒ Animals and other organisms
☒	☐ Clinical data
☒	☐ Dual use research of concern
☒	☐ Plants

Methods

n/a	Involved in the study
☒	☐ ChIP-seq
☐	☒ Flow cytometry
☒	☐ MRI-based neuroimaging

Antibodies

Antibodies used

Antibodies for immunoblotting and coimmunoprecipitation studies were as follows: mouse anti-HA (H3663, clone HA-7, Sigma Aldrich); rabbit anti-HA (H6908, Sigma Aldrich); mouse anti-FLAG M2 (F1804; Sigma Aldrich); mouse anti-myc (Ab-1; clone 9E10, OP1-200UG, Merck Millipore); rabbit anti-TNIP1 (Sigma Aldrich, HPA037893), mouse anti-actin (JLA20, Developmental Studies Hybridoma Bank, The University of Iowa), mouse anti-alpha-tubulin (B-5-1-2, ThermoFisher #32-2500), rabbit anti-IkB (Cell Signaling Technology #9242), rabbit anti-phospho IkB (Ser32) (14D4, Cell Signaling Technology #2859), mouse anti-SQSTM1 (ab56416, Abcam); rabbit anti-IRAK1 (D51G7, Cell Signaling Technology, 4504), rabbit anti-MYD88 (Cell Signaling Technology, 4283), mouse anti-EEA1 (E7659, clone N19, Sigma Aldrich), rabbit anti-LAMP1 (ab24170, Abcam); rabbit anti-RAB7 (C-19, sc-6563, Santa Cruz Biotechnology) Secondary antibodies were conjugated to HRP (Mouse Anti-Rabbit IgG Peroxidase conjugated, Light Chain Specific (Jackson ImmunoResearch #211-032-171) used at 1:2500, Goat Anti-Mouse IgG Peroxidase conjugated, Light Chain Specific (Jackson ImmunoResearch #115-035-174) used at 1:2500, Goat anti-mouse IgG, HRP conjugated (ThermoFisher #62-6520) used at 1:5000, Goat anti-Rabbit IgG, HRP conjugated (ThermoFisher #65-6120)), Alexa Fluor 568 or Alexa Fluor 488 (Molecular Probes, Invitrogen). For immunoprecipitation 2g of primary antibodies were used. Antibodies used for immunofluorescence imaging included: mouse anti-HA (H3663, clone HA-7, Sigma Aldrich) used at 1:300; rabbit anti-HA (H6908, Sigma Aldrich) used at 1:300; mouse anti-FLAG M2 (F1804; Sigma Aldrich) used at 1:200; mouse anti-myc (Ab-1)(clone 9E10, OP1-200UG, Merck Millipore) used at 1:150; rabbit anti-TNIP1 (Sigma Aldrich, HPA037893) used at 1:100, mouse anti-SQSTM1 (ab56416, Abcam) used at 1:100; rabbit anti-IRAK1 (D51G7, Cell Signaling Technology, 4504) used at 1:100, rabbit anti-MYD88 (Cell Signaling Technology, 4283) used at 1:100, mouse anti-EEA1 (E7659, clone N19, Sigma Aldrich) used at 1:100, rabbit anti-LAMP1 (ab24170, Abcam) used at 1:100, goat anti-RAB7 (C-19, sc-6563, Santa Cruz Biotechnology) used at 1:100. Secondary antibodies conjugated to Alexa Fluor 568, 594 or 488 were all used at 1:500 (Donkey anti-Goat IgG Alexa Fluor 488, Invitrogen, A-11055; Donkey anti Rabbit IgG, Alexa Fluor 488, Invitrogen, A-21206; donkey anti-mouse IgG Alexa Fluor 488, Invitrogen, A21202; Alexa Fluor 568 donkey anti-mouse IgG, Invitrogen, A10037; Donkey anti-Rabbit IgG, Alexa Fluor 594, A-21207).

Antibodies and dyes used for staining mouse tissues for flow cytometry include: Annexin V-FITC (BD Pharmingen #560931, 1:100), B220-Alexa Fluor 647 (RA3-6B2, BD Pharmingen #557683, 1:400), B220-BUV395 (RA3-6B2, BD Horizon #563793, 1:200), B220-BUV737 (RA3-6B2, BD Horizon #612838, 1:200), BCL6-A467 (K112-91, BD Pharmingen #561525, 1:40), BST2-PE (927, BioLegend #127010, 1:400), CCR7-PerCP-Cy5.5 (4B12, BioLegend 120116, 1:50), CD3-Alexa Fluor 700 (17A2, BioLegend #100216, 1:200), CD4-Alexa Fluor 647 (RM4-5, BioLegend #100530,1:400), CD4-BUV395 (6K1.5, BD Horizon, #563552 1:200), CD4-PerCP-Cy5.5 (RM4-5, BioLegend #116012, 1:400), CD8-BUV805 (53-6.7, BD Horizon #612898, 1:200), CD11b-PerCP-Cy5.5 (M1/70, BioLegend #101228, 1:400), CD11c-Alexa Fluor 647 (N418, BioLegend #117312, 1:200), CD11c-BV510 (N418, BioLegend #117353,1:400), CD11c-FITC (N418, BioLegend #117305, 1:800), CD19-Alexa Fluor 700 (eBio1D3, Invitrogen # 56-0193-82, 1:200), CD19-BV605 (6D5, BioLegend #115540, 1:400), CD19-BUV395 (1D3, BD Horizon #563557, 1:200), CD21/35-BV605 (7G6, BD Horizon, 1:400V), CD23-BV421 (B3B4, BioLegend, 1:400), CD25-APC (PC61, BioLegend #102012, 1:200), CD25-PE (PC62, BioLegend, 1:100), CD44-FITC (IM7, BD Pharmingen #563176, 1:50), CD44-Pacific Blue (IM7, BioLegend #103020, 1:400), CD45.2-PerCP-Cy5.5 (104, BD Biosciences # 552950, 1:200), CD45.1-BV605 (A20, BioLegend #110737, 1:100), CD45.1-BV711 (A20, BioLegend #110739, 1:200), CD95 (FAS)-BV510 (Jo2, BD Horizon #563646, 1:200), CD98-PE-Cy7 (RI.388, BioLegend #128214, 1:200), CD138-PE (281-2, BD Pharmingen # 561070, 1:400), CXCR3-PE (CXCR3-173, BioLegend #126506, 1:400), CXCR5-Biotin (2G8, BD Biosciences #551960, 1:40), FOXP3-FITC (FJK-16s, eBioscience #11-5773-82, 1:200), FOXP3-PE-Cy7 (FJK-16s, eBioscience #25-5773-82, 1:400), IA/IE-BV421 (M5/114.15.2, BioLegend #107631, 1:800), IgD-PerCP-Cy5.5 (11-26c.2a, BD Pharmingen #564273, 1:400), IgD-PE (11-26c.2a, BioLegend #405705, 1:800), IgM-FITC (II/41, BD Pharmingen #553437, 1:200), IgM-PE-Cy7 (II/41, Invitrogen #25-5790-82, 1:400), PD1-BV421 (29F.1A12, BioLegend #135217, 1:200), Ly6C-Biotin (BD Pharmingen #557359 1:200), Ly6G-FITC (Biolegend, #127606, 1:200), SiglecH-APC (551, BioLegend #129611, 1:200), streptavidin-BV510 (BioLegend #405233, 1:400), streptavidin-PE-Cy7 (#25-4317-82, eBioscience, 1:400), Live Dead APC-Cy7 (eFluor 780)(eBioscience #65-0865-18, 1:1000), LIVE/DEAD Fixable Aqua Dead Cell Stain (Invitrogen #L34957, 1:1000), Fc Block CD16/CD32 (2.462, BD Pharmingen #553141, 1:100) Cell Trace Violet (Molecular Probes, #C34557, manufacturer's recommendations).
Antibodies used to stain human PBMCs include: CD11c-BUV395 (B-ly6, BD Biosciences #563787, 1:50), CD11c-BV510 (B-ly6, BD Biosciences #563026, 1:25), CD127-BB700 (HIL-7R-M21, BD Biosciences #566398, 1:25), CD19- APC-Cy7 (SJ25C1, BD Biosciences #348794, 1:50), CD19-BV650 (HIB19, BioLegend #302238, 1:50), CD24-BV605 (ML5, BioLegend #311124, 1:25), CD24-BV711 (ML5, BD Biosciences #563401, 1:50), CD25-APC-R700 (2A3, BD Biosciences #565106, 1:50), CD27-PE-Cy7(M-T271, BD Biosciences #560609, 1:20), CD27-APC-eFluor780 (O323, eBiosciences #47-0279, 1:50), CD38-APC (HB-7, BD Biosciences #345807, 1:20), CD38-BV605 (HIT2, BioLegend #303532, 1:25), CD3-BV786 (SK7, BD Biosciences #563799, 1:200), CD3-FITC (UCHT1, BioLegend #300406, 1:50), CD45RA- PE-Cy7 (HI100, eBioscience #25-0458-73, 1:50), CD45RA-Pacific Blue (HI100, BioLegend #304123, 1:100), CD4-BUV496 (SK3, BD Biosciences #564651, 1:100), CD56-BUV737 (NCAM16.2, BD Pharmingen #564447, 1:400), CD8-BV421 (RPA-T8, BD Biosciences #562428, 1:100), CXCR3-PE (G025H7, BioLegend #353706, 1:50), CXCR5-Alexa Fluor 647 (RF8B2, BD Biosciences #558113, 1:50), IgD-BV421 (IA6-2, BD Biosciences #562518, 1:50), IgD-BV510 (IA6-2, BioLegend #348220, 1:30), LIVE/DEAD Fixable Blue Dead Cell Stain (Invitrogen #L23105, 1:1000), LIVE/DEAD Stain Kit Green Fluorescent (Invitrogen #L23101, 1:1000) PD1- PE-

CF594 (EH12.2H7, BioLegend #329940, 1:50).

Validation

All antibodies used in the study were commercially bought and any validation statements are noted on the manufacturer's websites. This work includes over 100 antibodies that have been extensively used and validated by the indicated manufacturers and no new antibodies were generated or used in this study.

Eukaryotic cell lines

Policy information about cell lines and Sex and Gender in Research

Cell line source(s)	HEK293 and HEK293T cells were sourced from the American Type Culture Collection (ATCC). HEK293 cells (Invitro Technologies #ATCC CRL-1573) and HEK293T cells (Invitro Technologies #ATCC CRL-3216)
Authentication	The cell line used has not been authenticated by STR profiling.
Mycoplasma contamination	PlasmoTestTM (Invivogen) Cell lines used in this study were negative for mycoplasma.
Commonly misidentified lines (See ICLAC register)	The cell line used is not listed in the database of commonly misidentified cell lines.

Animals and other research organisms

Policy information about studies involving animals; ARRIVE guidelines recommended for reporting animal research, and Sex and Gender in Research

Laboratory animals

Mice used in this study were C57BL/6 Ncrl and B6.129-Rag1tm1Mom (Rag1−/−) mice were used as recipients in the BM chimera experiment and C57BL/6-Ptprca mice were used as WT (CD45.1) donors. The mice were cohoused by litters and. Both male and female mice were used and the sex has been identified in the figure legends. CFW/crl female mice from 6-16 weeks of age were used to mate with stud males for generation of CRISPR/Cas9 TNIP1 gene edited mice.

The ages of mice used in each experiment are specified in the figure legends and are indicated below:
Fig. 1g 20-28 week-old male (n=8) and female (n=19) vikala mice; Tnip1+/+(n=6), Tnip1vik/+(n=14) and Tnip1vik/vik (n=7) mice
Fig 1h 12 weeks-old male (n=10) and female (n=10) vikala mice
Fig. 1i I20-22 week-old male (n=9) and female (n=9) vikala; Tnip1+/+(n=6), Tnip1vik/+(n=6) and Tnip1vik/vik (n=6).
Fig. 1j-k. 20-28 week-old vikala mice; Tnip1+/+(n=10), Tnip1vik/+(n=11) and Tnip1vik/vik (n=8).

Fig. 2 a-f 22-28 week-old male (n=8) and female (n=19) vikala mice; Tnip1+/+(n=6), Tnip1vik/+(n=14) and Tnip1vik/vik (n=7)
Fig. 2 g-l bone marrow chimera experiment of 20 female mice aged to 25-27 weeks.

Fig 3. a-female (n=12) and female (n=17) vikala mice aged 16-20 weeks:
Fig 3. g-h male and female mice aged 16-20 weeks:

Fig 4. a-f mice were aged 20-30 weeks

Fig. 5d cell lysates were from 20 week old mice
Fig 5 i from mice aged 16-20 weeks of age

Fig 7a-d mice were 6-12 weeks of age

Fig. 8c mice were 16 weeks old

Ext. 2 a,c 12 week-old mice
Ext. 2 b 10 and 24 week-old mice
Ext. 2 d,e mice were 20-28 weeks of age
Ext. 2 f-g 12 and 24 week-old mice

Ext 3. a, b, e, f used 20-28 week old mice
Ext 3 c, d 20-week-old (c) and 20–28-week-old (d) mouse cohorts
Ext 3 g-h 16-20 MyD88 or 20-30 week-old Tlr7 mice

Ext. 4 a, d B cells from 40-week (a, d)
Ext 4c 28-29-week old mice
Ext 4 b 10-12-week old mice
Ext. 4 f, 16-20 week-old mice
Ext 4 e, j bone marrow derived pDCs from 16-20 week-old mice

Ext. 5 i and j BMDM were from mice 20 week-old male mice

Ext. 7a salivary gland sections were from 16 week old mice

Wild animals

Wild animals were not used in this study.

Reporting on sex	A combination of male and female mice were used in this study. Sex based analysis was not performed throughout this study as we did not observe differences based on cellular phenotype or ELISA data sets between male and female mice across the age ranges used in the study.
Field-collected samples	Field-collected samples were not used in this study.
Ethics oversight	Experiments involving animals were conducted in accordance with the Australian National University Animal Experimentation Ethics Committee regulations.

Note that full information on the approval of the study protocol must also be provided in the manuscript.

Plants

Seed stocks	n/a
Novel plant genotypes	n/a
Authentication	n/a

Flow Cytometry

Plots

Confirm that:

☒ The axis labels state the marker and fluorochrome used (e.g. CD4-FITC).

☒ The axis scales are clearly visible. Include numbers along axes only for bottom left plot of group (a 'group' is an analysis of identical markers).

☒ All plots are contour plots with outliers or pseudocolor plots.

☒ A numerical value for number of cells or percentage (with statistics) is provided.

Methodology

Sample preparation	Human PBMCs were isolated using Ficoll-Paque gradient centrifugation and frozen thawed before staining for flow cytometric analysis. Single cell suspensions were prepared from mouse spleens, Fc receptors blocked and cells stained with fluorochrome conjugated antibodies.
Instrument	Splenocytes and human PBMC samples were acquired on a Fortessa or Fortessa X-20 cytometer.
Software	FlowJo version 10.6.1 (FlowJo LLC) was used to analyse all flow cytometric data
Cell population abundance	Sample purity was based on flow cytometry sorting analysis and stringent gating (supplementary figure showing gating strategies). Abundance of populations are indicated in the flow cytometry gating figures of the manuscript.
Gating strategy	SC-H/FSC-A (cells were gated using a diagonal gating strategy to exclude cells with disproportional FSC-H and FSC-A size), SSC-W/SSC-H (cells with large SSC-W from scatter were eliminated), FSC-A/Live dead (cells staining negative for the live/dead marker were selected as "live") and FSC-A/SSC-A (Cells were gated as lymphocytes if they had a lower size and granularity relative to other signals detected). Once cells were established as singlets, live and lymphocytes analysis was completed as described in the manuscript, where possible biphasic populations were used to identify positive and negative populations.

☒ Tick this box to confirm that a figure exemplifying the gating strategy is provided in the Supplementary Information.
