# Supplementary figures and images for: A TNIP1-driven systemic autoimmune disorder with elevated IgG4 (part 1 of 2)
Source: Nat Immunol. 2024 Jul 26;25(9):1678–91. doi: 10.1038/s41590-024-01902-0 (PMC11362012; doi:10.1038/s41590-024-01902-0)

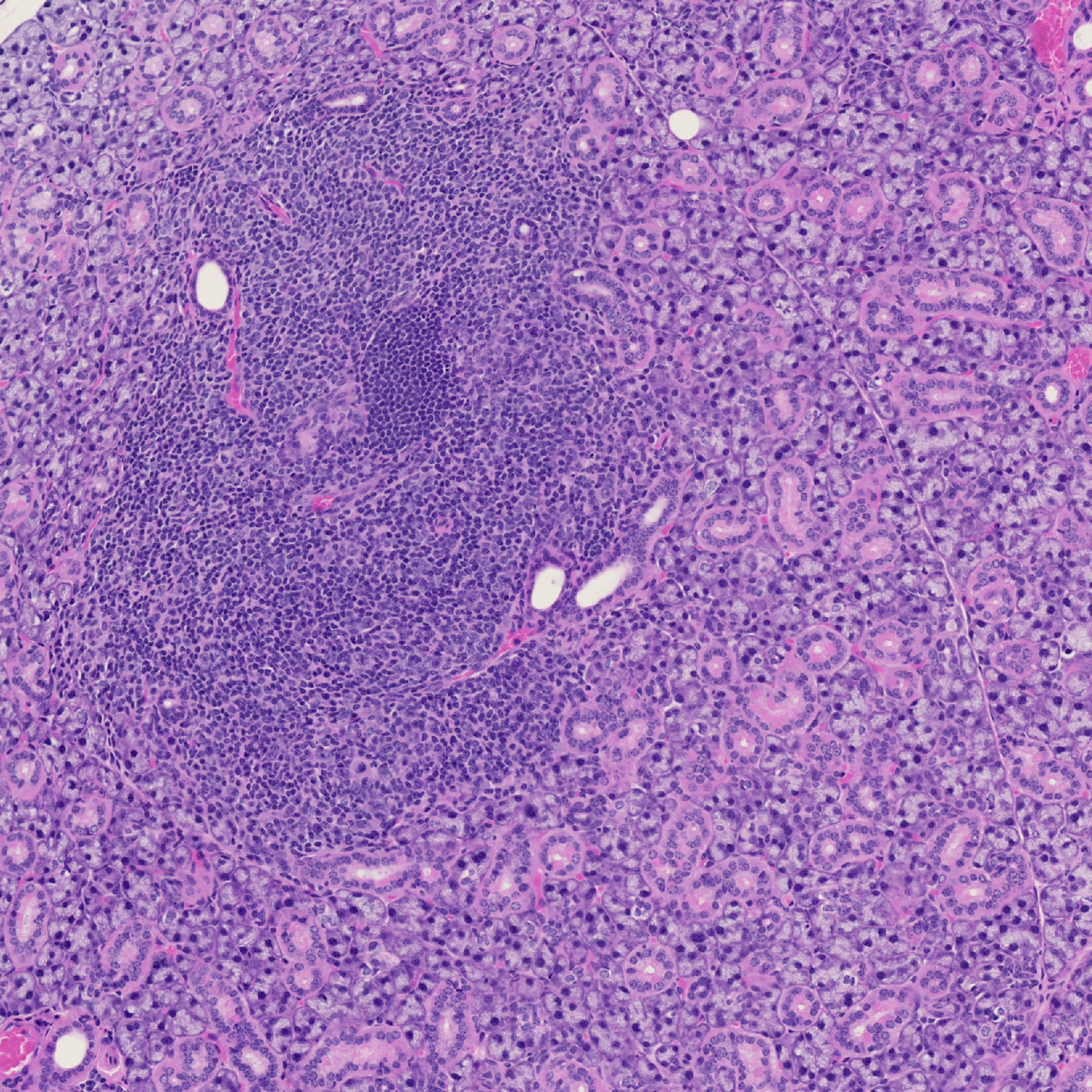

Supplement: Supplementary file 3 — Salivary gland images for Fig. 1j. [file 41590_2024_1902_MOESM3_ESM.zip › Fig 1j Homozygous SG Vikala.tif]

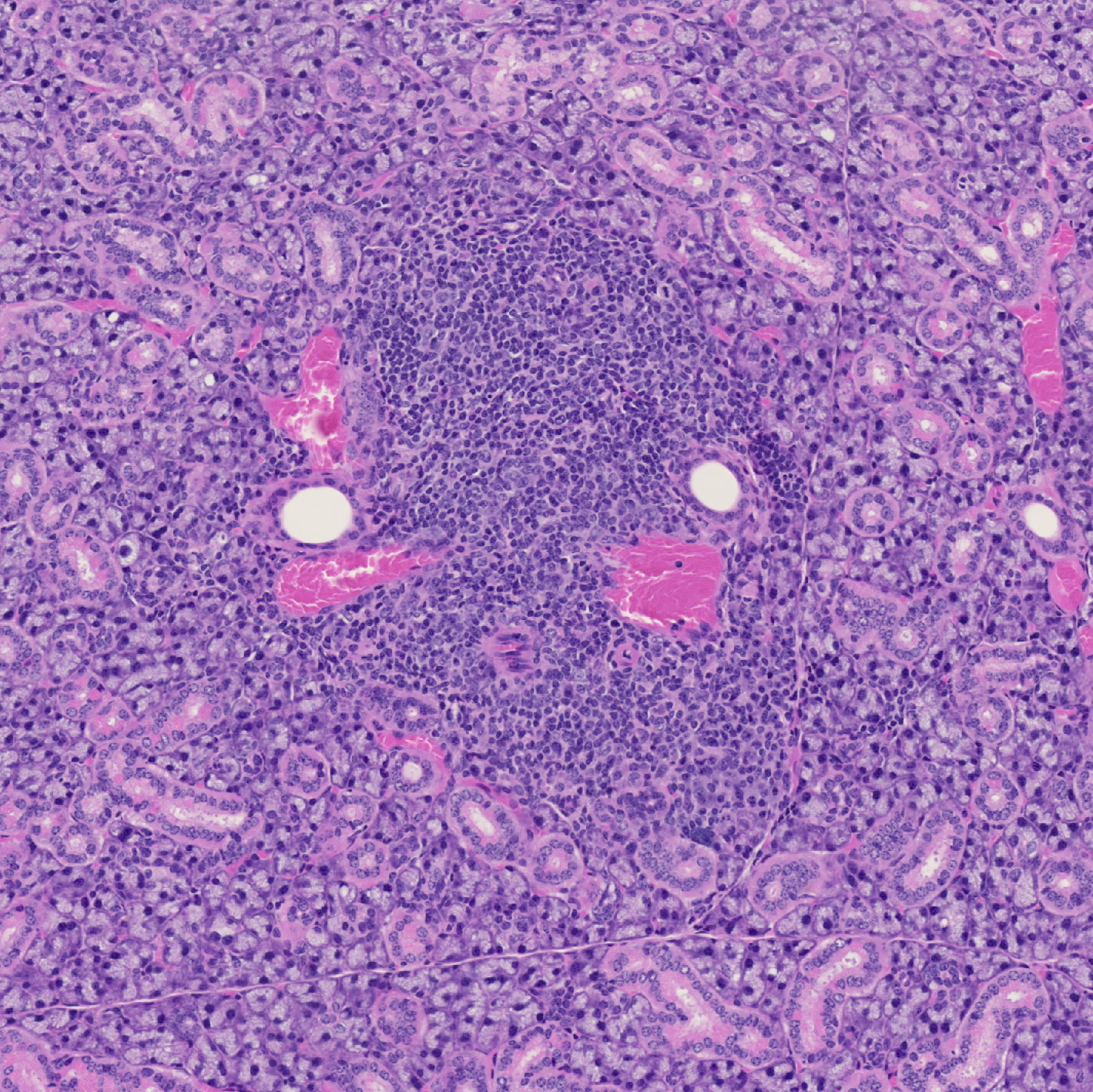

Supplement: Supplementary file 3 — Salivary gland images for Fig. 1j. [file 41590_2024_1902_MOESM3_ESM.zip › Fig 1j Heterozygous SG Vikala.tif]

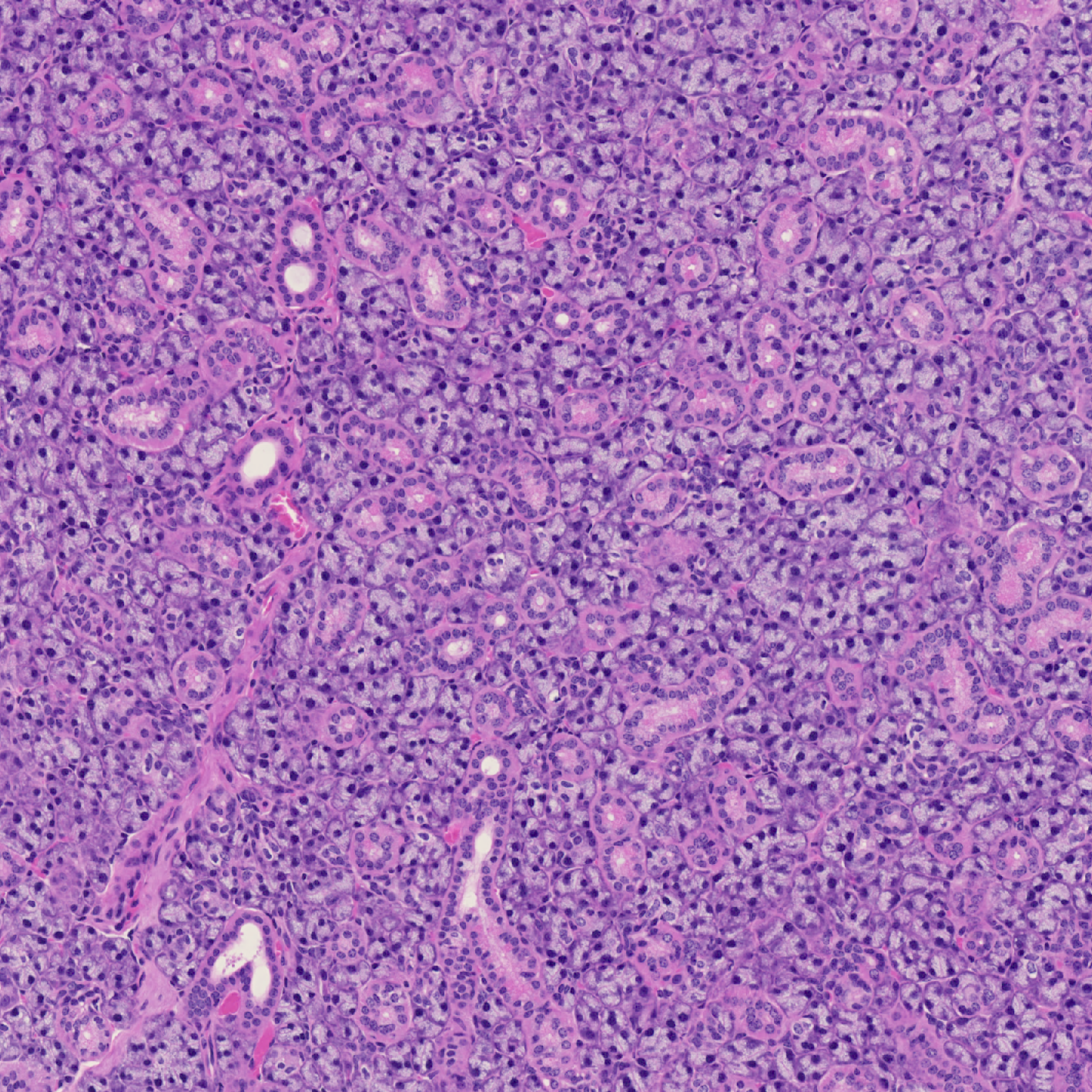

Supplement: Supplementary file 3 — Salivary gland images for Fig. 1j. [file 41590_2024_1902_MOESM3_ESM.zip › Fig 1j Wildtype SG.tif]

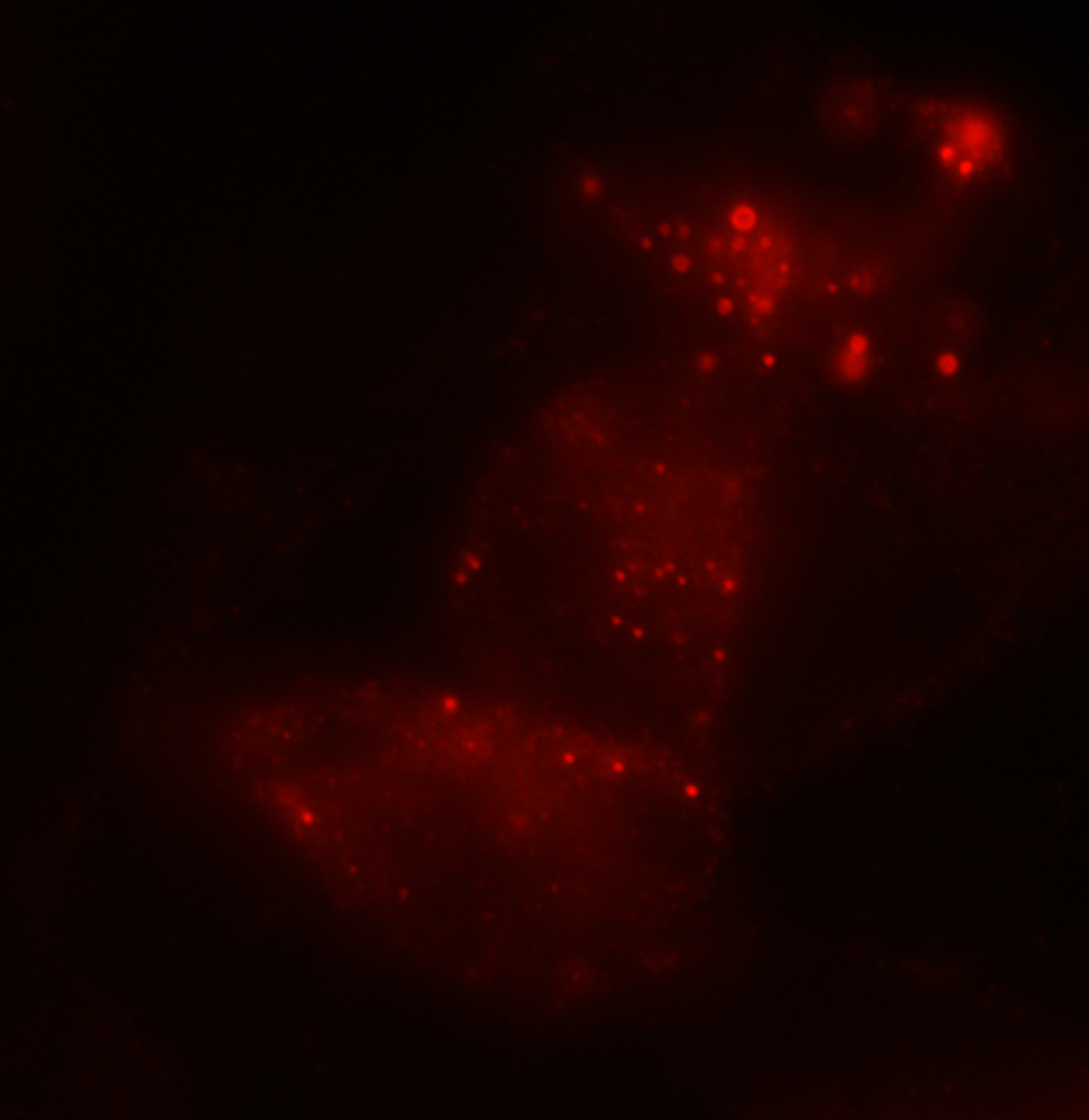

Supplement: Supplementary file 8 — Image files Fig. 5j. [file 41590_2024_1902_MOESM8_ESM.zip › Fig 5j Q333PhaTNIP-tnip.tif]

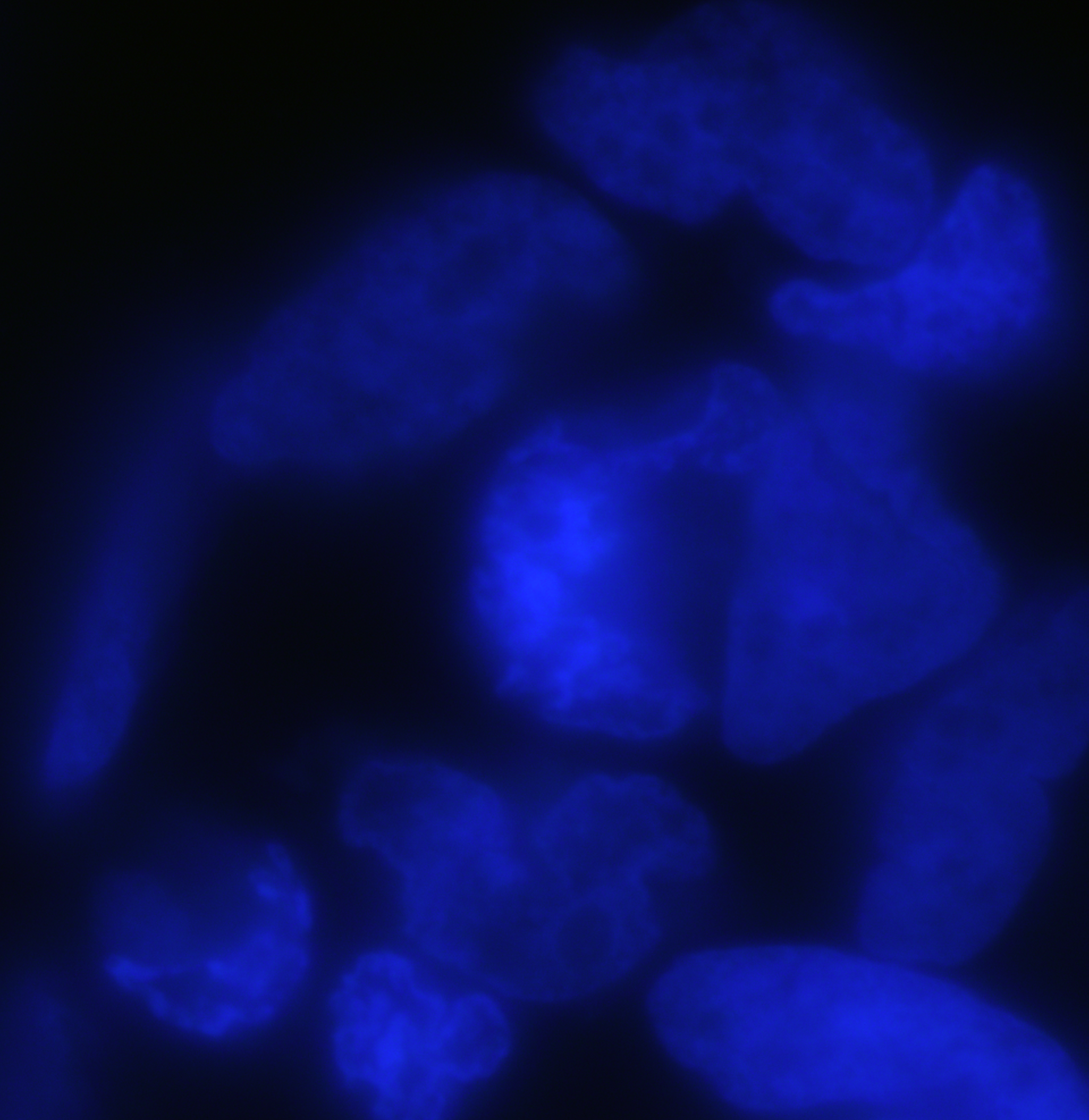

Supplement: Supplementary file 8 — Image files Fig. 5j. [file 41590_2024_1902_MOESM8_ESM.zip › Fig 5j Q333PhaTNIP-dna.tif]

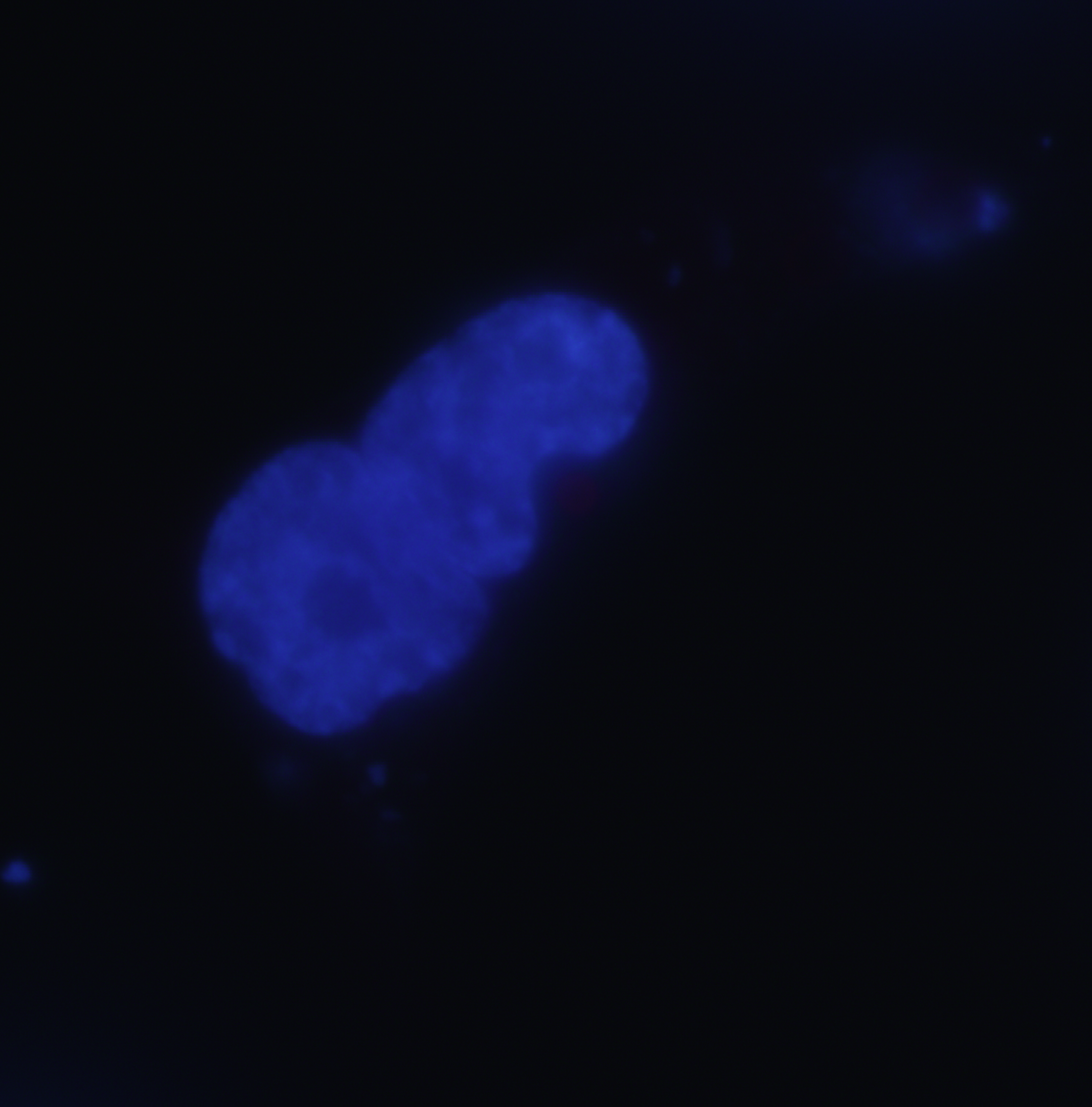

Supplement: Supplementary file 8 — Image files Fig. 5j. [file 41590_2024_1902_MOESM8_ESM.zip › Fig 5j D472NTNIPha-dna.tif]

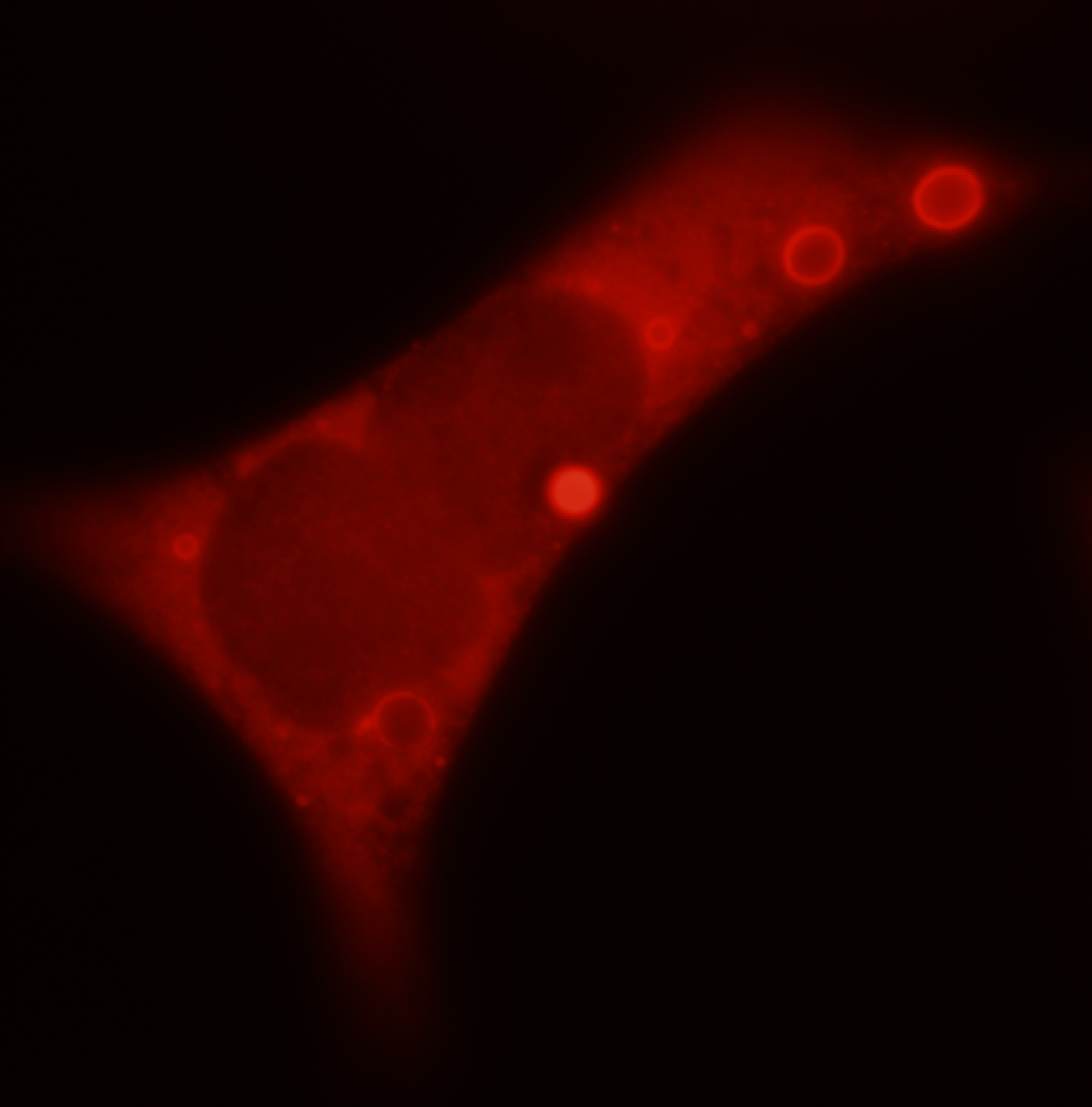

Supplement: Supplementary file 8 — Image files Fig. 5j. [file 41590_2024_1902_MOESM8_ESM.zip › Fig 5j D472NTNIPha-tnip.tif]

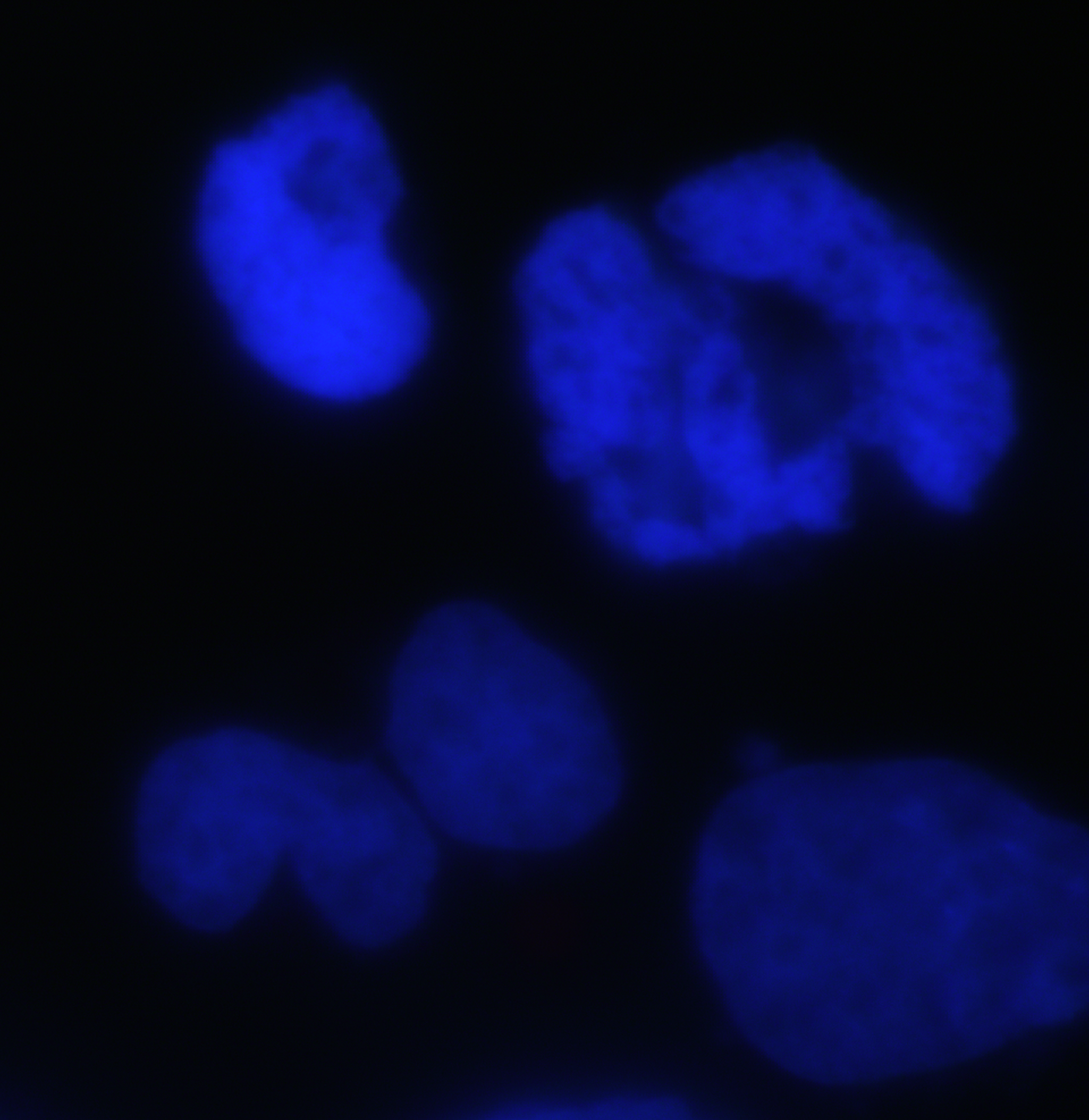

Supplement: Supplementary file 8 — Image files Fig. 5j. [file 41590_2024_1902_MOESM8_ESM.zip › Fig 5j wthaTNIP-dna.tif]

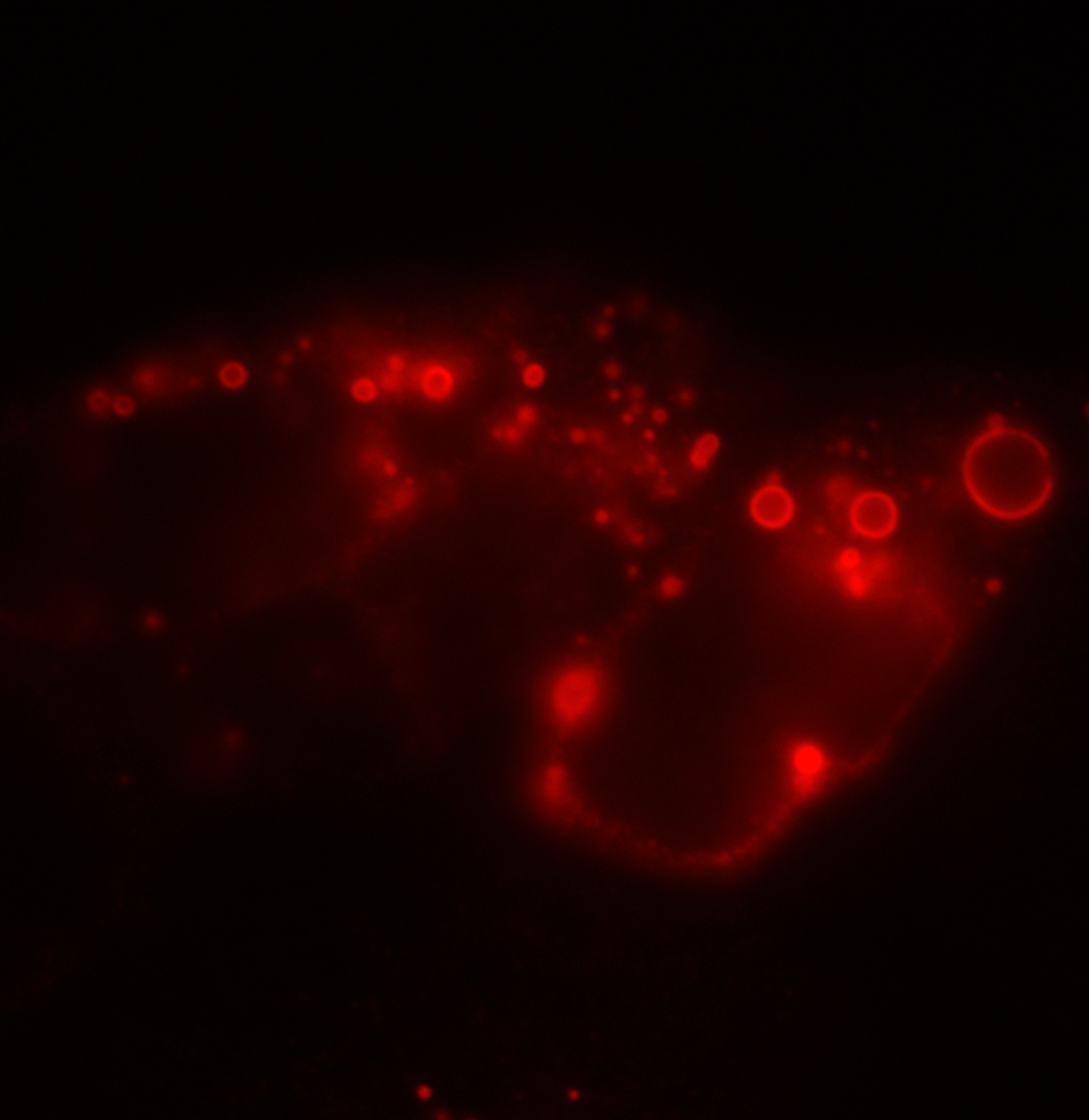

Supplement: Supplementary file 8 — Image files Fig. 5j. [file 41590_2024_1902_MOESM8_ESM.zip › Fig 5j wthaTNIP-tnip.tif]

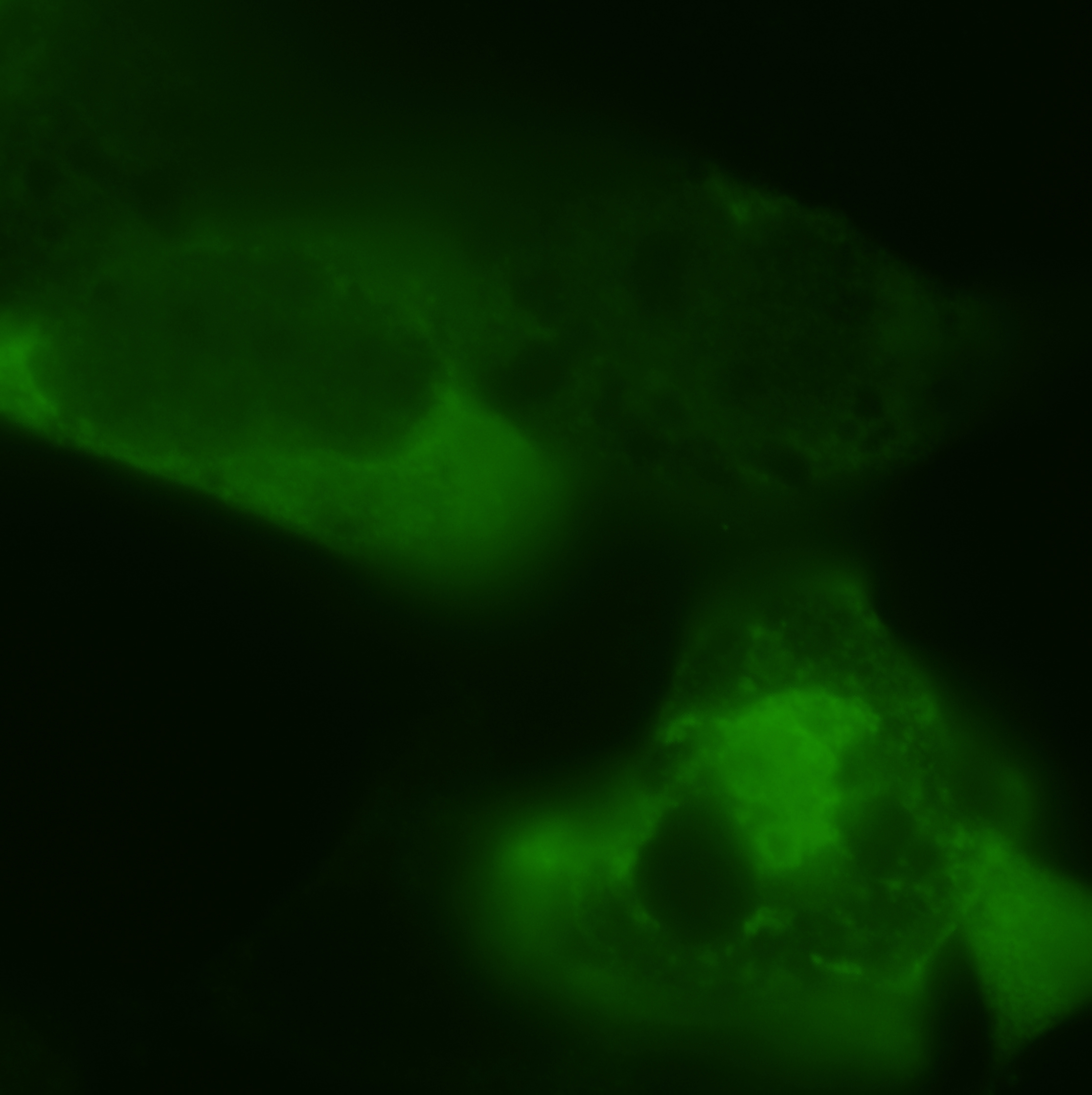

Supplement: Supplementary file 12 — Image files for Fig. 6a,b,d. [file 41590_2024_1902_MOESM12_ESM.zip › Fig 6a Q333PTNIP+Atg7-atg7.jpg]

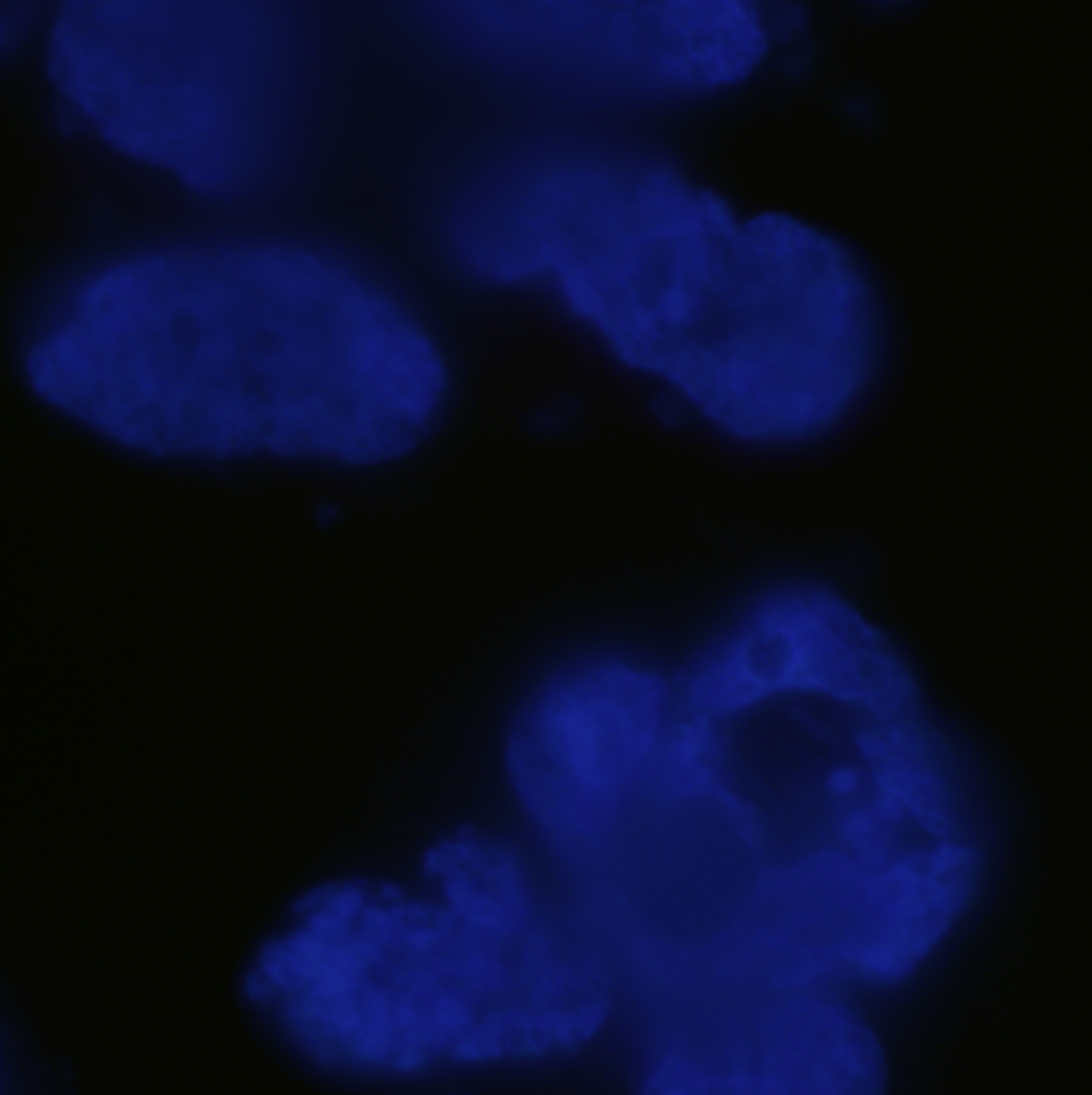

Supplement: Supplementary file 12 — Image files for Fig. 6a,b,d. [file 41590_2024_1902_MOESM12_ESM.zip › Fig 6a Q333PTNIP+Atg7-dna.jpg]

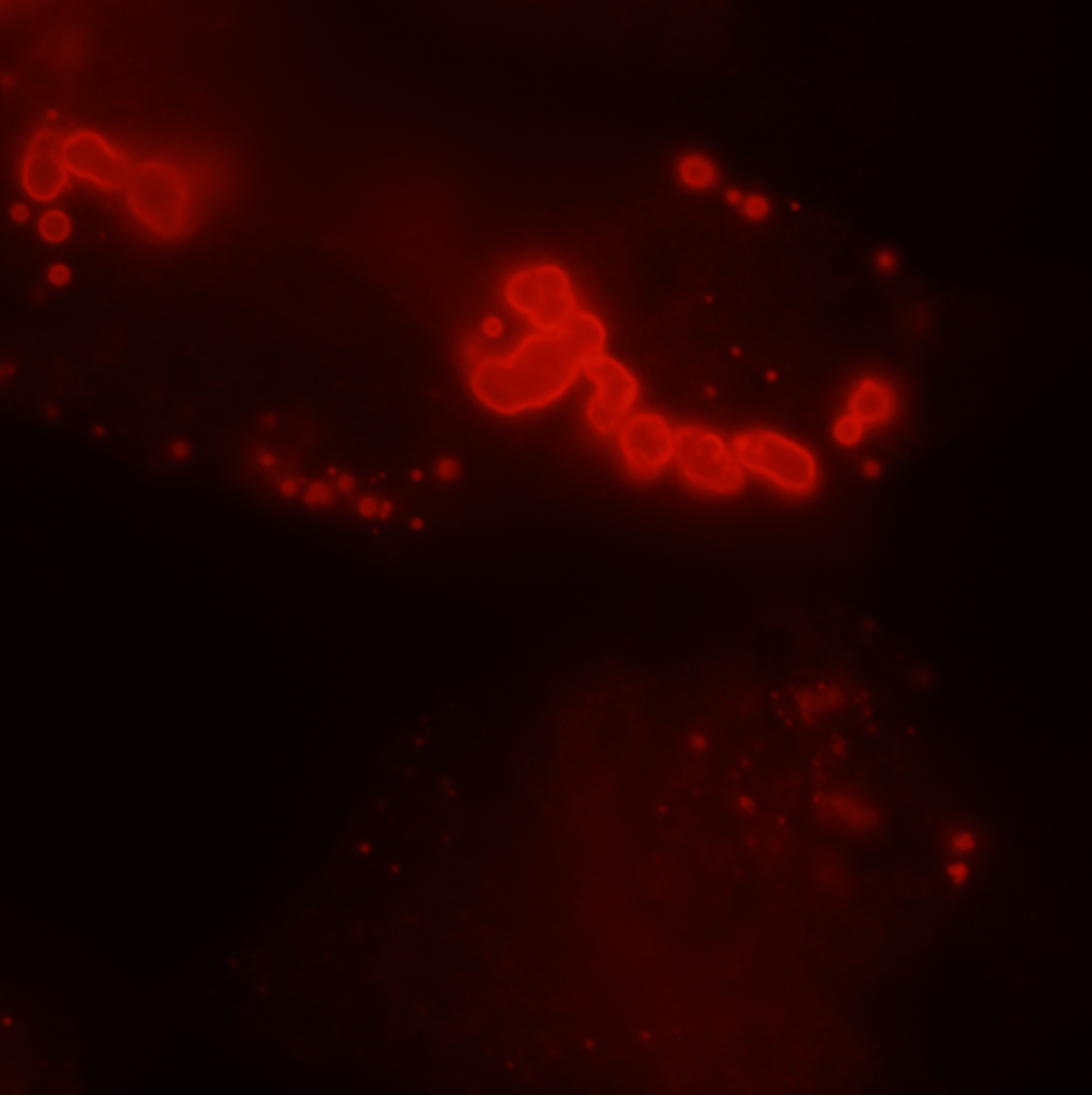

Supplement: Supplementary file 12 — Image files for Fig. 6a,b,d. [file 41590_2024_1902_MOESM12_ESM.zip › Fig 6a Q333PTNIP+Atg7-tnip.jpg]

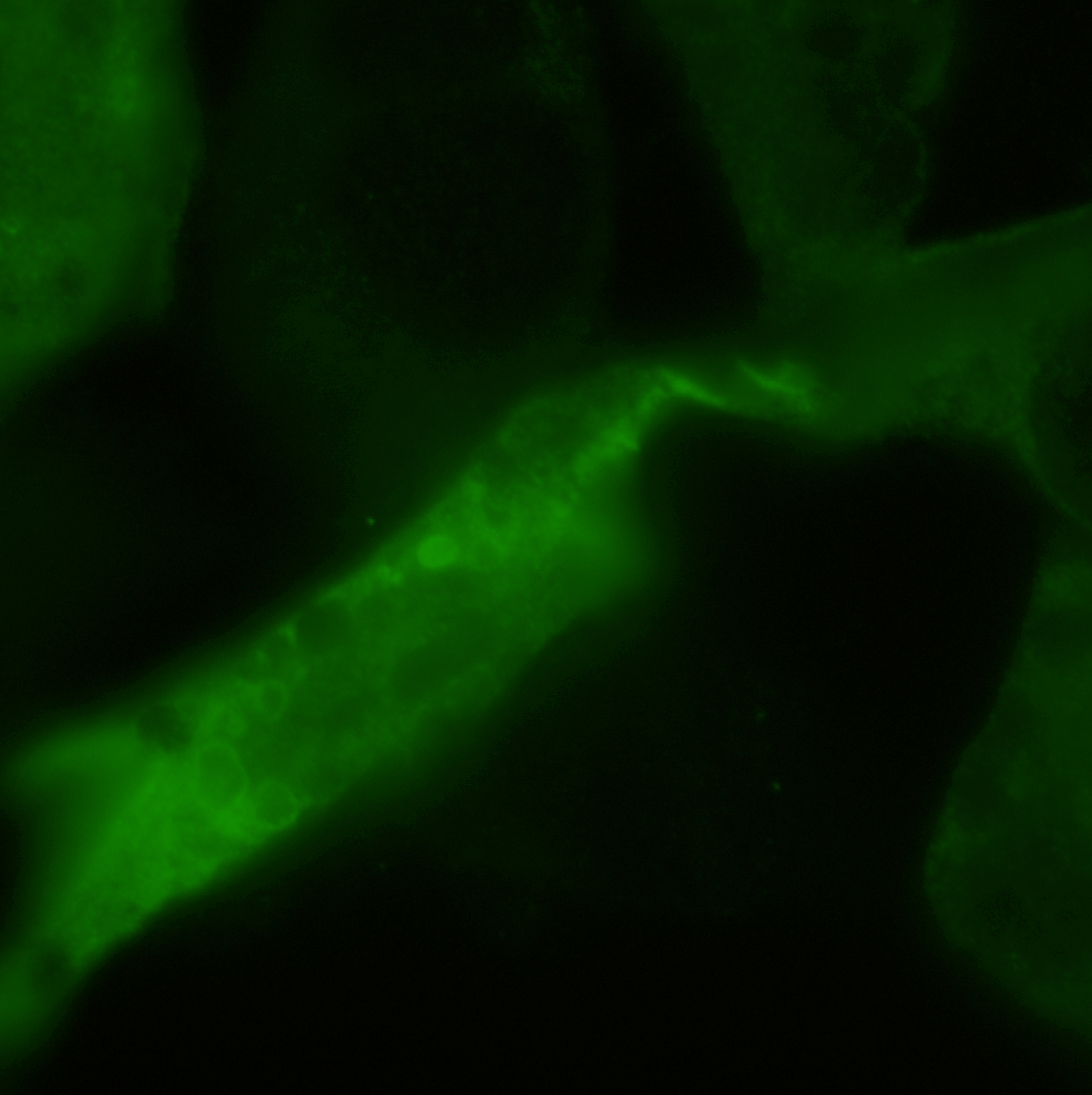

Supplement: Supplementary file 12 — Image files for Fig. 6a,b,d. [file 41590_2024_1902_MOESM12_ESM.zip › Fig 6a wtTNIP+Atg7-atg7.jpg]

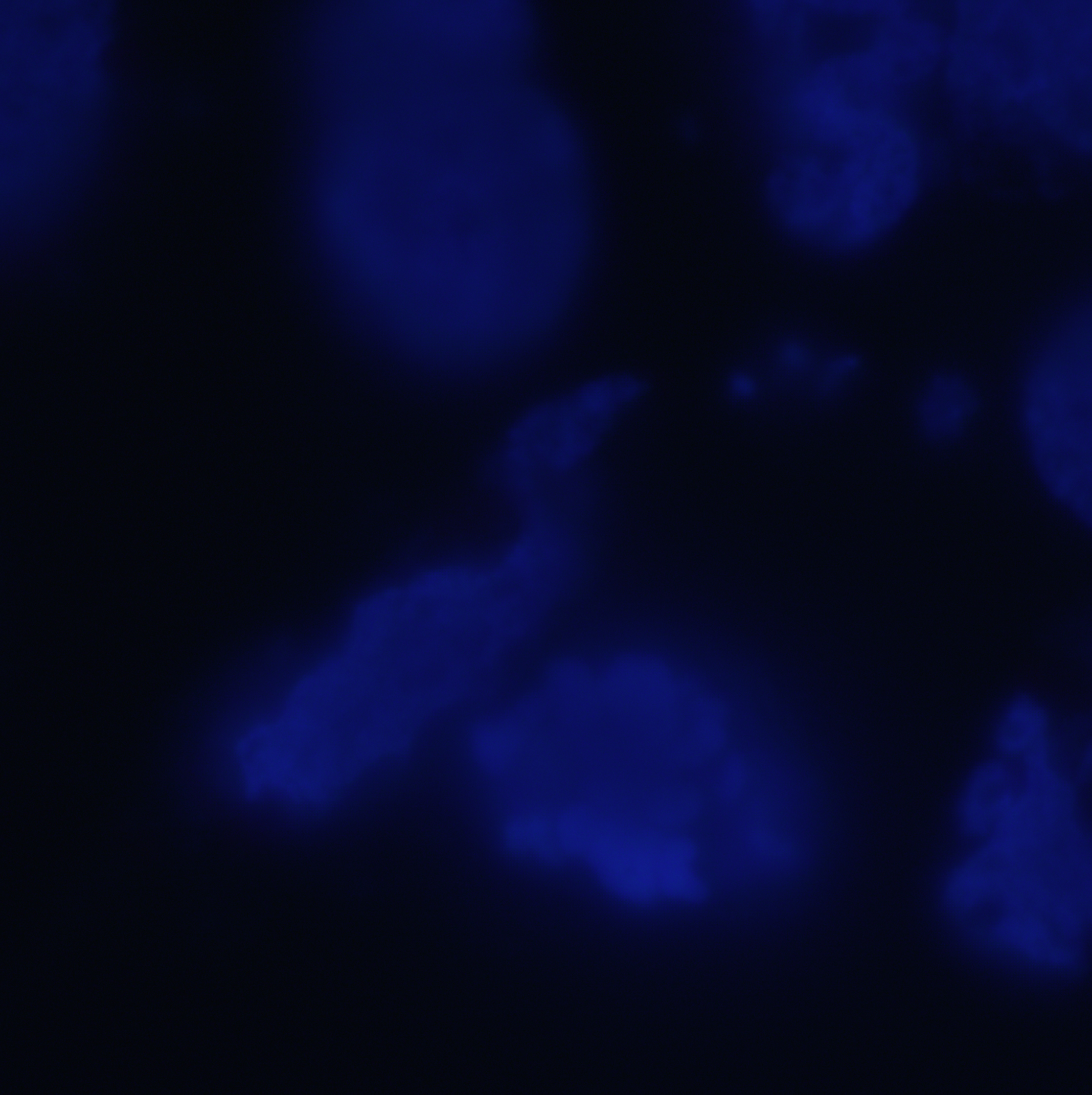

Supplement: Supplementary file 12 — Image files for Fig. 6a,b,d. [file 41590_2024_1902_MOESM12_ESM.zip › Fig 6a wtTNIP+Atg7-dna.jpg]

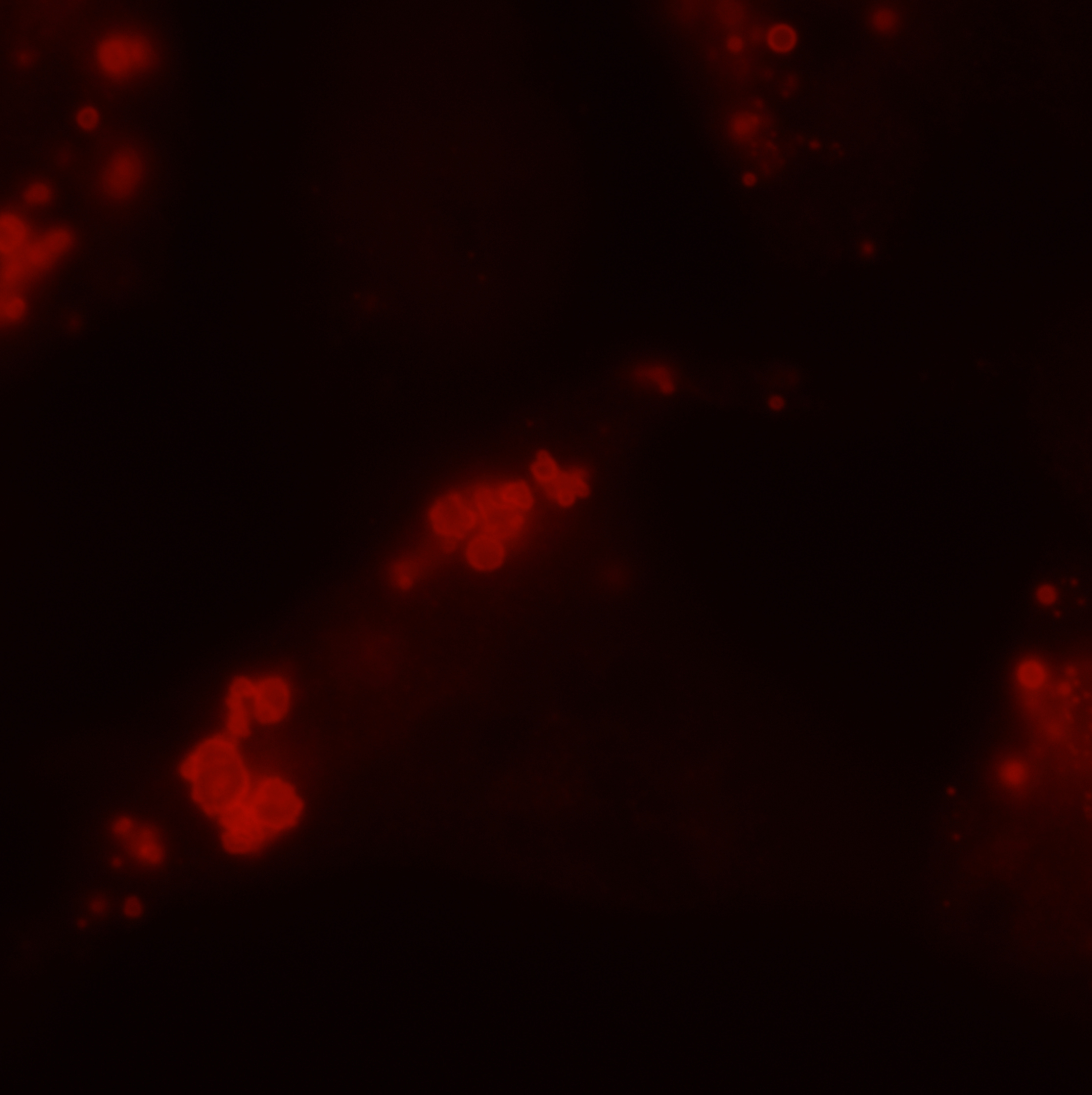

Supplement: Supplementary file 12 — Image files for Fig. 6a,b,d. [file 41590_2024_1902_MOESM12_ESM.zip › Fig 6a wtTNIP+Atg7-tnip.jpg]

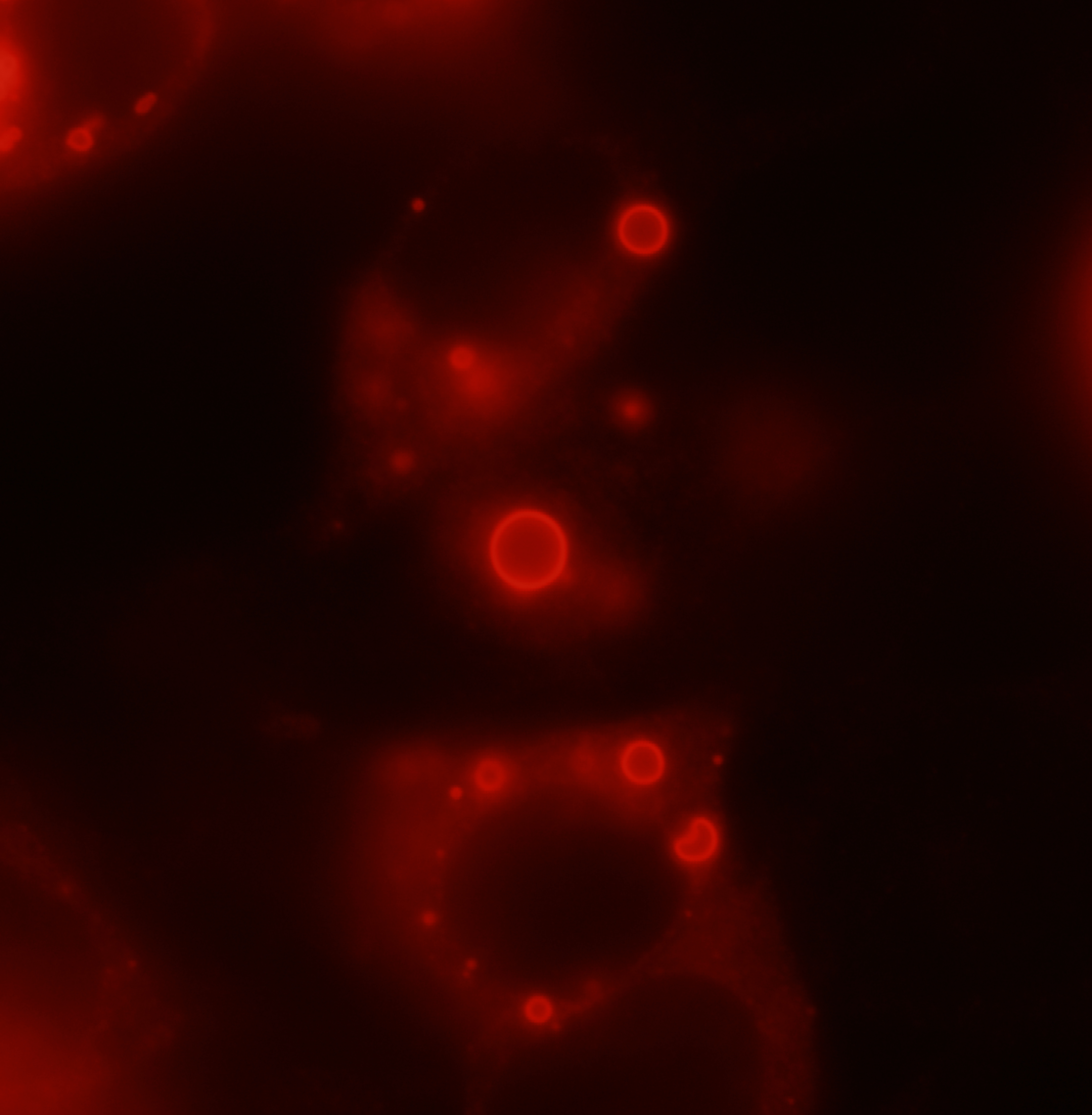

Supplement: Supplementary file 12 — Image files for Fig. 6a,b,d. [file 41590_2024_1902_MOESM12_ESM.zip › Fig 6b d472nTNIP+MyD88-tnip.jpg]

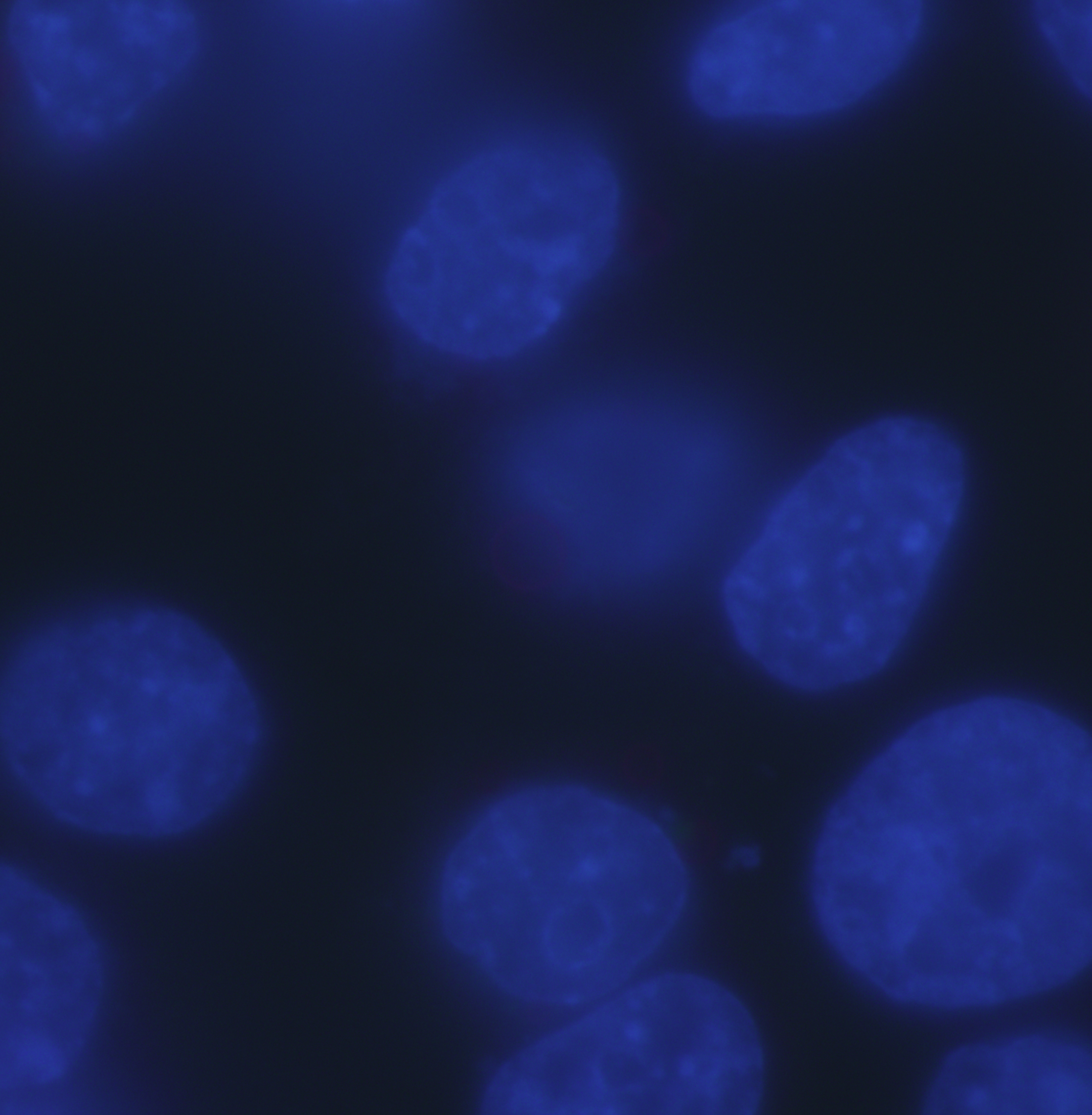

Supplement: Supplementary file 12 — Image files for Fig. 6a,b,d. [file 41590_2024_1902_MOESM12_ESM.zip › Fig 6b d472nTNIP+MyD88-dna.jpg]

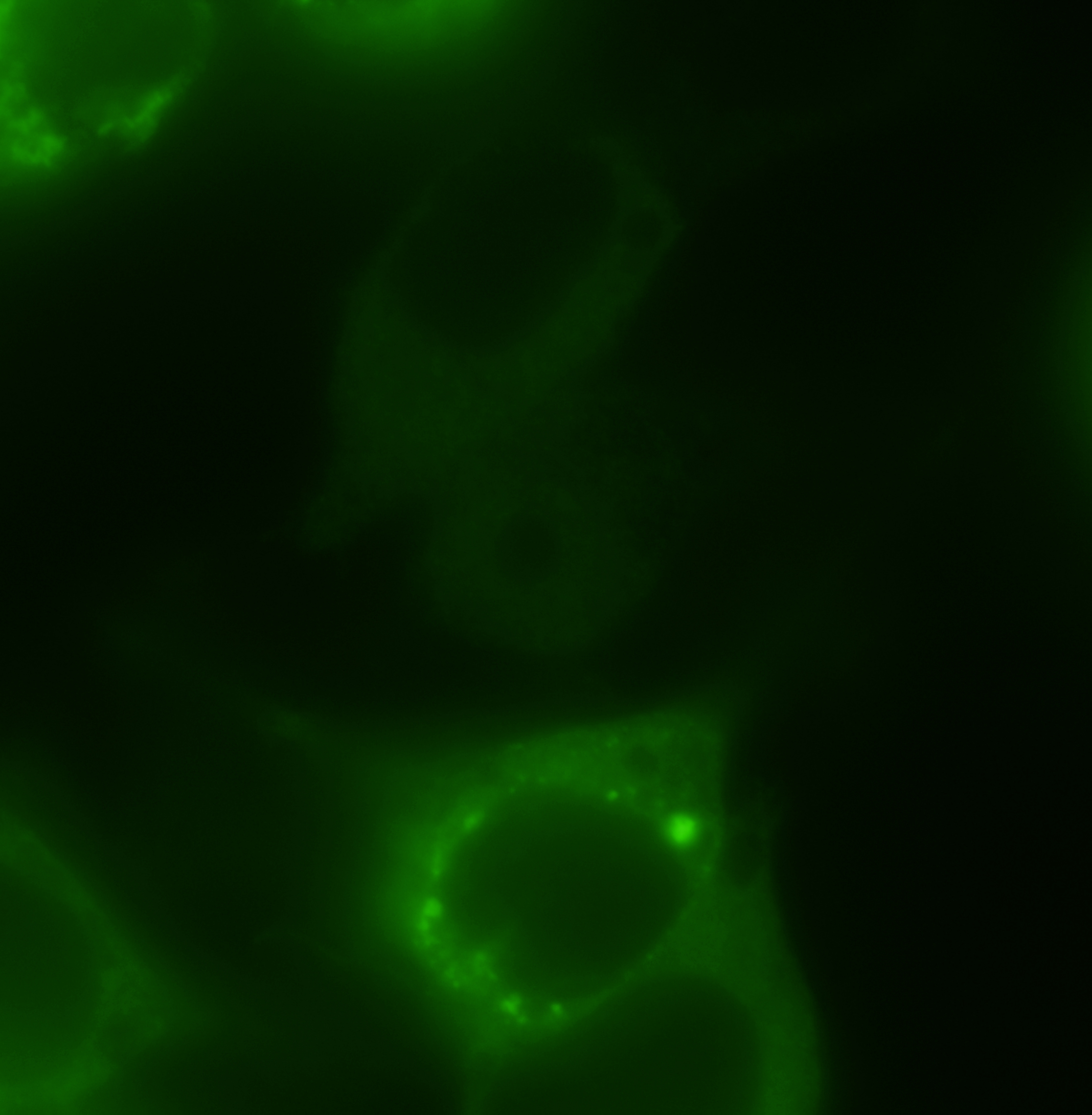

Supplement: Supplementary file 12 — Image files for Fig. 6a,b,d. [file 41590_2024_1902_MOESM12_ESM.zip › Fig 6b d472nTNIP+MyD88-myd88.jpg]

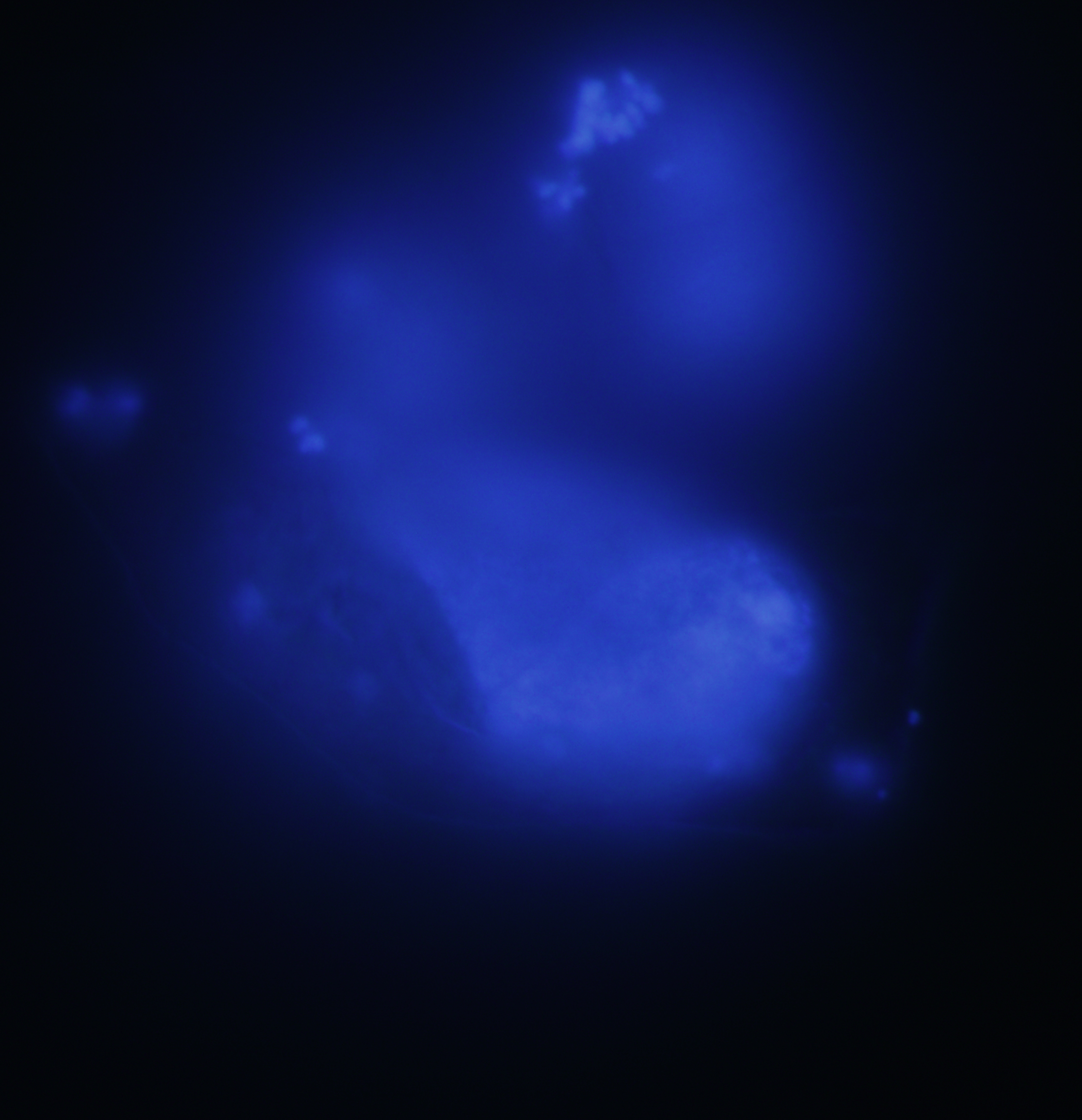

Supplement: Supplementary file 12 — Image files for Fig. 6a,b,d. [file 41590_2024_1902_MOESM12_ESM.zip › Fig 6b Q333PTNIPHA+MyD88-dna.jpg]

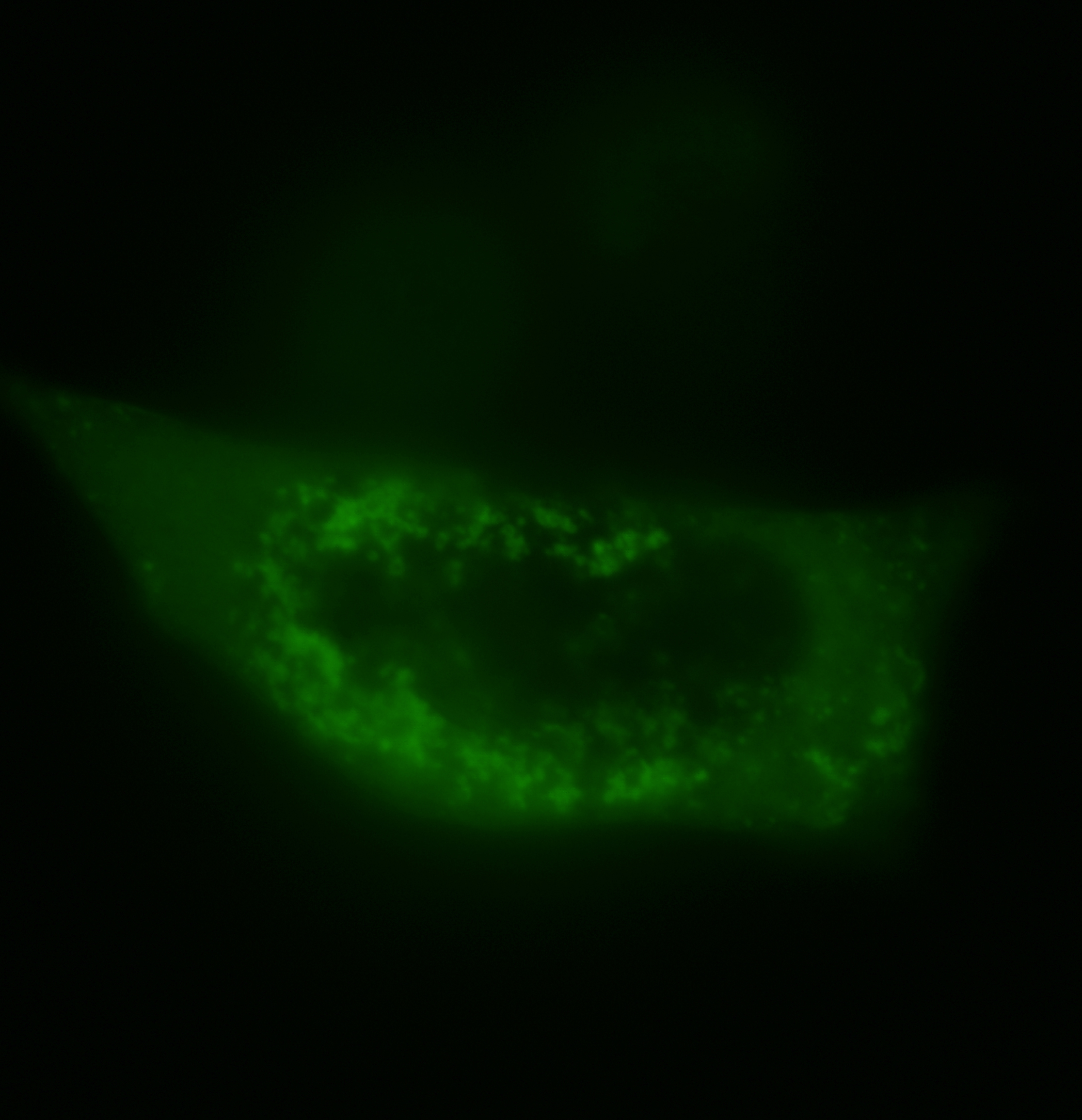

Supplement: Supplementary file 12 — Image files for Fig. 6a,b,d. [file 41590_2024_1902_MOESM12_ESM.zip › Fig 6b Q333PTNIPHA+MyD88-myd88.jpg]

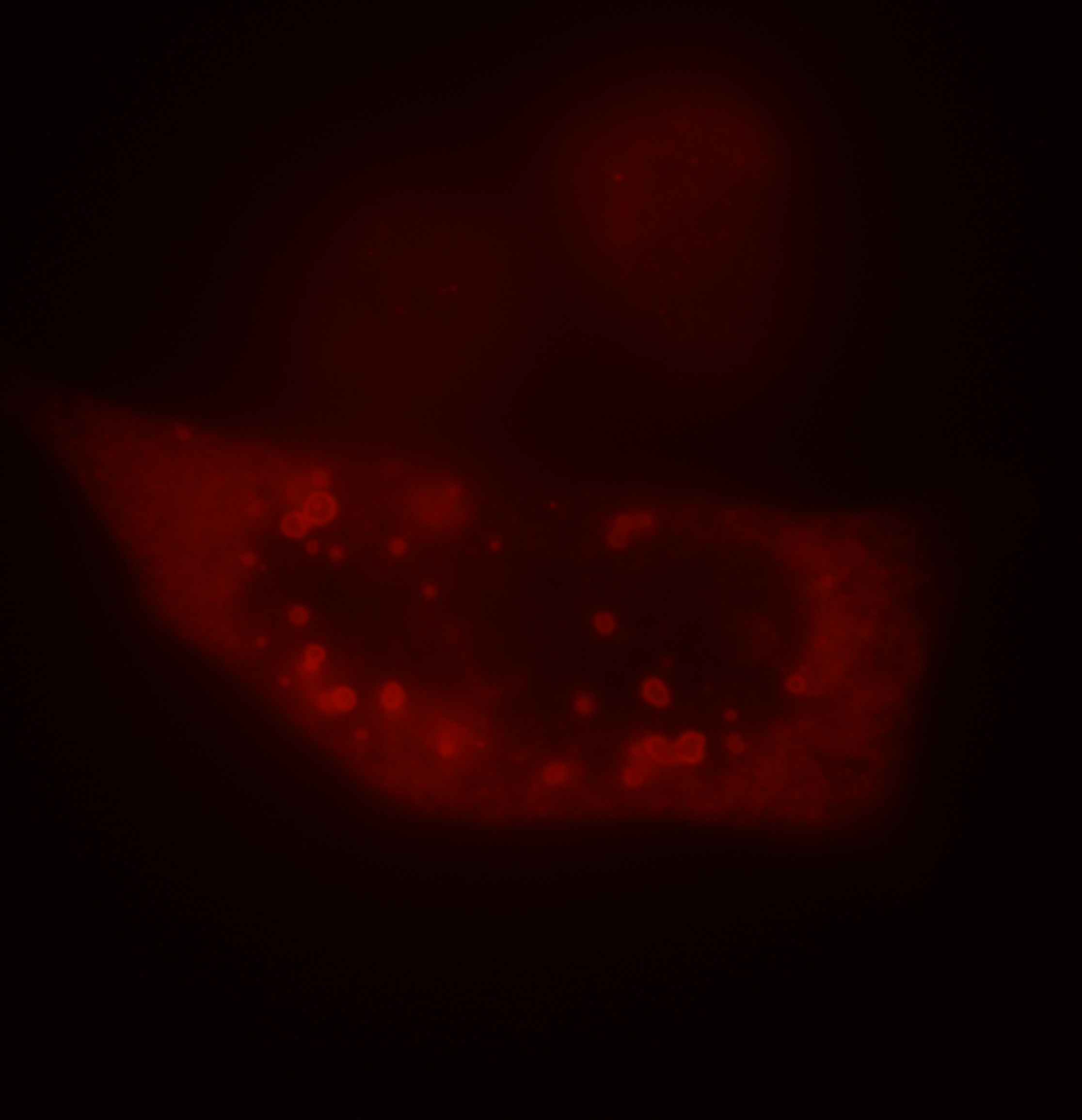

Supplement: Supplementary file 12 — Image files for Fig. 6a,b,d. [file 41590_2024_1902_MOESM12_ESM.zip › Fig 6b Q333PTNIPHA+MyD88-tnip.jpg]

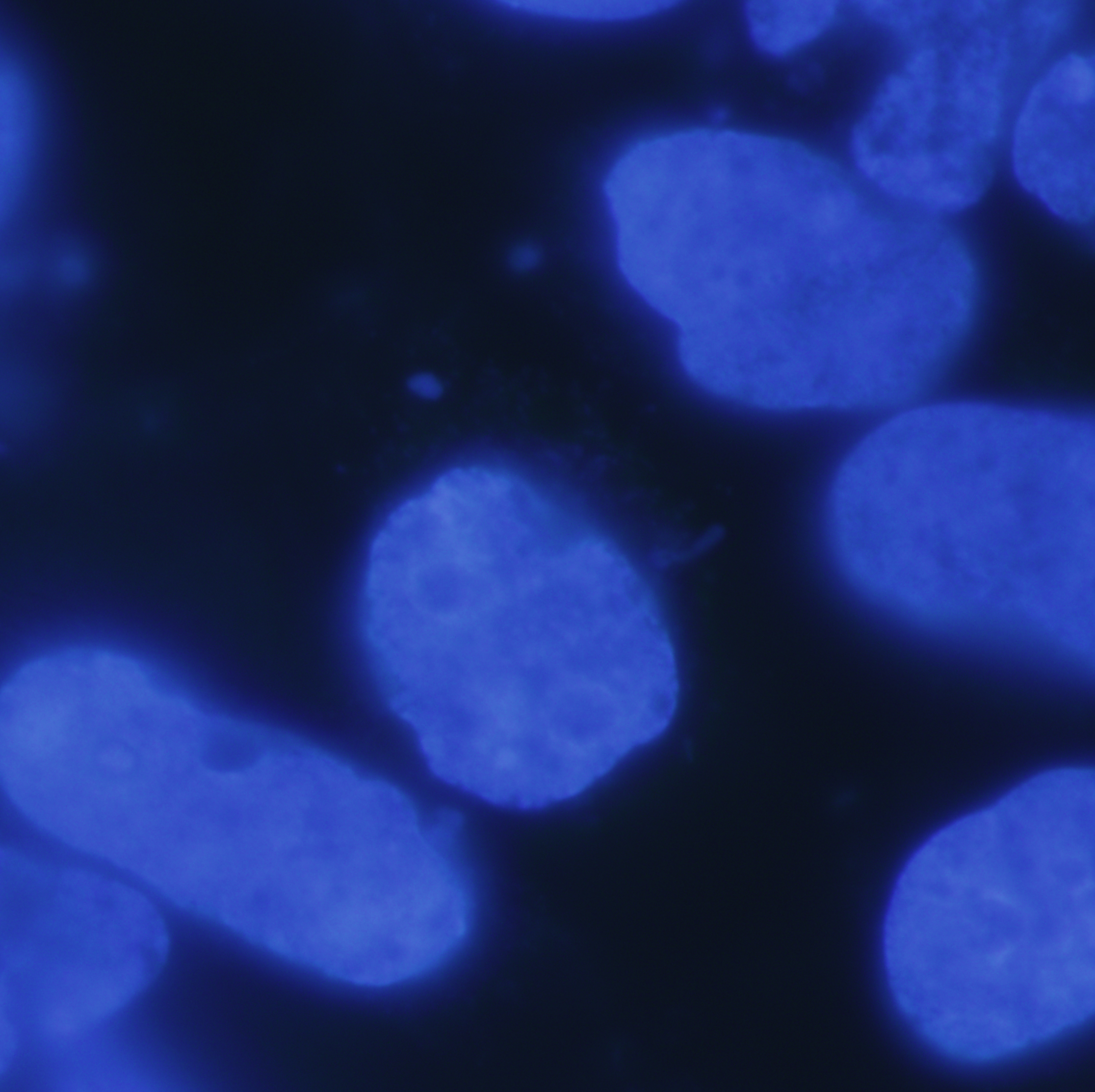

Supplement: Supplementary file 12 — Image files for Fig. 6a,b,d. [file 41590_2024_1902_MOESM12_ESM.zip › Fig 6b WTTNIP+MyD88-dna.jpg]

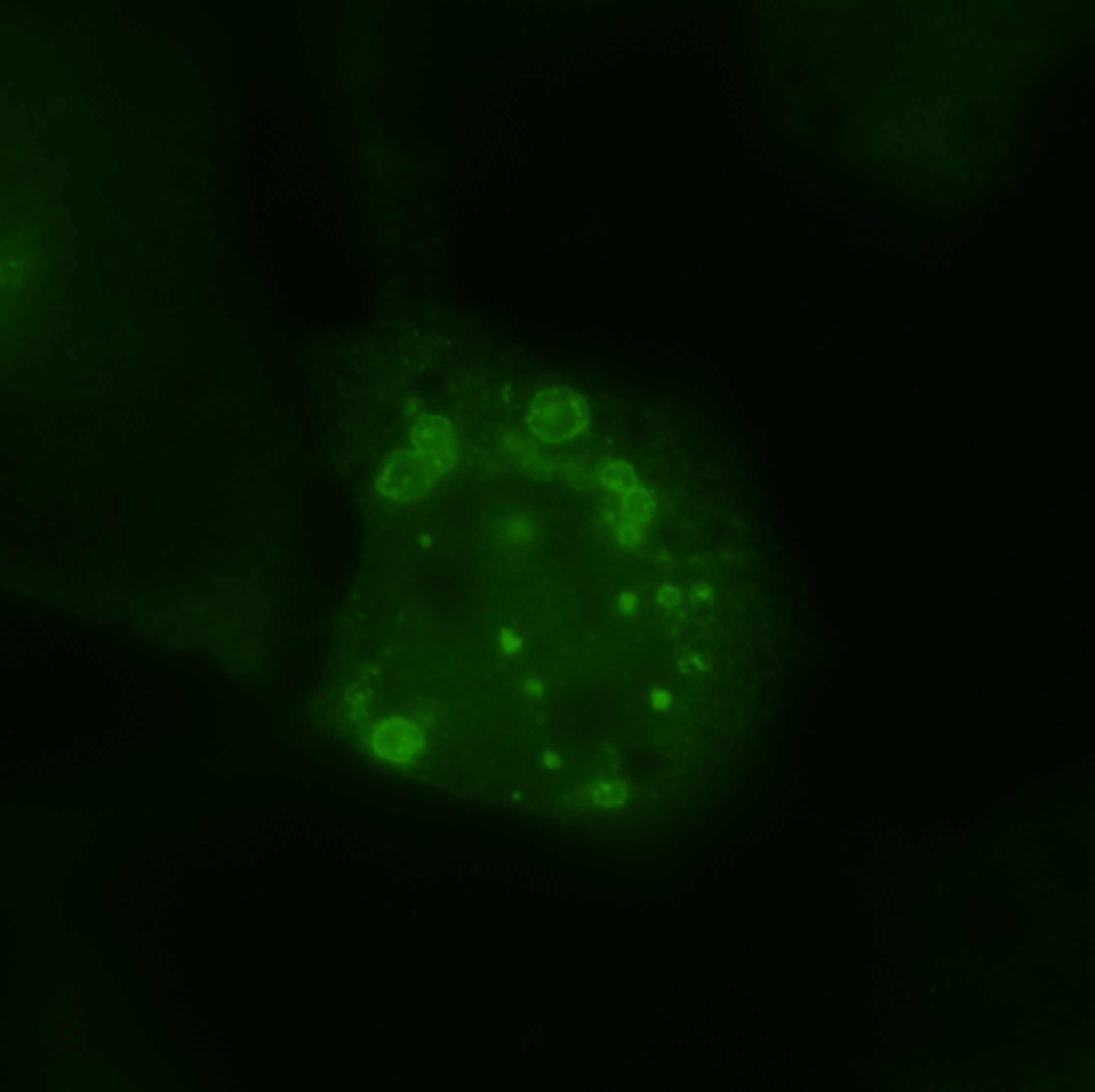

Supplement: Supplementary file 12 — Image files for Fig. 6a,b,d. [file 41590_2024_1902_MOESM12_ESM.zip › Fig 6b WTTNIP+MyD88-myd88.jpg]

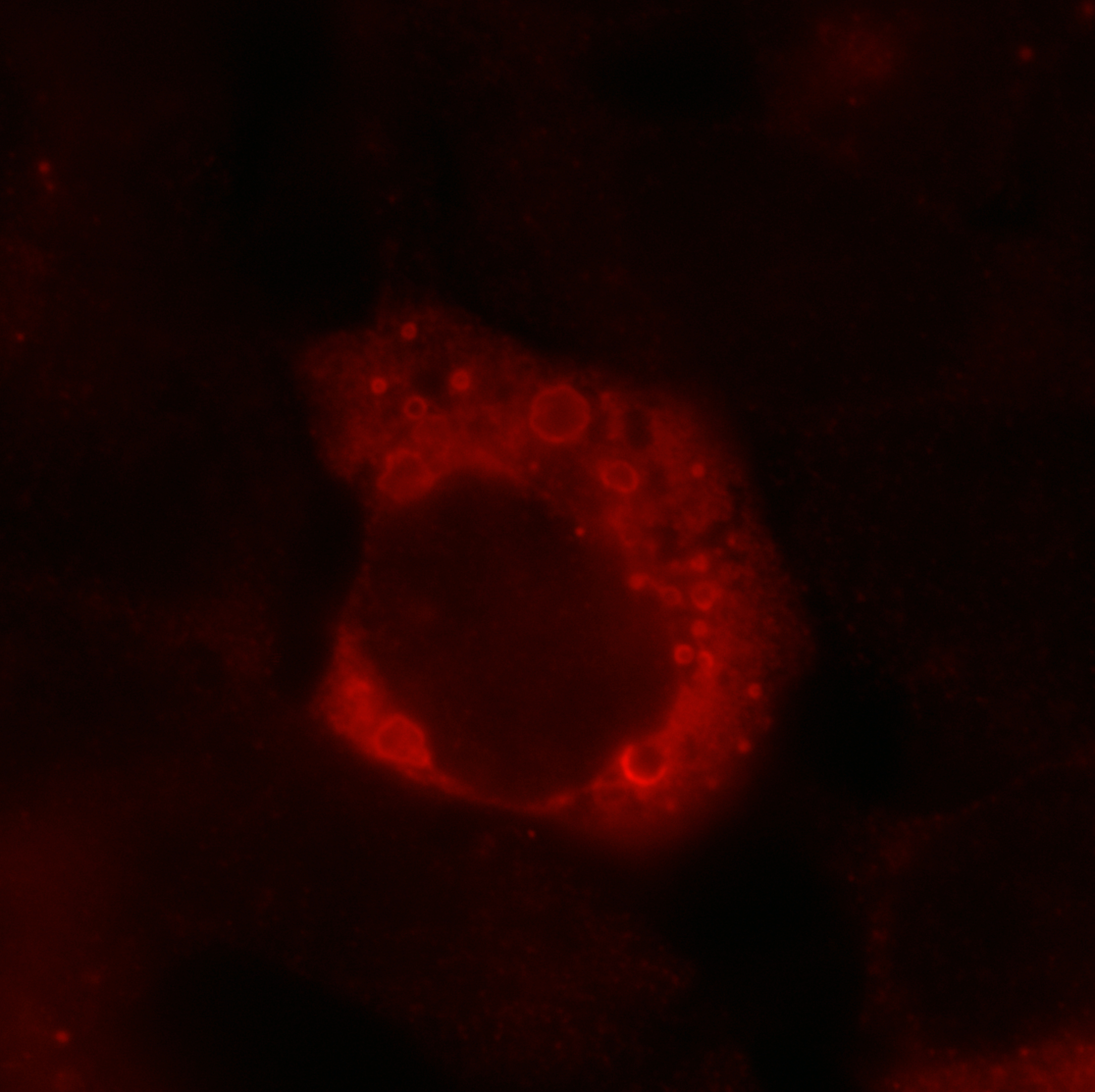

Supplement: Supplementary file 12 — Image files for Fig. 6a,b,d. [file 41590_2024_1902_MOESM12_ESM.zip › Fig 6b WTTNIP+MyD88-tnip.jpg]

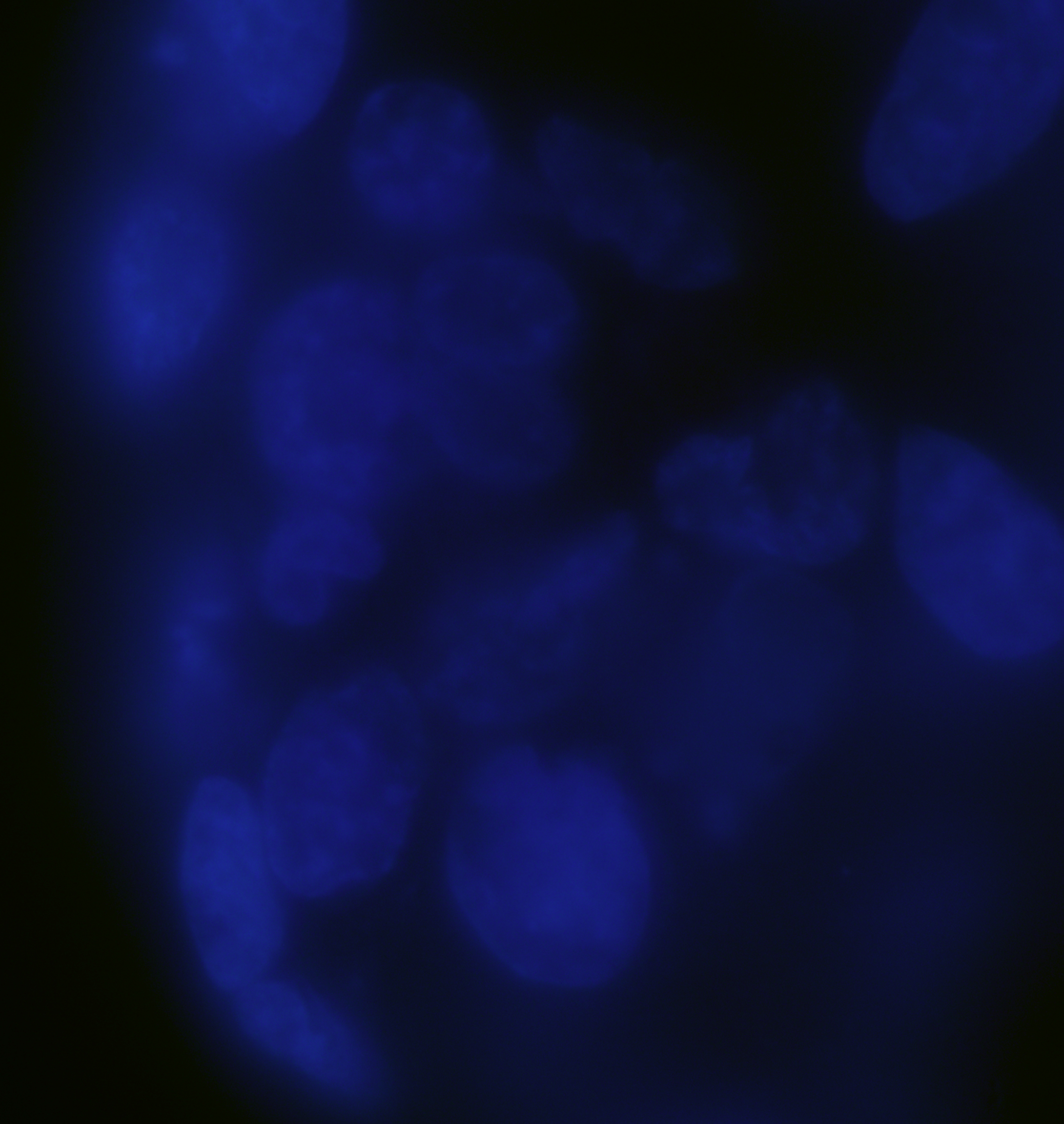

Supplement: Supplementary file 12 — Image files for Fig. 6a,b,d. [file 41590_2024_1902_MOESM12_ESM.zip › Fig 6d IRAK1+Q333PTNIP-dna.jpg]

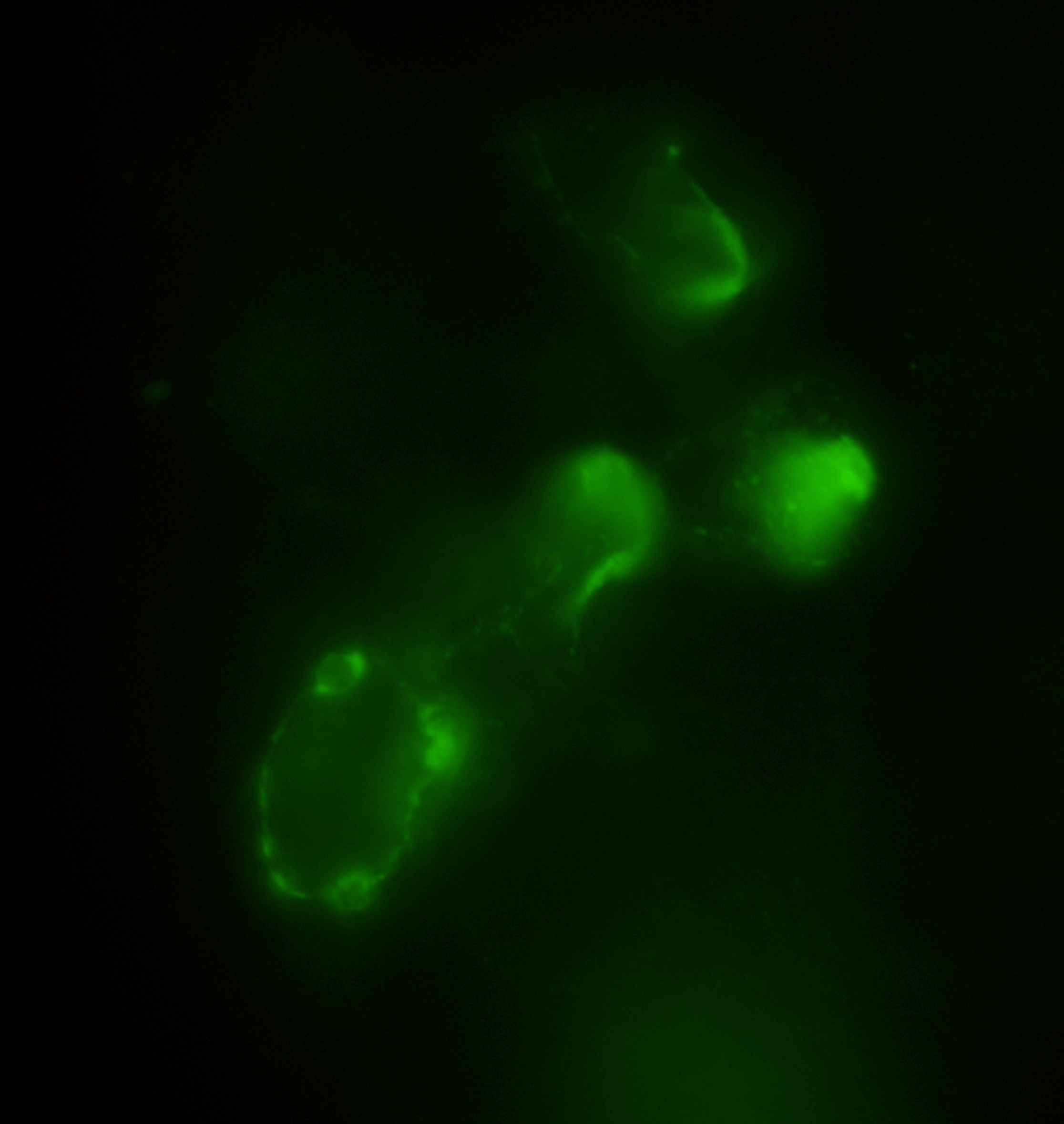

Supplement: Supplementary file 12 — Image files for Fig. 6a,b,d. [file 41590_2024_1902_MOESM12_ESM.zip › Fig 6d IRAK1+Q333PTNIP-IRAK.jpg]

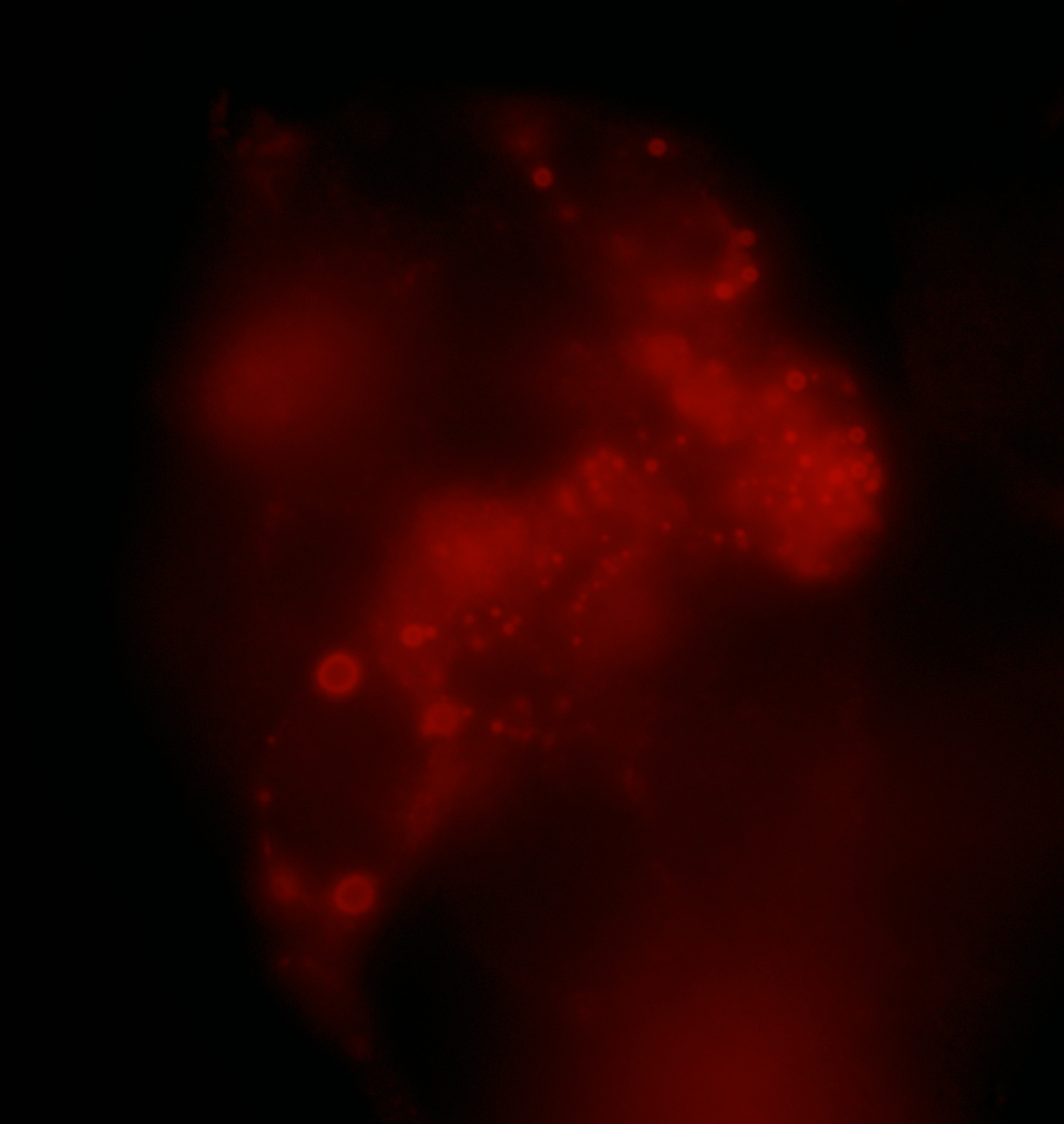

Supplement: Supplementary file 12 — Image files for Fig. 6a,b,d. [file 41590_2024_1902_MOESM12_ESM.zip › Fig 6d IRAK1+Q333PTNIP-tnip.jpg]

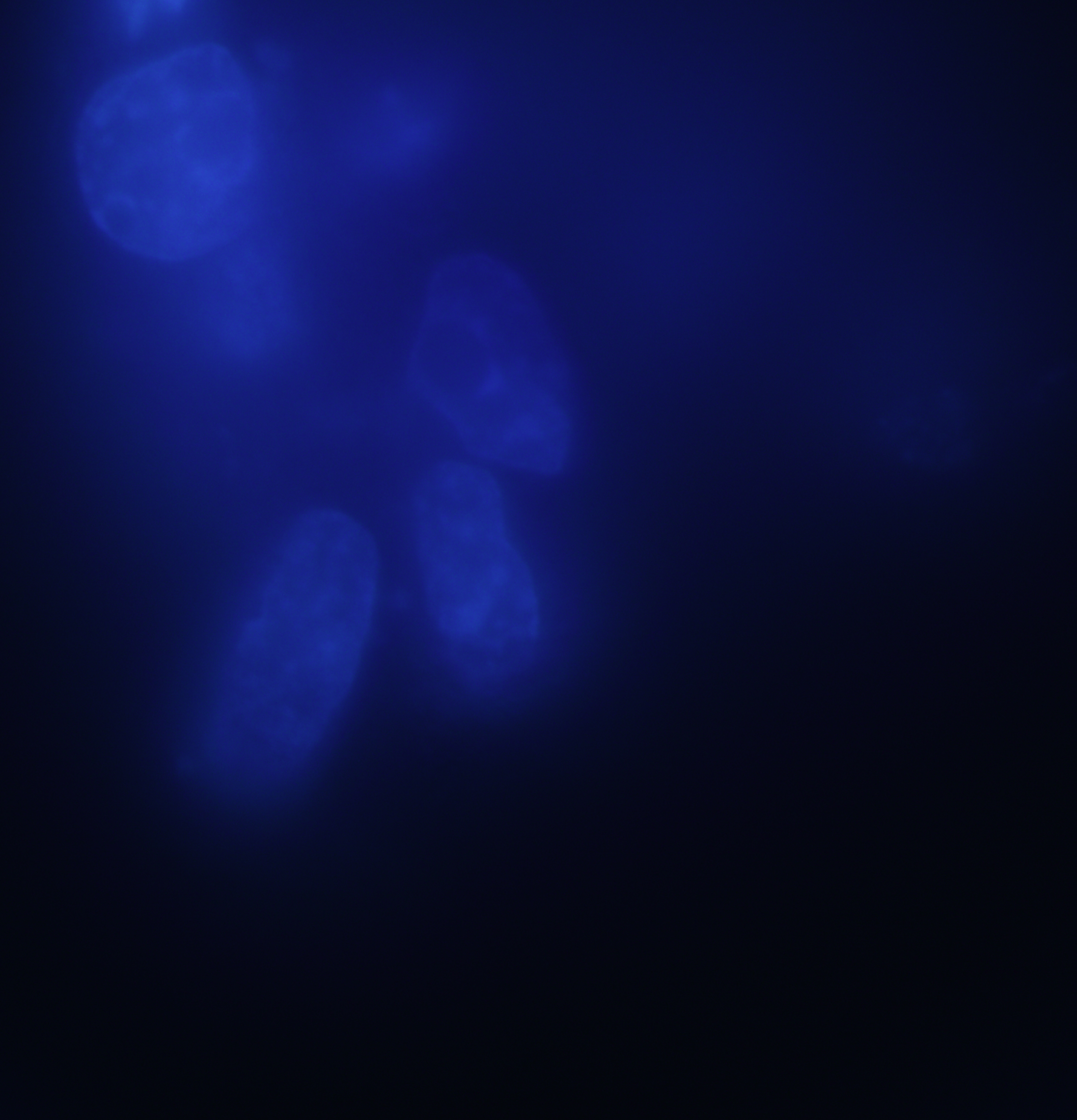

Supplement: Supplementary file 12 — Image files for Fig. 6a,b,d. [file 41590_2024_1902_MOESM12_ESM.zip › Fig 6d IRAK1+WTTNIP-dna.jpg]

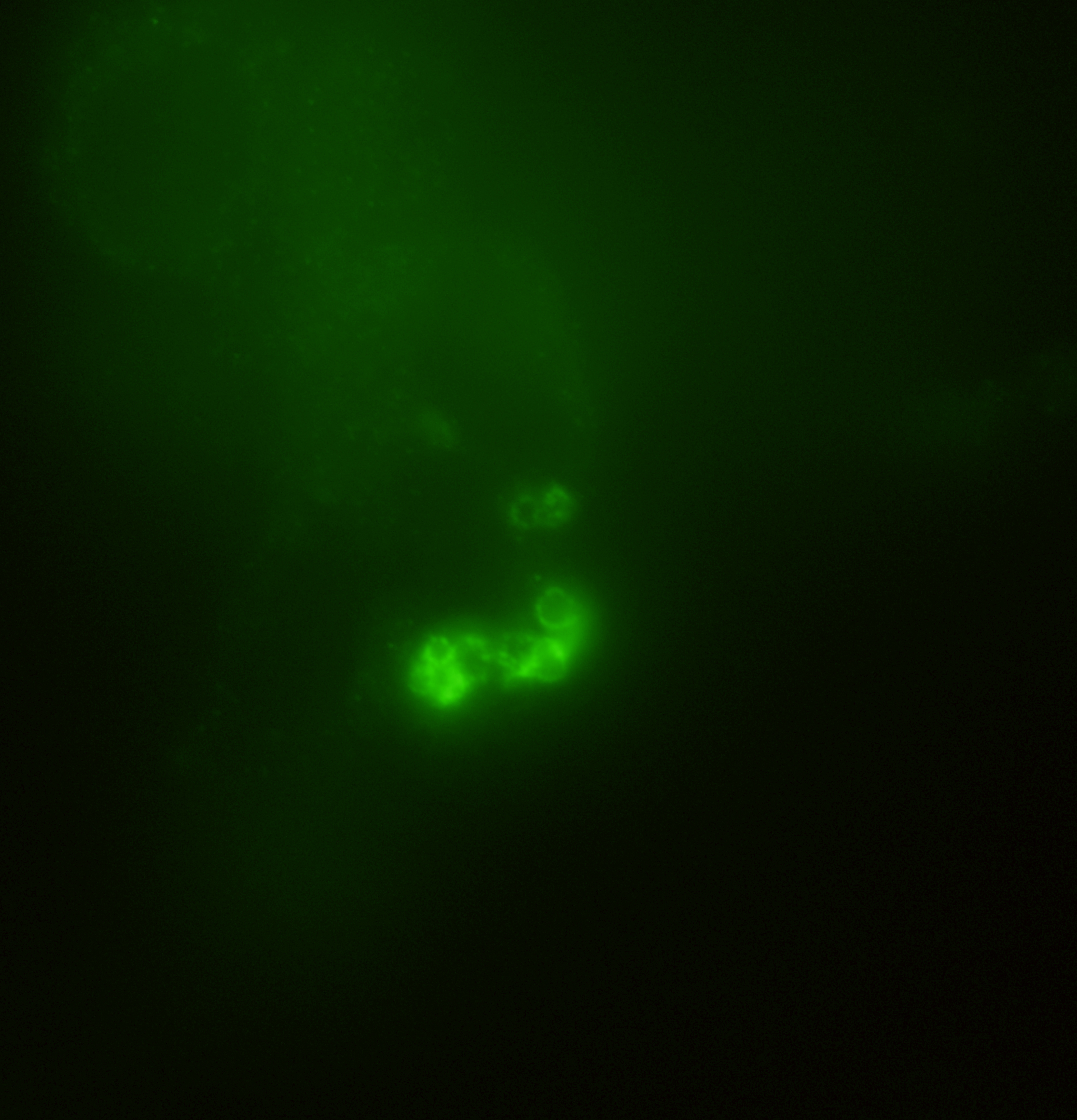

Supplement: Supplementary file 12 — Image files for Fig. 6a,b,d. [file 41590_2024_1902_MOESM12_ESM.zip › Fig 6d IRAK1+WTTNIP-IRAK.jpg]

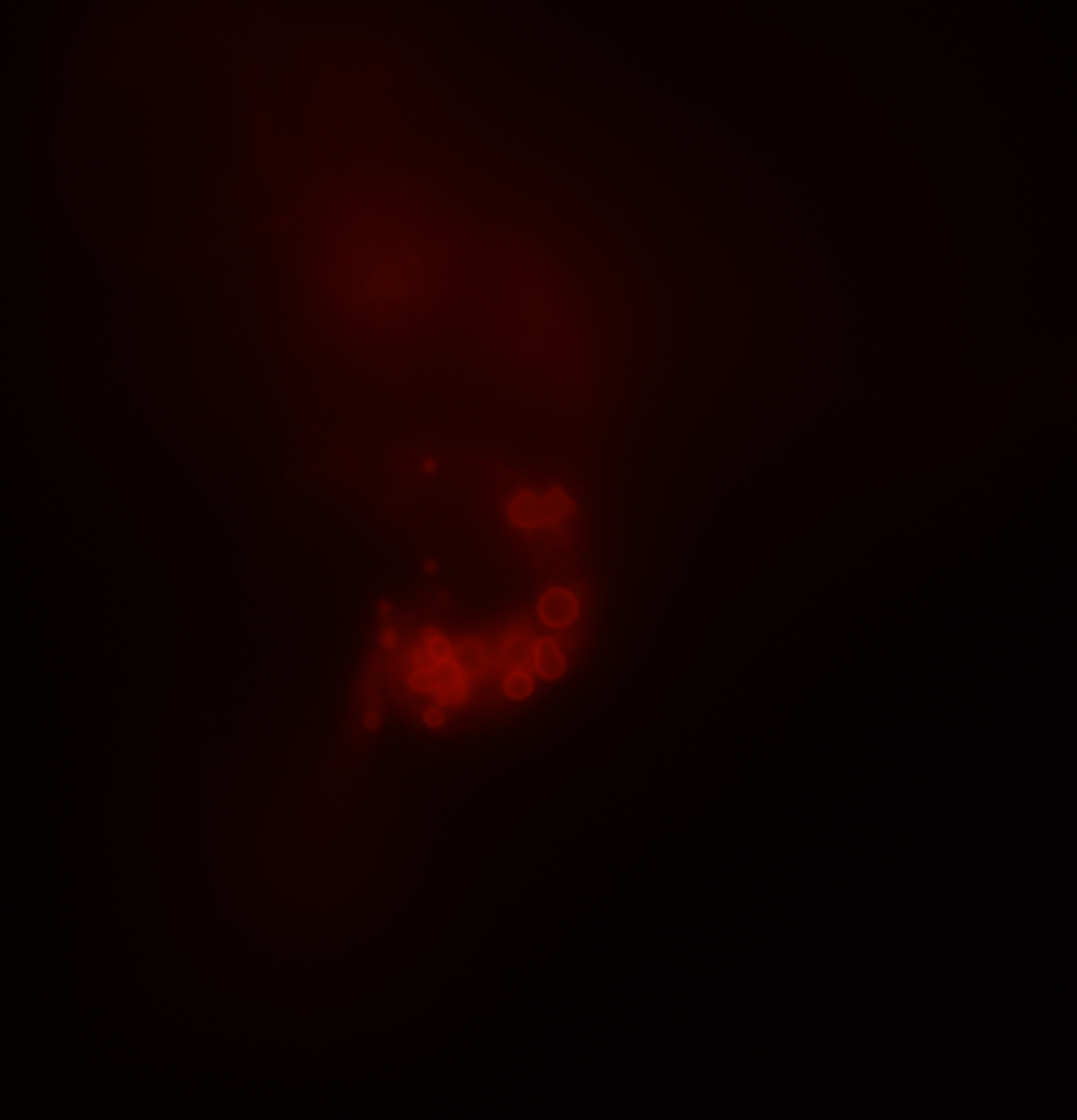

Supplement: Supplementary file 12 — Image files for Fig. 6a,b,d. [file 41590_2024_1902_MOESM12_ESM.zip › Fig 6d IRAK1+WTTNIP-tnip.jpg]

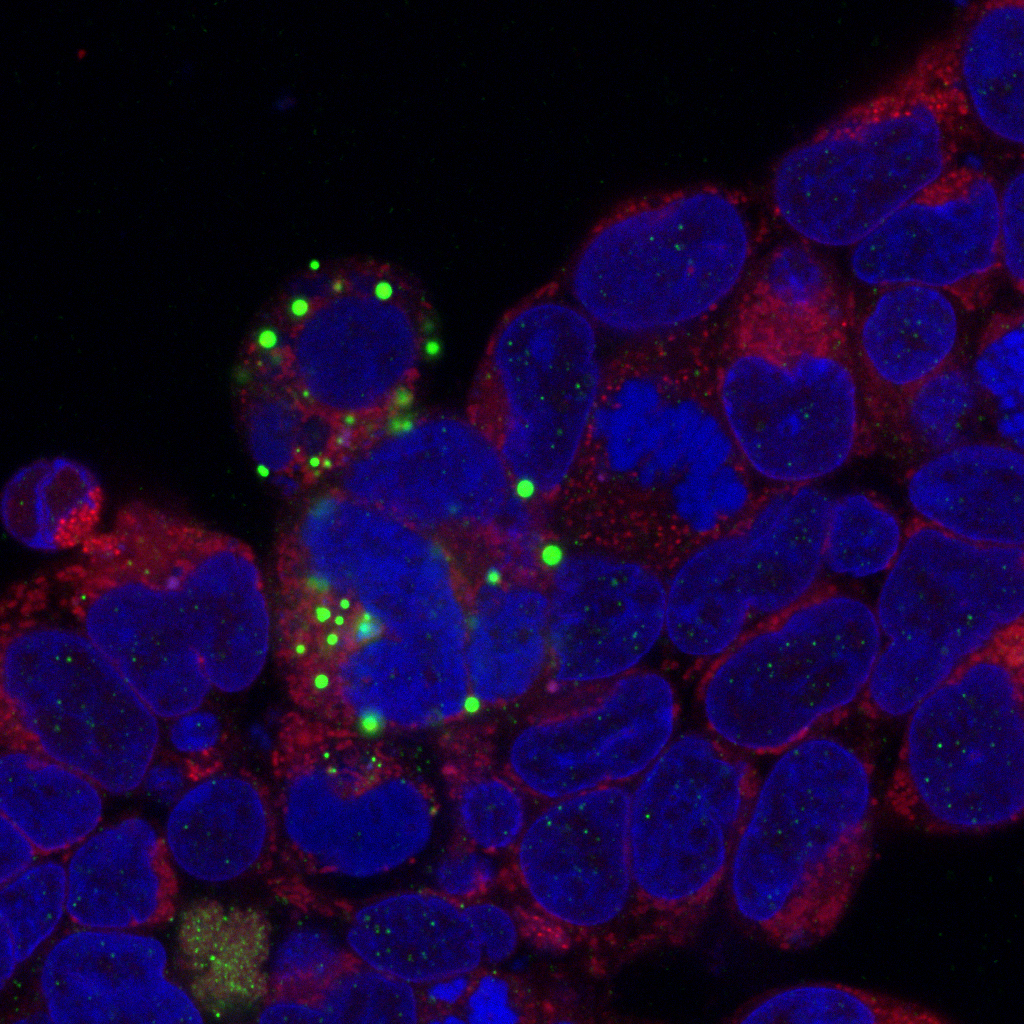

Supplement: Supplementary file 14 — Image files for Fig. 8a–c. [file 41590_2024_1902_MOESM14_ESM.zip › Fig 8b MTDR_oligomycin q333PTNIP.tif]

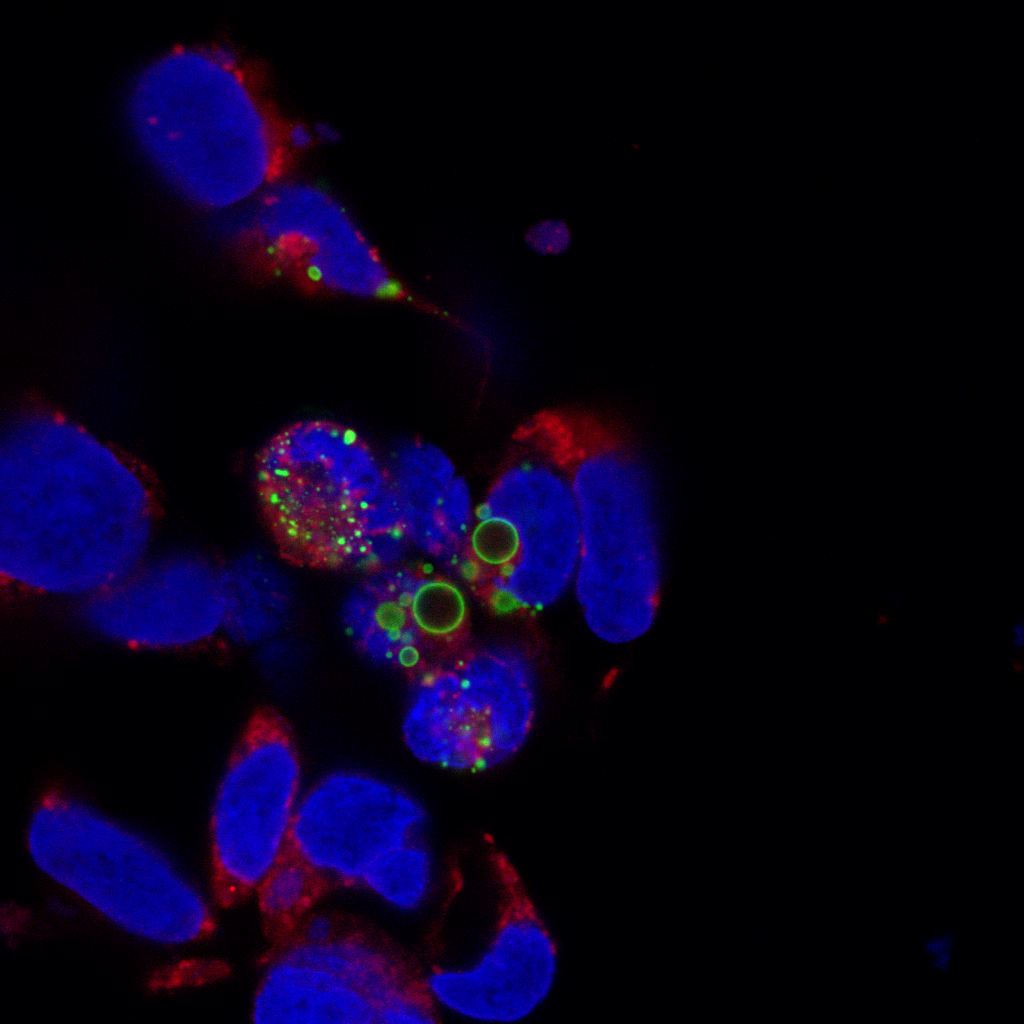

Supplement: Supplementary file 14 — Image files for Fig. 8a–c. [file 41590_2024_1902_MOESM14_ESM.zip › Fig 8a MTDR_wtTNIP.tif]

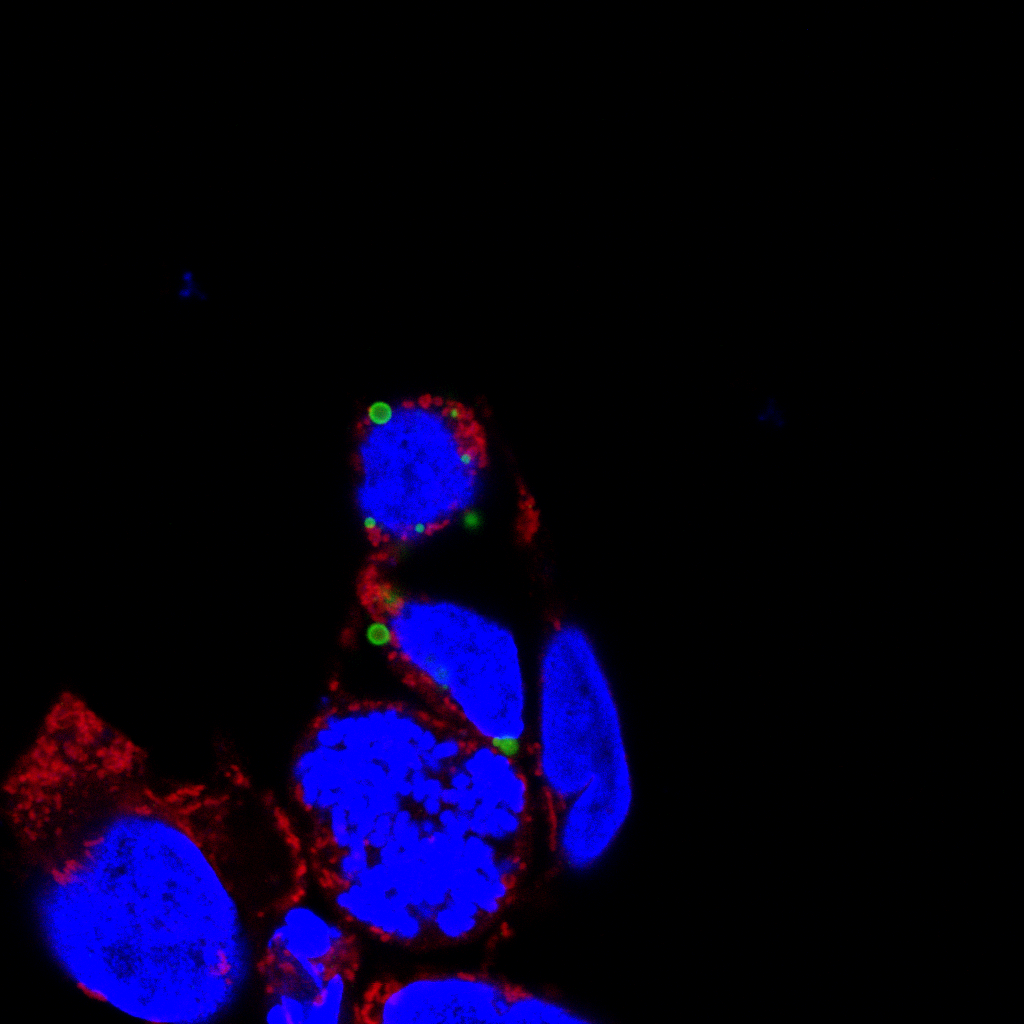

Supplement: Supplementary file 14 — Image files for Fig. 8a–c. [file 41590_2024_1902_MOESM14_ESM.zip › Fig 8a MTDR_q333pTNIP.tif]

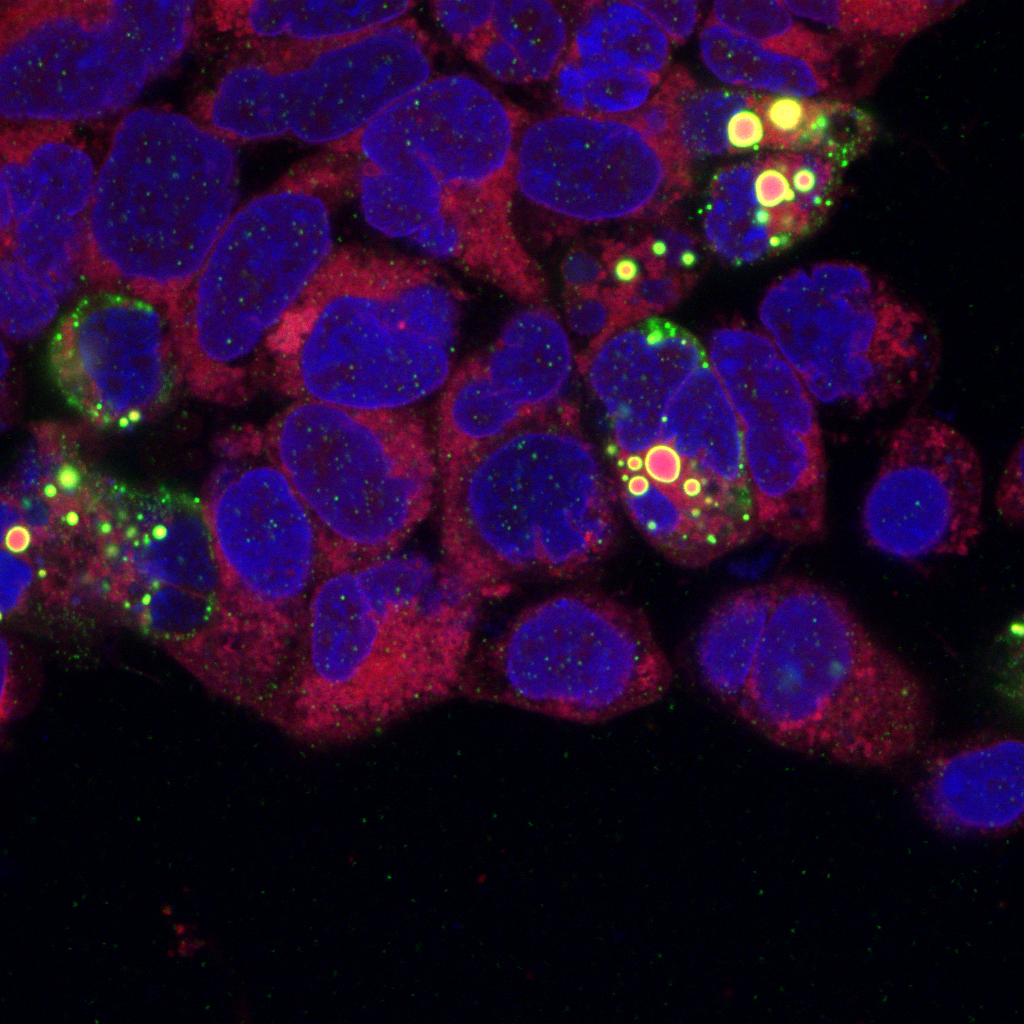

Supplement: Supplementary file 14 — Image files for Fig. 8a–c. [file 41590_2024_1902_MOESM14_ESM.zip › Fig 8b MTDR_oligomycin wtTNIP.tif]

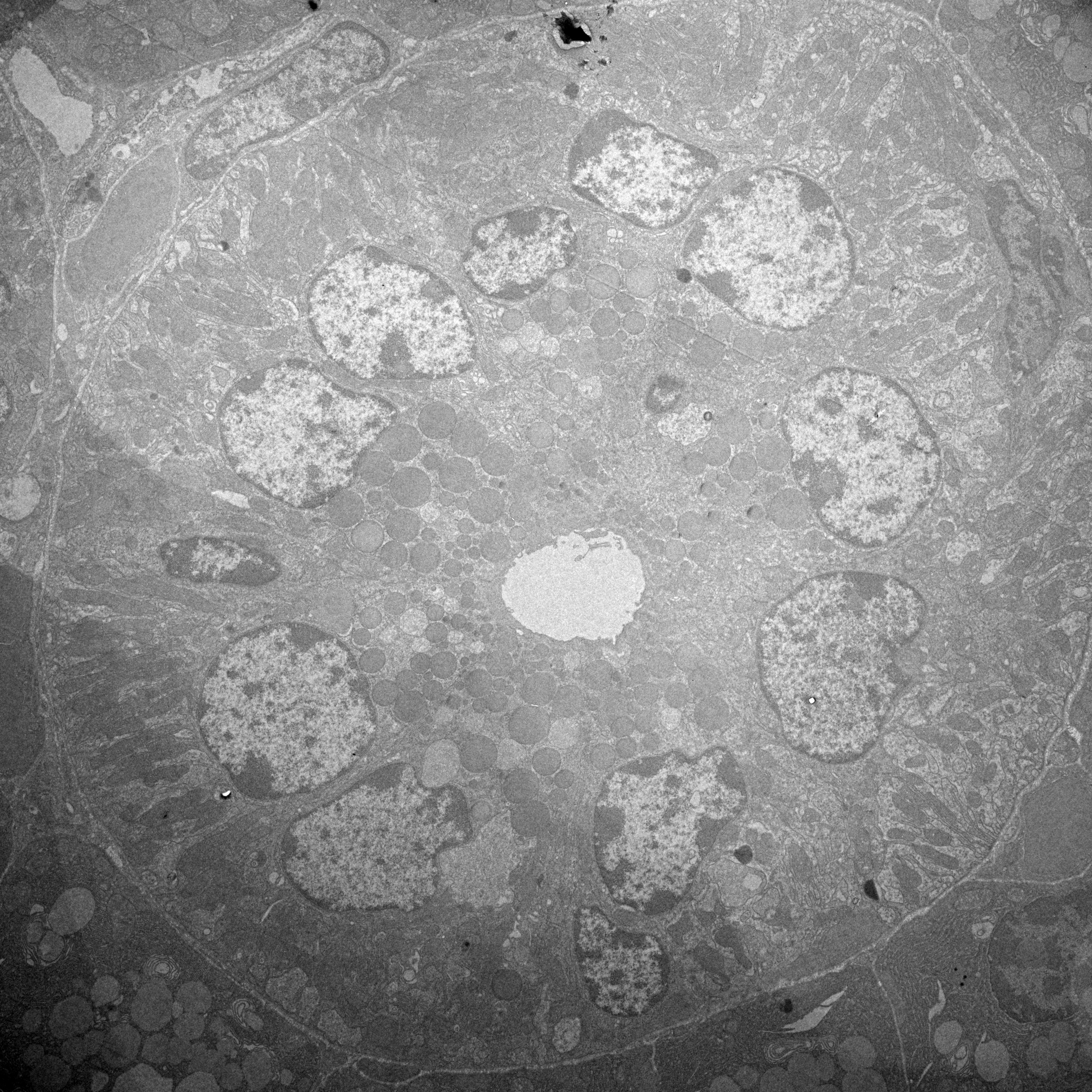

Supplement: Supplementary file 14 — Image files for Fig. 8a–c. [file 41590_2024_1902_MOESM14_ESM.zip › Fig 8c 800X _80kV _Hom_14F.jpg]

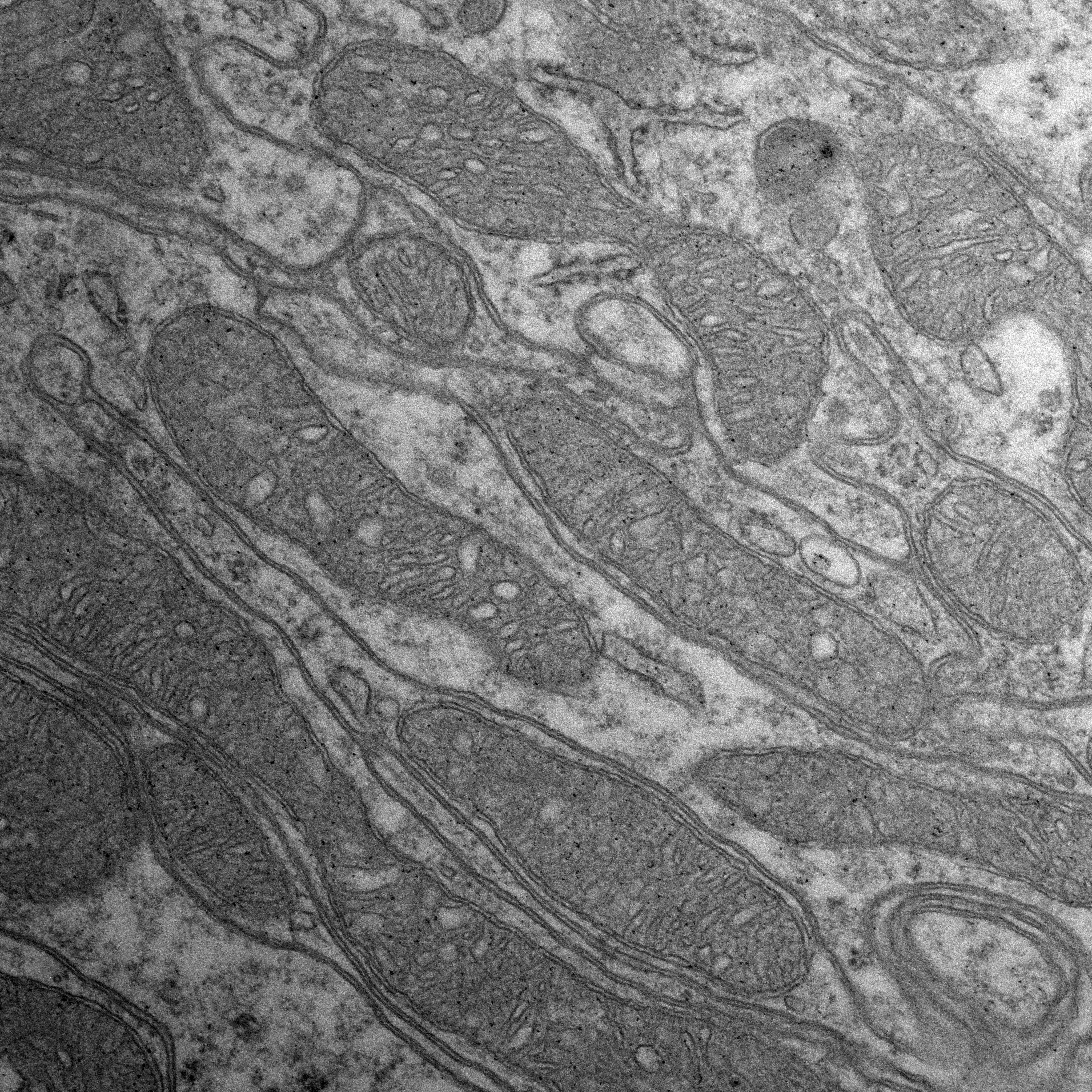

Supplement: Supplementary file 14 — Image files for Fig. 8a–c. [file 41590_2024_1902_MOESM14_ESM.zip › Fig 8c 10kX _80kV _Hom_14F.jpg]

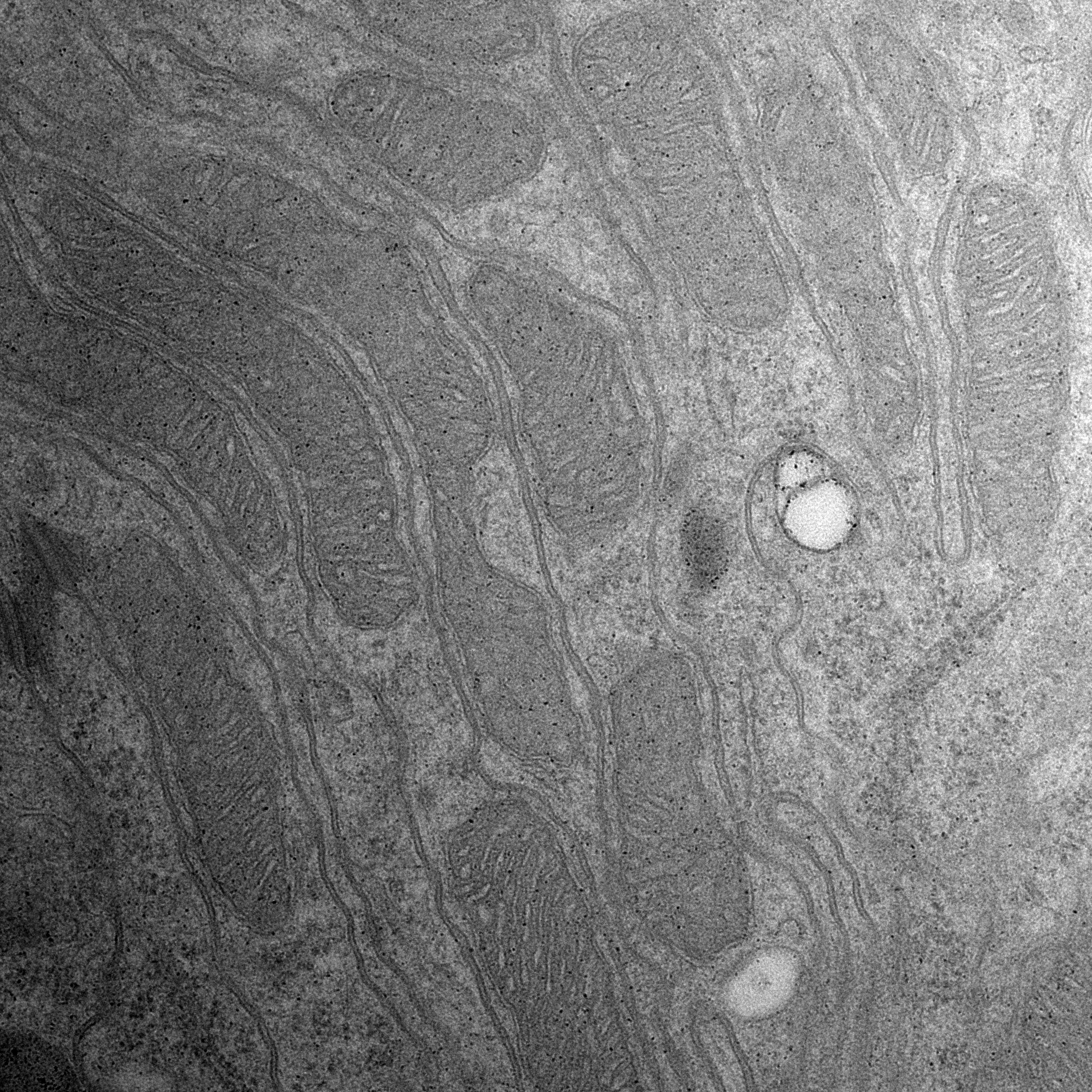

Supplement: Supplementary file 14 — Image files for Fig. 8a–c. [file 41590_2024_1902_MOESM14_ESM.zip › Fig 8c 10kX _80kV _WT_68F.jpg]

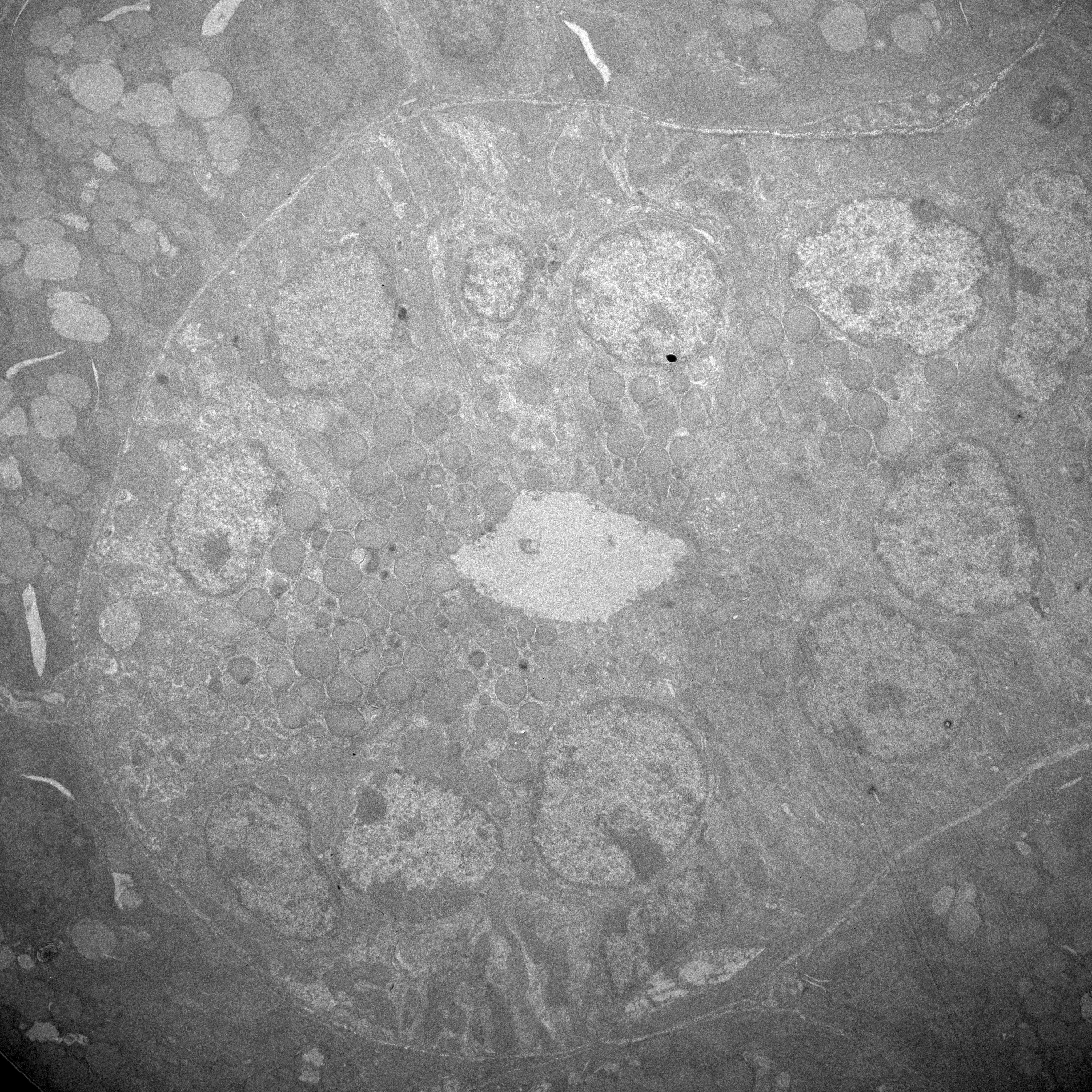

Supplement: Supplementary file 14 — Image files for Fig. 8a–c. [file 41590_2024_1902_MOESM14_ESM.zip › Fig 8c 800X _80kV _WT_68F.jpg]

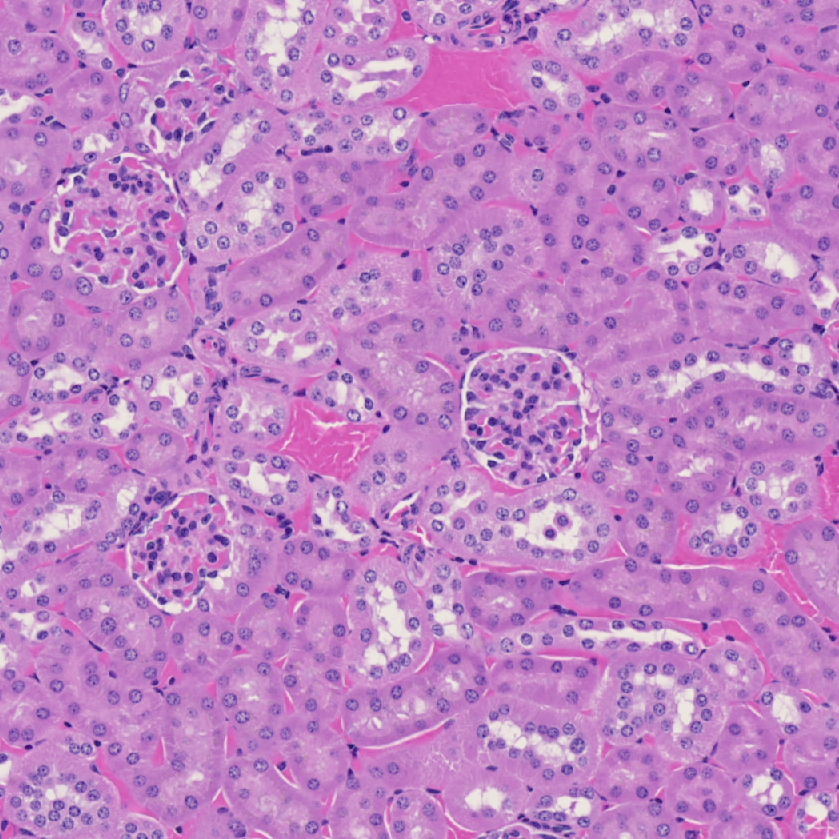

Supplement: Supplementary file 16 — Image files for Extended Data Fig. 2c,e. [file 41590_2024_1902_MOESM16_ESM.zip › Wildtype_Kidney_Vikala.tif]

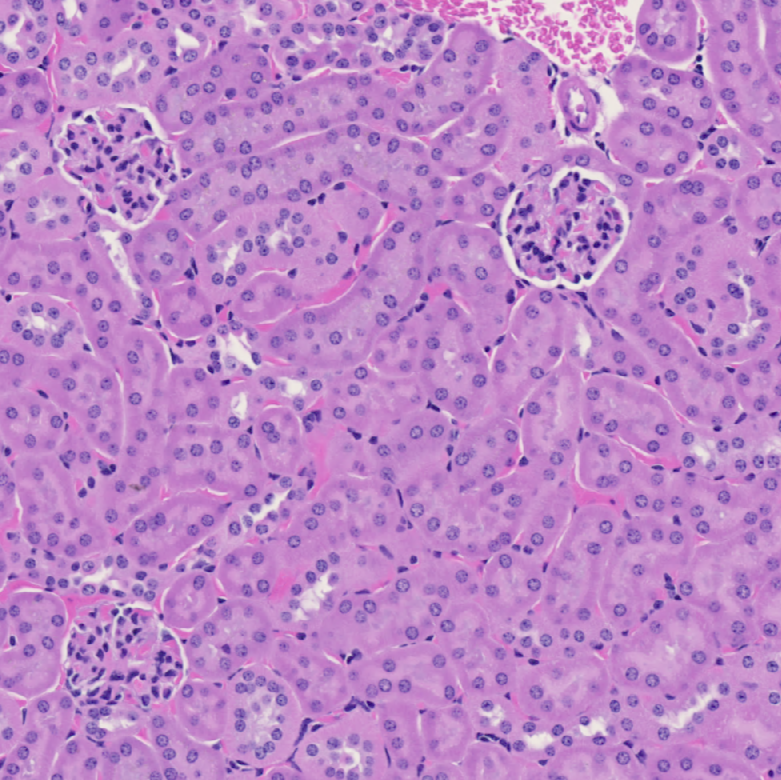

Supplement: Supplementary file 16 — Image files for Extended Data Fig. 2c,e. [file 41590_2024_1902_MOESM16_ESM.zip › Homozygous_Kidney_Vikala.tif]

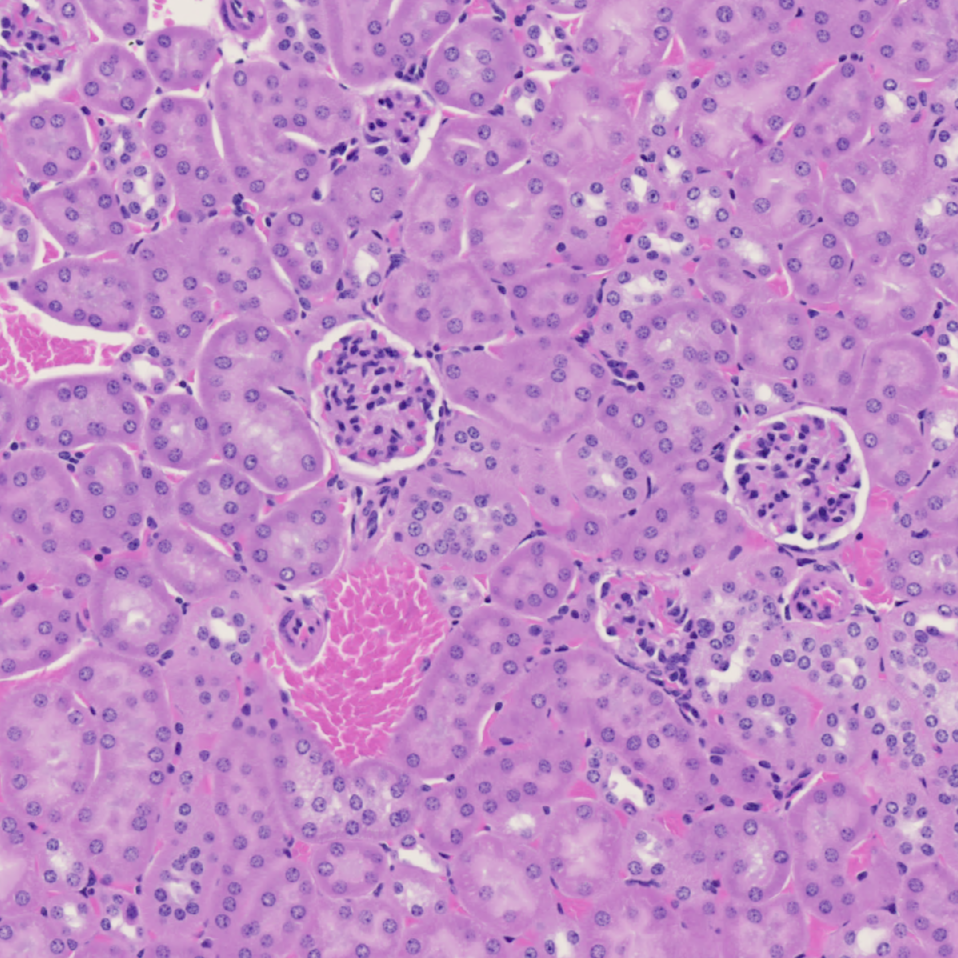

Supplement: Supplementary file 16 — Image files for Extended Data Fig. 2c,e. [file 41590_2024_1902_MOESM16_ESM.zip › Heterozygous_Kidney_Vikala.tif]

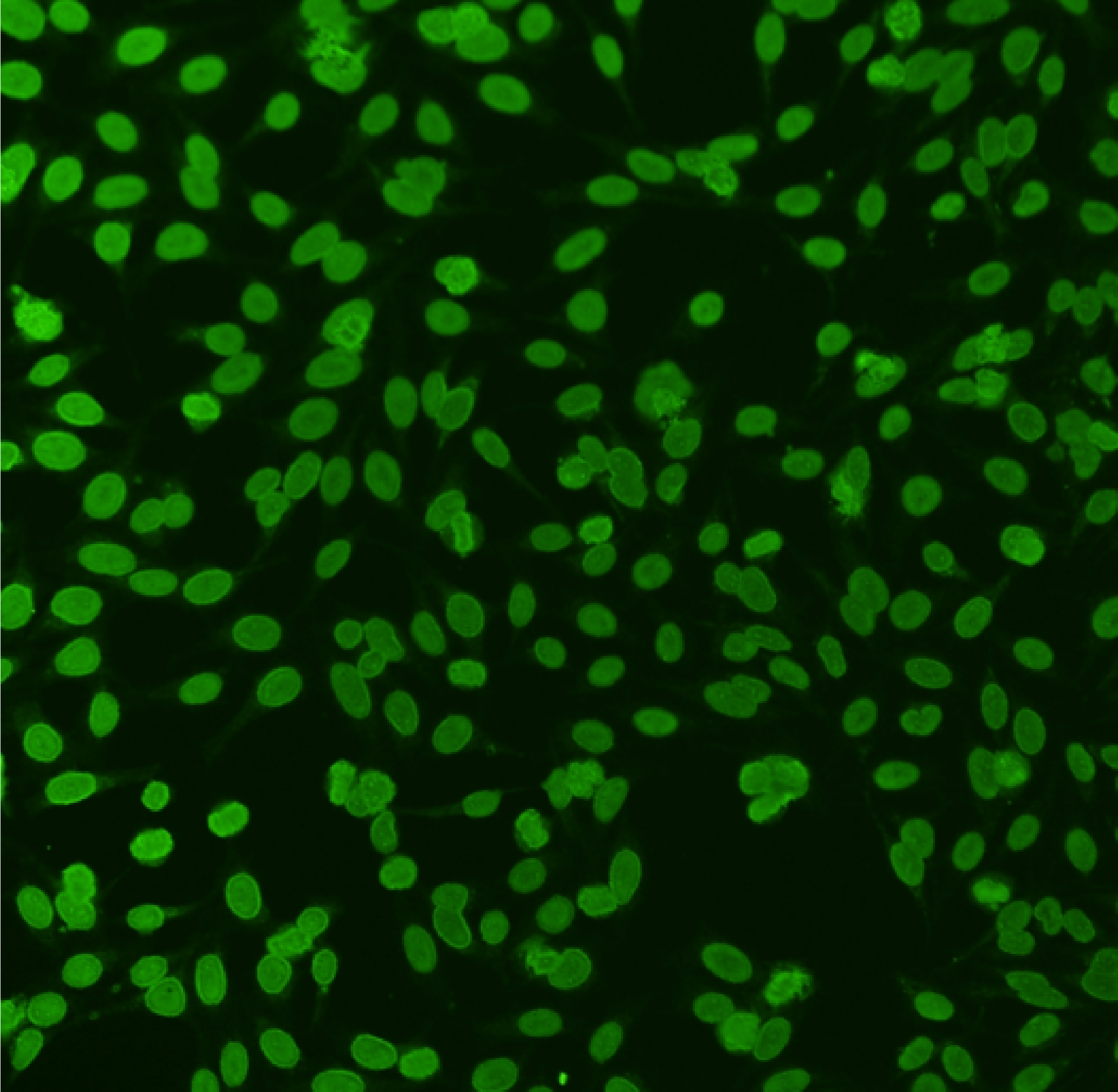

Supplement: Supplementary file 16 — Image files for Extended Data Fig. 2c,e. [file 41590_2024_1902_MOESM16_ESM.zip › ext 2c Hep2 ANA homogenous.tif]

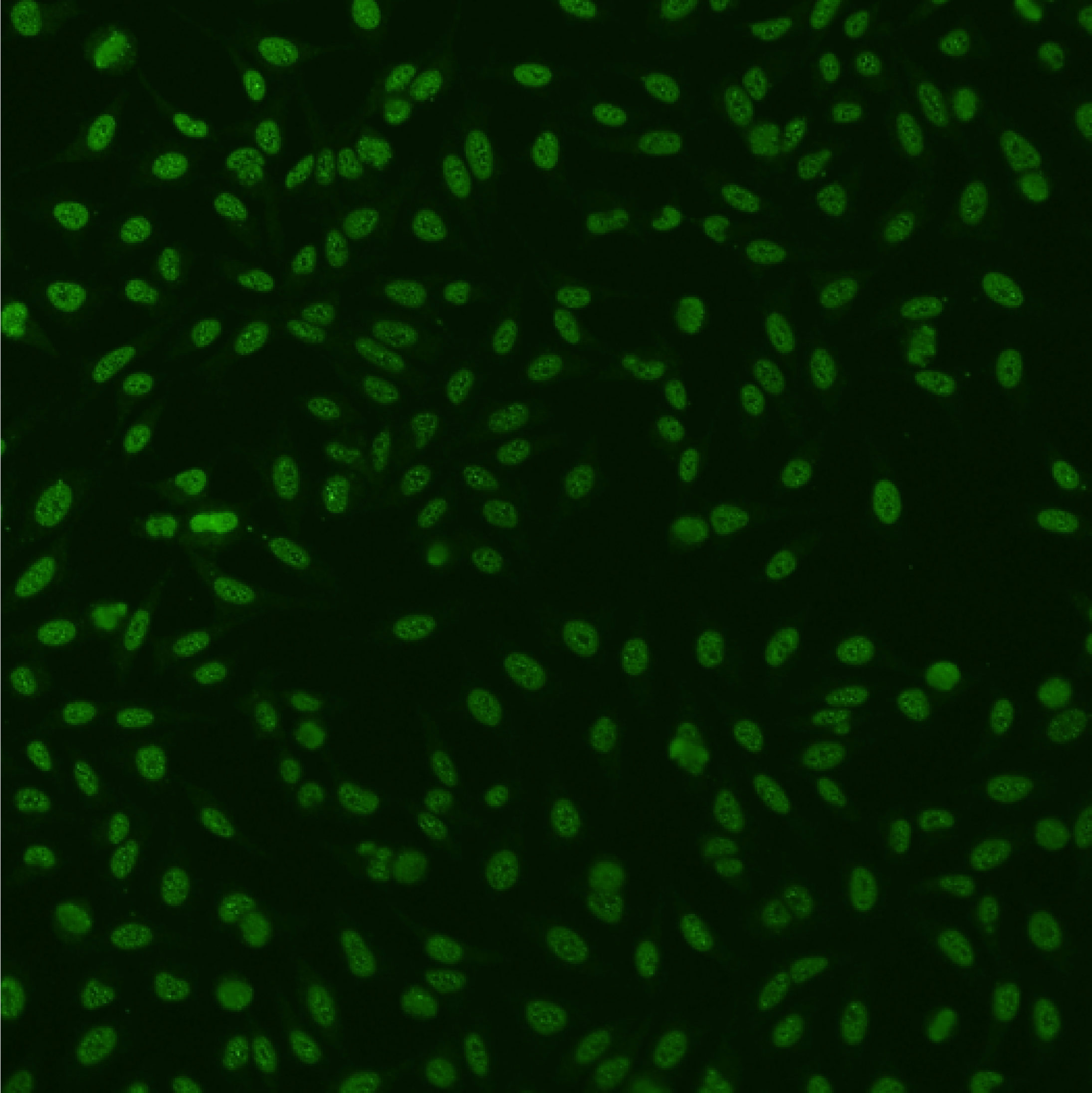

Supplement: Supplementary file 16 — Image files for Extended Data Fig. 2c,e. [file 41590_2024_1902_MOESM16_ESM.zip › ext 2c Hep2 ANA 2 speckled.tif]

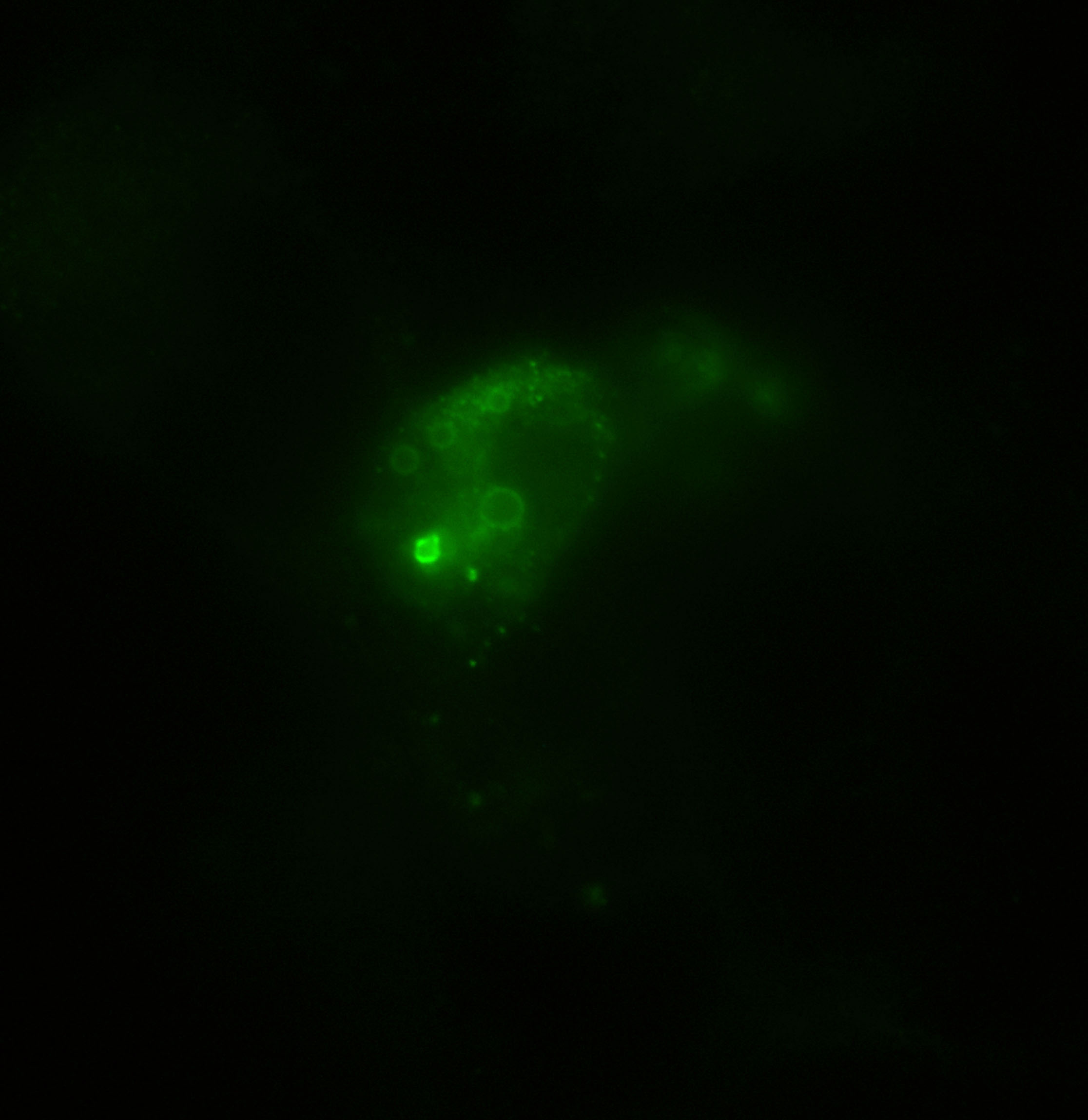

Supplement: Supplementary file 17 — Image files for Extended Data Fig. 5a–h. [file 41590_2024_1902_MOESM17_ESM.zip › ED Fig 5h WTTNIP-LC3bFLAG-tnip.jpg]

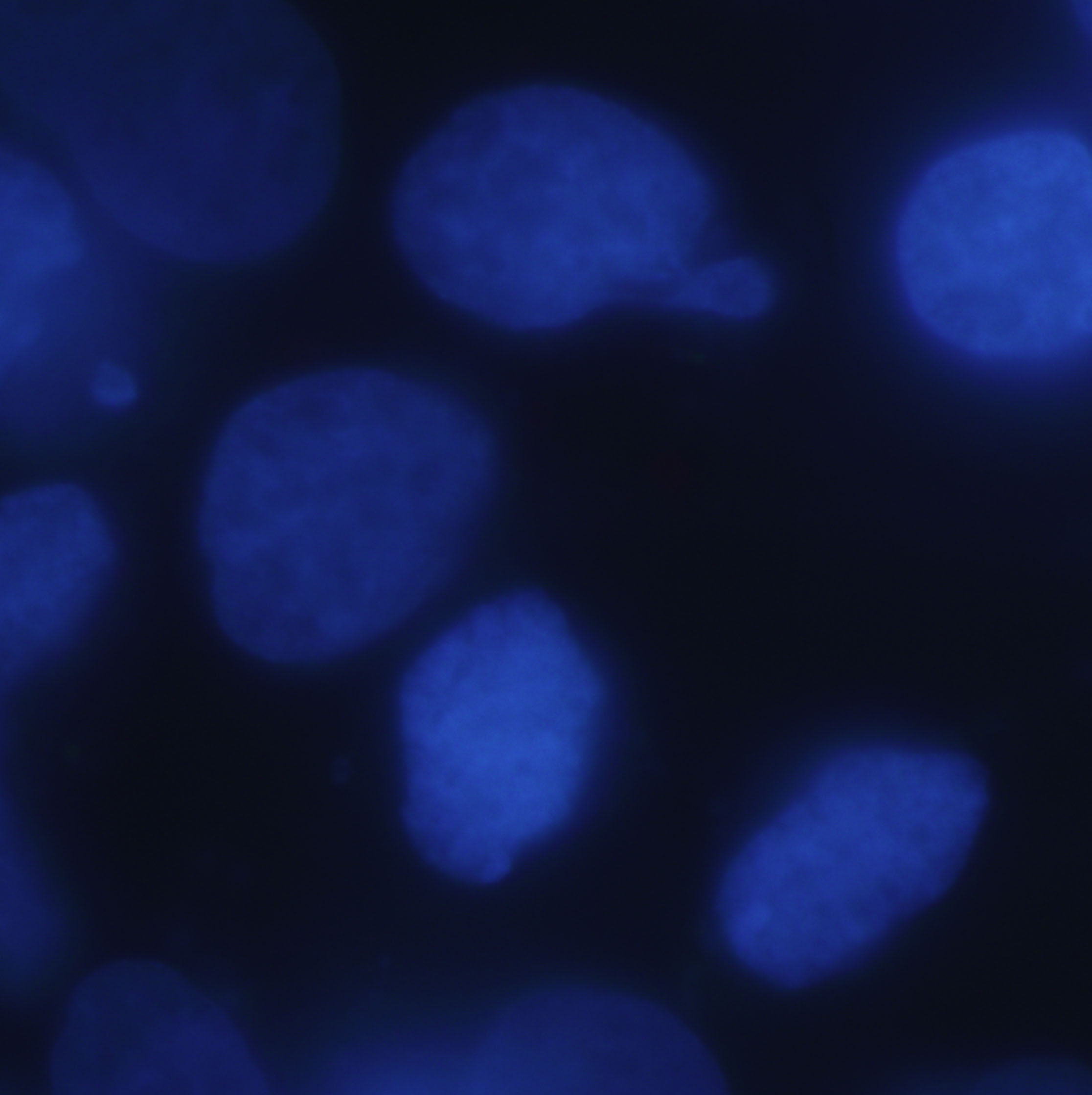

Supplement: Supplementary file 17 — Image files for Extended Data Fig. 5a–h. [file 41590_2024_1902_MOESM17_ESM.zip › ED Fig 5a Q333PTNIP+T6antip62TNIP-dna.jpg]

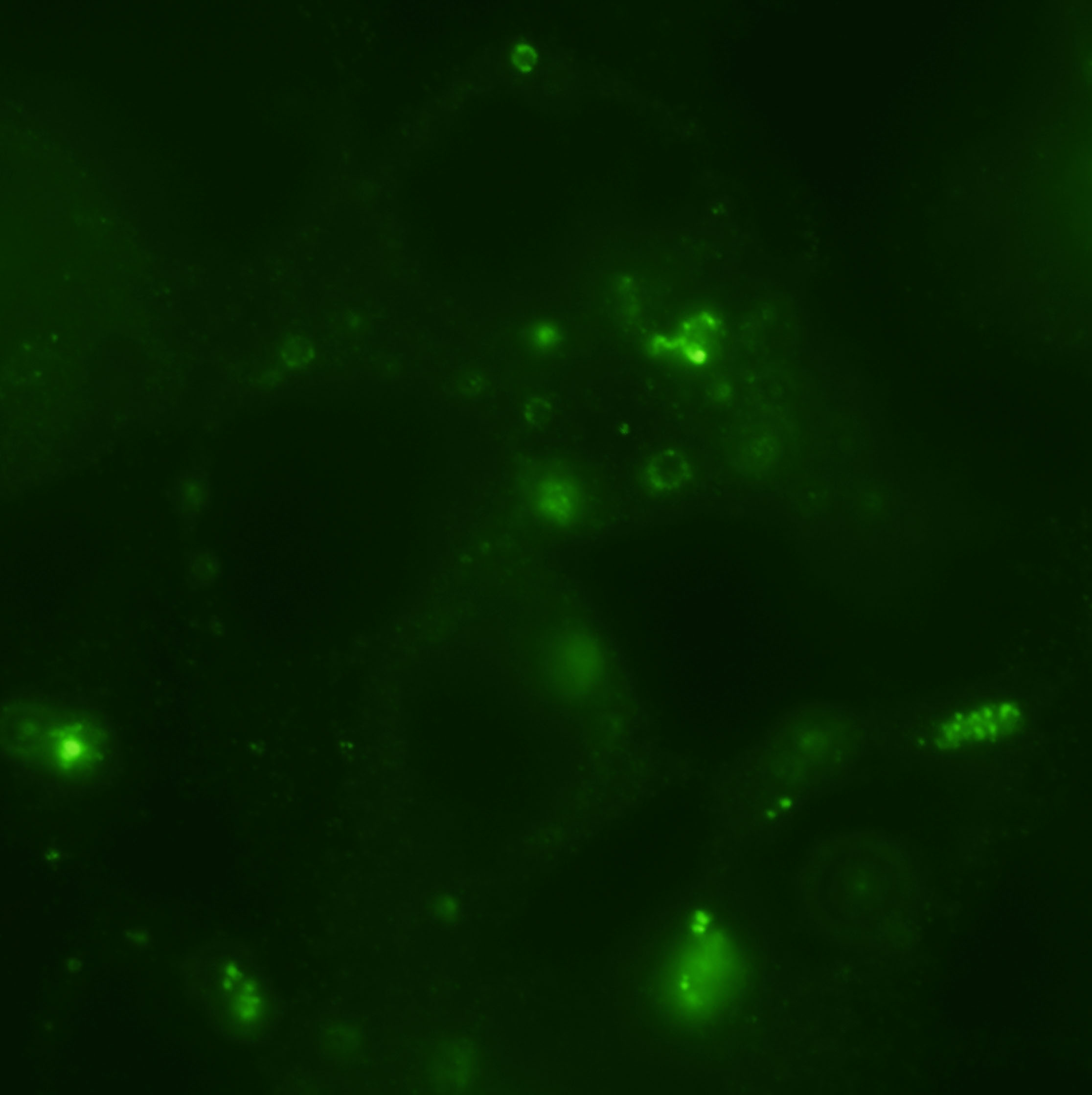

Supplement: Supplementary file 17 — Image files for Extended Data Fig. 5a–h. [file 41590_2024_1902_MOESM17_ESM.zip › ED Fig 5a Q333PTNIP+T6antip62TNIP-p62.jpg]

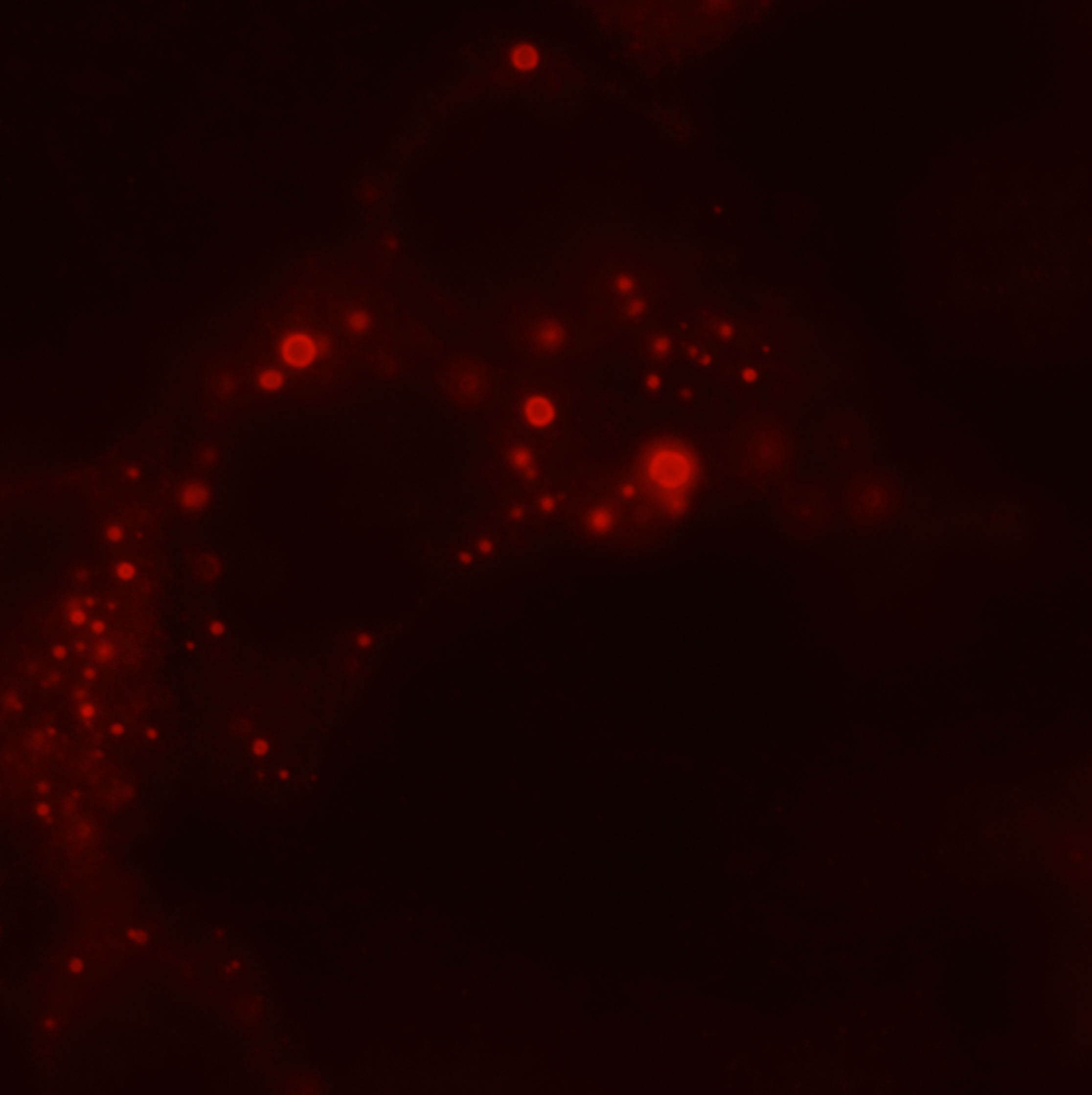

Supplement: Supplementary file 17 — Image files for Extended Data Fig. 5a–h. [file 41590_2024_1902_MOESM17_ESM.zip › ED Fig 5a Q333PTNIP+T6antip62TNIP-tnip.jpg]

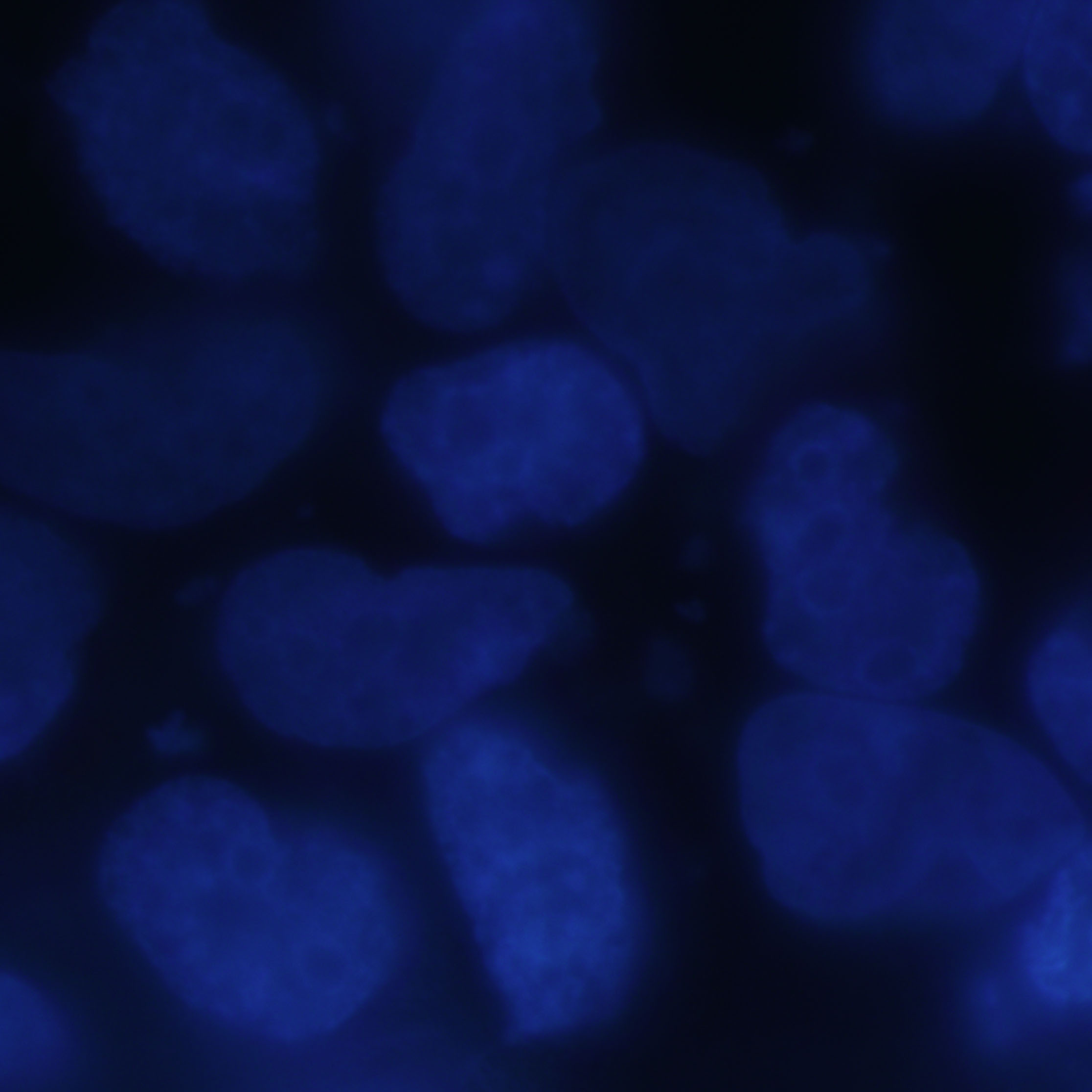

Supplement: Supplementary file 17 — Image files for Extended Data Fig. 5a–h. [file 41590_2024_1902_MOESM17_ESM.zip › ED Fig 5a WTTNIP+T6antip62TNIP-dna.jpg]

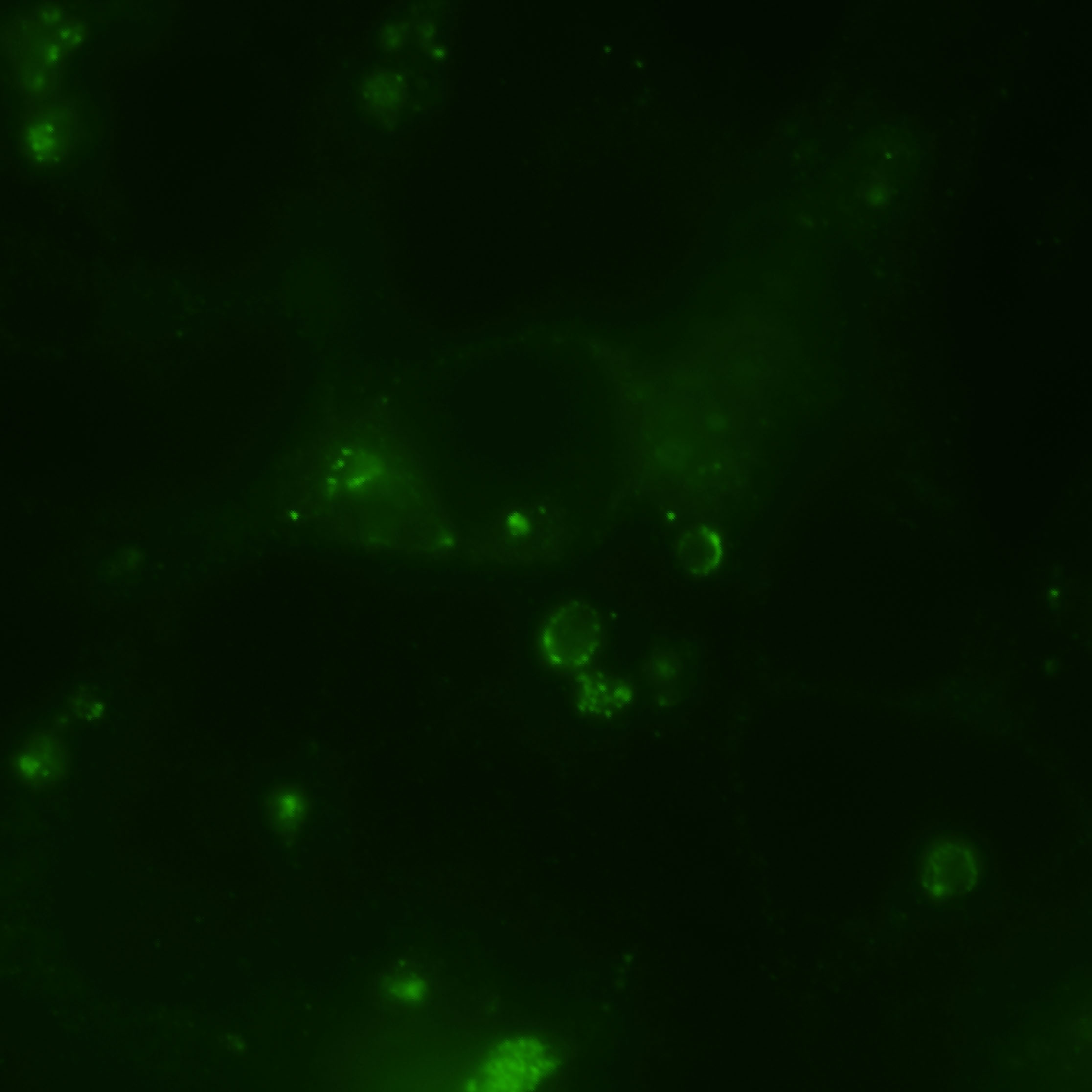

Supplement: Supplementary file 17 — Image files for Extended Data Fig. 5a–h. [file 41590_2024_1902_MOESM17_ESM.zip › ED Fig 5a WTTNIP+T6antip62TNIP-p62.jpg]

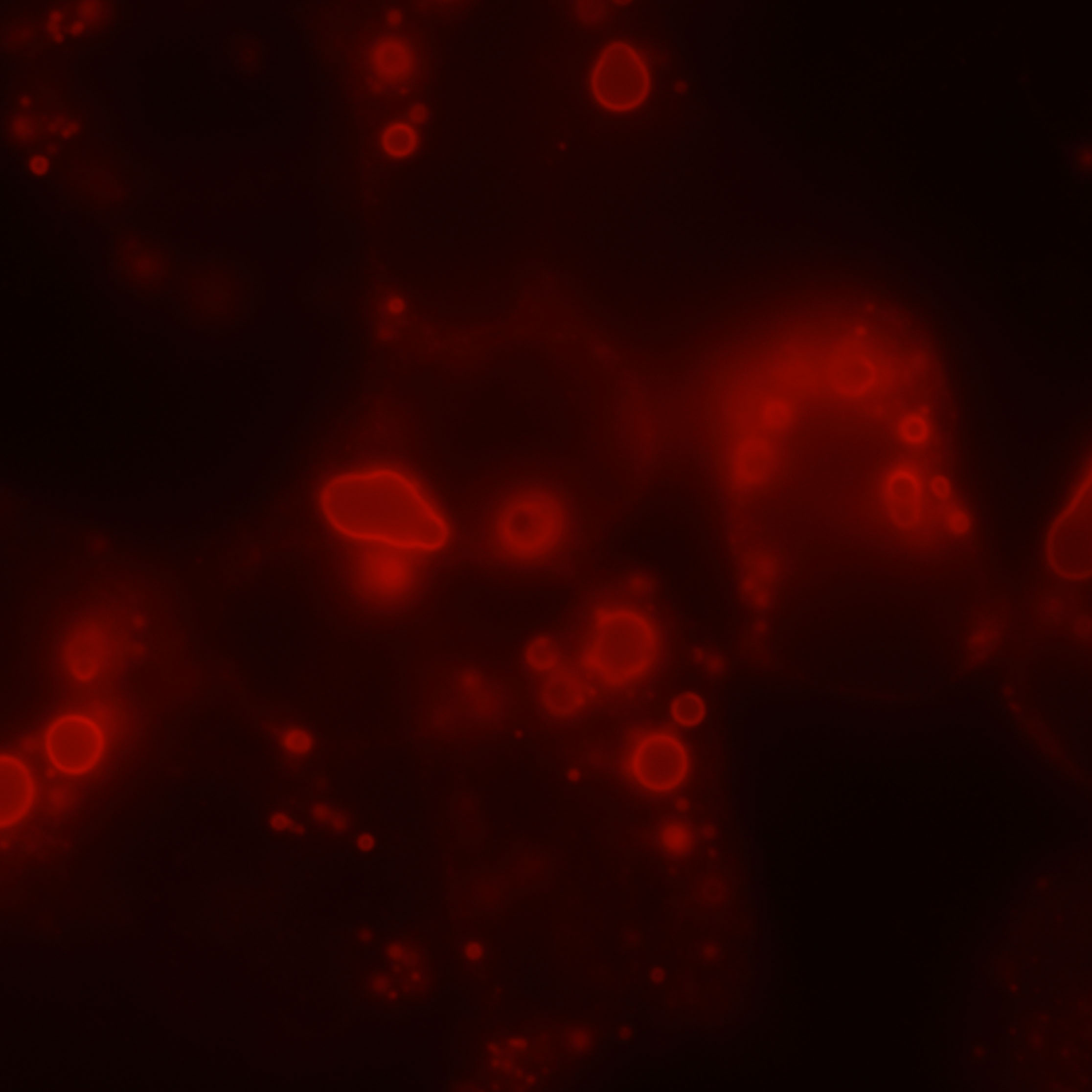

Supplement: Supplementary file 17 — Image files for Extended Data Fig. 5a–h. [file 41590_2024_1902_MOESM17_ESM.zip › ED Fig 5a WTTNIP+T6antip62TNIP-tnip.jpg]

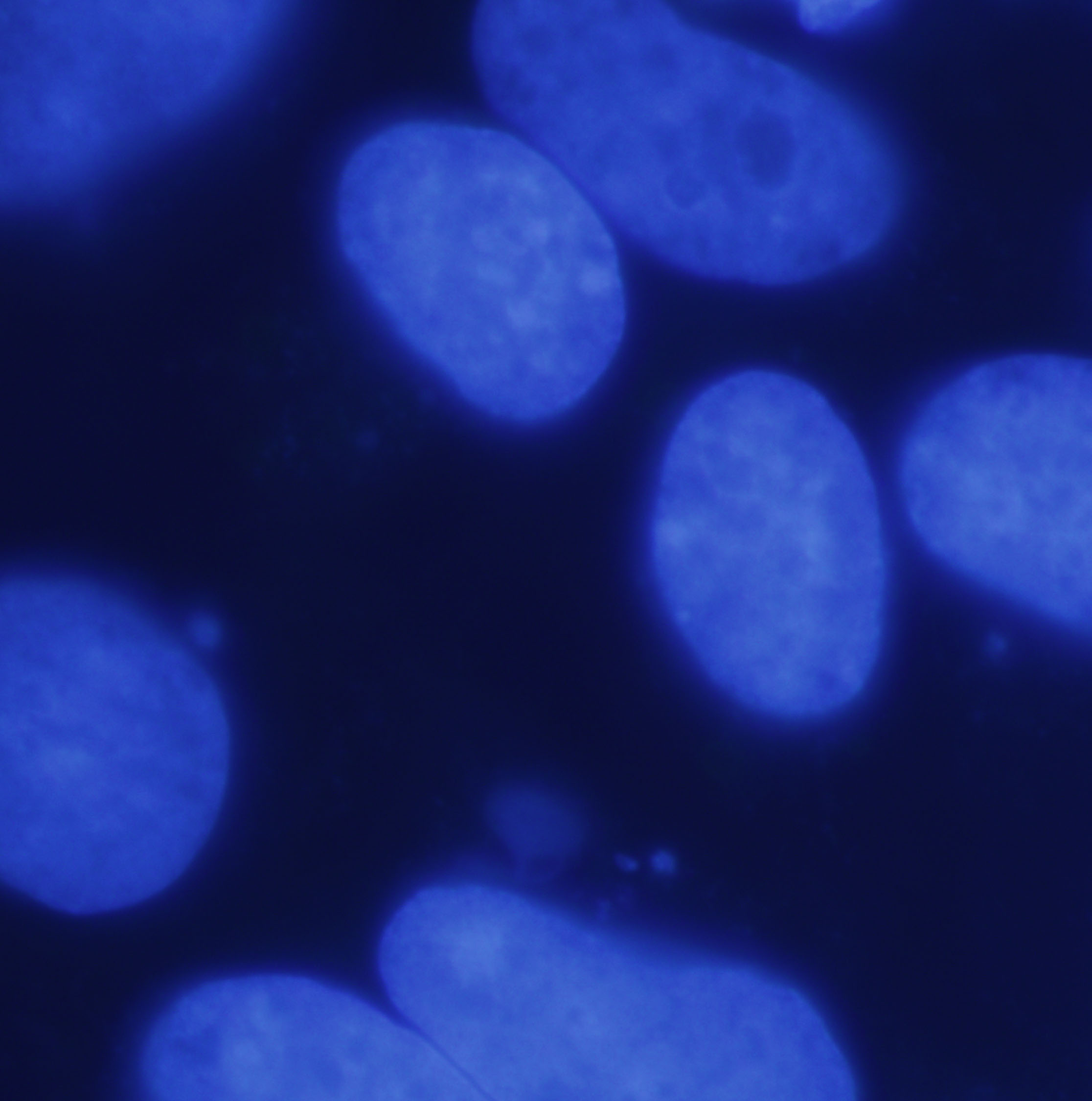

Supplement: Supplementary file 17 — Image files for Extended Data Fig. 5a–h. [file 41590_2024_1902_MOESM17_ESM.zip › ED Fig 5b Q333PTNIP+TRAF6-dna.jpg]

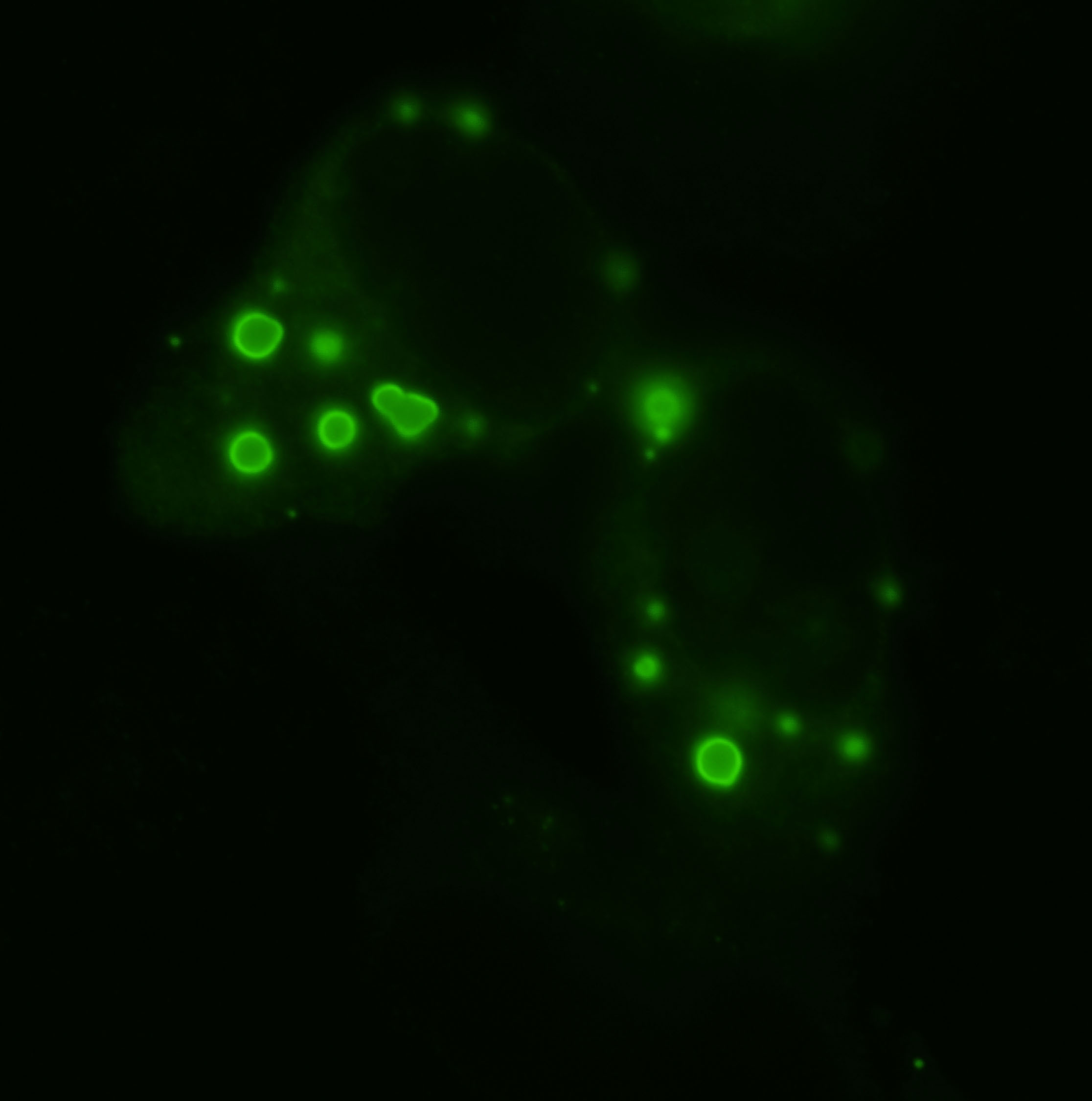

Supplement: Supplementary file 17 — Image files for Extended Data Fig. 5a–h. [file 41590_2024_1902_MOESM17_ESM.zip › ED Fig 5b Q333PTNIP+TRAF6-tnip.jpg]

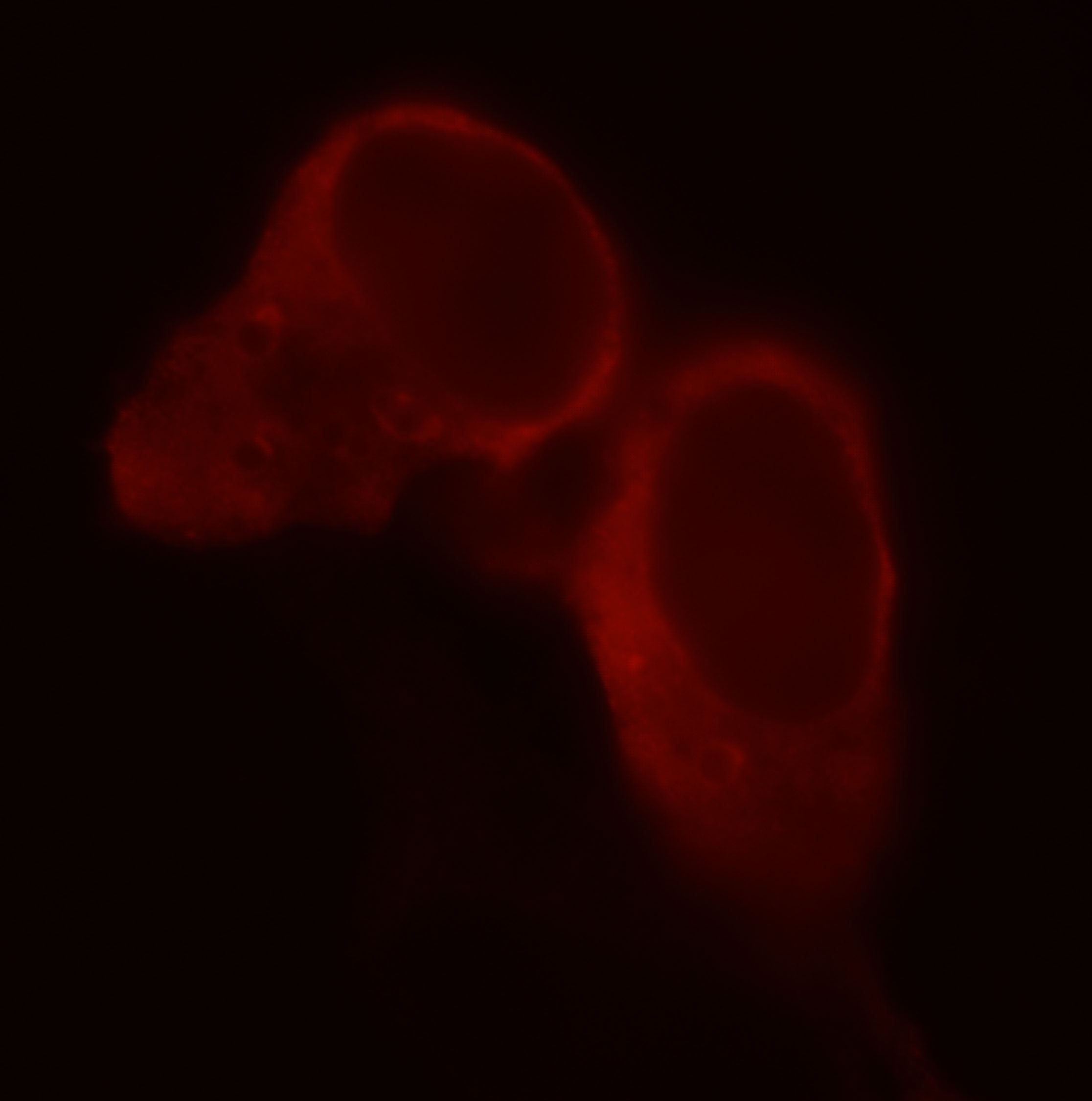

Supplement: Supplementary file 17 — Image files for Extended Data Fig. 5a–h. [file 41590_2024_1902_MOESM17_ESM.zip › ED Fig 5b Q333PTNIP+TRAF6-traf6.jpg]

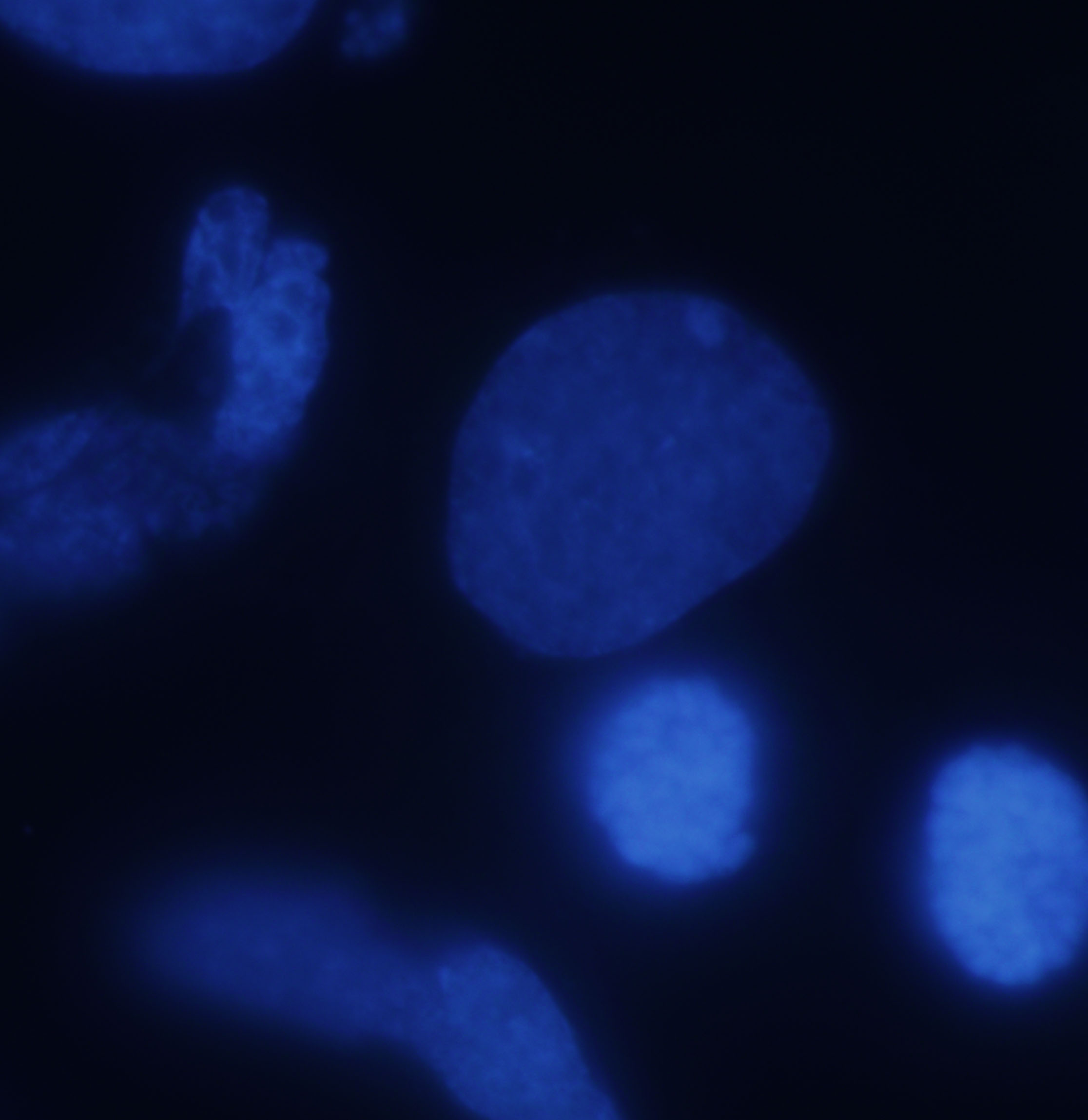

Supplement: Supplementary file 17 — Image files for Extended Data Fig. 5a–h. [file 41590_2024_1902_MOESM17_ESM.zip › ED Fig 5b WTTNIP1+TRAF6-dna.jpg]

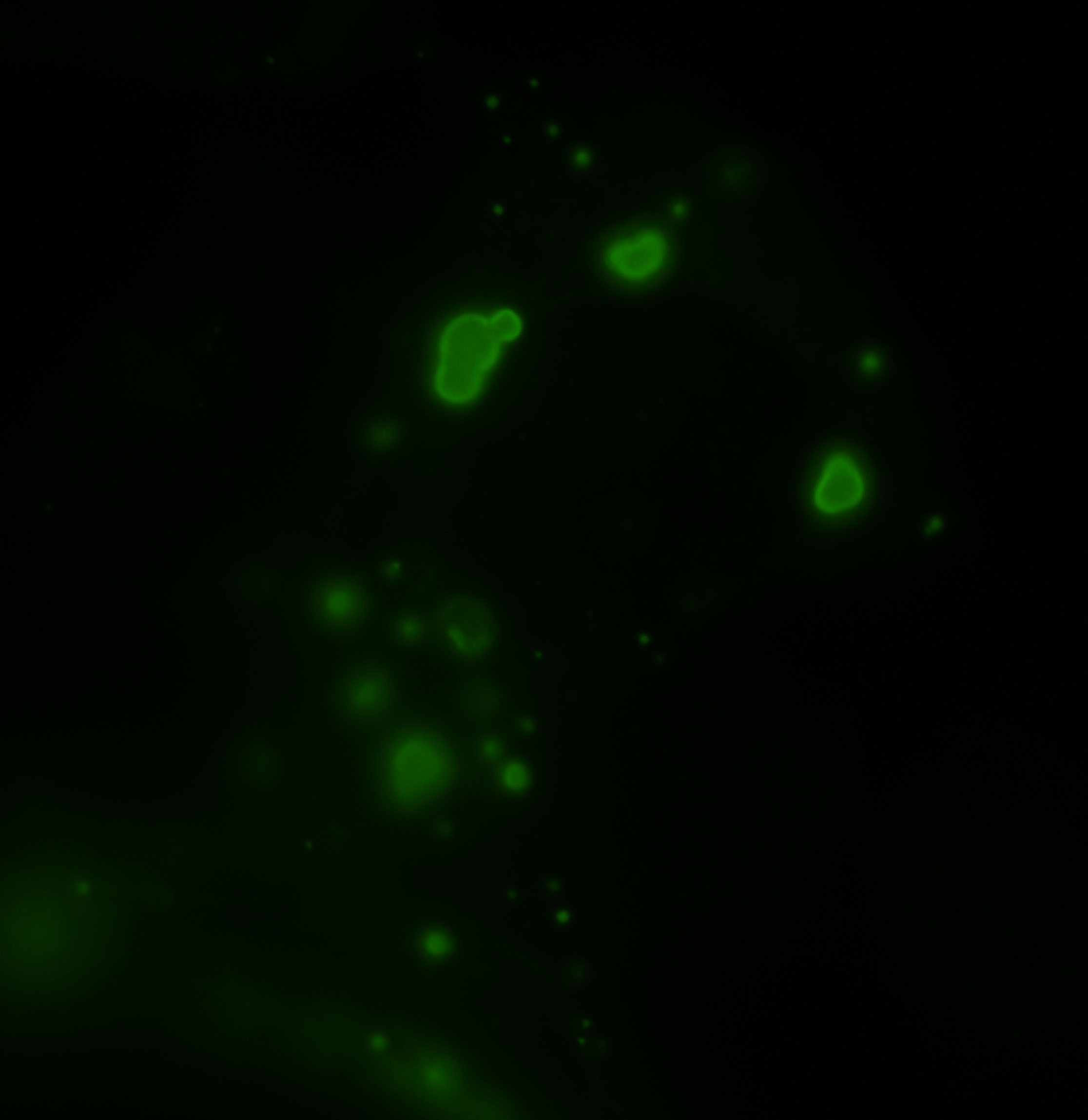

Supplement: Supplementary file 17 — Image files for Extended Data Fig. 5a–h. [file 41590_2024_1902_MOESM17_ESM.zip › ED Fig 5b WTTNIP1+TRAF6-tnip.jpg]

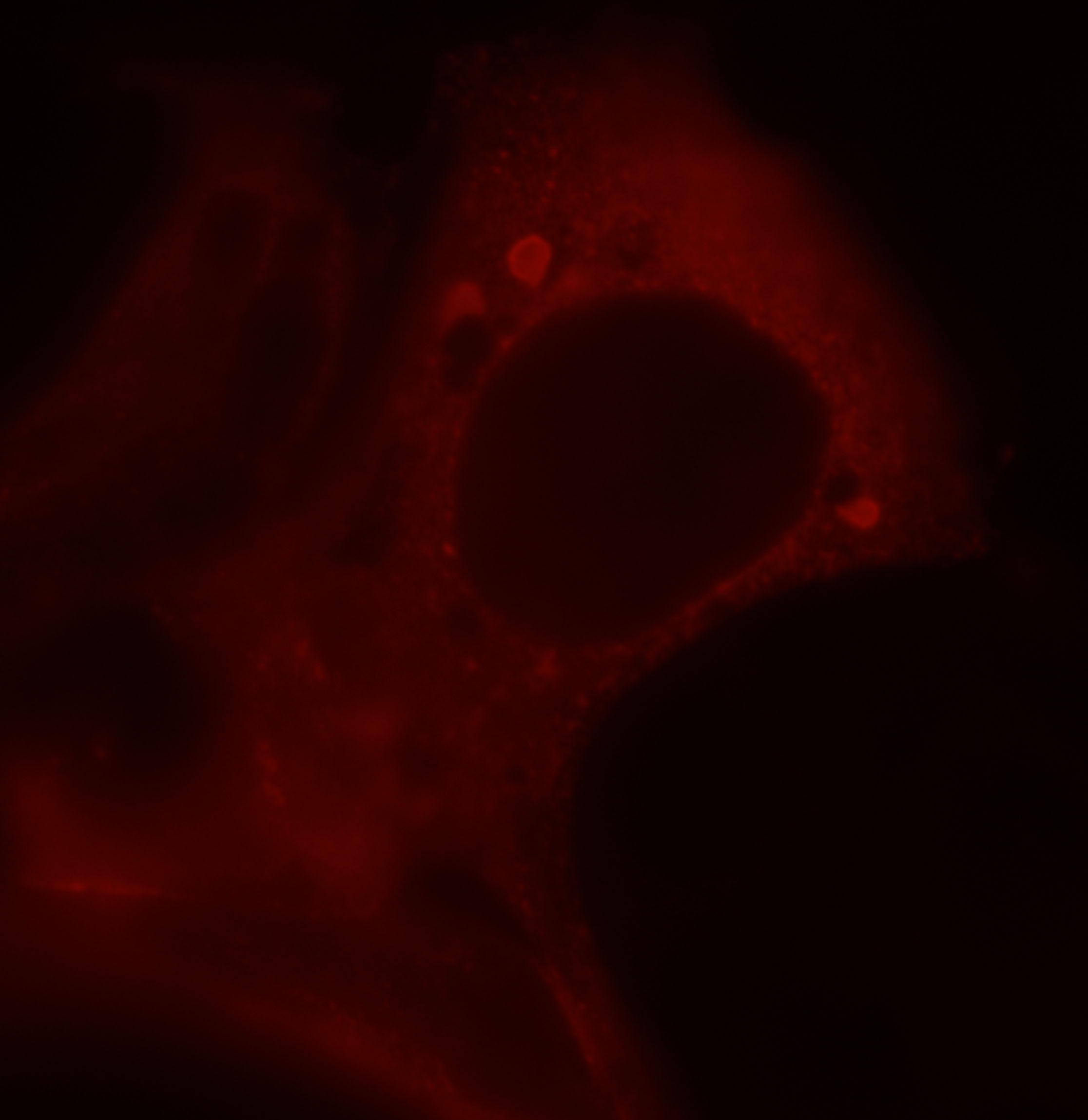

Supplement: Supplementary file 17 — Image files for Extended Data Fig. 5a–h. [file 41590_2024_1902_MOESM17_ESM.zip › ED Fig 5b WTTNIP1+TRAF6-traf6.jpg]

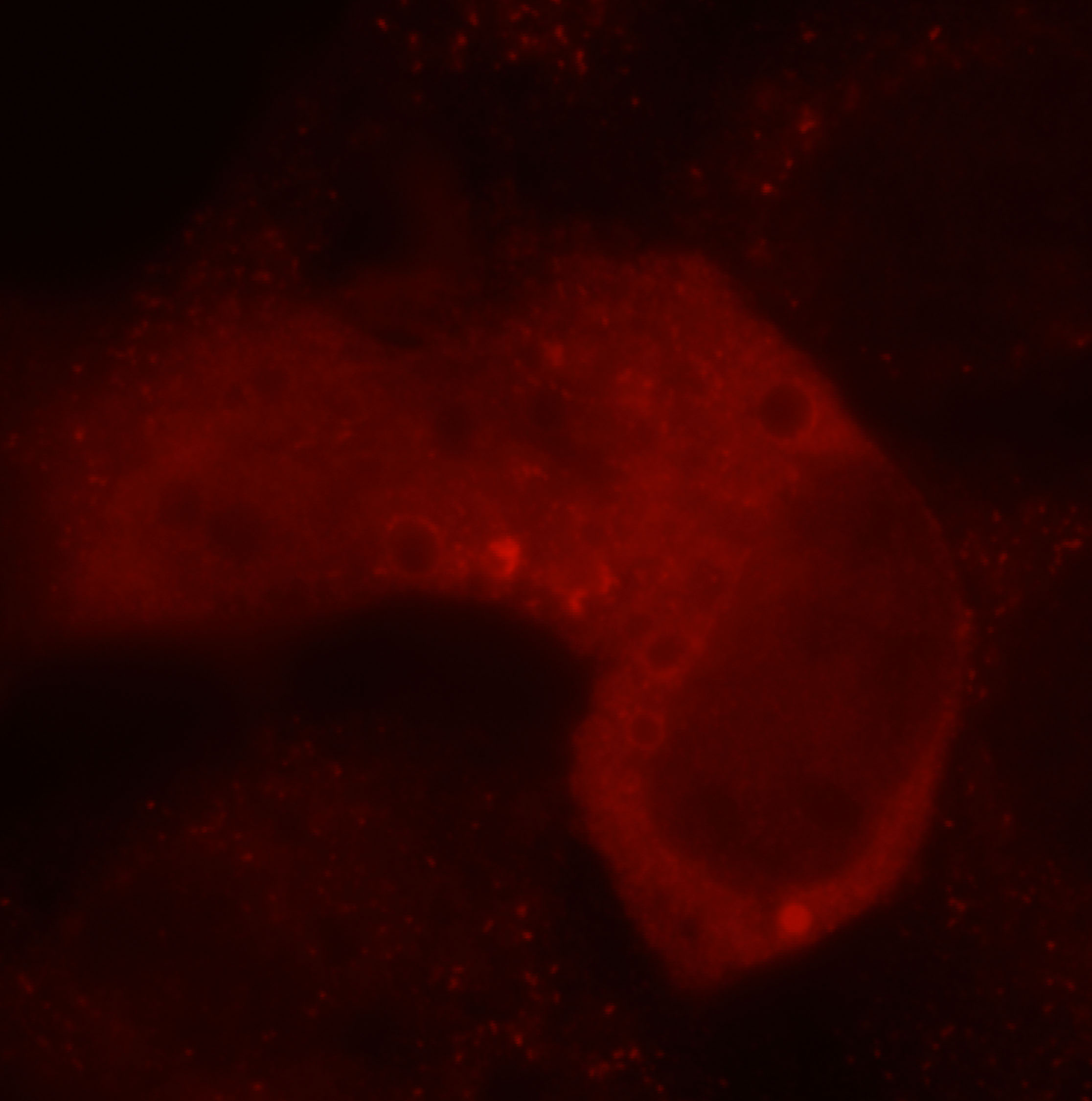

Supplement: Supplementary file 17 — Image files for Extended Data Fig. 5a–h. [file 41590_2024_1902_MOESM17_ESM.zip › ED Fig 5c Q333PTNIP+A20myc-a20.jpg]

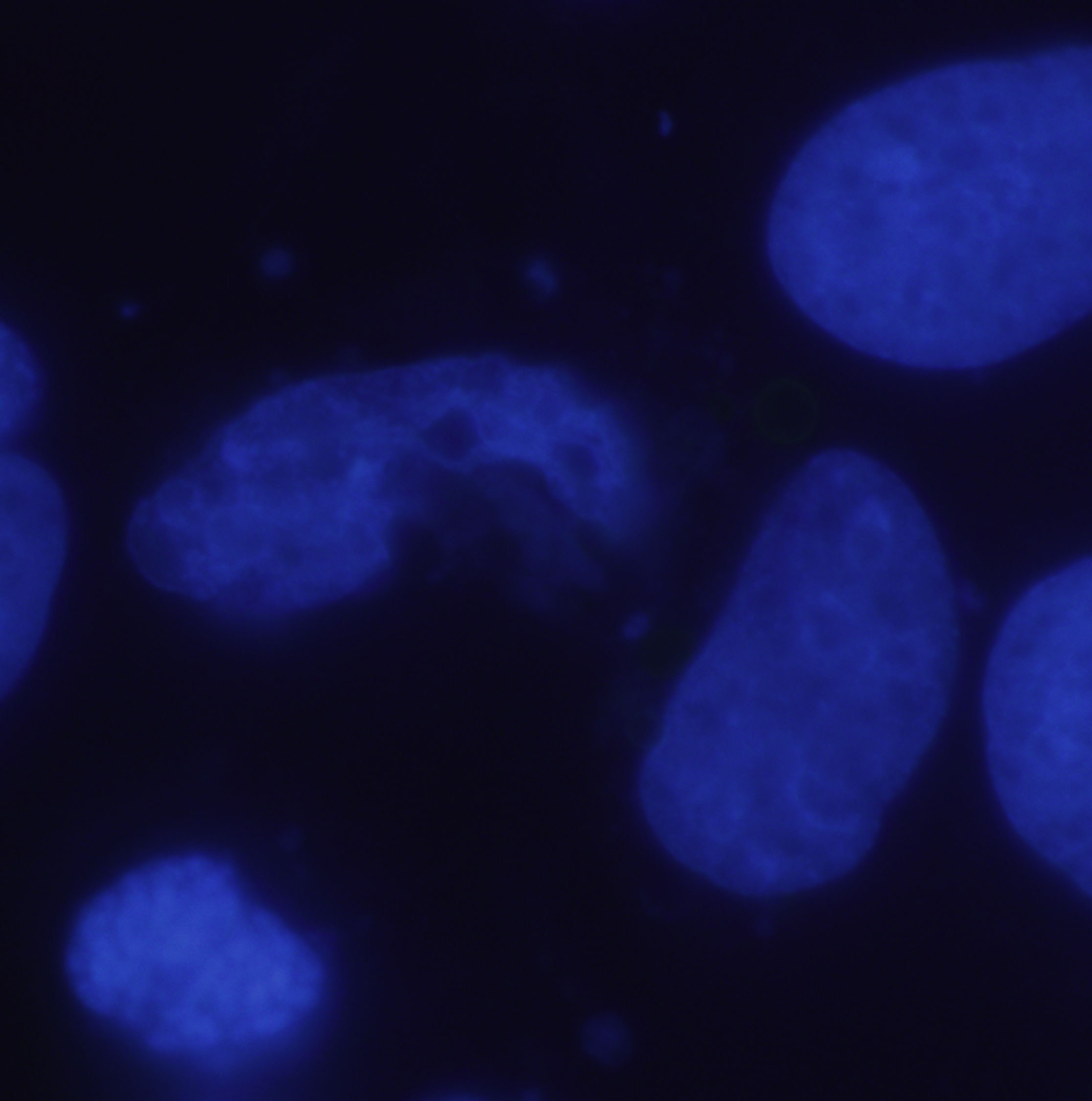

Supplement: Supplementary file 17 — Image files for Extended Data Fig. 5a–h. [file 41590_2024_1902_MOESM17_ESM.zip › ED Fig 5c Q333PTNIP+A20myc-dna.jpg]

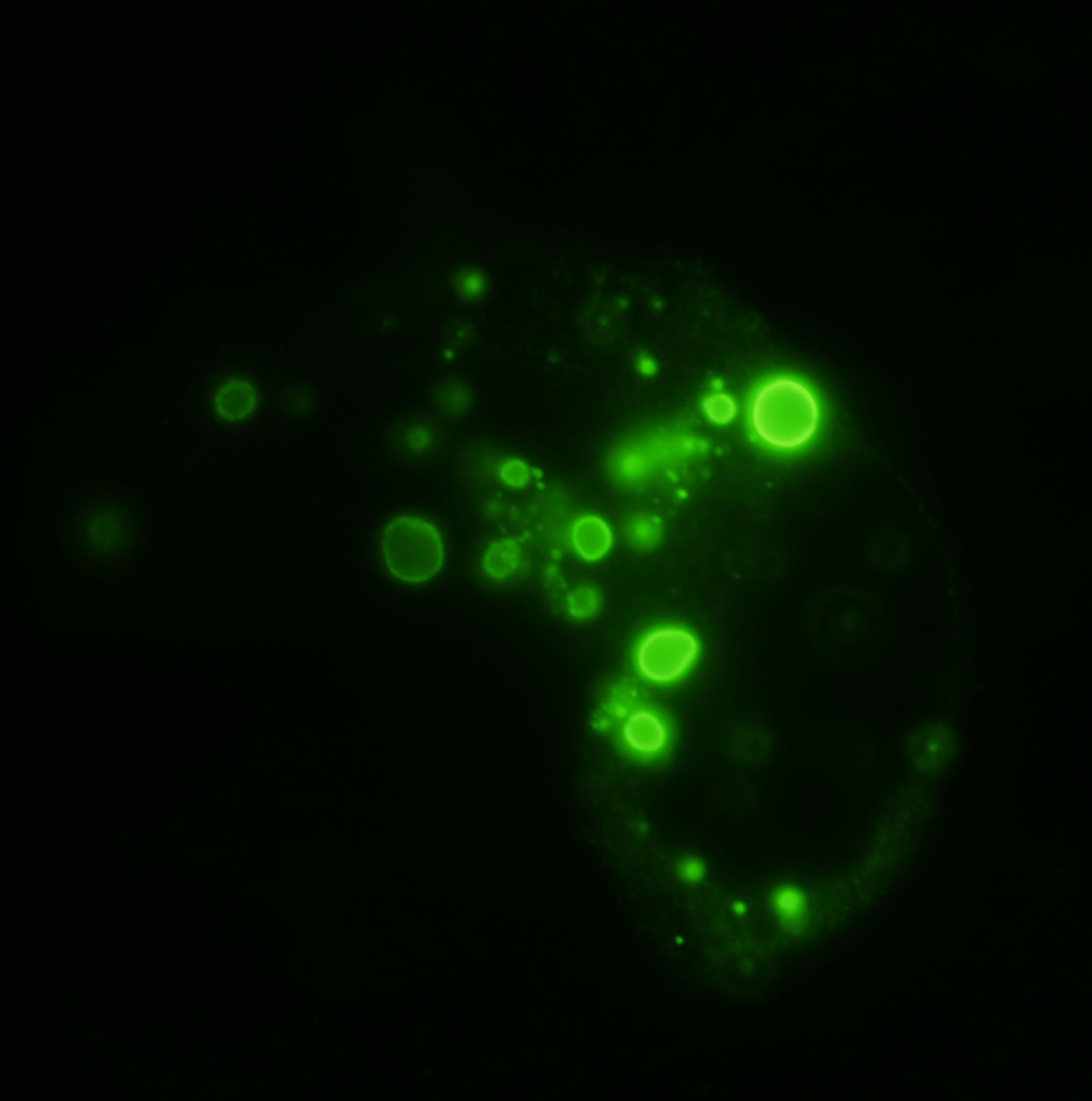

Supplement: Supplementary file 17 — Image files for Extended Data Fig. 5a–h. [file 41590_2024_1902_MOESM17_ESM.zip › ED Fig 5c Q333PTNIP+A20myc-tnip.jpg]

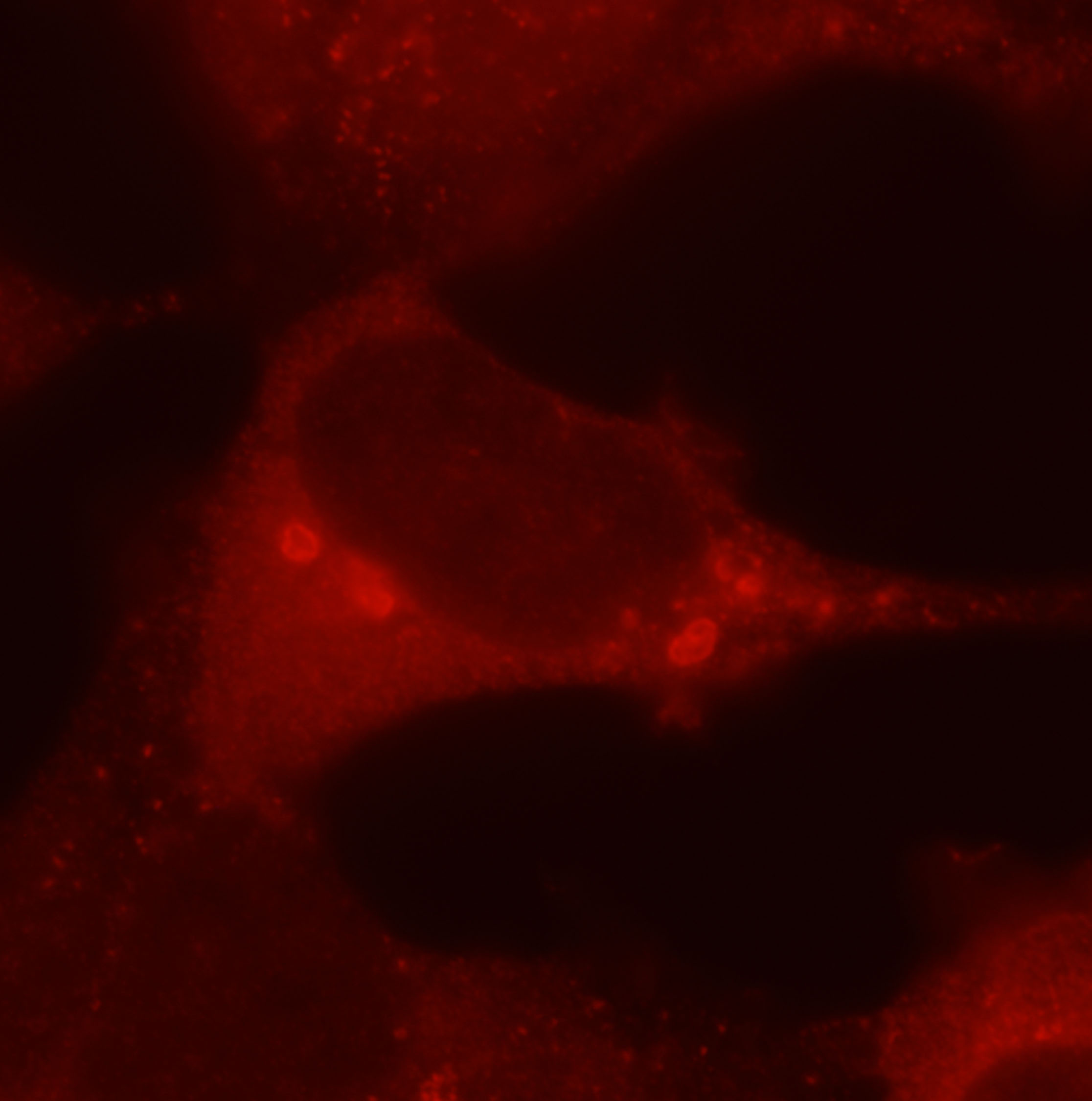

Supplement: Supplementary file 17 — Image files for Extended Data Fig. 5a–h. [file 41590_2024_1902_MOESM17_ESM.zip › ED Fig 5c WTTNIP+A20myc-a20.jpg]

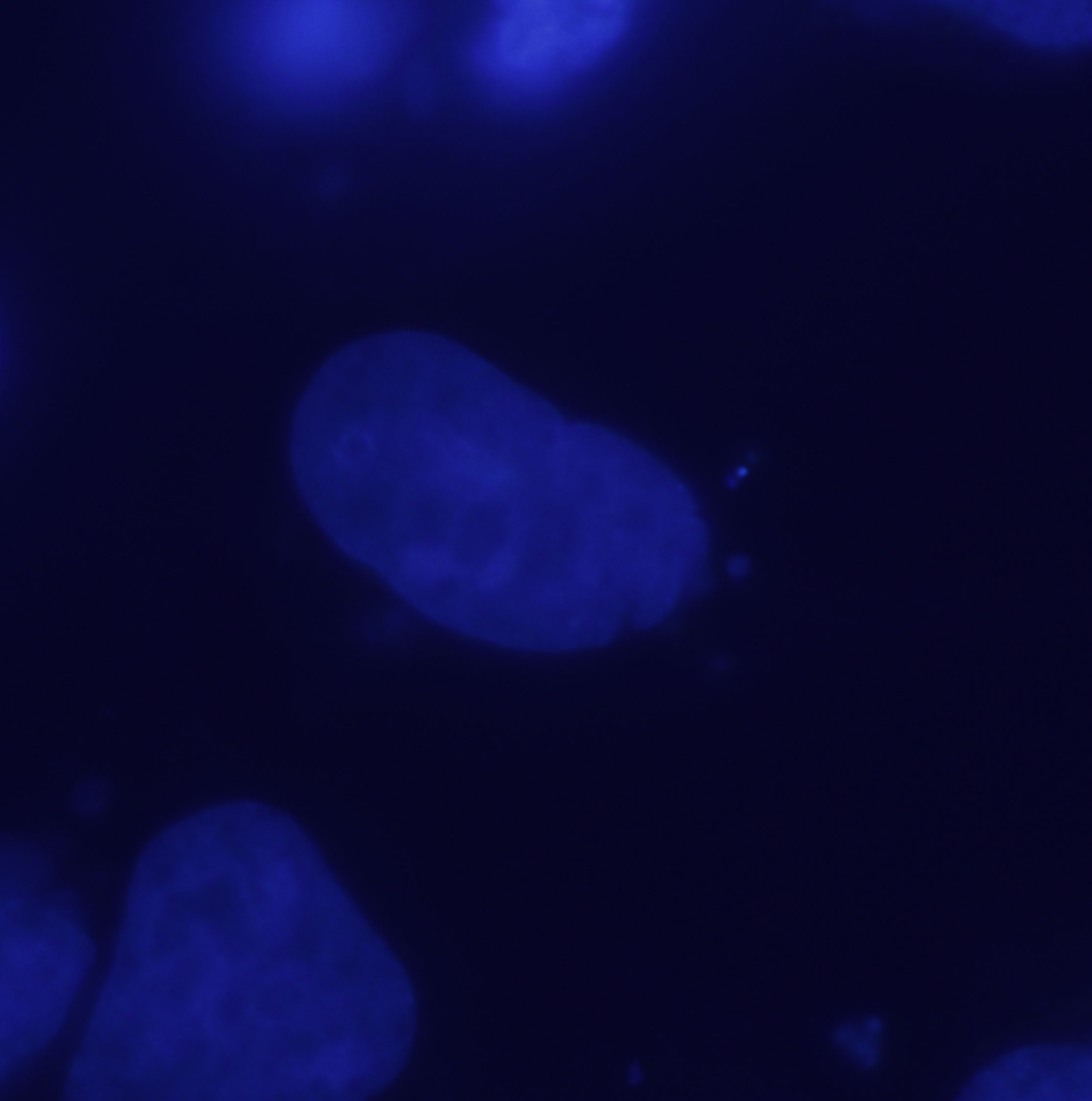

Supplement: Supplementary file 17 — Image files for Extended Data Fig. 5a–h. [file 41590_2024_1902_MOESM17_ESM.zip › ED Fig 5c WTTNIP+A20myc-dna.jpg]

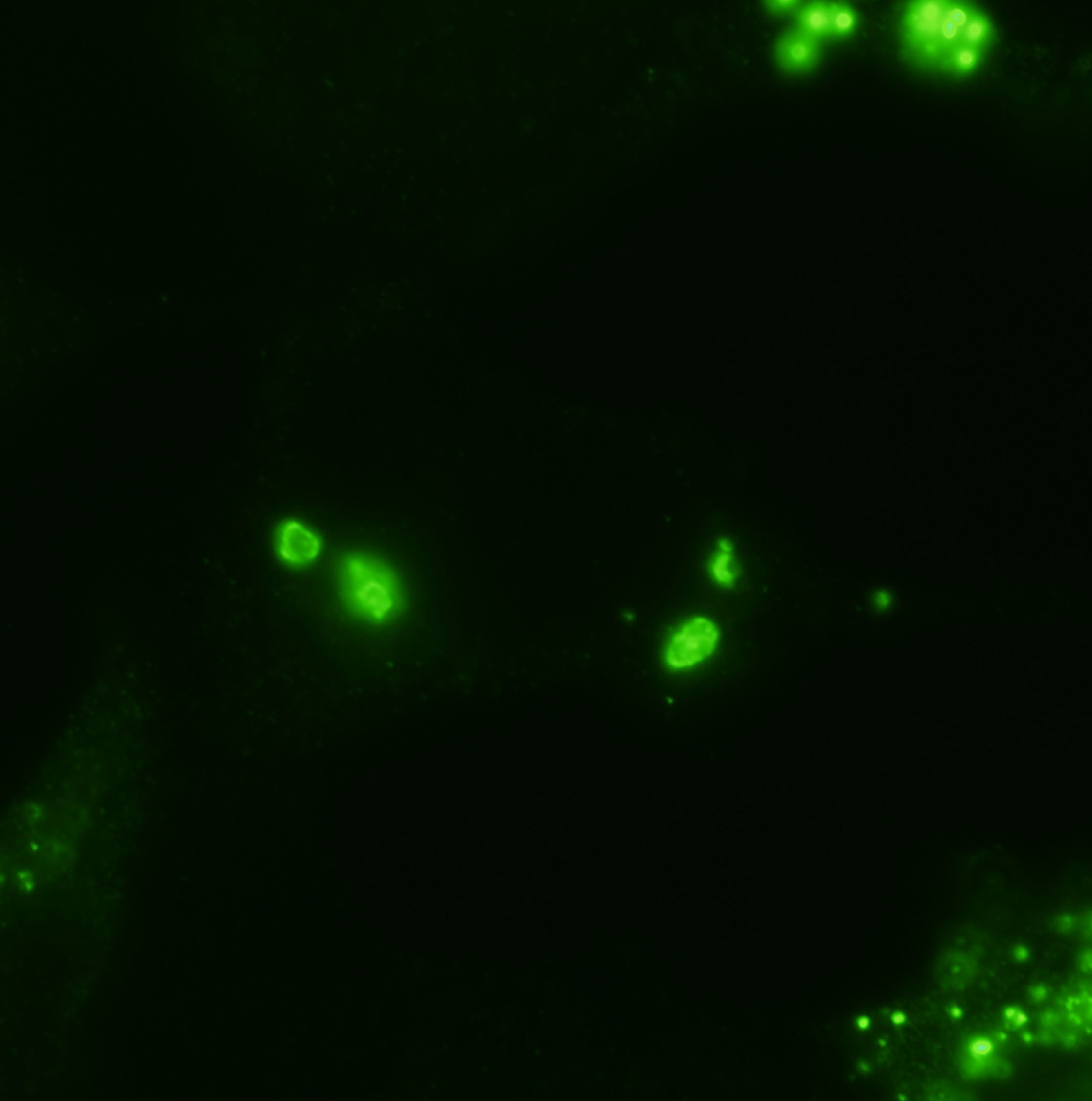

Supplement: Supplementary file 17 — Image files for Extended Data Fig. 5a–h. [file 41590_2024_1902_MOESM17_ESM.zip › ED Fig 5c WTTNIP+A20myc-tnip.jpg]

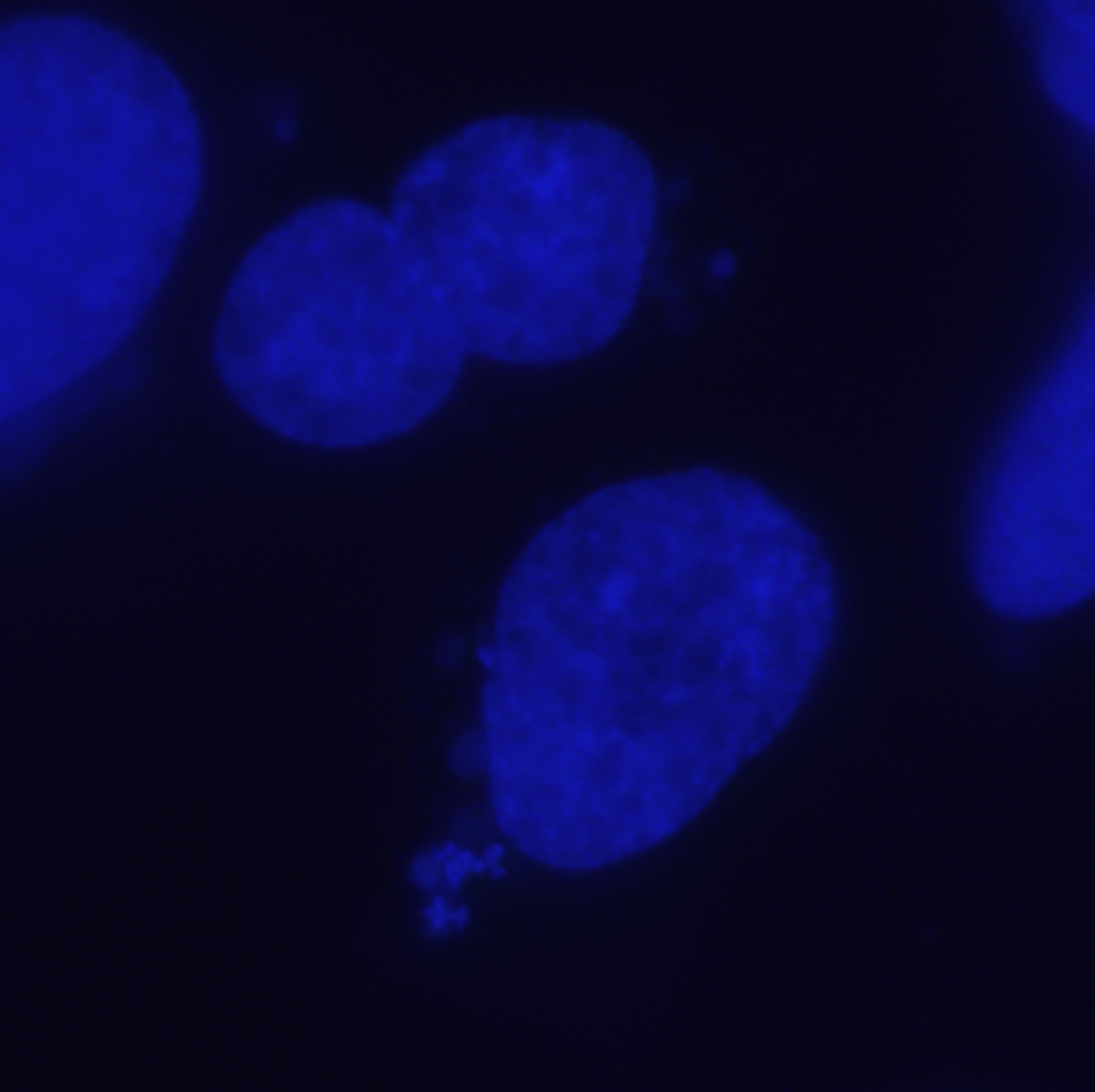

Supplement: Supplementary file 17 — Image files for Extended Data Fig. 5a–h. [file 41590_2024_1902_MOESM17_ESM.zip › ED Fig 5d TAX1BP1+Q333PTNIP-dna.jpg]

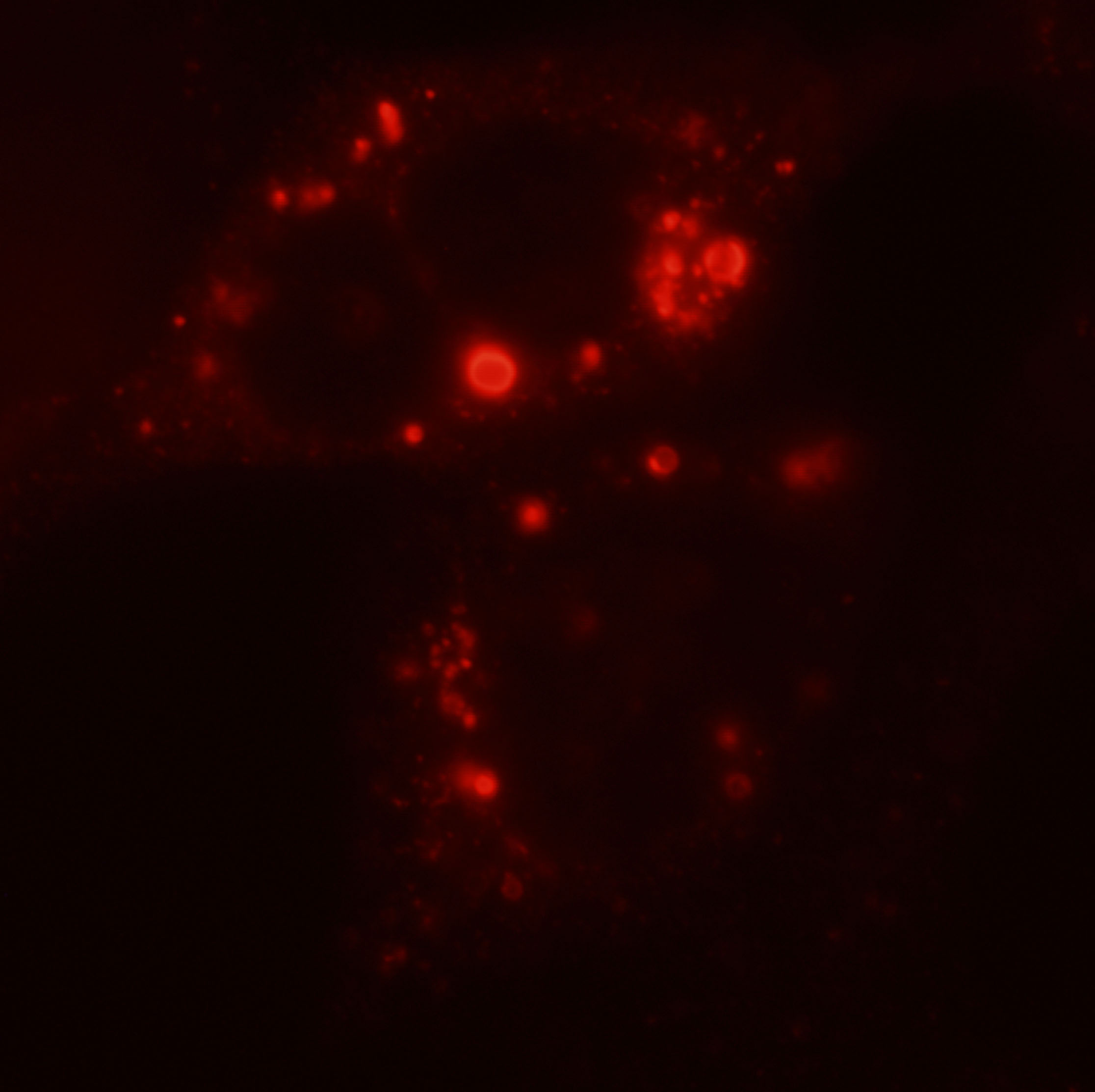

Supplement: Supplementary file 17 — Image files for Extended Data Fig. 5a–h. [file 41590_2024_1902_MOESM17_ESM.zip › ED Fig 5d TAX1BP1+Q333PTNIP-t6bp.jpg]

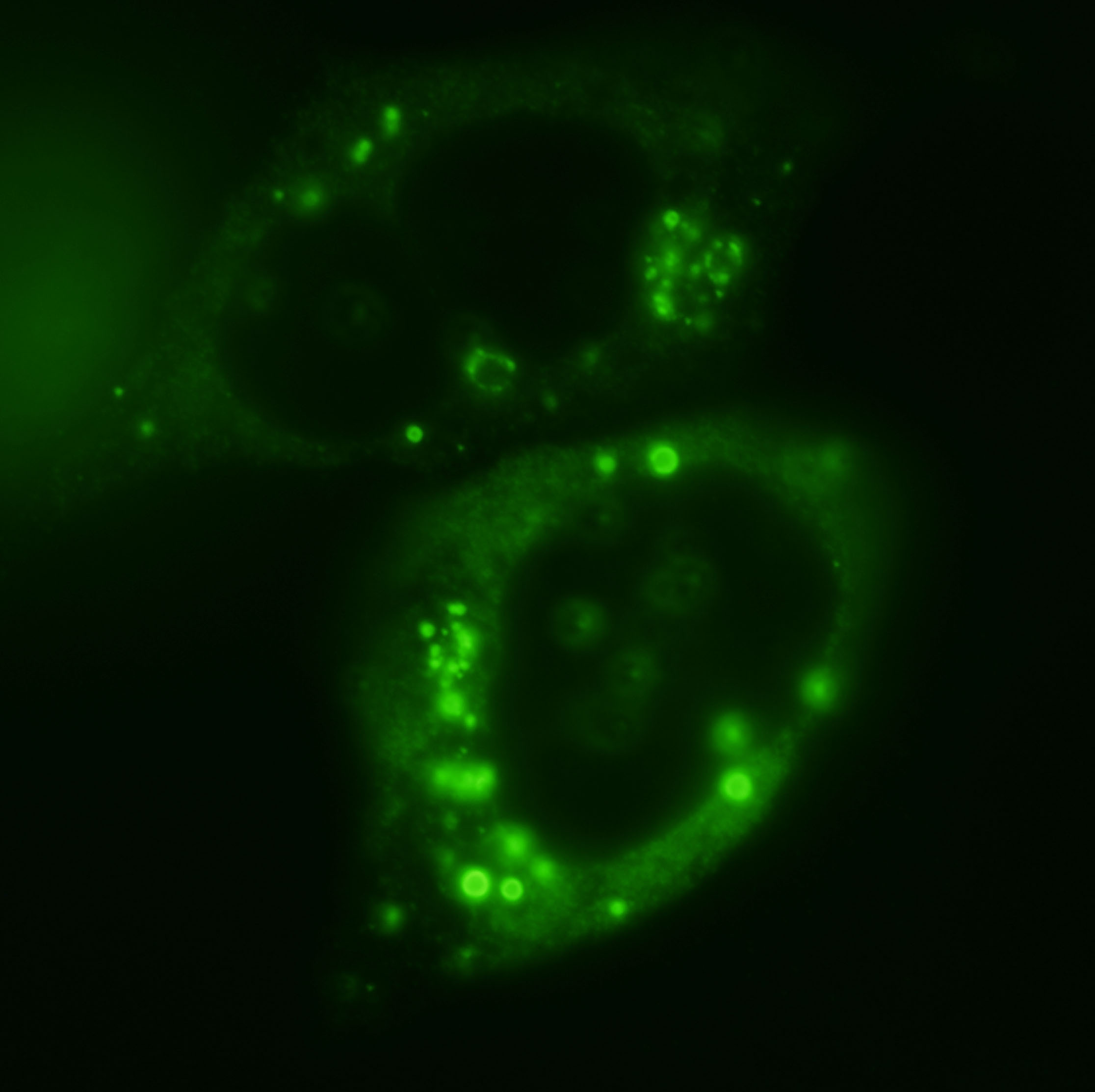

Supplement: Supplementary file 17 — Image files for Extended Data Fig. 5a–h. [file 41590_2024_1902_MOESM17_ESM.zip › ED Fig 5d TAX1BP1+Q333PTNIP-tnip.jpg]

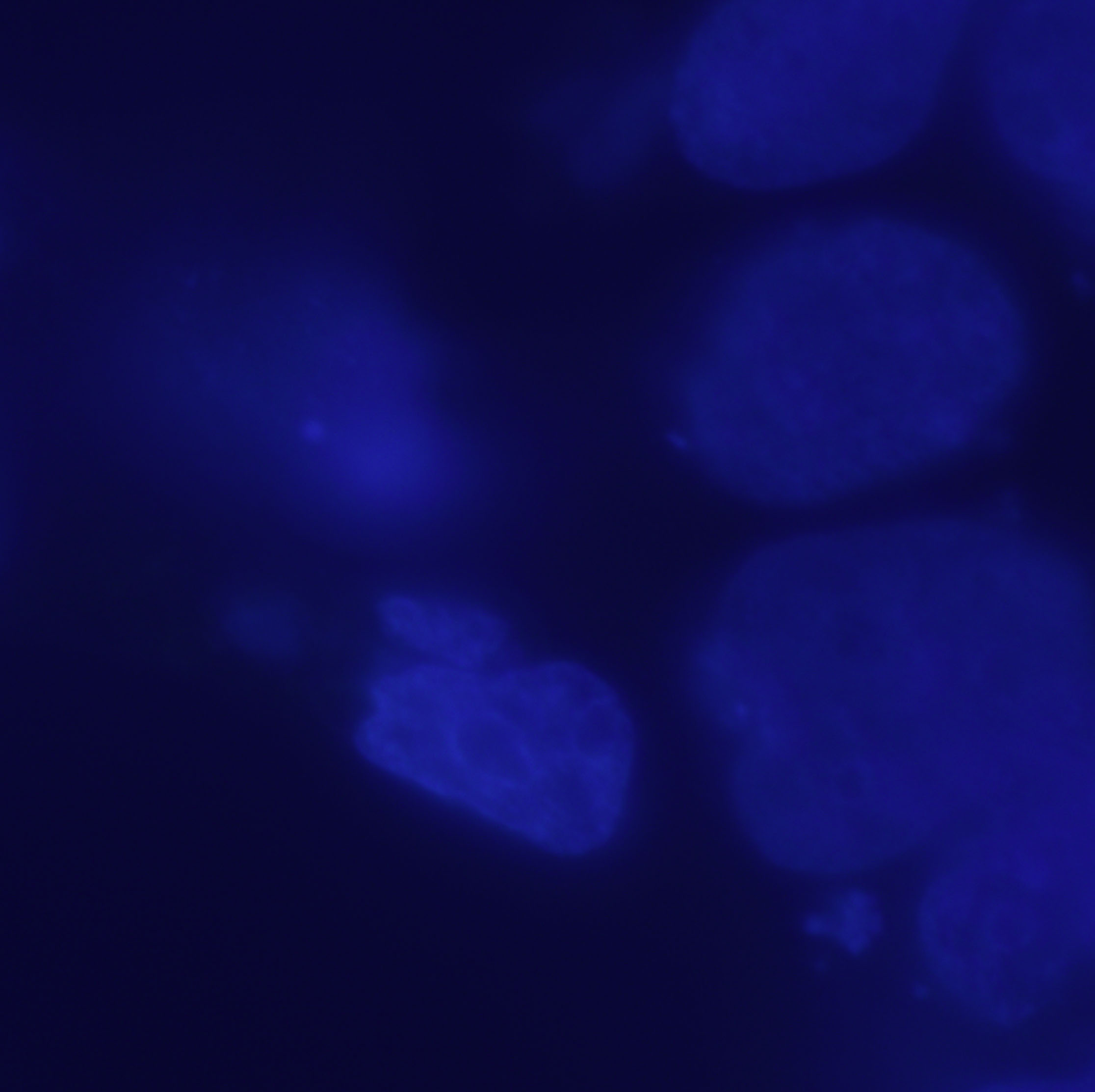

Supplement: Supplementary file 17 — Image files for Extended Data Fig. 5a–h. [file 41590_2024_1902_MOESM17_ESM.zip › Ed Fig 5d TAX1BP1+WTTNIP-dna.jpg]

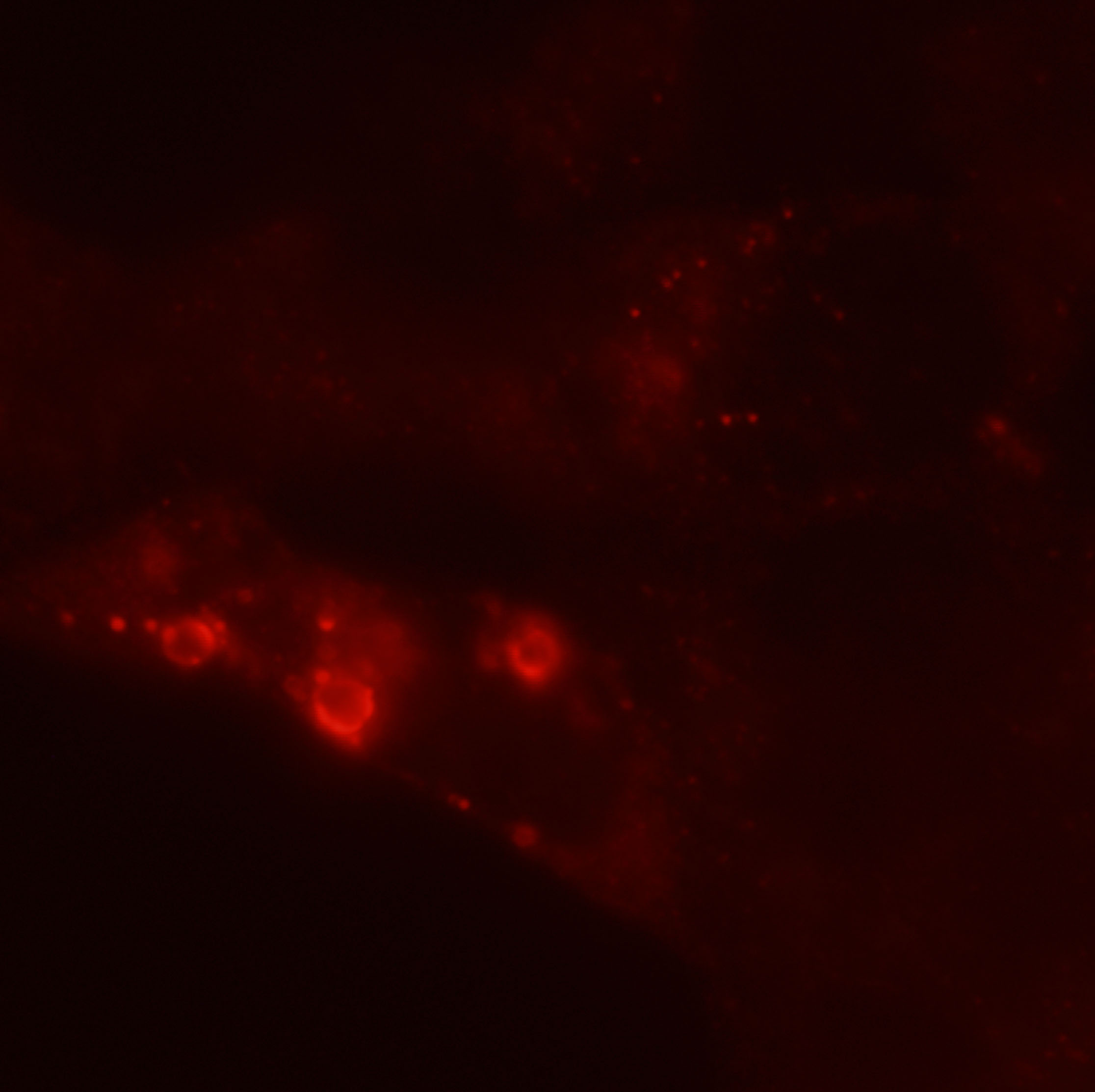

Supplement: Supplementary file 17 — Image files for Extended Data Fig. 5a–h. [file 41590_2024_1902_MOESM17_ESM.zip › Ed Fig 5d TAX1BP1+WTTNIP-t6bp.jpg]

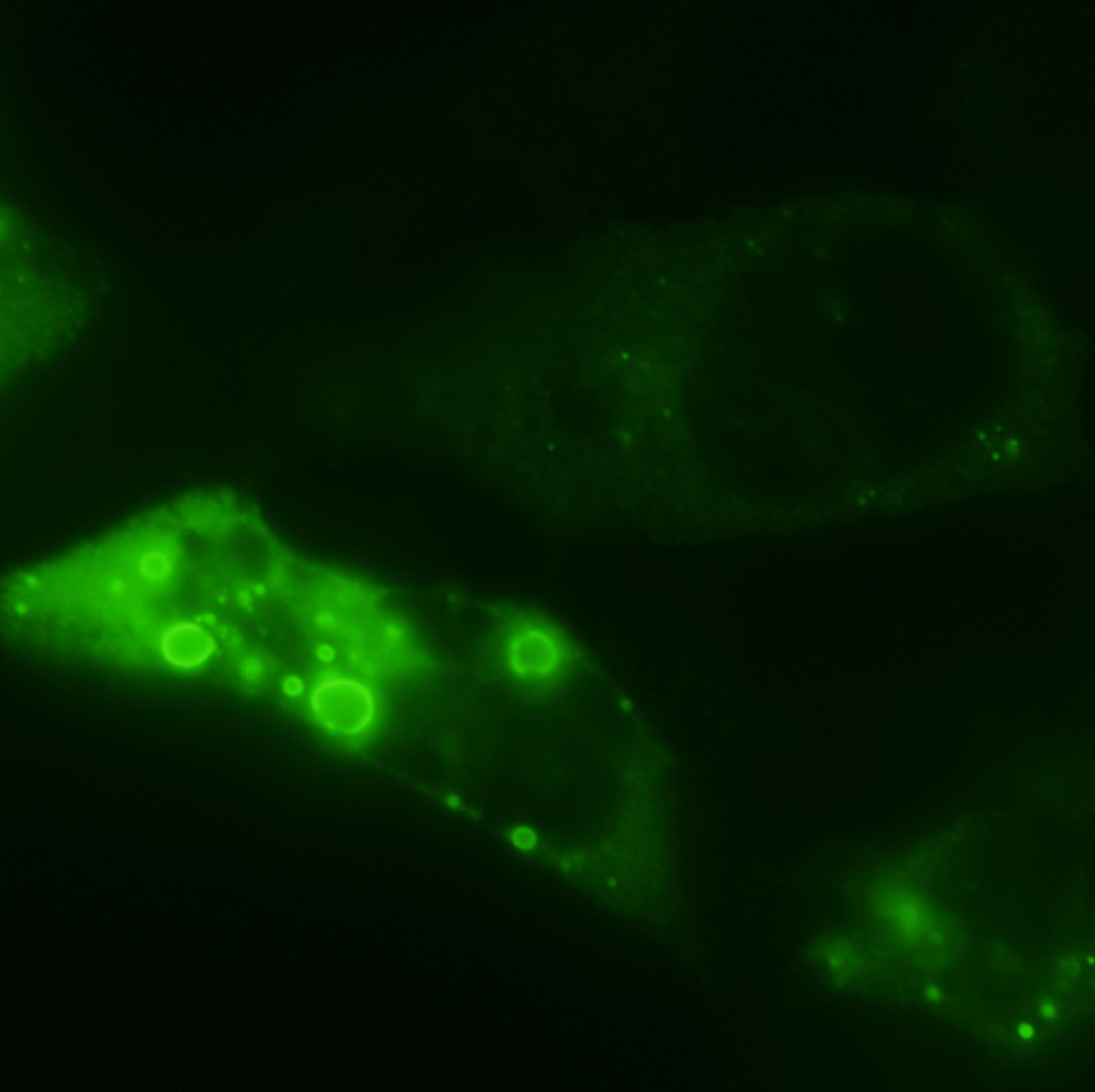

Supplement: Supplementary file 17 — Image files for Extended Data Fig. 5a–h. [file 41590_2024_1902_MOESM17_ESM.zip › Ed Fig 5d TAX1BP1+WTTNIP-tnip.jpg]

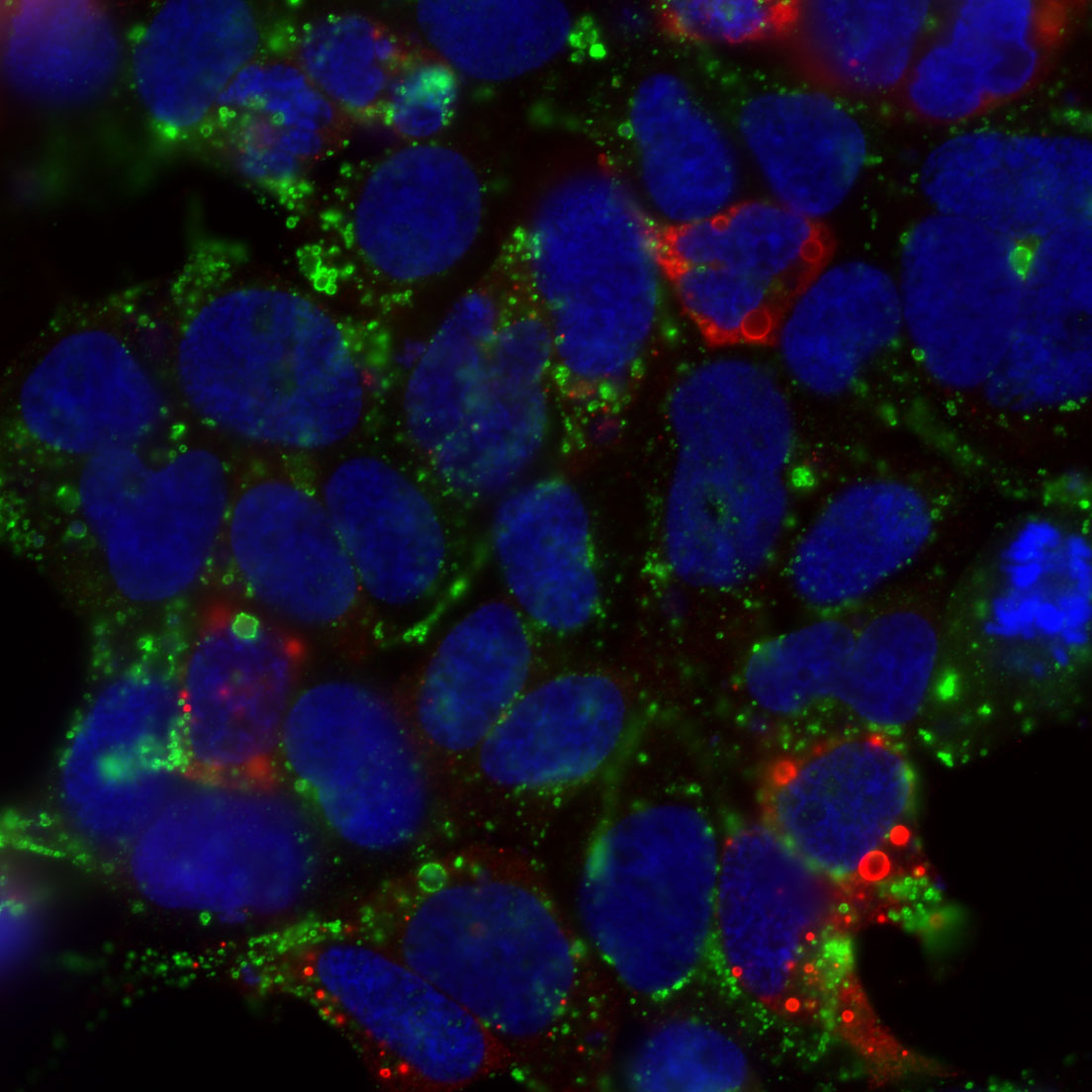

Supplement: Supplementary file 17 — Image files for Extended Data Fig. 5a–h. [file 41590_2024_1902_MOESM17_ESM.zip › ED Fig 5e tnip lamp1 q333p 5_overlay.jpg]

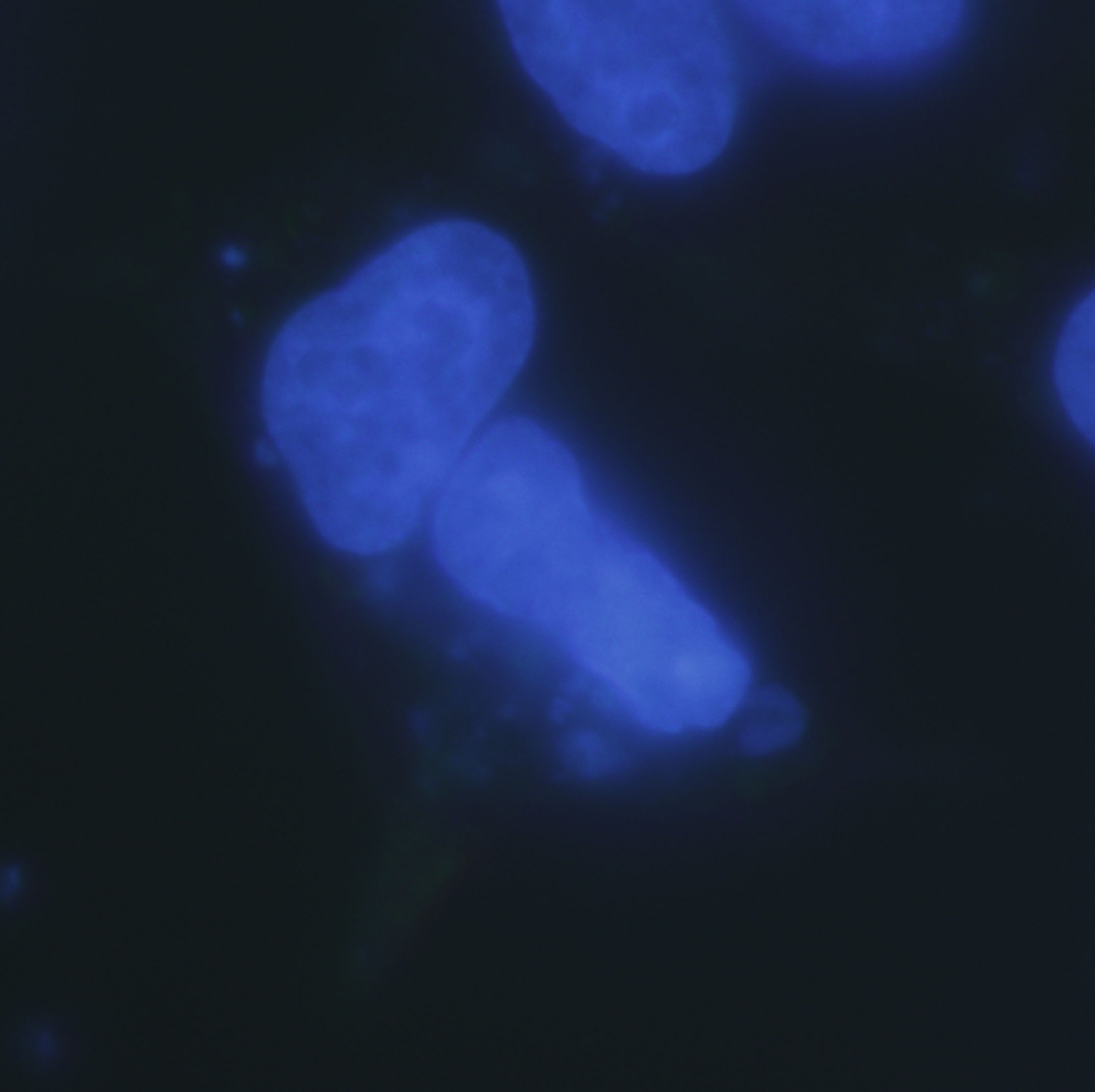

Supplement: Supplementary file 17 — Image files for Extended Data Fig. 5a–h. [file 41590_2024_1902_MOESM17_ESM.zip › ED Fig 5e TNIPWTRbLAMP1-dna.jpg]

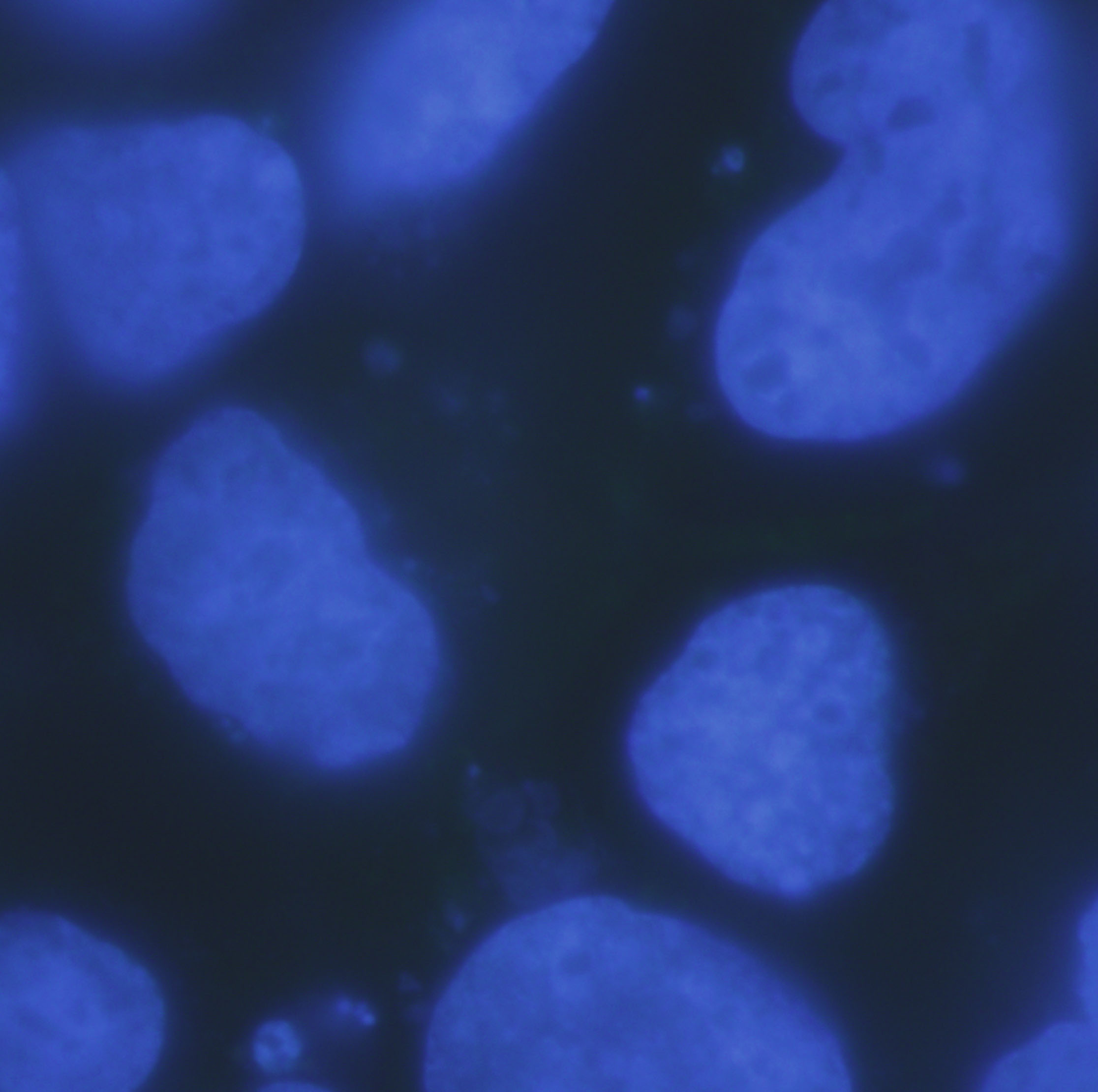

Supplement: Supplementary file 17 — Image files for Extended Data Fig. 5a–h. [file 41590_2024_1902_MOESM17_ESM.zip › ED Fig 5e TNIPWTRbLAMP1-dna2.jpg]

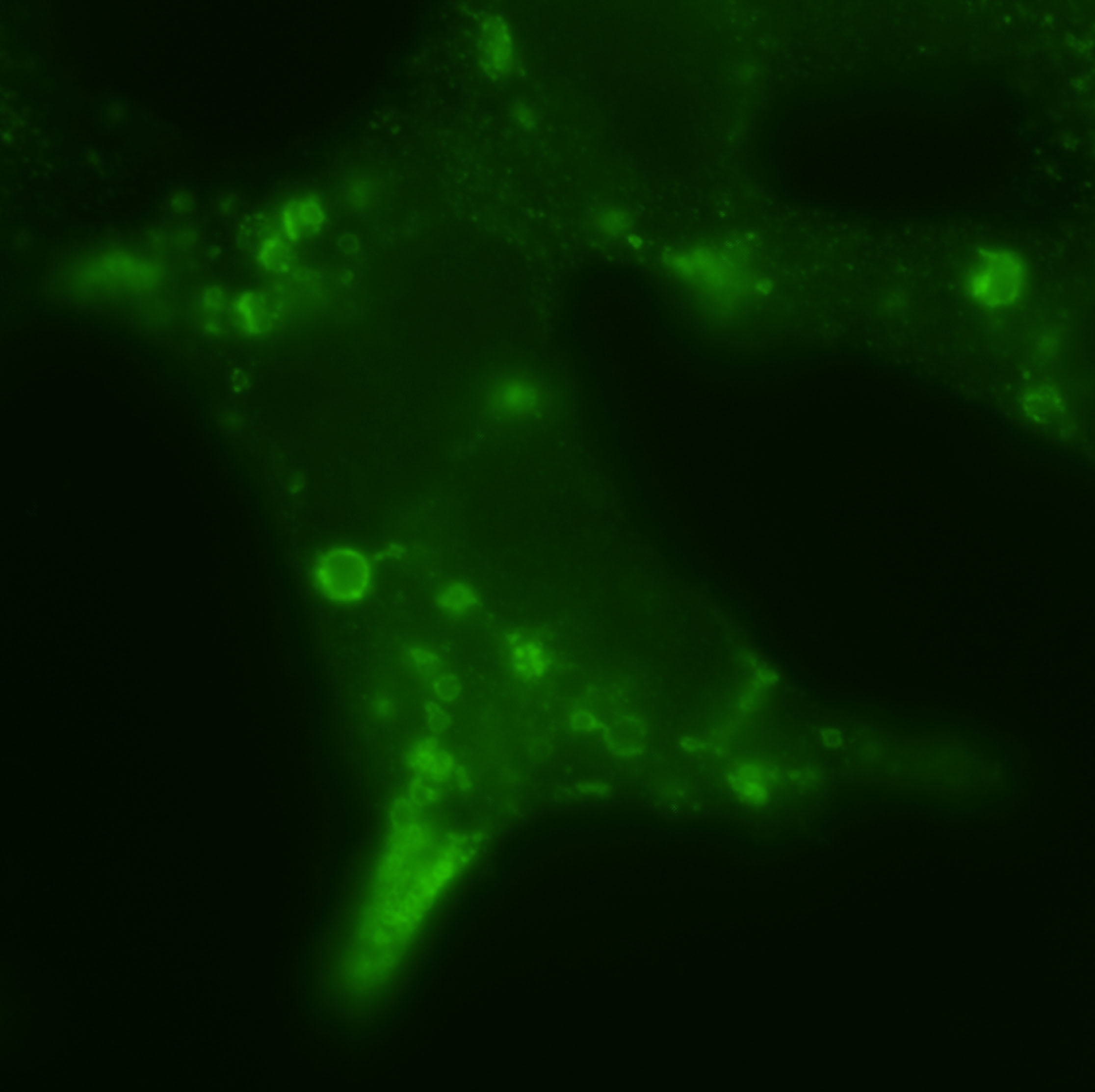

Supplement: Supplementary file 17 — Image files for Extended Data Fig. 5a–h. [file 41590_2024_1902_MOESM17_ESM.zip › ED Fig 5e TNIPWTRbLAMP1-lamp.jpg]

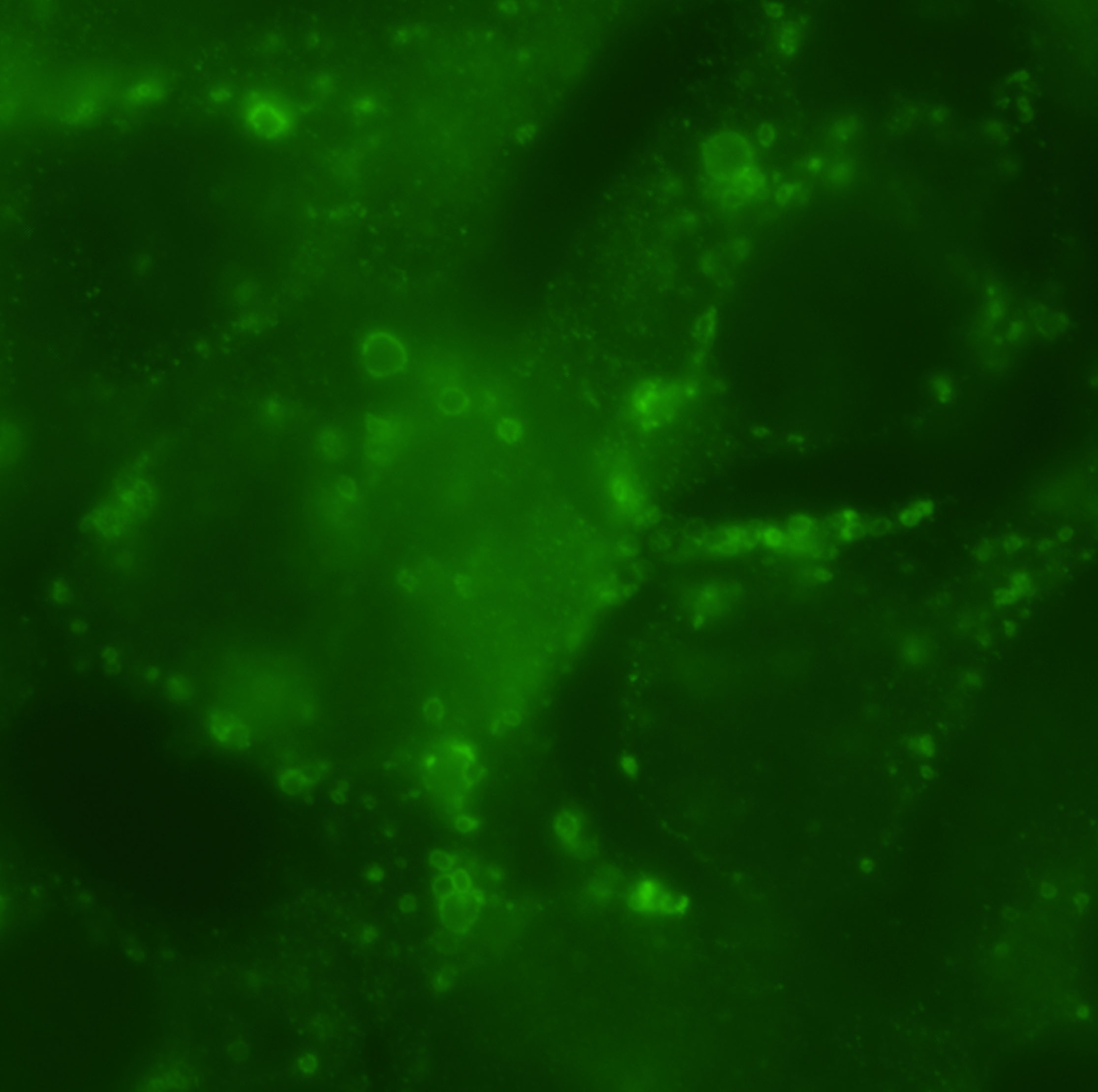

Supplement: Supplementary file 17 — Image files for Extended Data Fig. 5a–h. [file 41590_2024_1902_MOESM17_ESM.zip › ED Fig 5e TNIPWTRbLAMP1-lamp2.jpg]

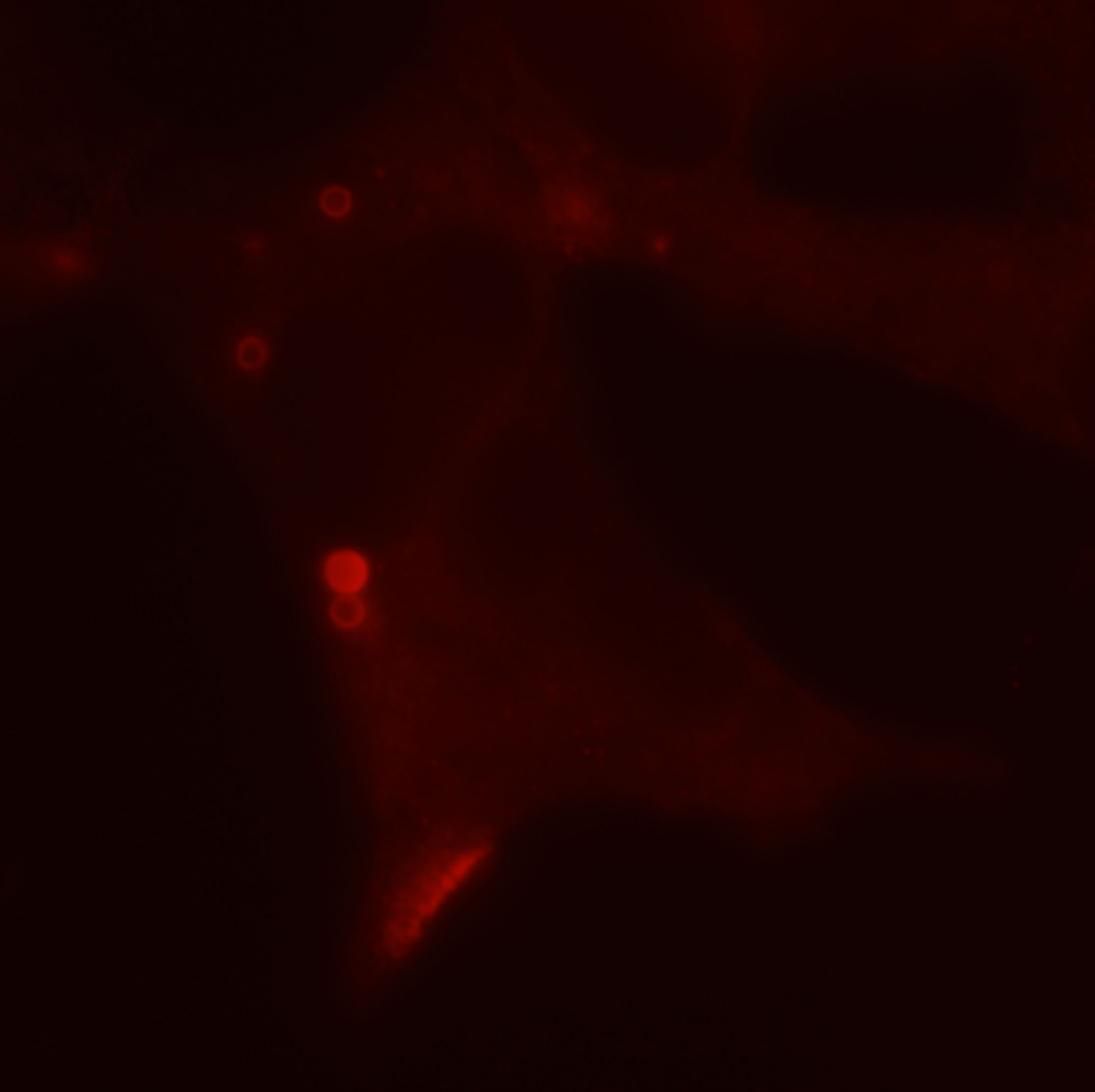

Supplement: Supplementary file 17 — Image files for Extended Data Fig. 5a–h. [file 41590_2024_1902_MOESM17_ESM.zip › ED Fig 5e TNIPWTRbLAMP1-tnip.jpg]

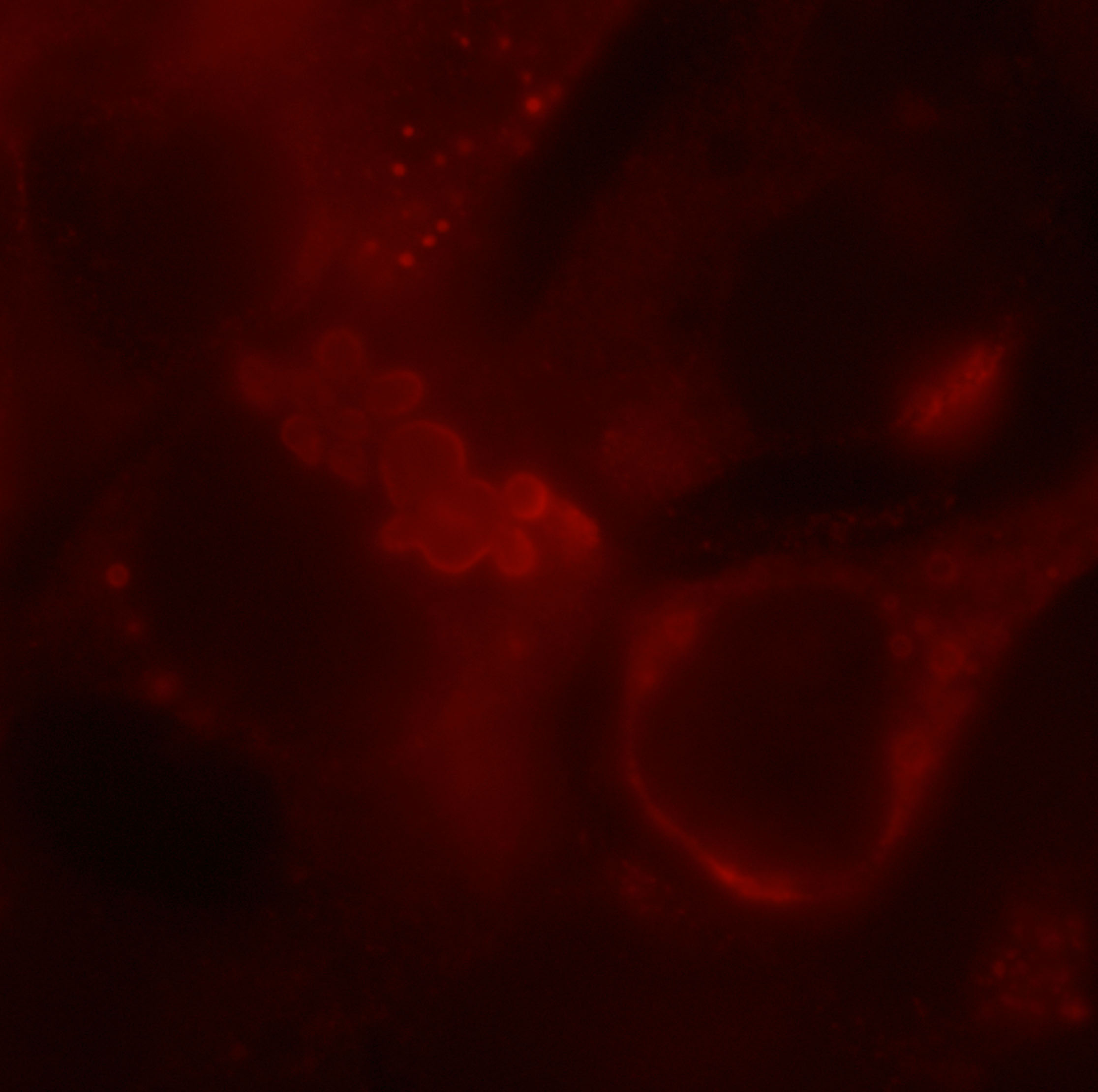

Supplement: Supplementary file 17 — Image files for Extended Data Fig. 5a–h. [file 41590_2024_1902_MOESM17_ESM.zip › ED Fig 5e TNIPWTRbLAMP1-tnip2.jpg]

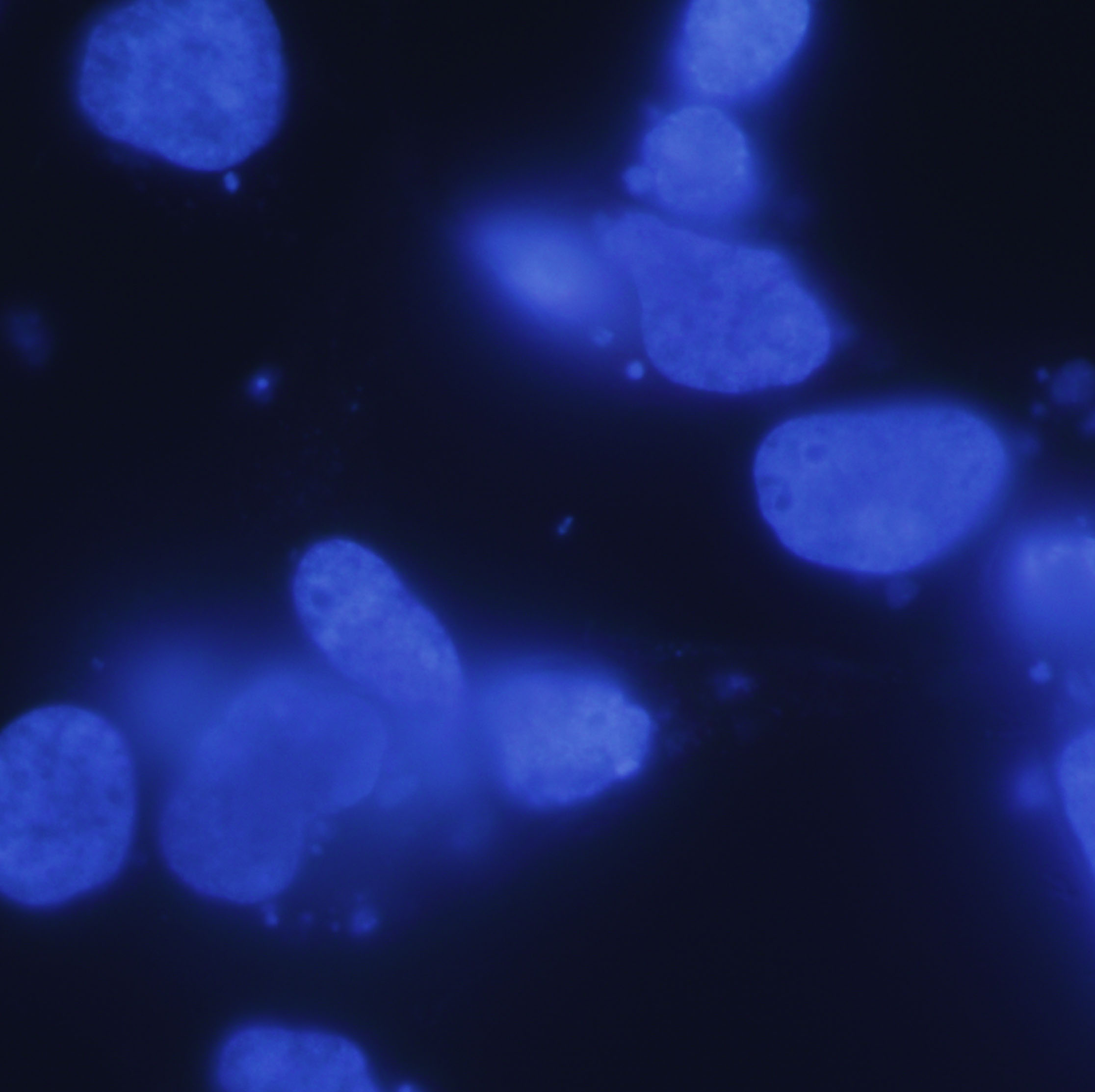

Supplement: Supplementary file 17 — Image files for Extended Data Fig. 5a–h. [file 41590_2024_1902_MOESM17_ESM.zip › ED Fig 5f Q333PTNIPeea1-dna.jpg]

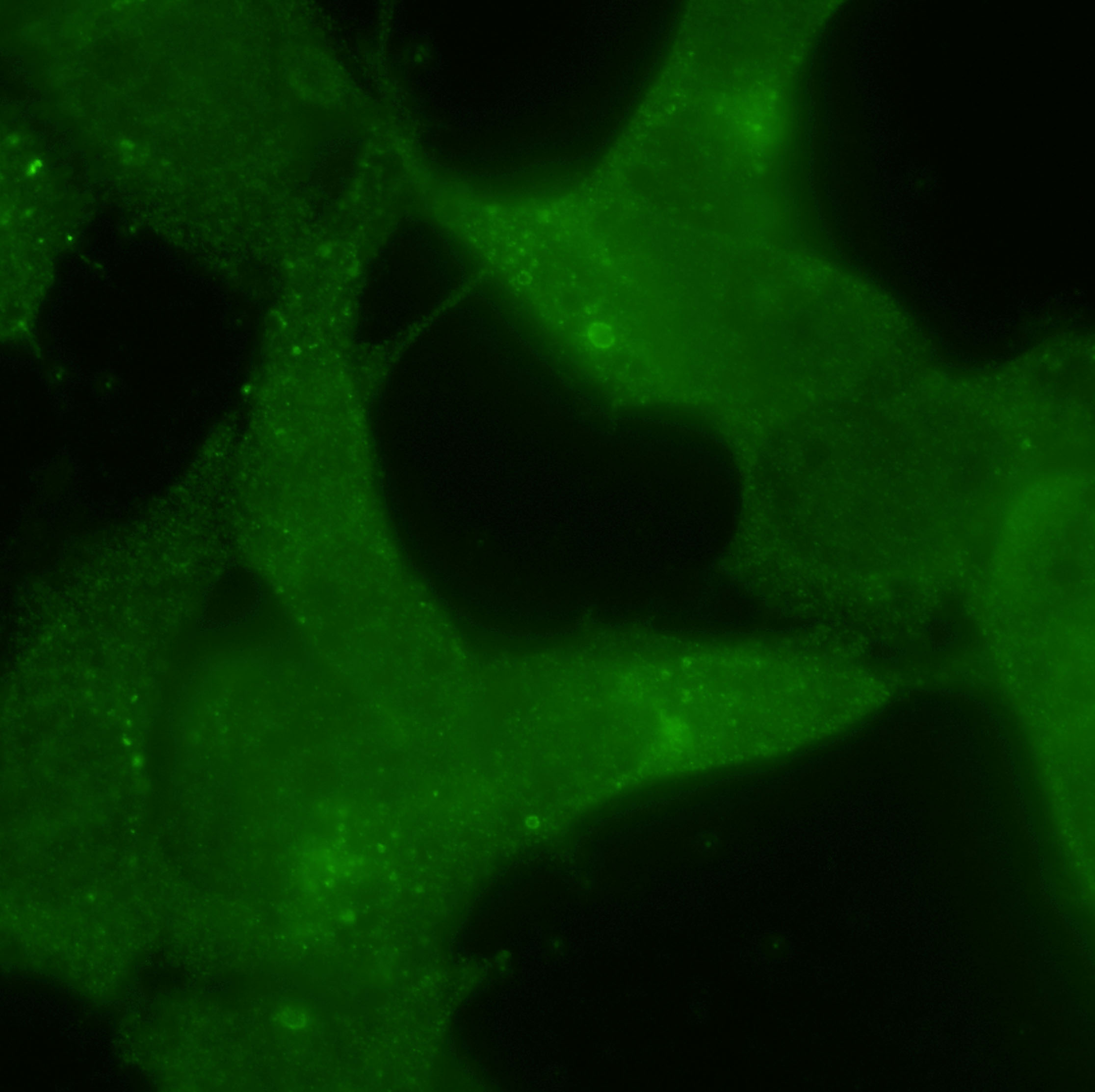

Supplement: Supplementary file 17 — Image files for Extended Data Fig. 5a–h. [file 41590_2024_1902_MOESM17_ESM.zip › ED Fig 5f Q333PTNIPeea1-eea1.jpg]

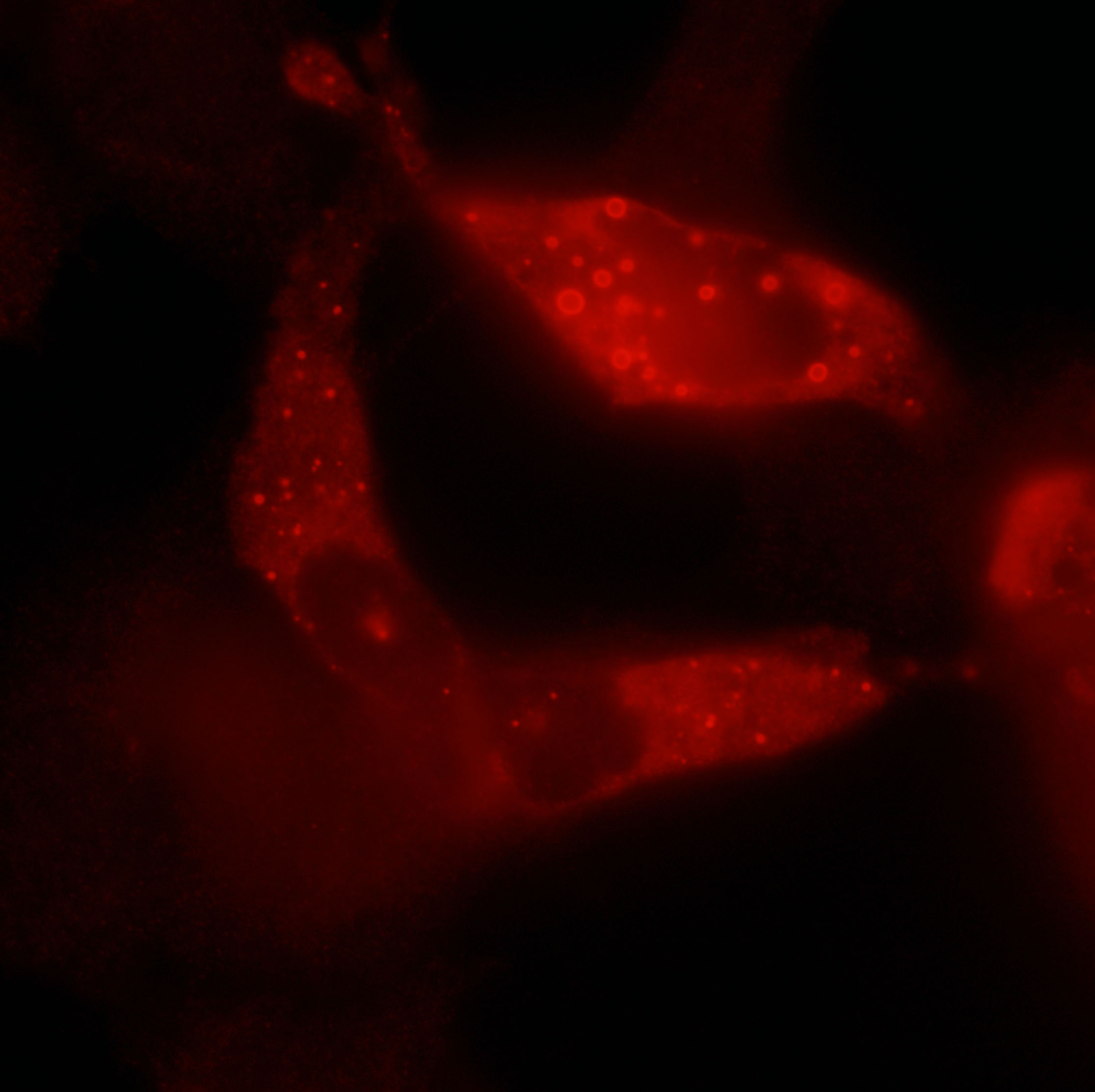

Supplement: Supplementary file 17 — Image files for Extended Data Fig. 5a–h. [file 41590_2024_1902_MOESM17_ESM.zip › ED Fig 5f Q333PTNIPeea1-tnip1.jpg]

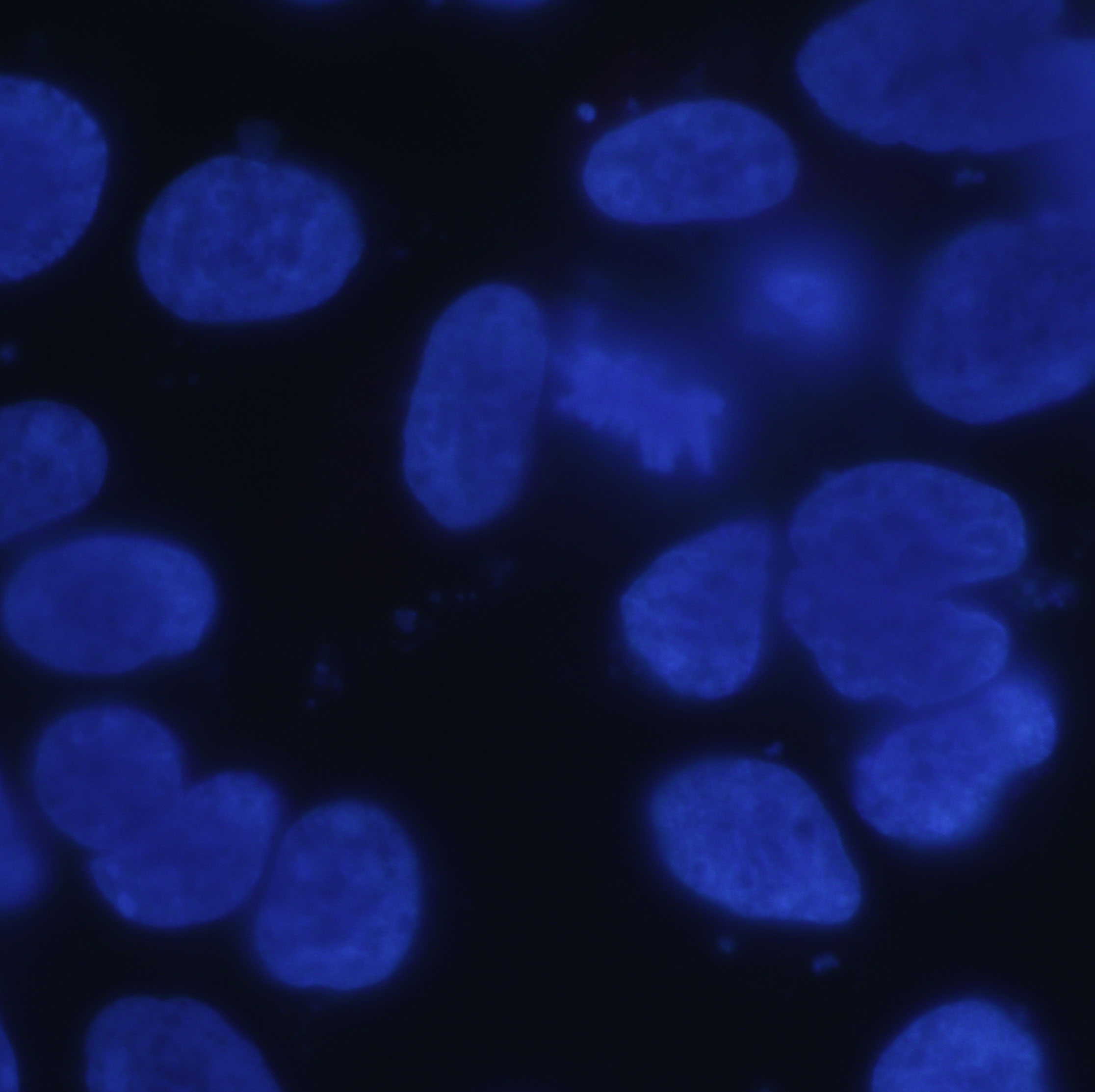

Supplement: Supplementary file 17 — Image files for Extended Data Fig. 5a–h. [file 41590_2024_1902_MOESM17_ESM.zip › ED Fig 5f WTTNIPeea1-dna.jpg]

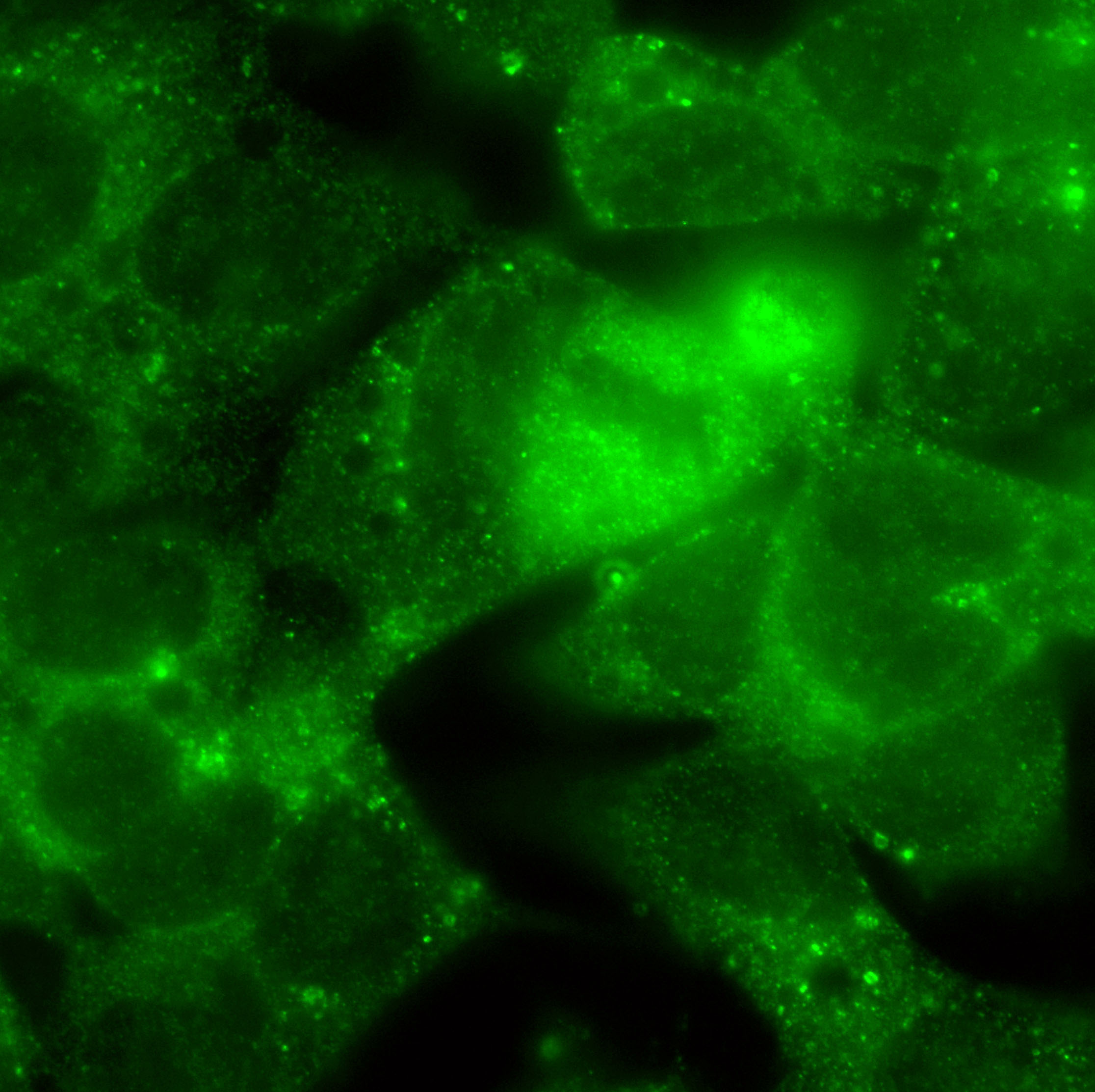

Supplement: Supplementary file 17 — Image files for Extended Data Fig. 5a–h. [file 41590_2024_1902_MOESM17_ESM.zip › ED Fig 5f WTTNIPeea1-eea.jpg]

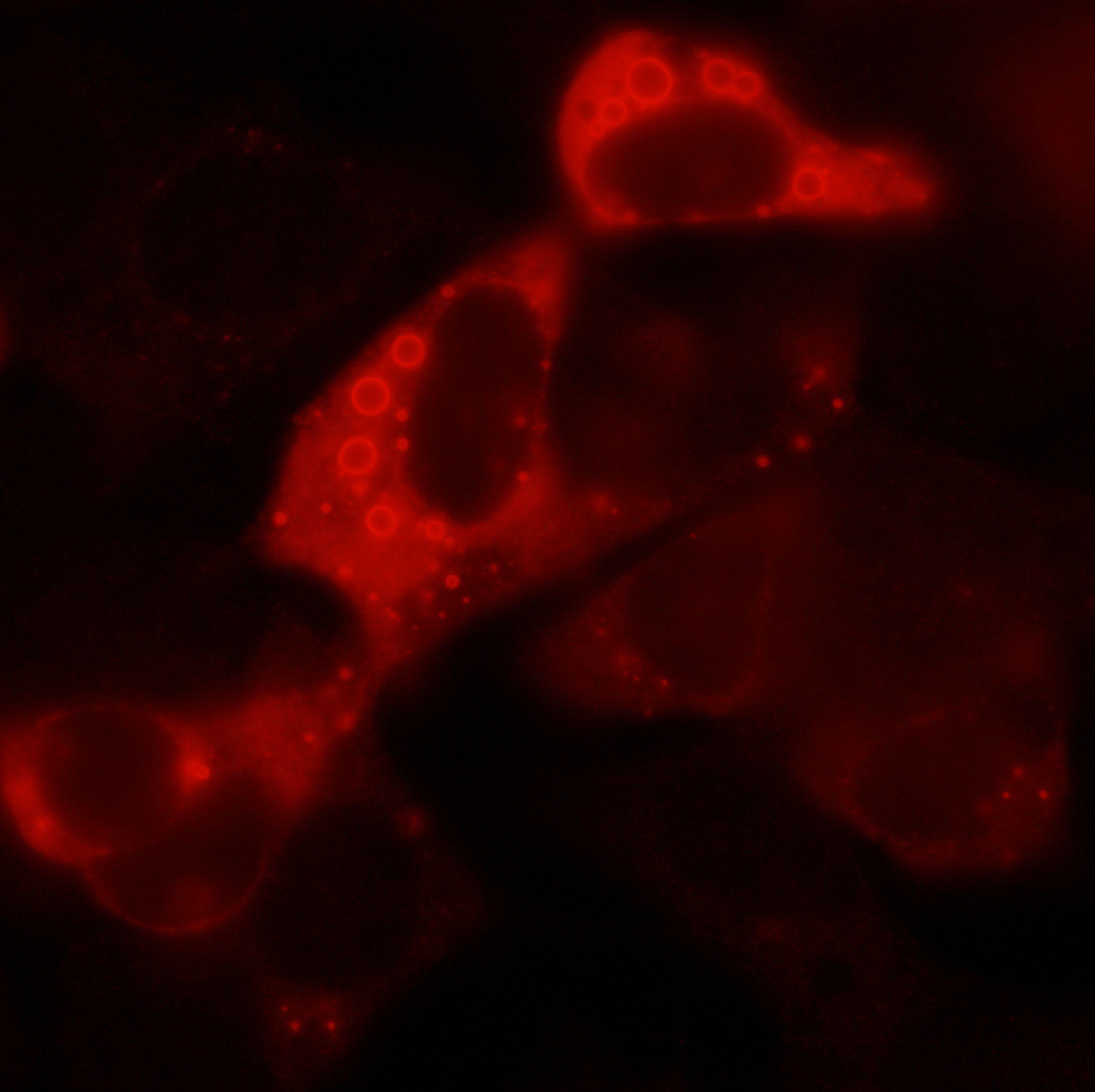

Supplement: Supplementary file 17 — Image files for Extended Data Fig. 5a–h. [file 41590_2024_1902_MOESM17_ESM.zip › ED Fig 5f WTTNIPeea1-tnip.jpg]

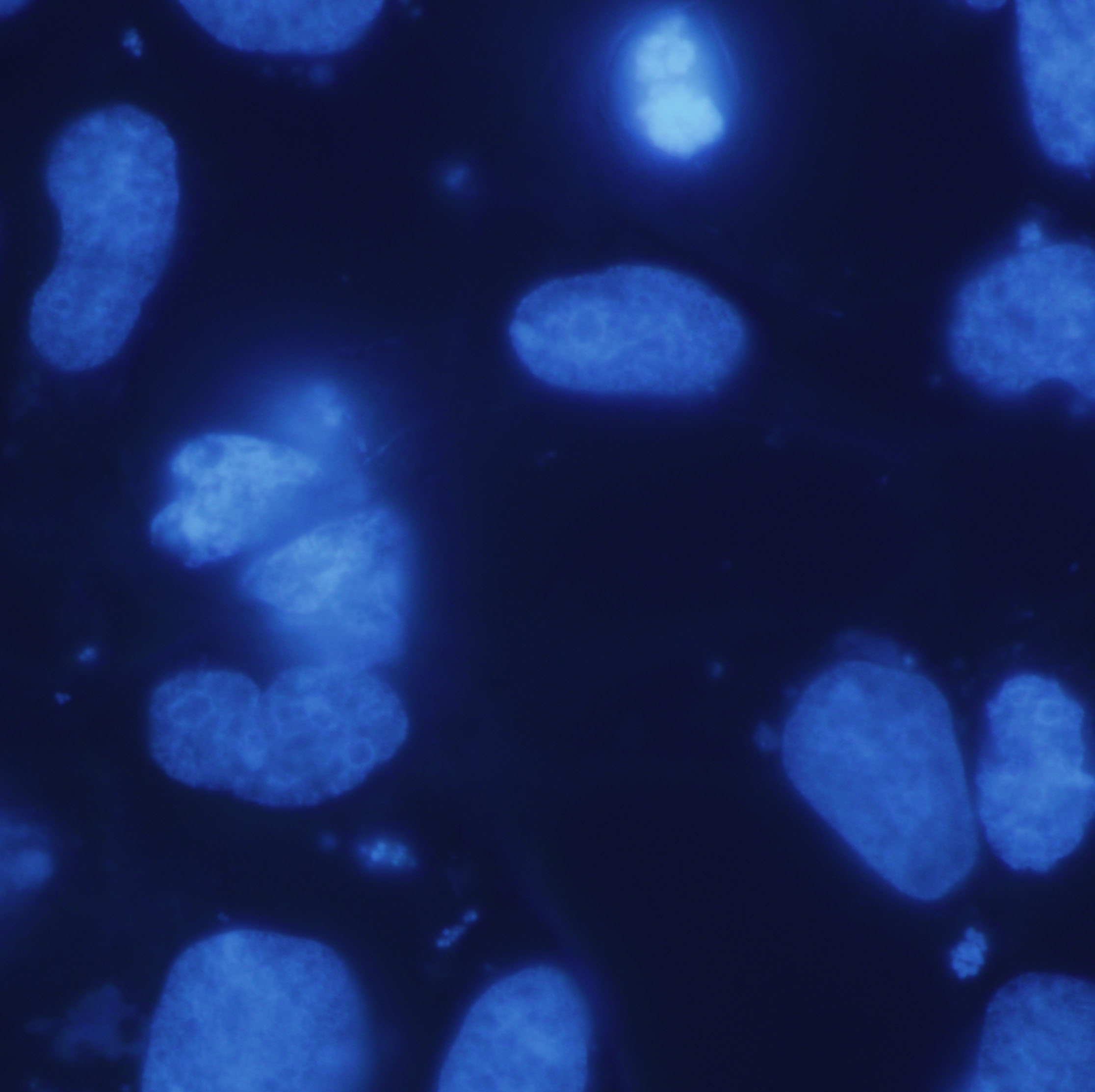

Supplement: Supplementary file 17 — Image files for Extended Data Fig. 5a–h. [file 41590_2024_1902_MOESM17_ESM.zip › ED Fig 5g Q333PTNIPrab7-dna.jpg]

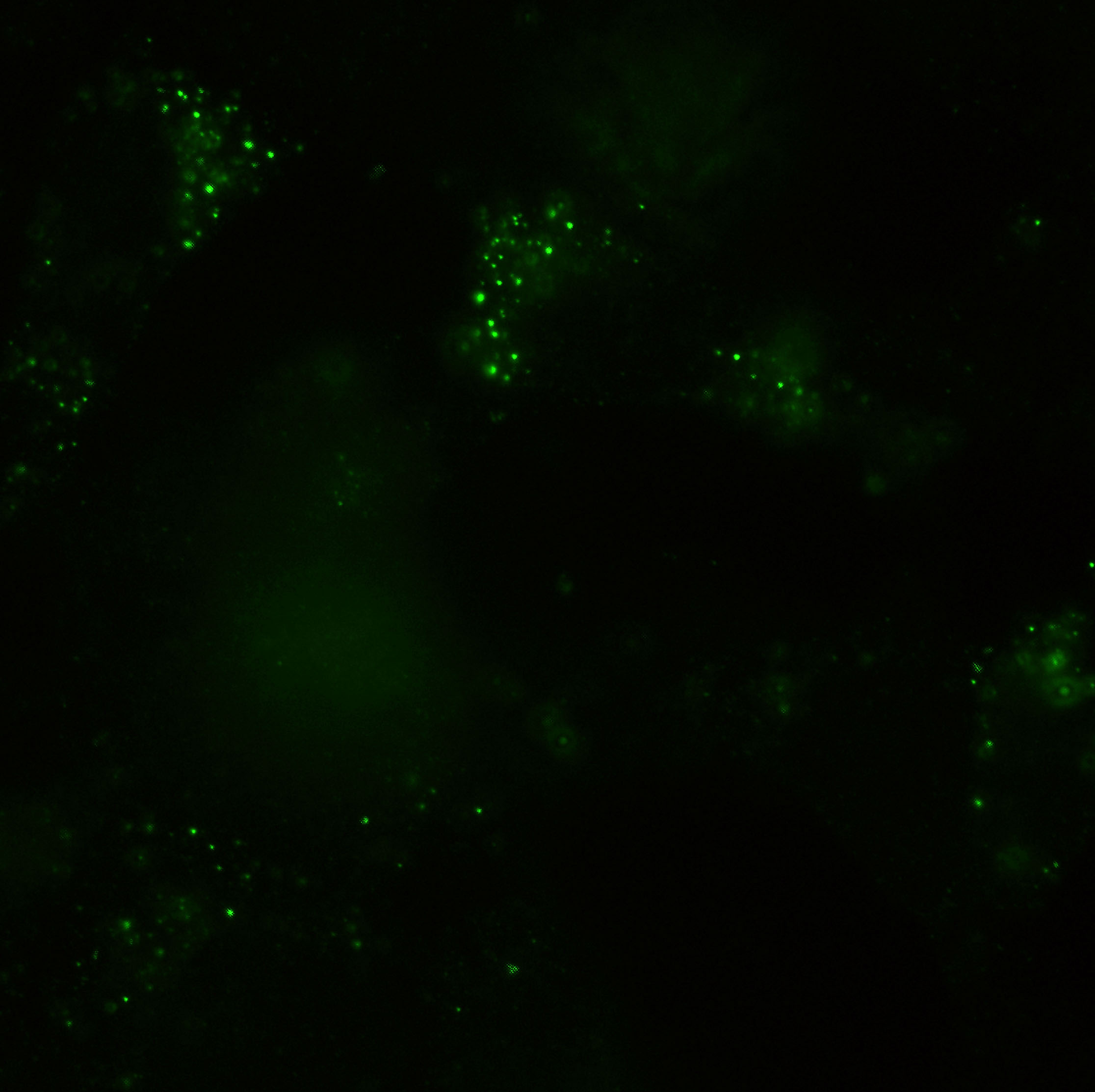

Supplement: Supplementary file 17 — Image files for Extended Data Fig. 5a–h. [file 41590_2024_1902_MOESM17_ESM.zip › ED Fig 5g Q333PTNIPrab7-rab.jpg]

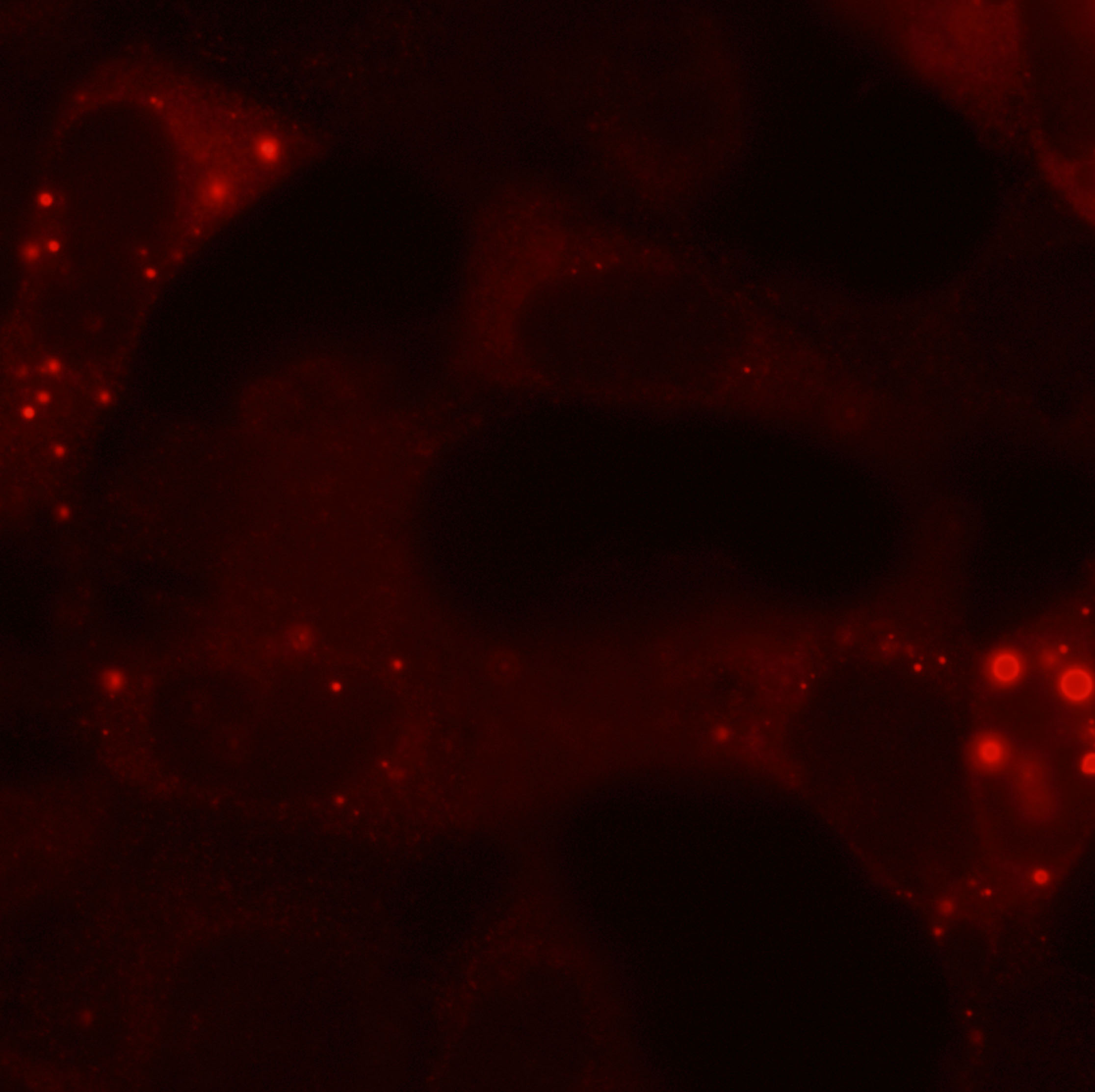

Supplement: Supplementary file 17 — Image files for Extended Data Fig. 5a–h. [file 41590_2024_1902_MOESM17_ESM.zip › ED Fig 5g Q333PTNIPrab7-tnip.jpg]

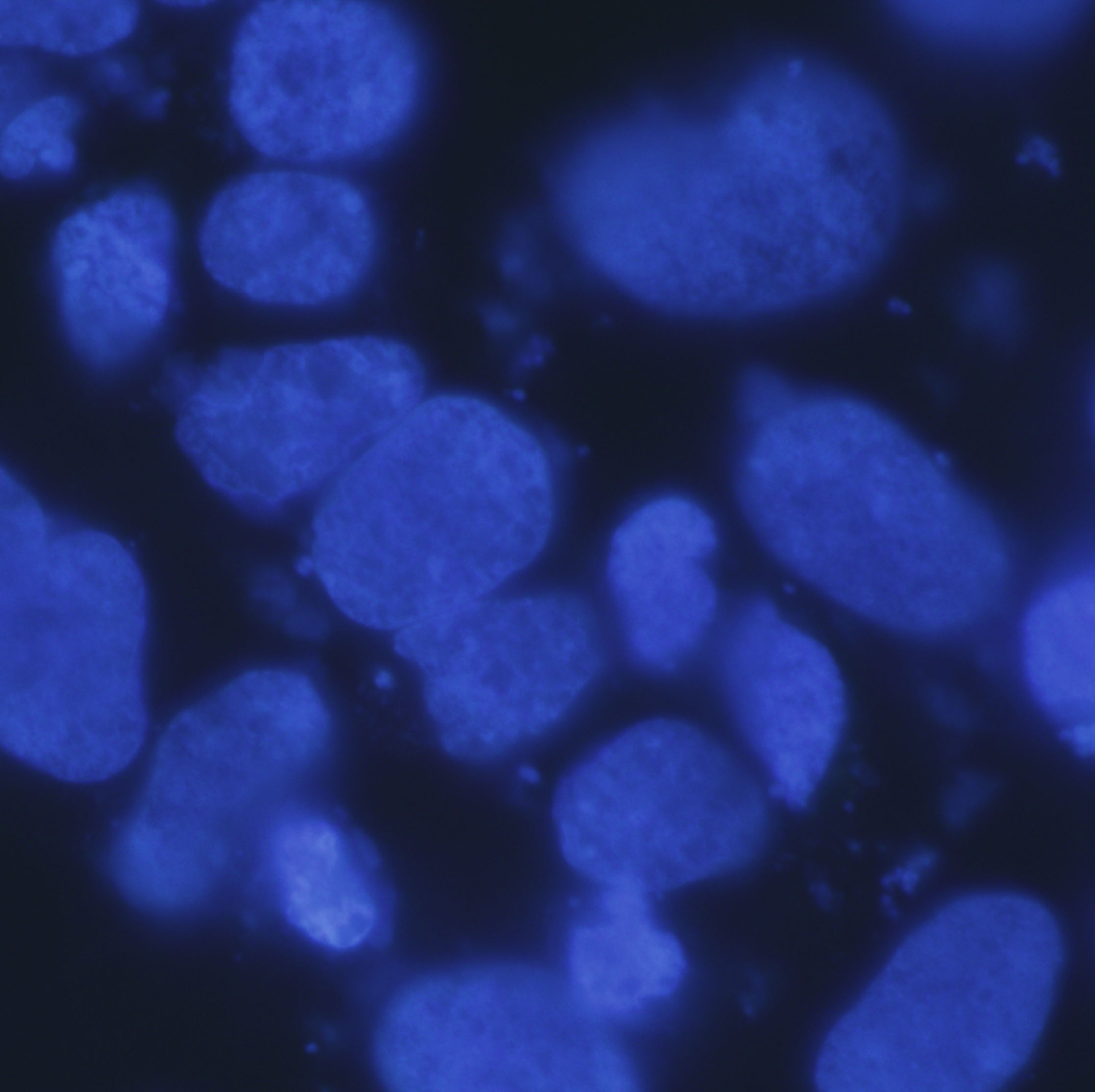

Supplement: Supplementary file 17 — Image files for Extended Data Fig. 5a–h. [file 41590_2024_1902_MOESM17_ESM.zip › ED Fig 5g WTTNIPrab7-dna.jpg]

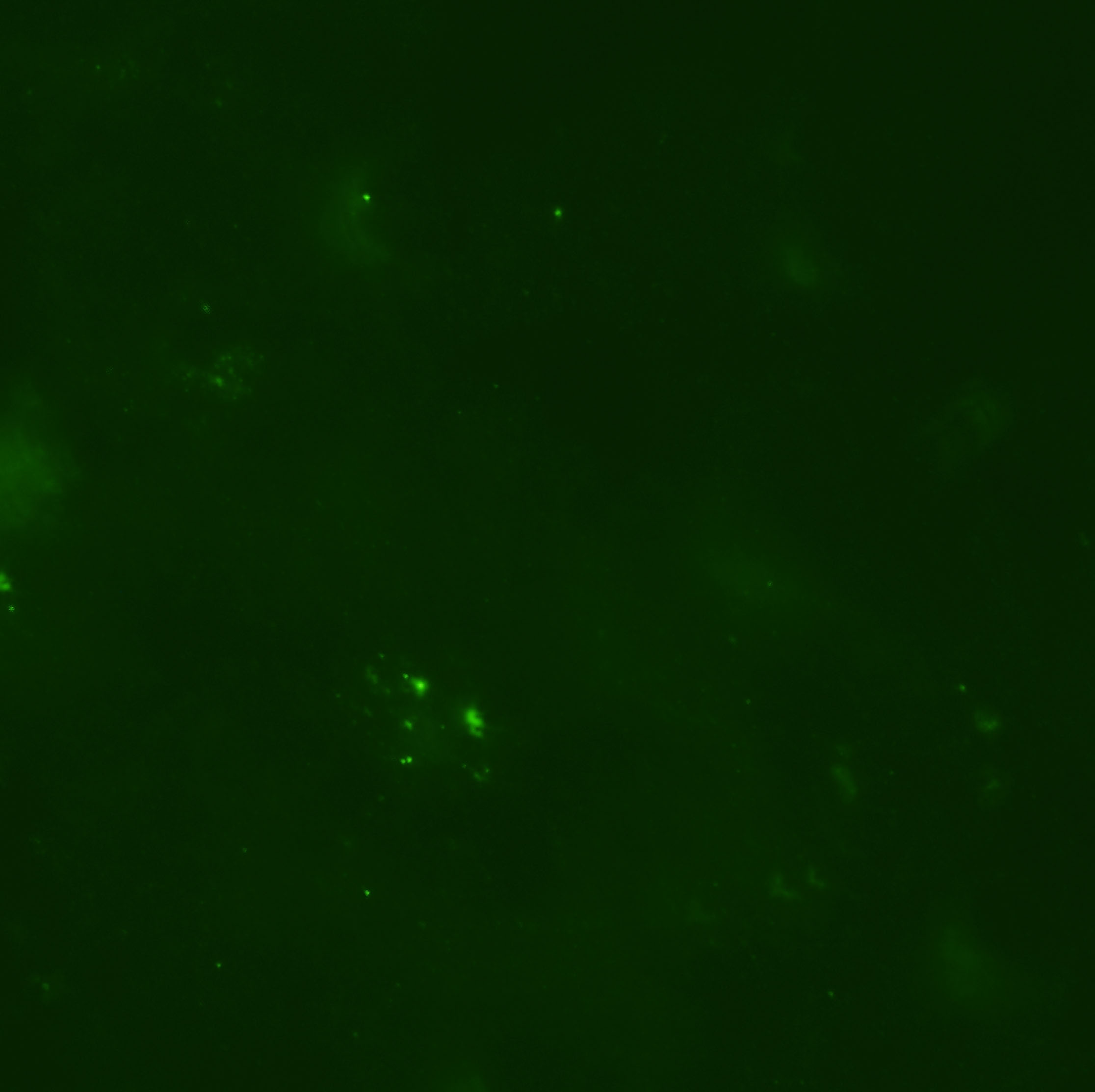

Supplement: Supplementary file 17 — Image files for Extended Data Fig. 5a–h. [file 41590_2024_1902_MOESM17_ESM.zip › ED Fig 5g WTTNIPrab7-rab.jpg]

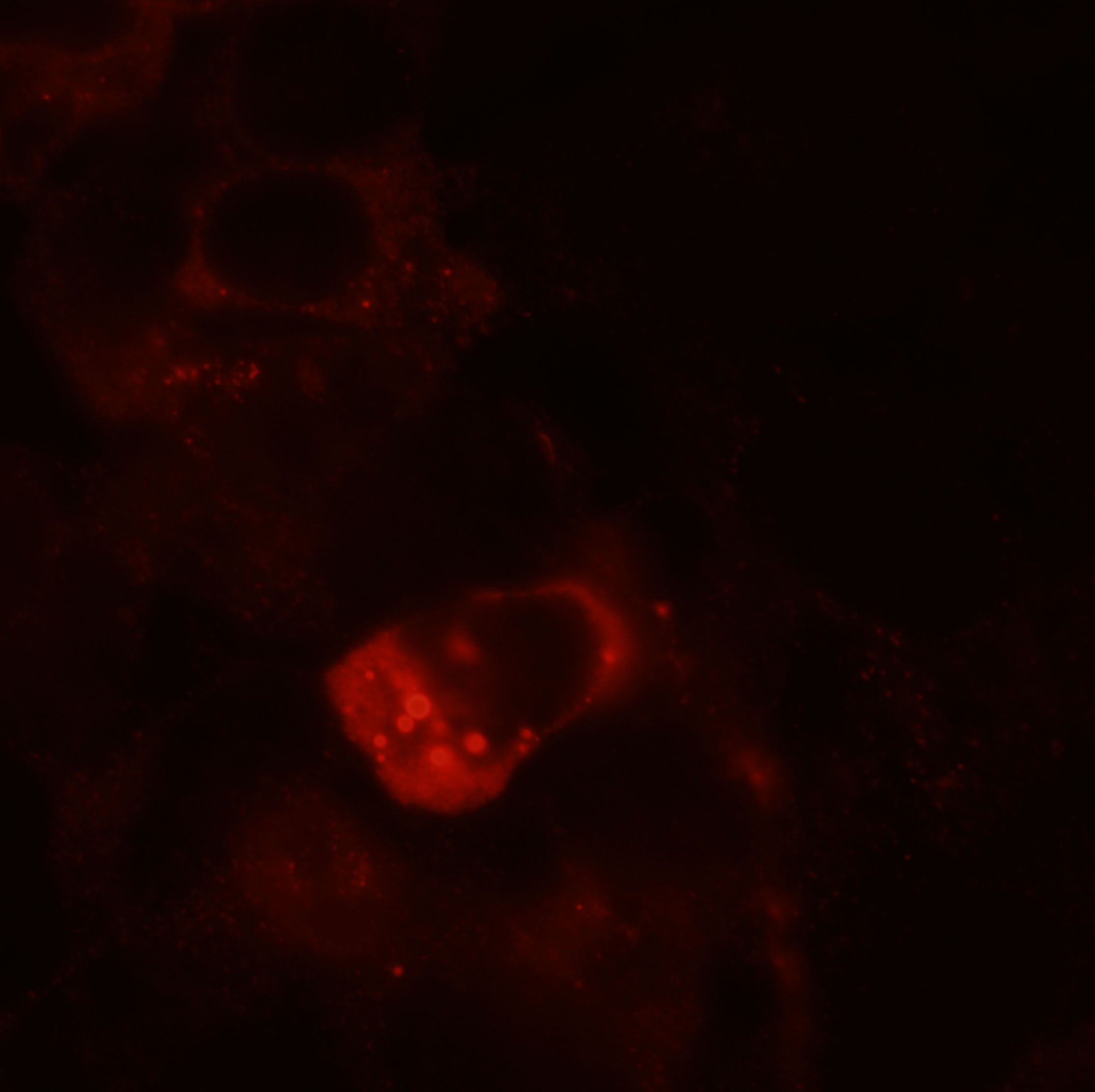

Supplement: Supplementary file 17 — Image files for Extended Data Fig. 5a–h. [file 41590_2024_1902_MOESM17_ESM.zip › ED Fig 5g WTTNIPrab7-tnip.jpg]

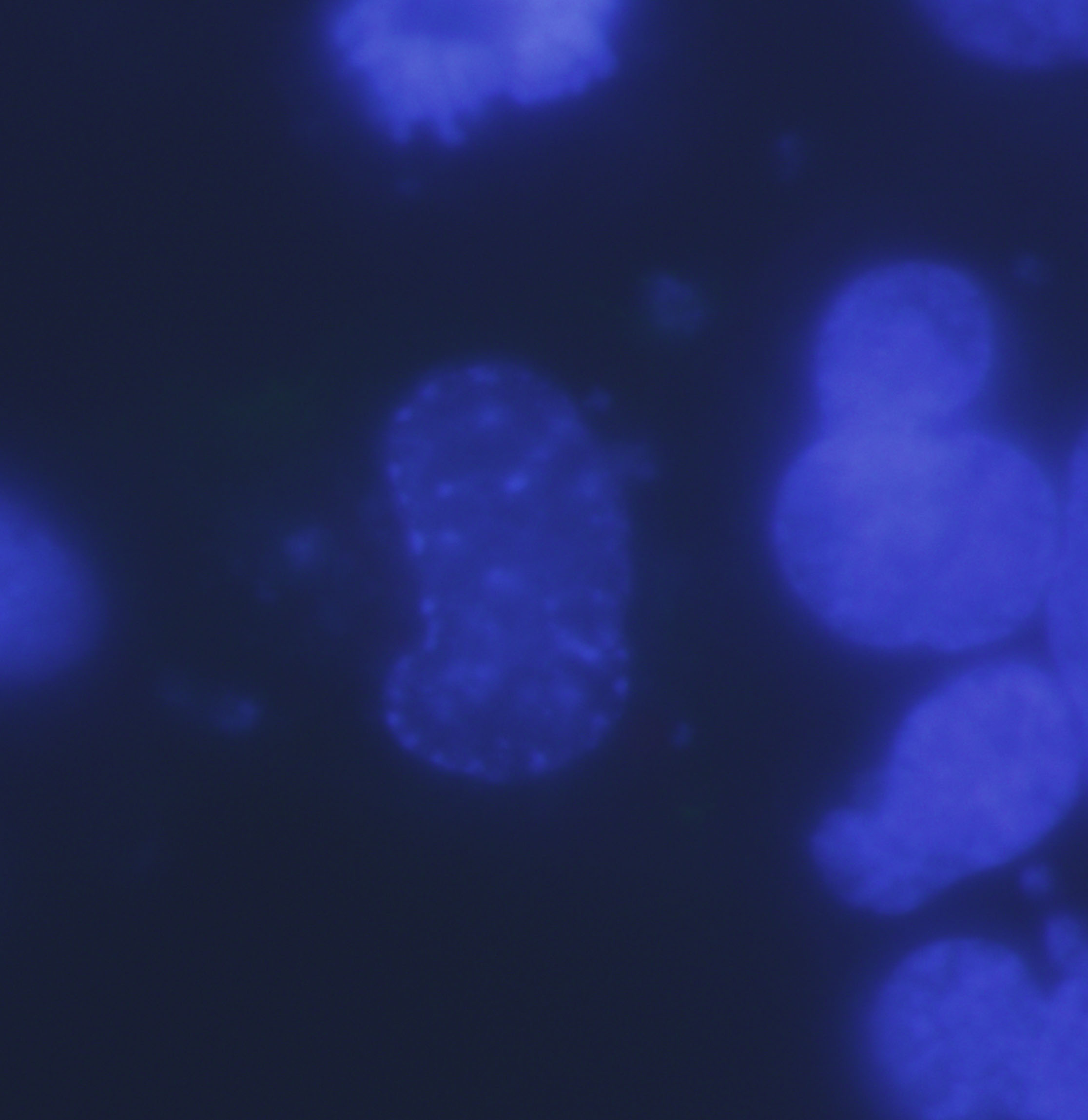

Supplement: Supplementary file 17 — Image files for Extended Data Fig. 5a–h. [file 41590_2024_1902_MOESM17_ESM.zip › ED Fig 5h q333pTNIP-LC3bFLAG-dna.jpg]

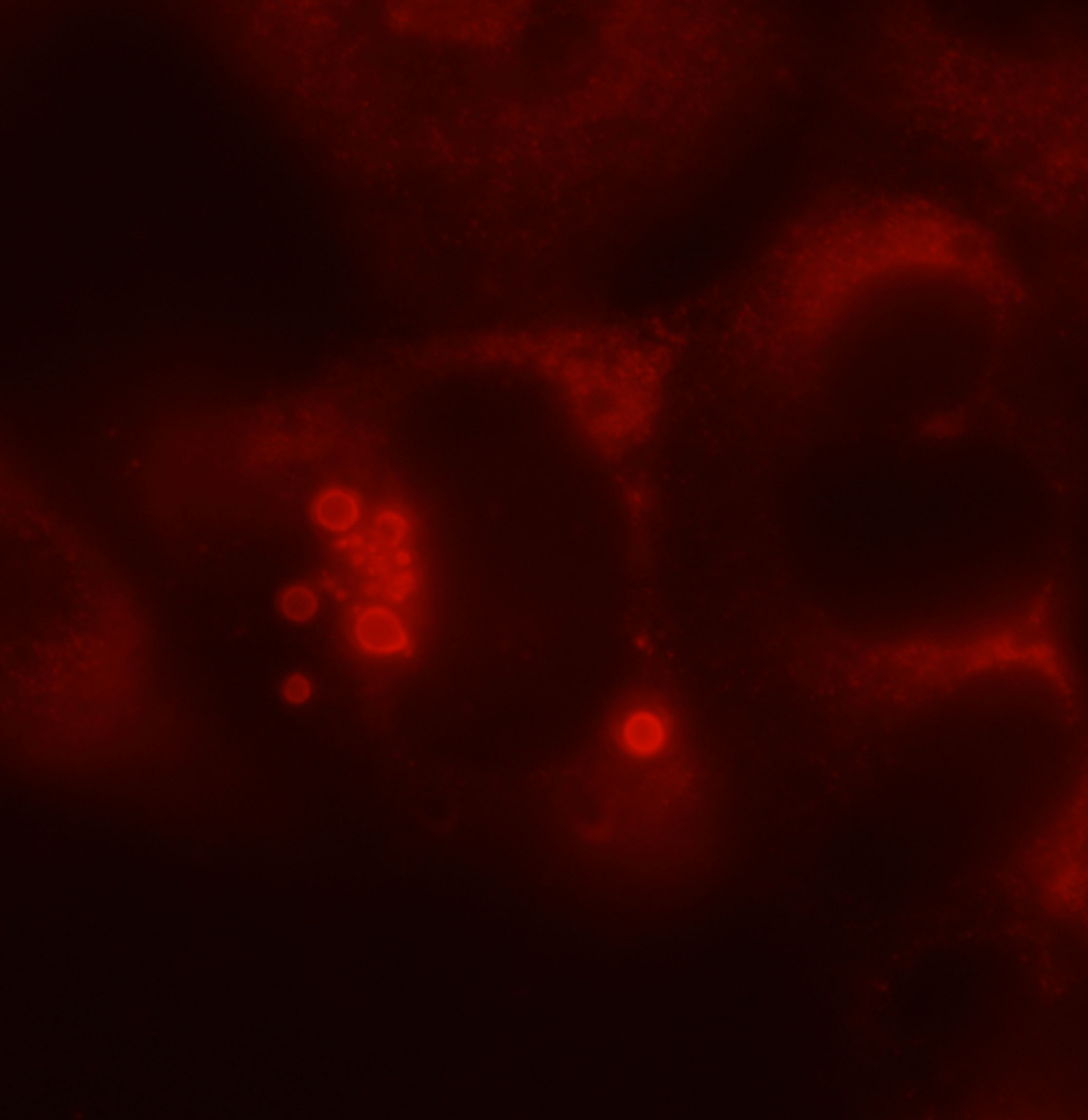

Supplement: Supplementary file 17 — Image files for Extended Data Fig. 5a–h. [file 41590_2024_1902_MOESM17_ESM.zip › ED Fig 5h q333pTNIP-LC3bFLAG-lc3.jpg]

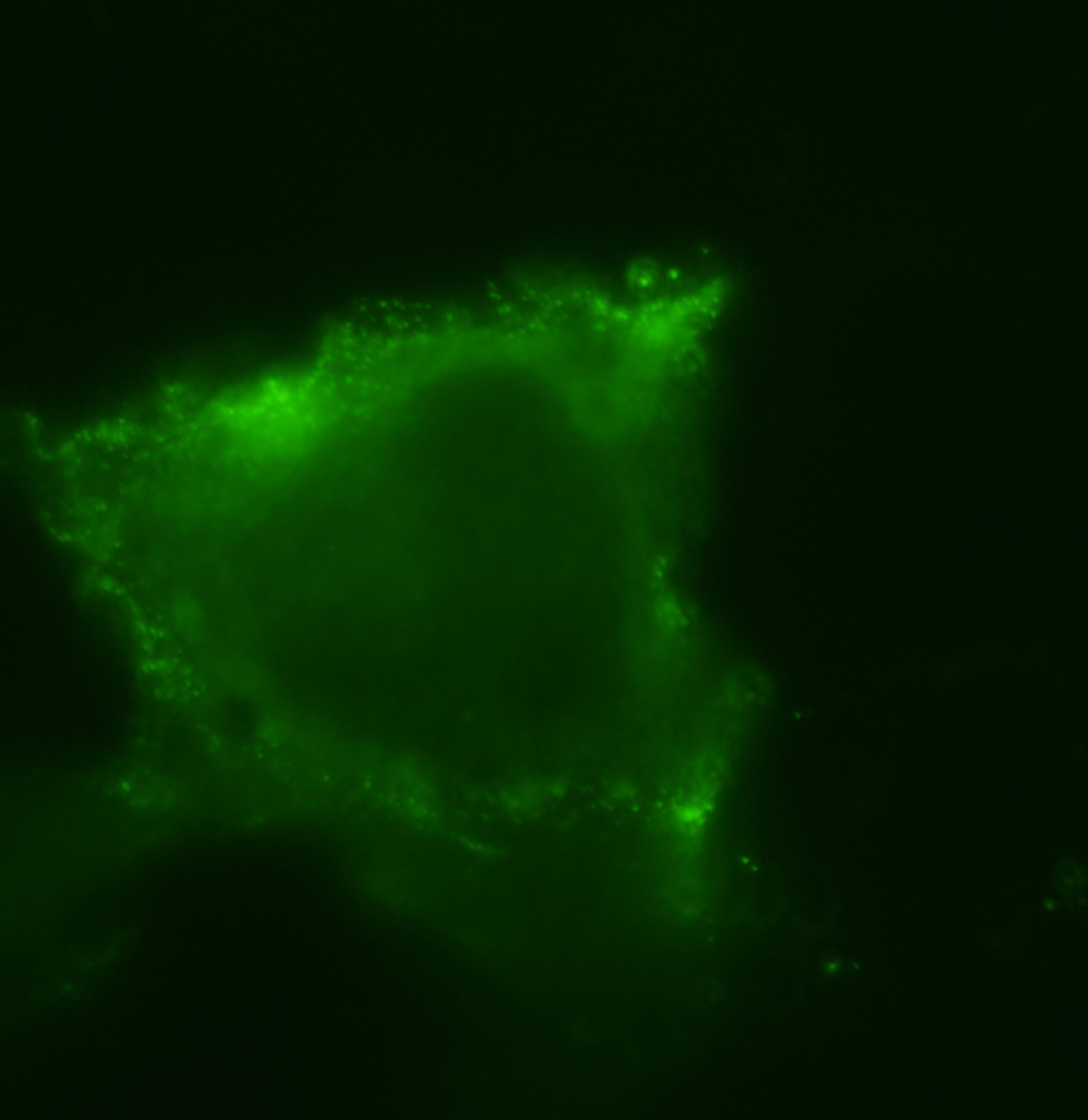

Supplement: Supplementary file 17 — Image files for Extended Data Fig. 5a–h. [file 41590_2024_1902_MOESM17_ESM.zip › ED Fig 5h q333pTNIP-LC3bFLAG-tnip.jpg]

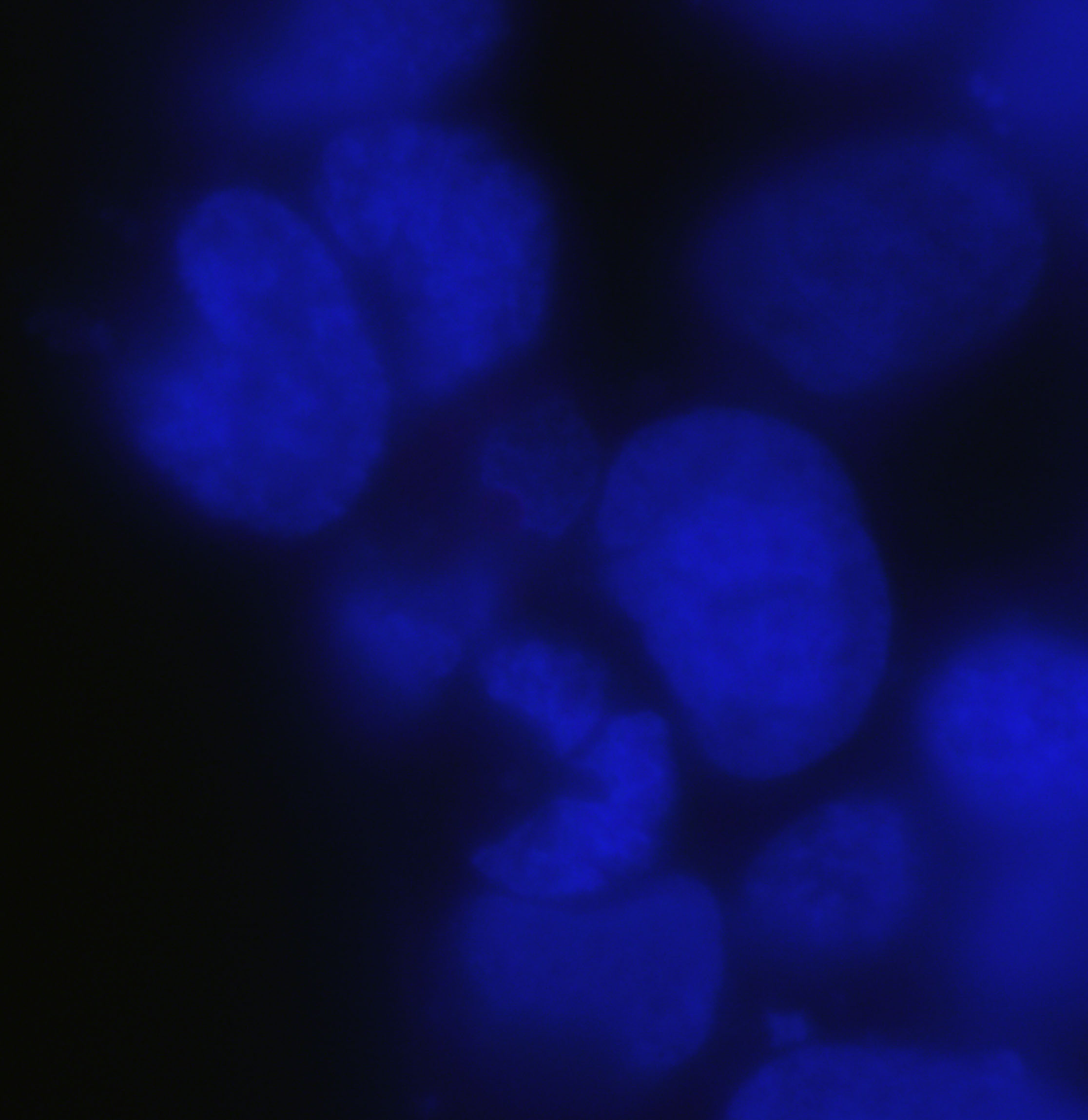

Supplement: Supplementary file 17 — Image files for Extended Data Fig. 5a–h. [file 41590_2024_1902_MOESM17_ESM.zip › ED Fig 5h WTTNIP-LC3bFLAG-dna.jpg]

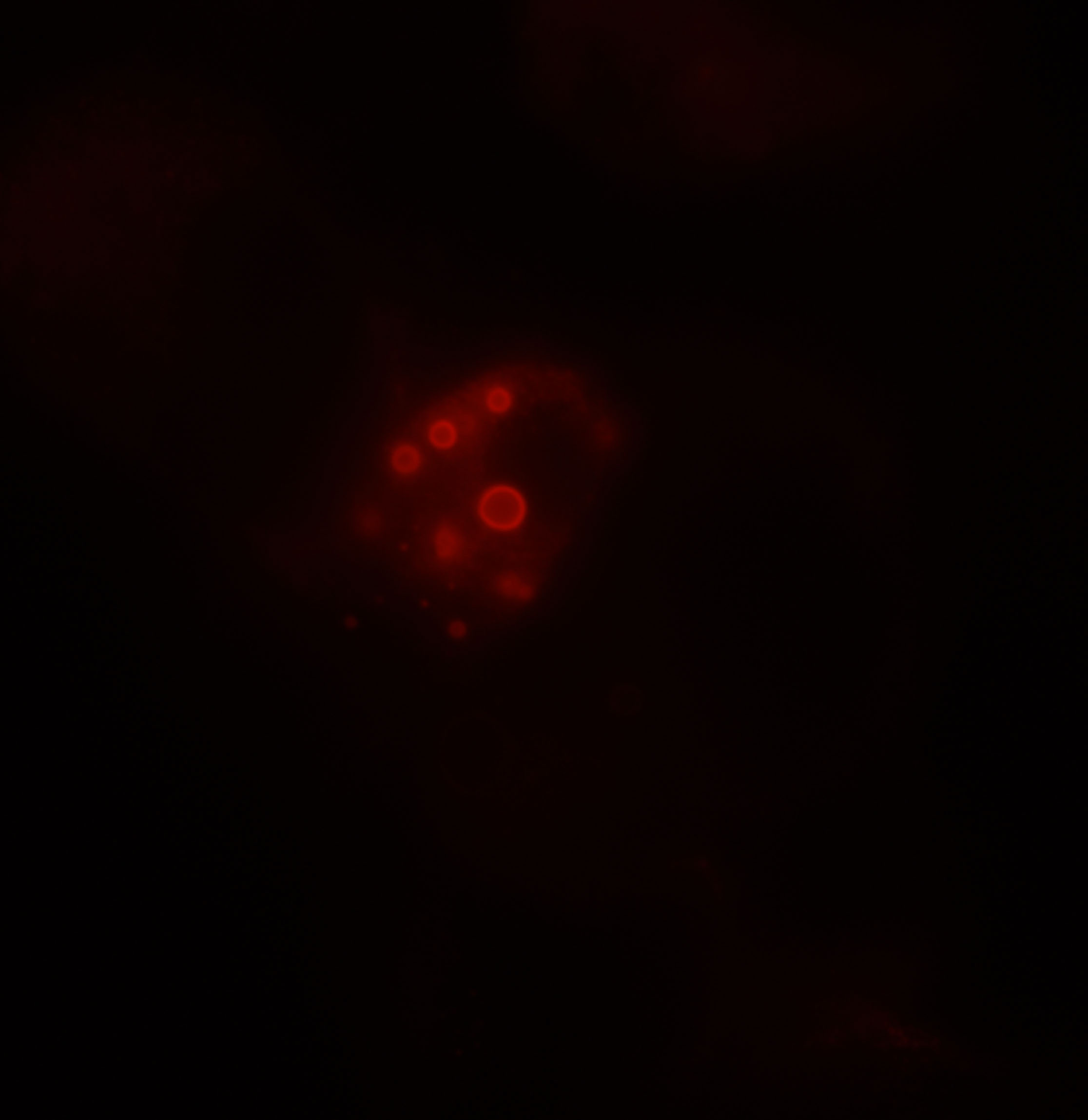

Supplement: Supplementary file 17 — Image files for Extended Data Fig. 5a–h. [file 41590_2024_1902_MOESM17_ESM.zip › ED Fig 5h WTTNIP-LC3bFLAG-lc3.jpg]

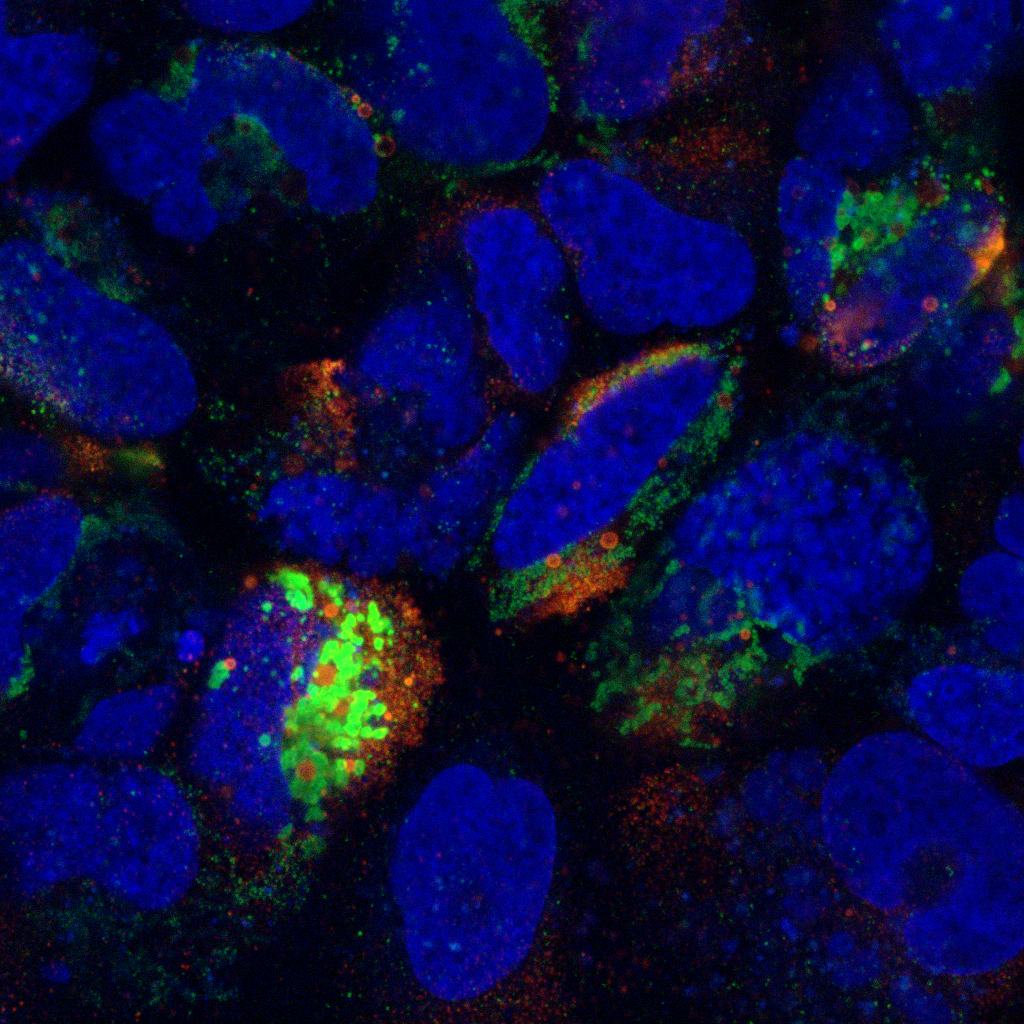

Supplement: Supplementary file 18 — Image files Extended Data Fig. 6a,b. [file 41590_2024_1902_MOESM18_ESM.zip › ED Fig 6a oligoq333p TNIP1T6BPTOM20.tif]

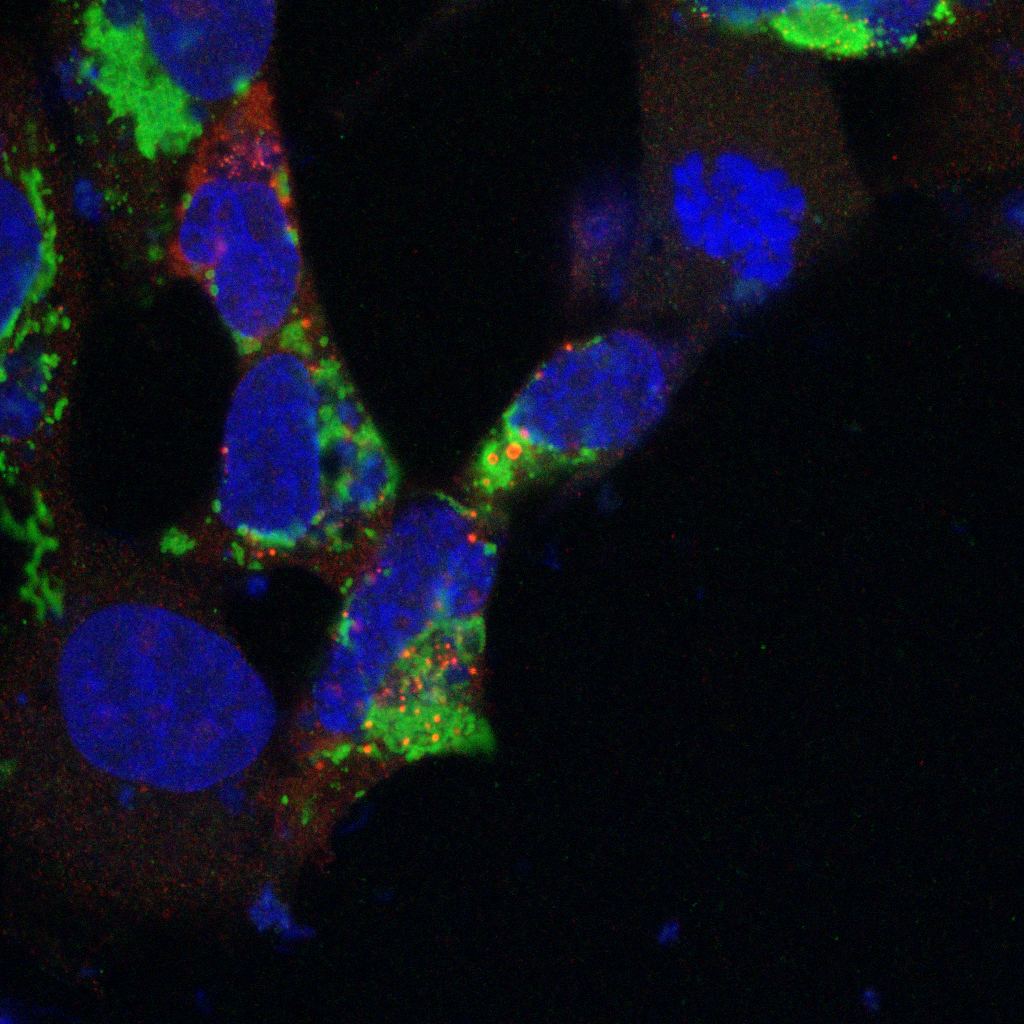

Supplement: Supplementary file 18 — Image files Extended Data Fig. 6a,b. [file 41590_2024_1902_MOESM18_ESM.zip › ED Fig 6a q333p TNIP1T6BPTOM20.tif]

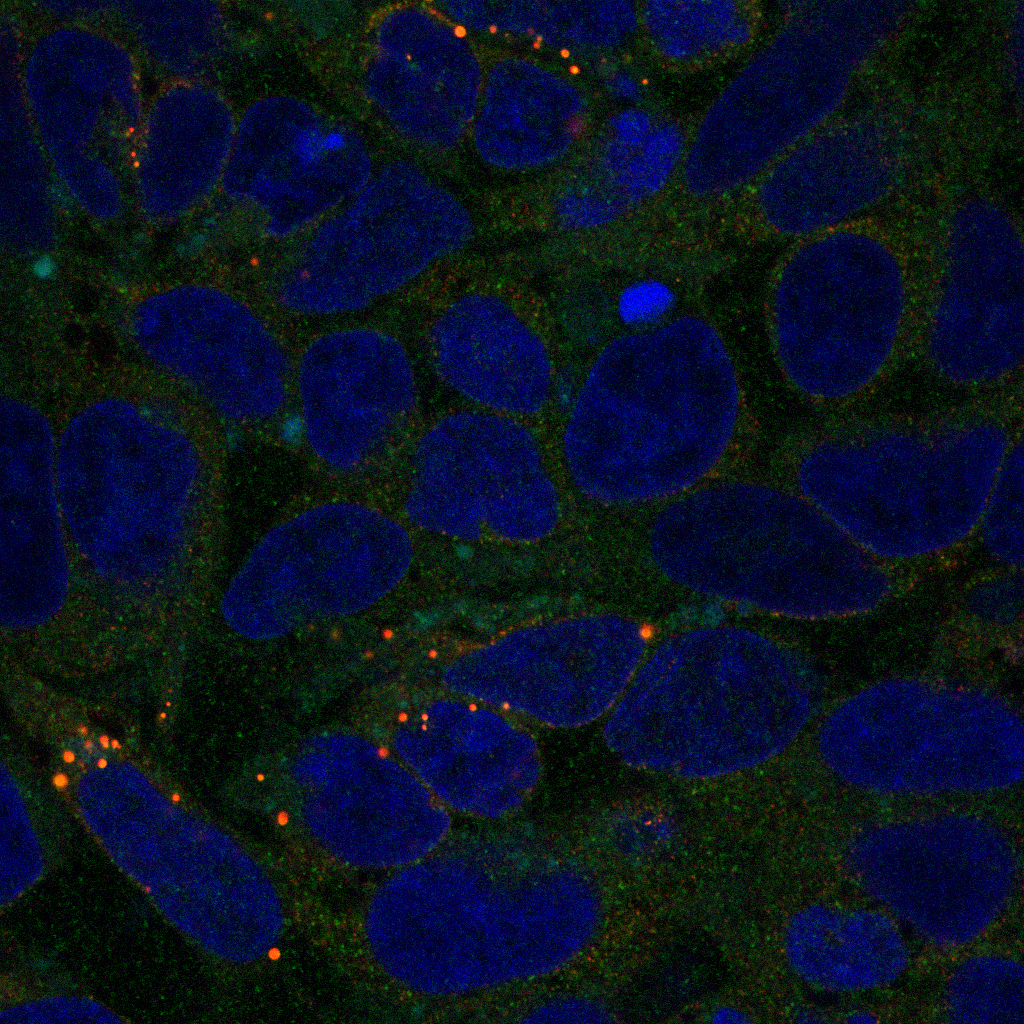

Supplement: Supplementary file 18 — Image files Extended Data Fig. 6a,b. [file 41590_2024_1902_MOESM18_ESM.zip › ED Fig 6b q333p TNIP1 IRGM.tif]

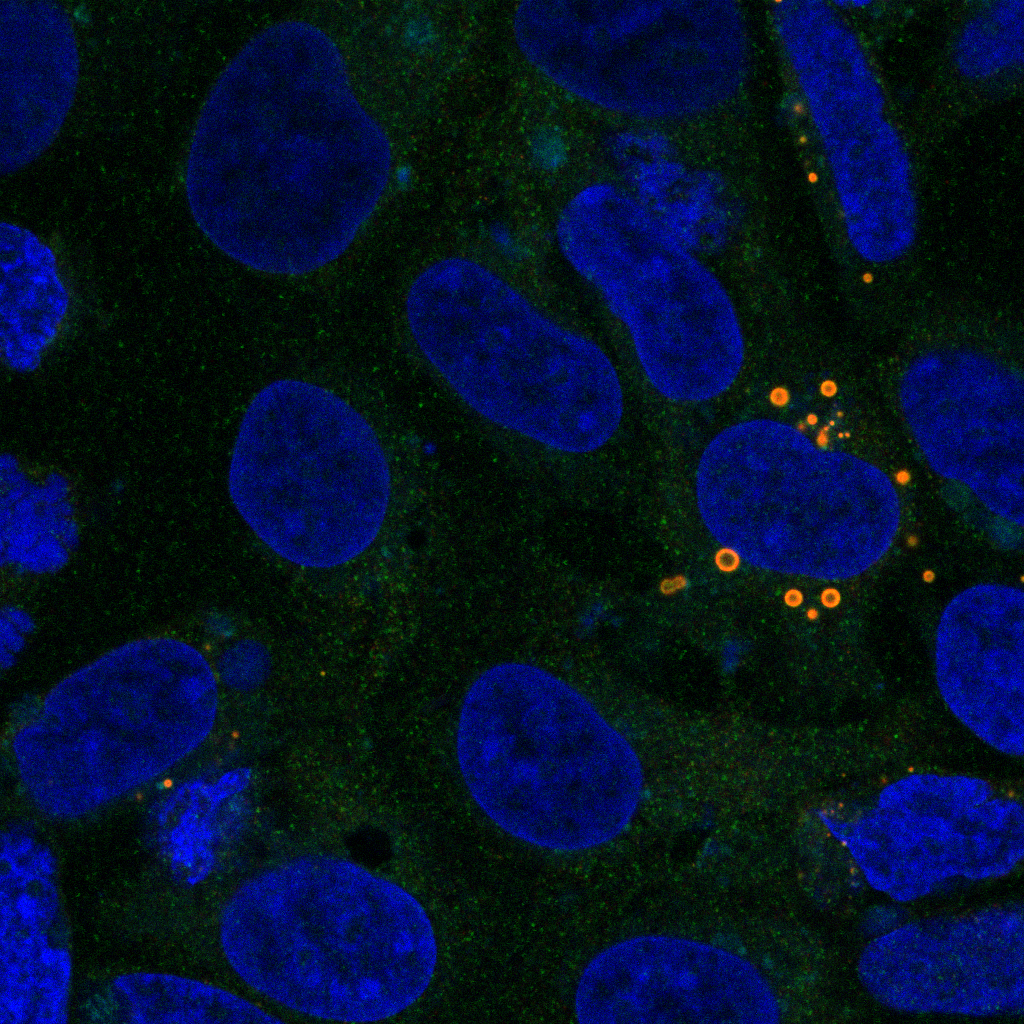

Supplement: Supplementary file 18 — Image files Extended Data Fig. 6a,b. [file 41590_2024_1902_MOESM18_ESM.zip › ED Fig 6b wtTNIP1 IRGM.tif]

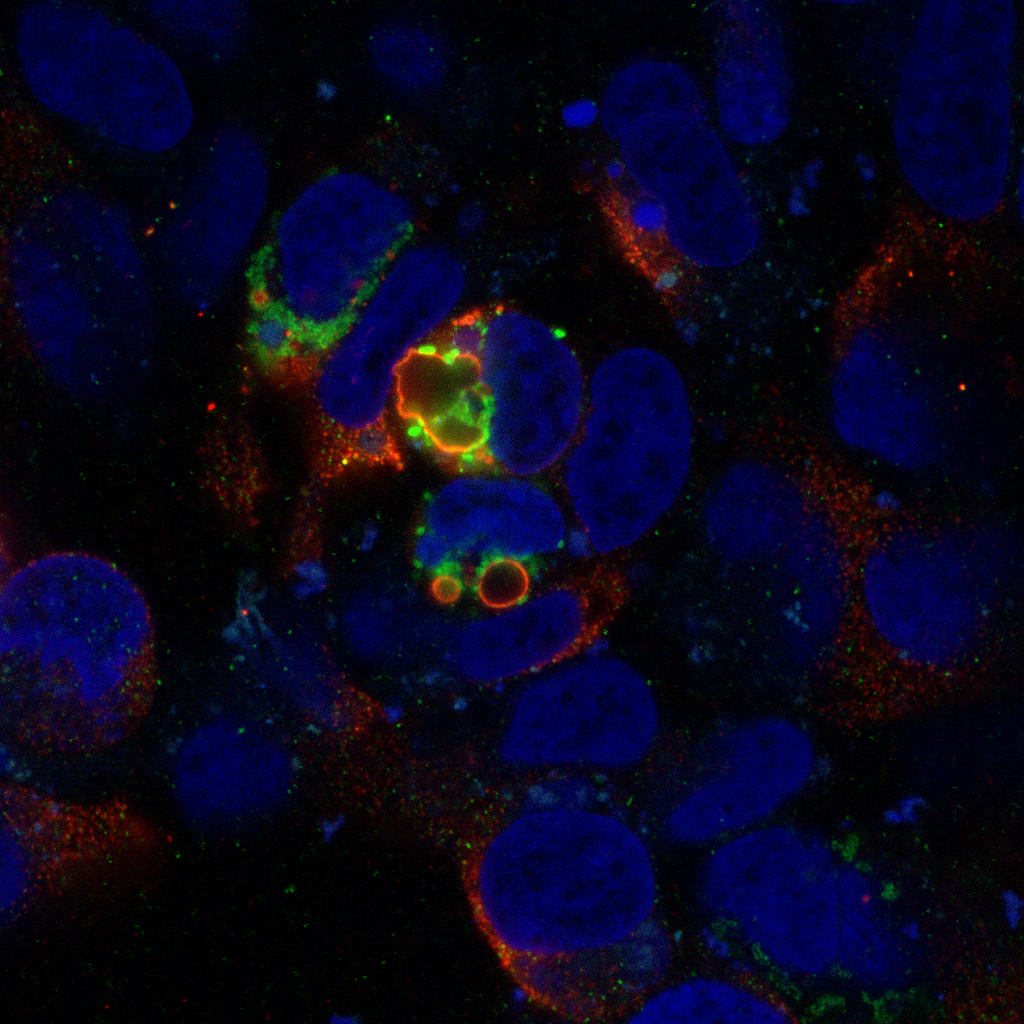

Supplement: Supplementary file 18 — Image files Extended Data Fig. 6a,b. [file 41590_2024_1902_MOESM18_ESM.zip › ED Fig 6a wt TNIP1T6BPTOM20.tif]

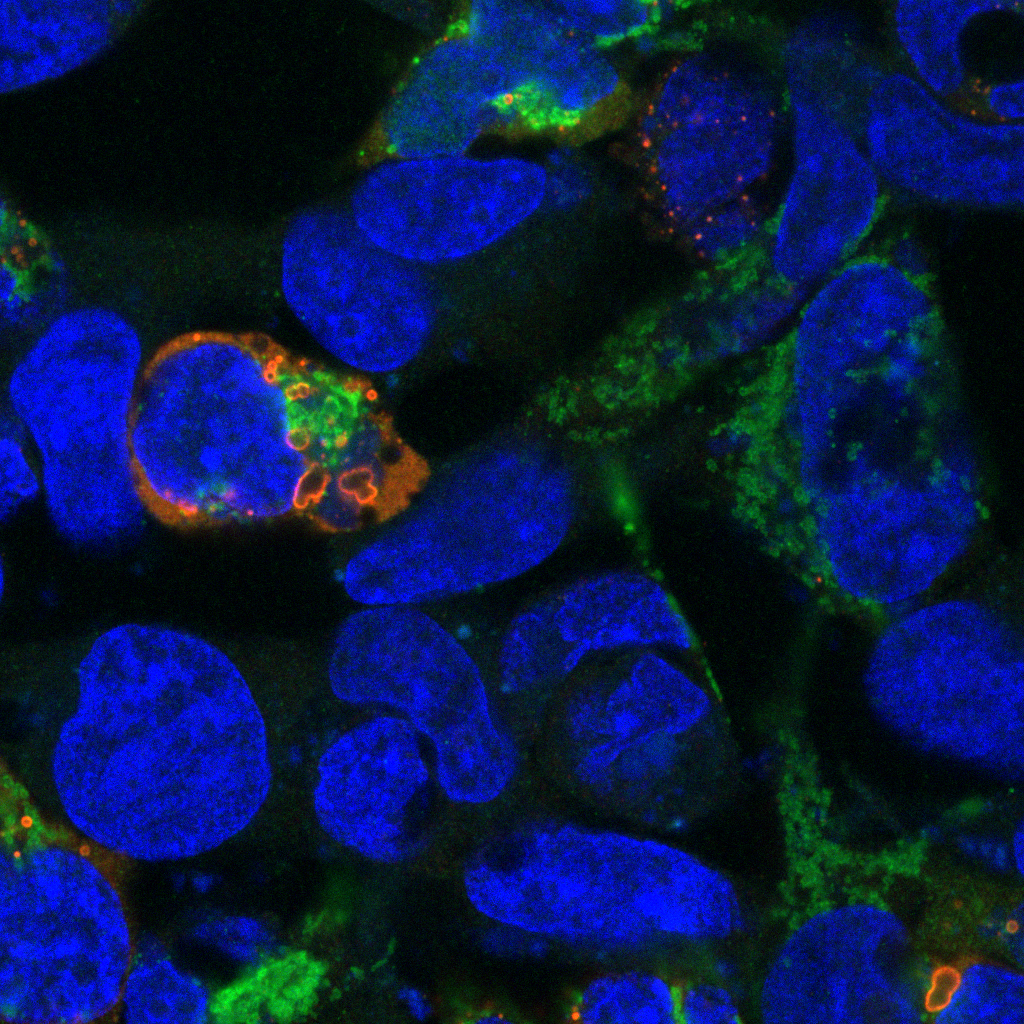

Supplement: Supplementary file 18 — Image files Extended Data Fig. 6a,b. [file 41590_2024_1902_MOESM18_ESM.zip › ED Fig 6a oligowt TNIP1T6BPTOM20.tif]

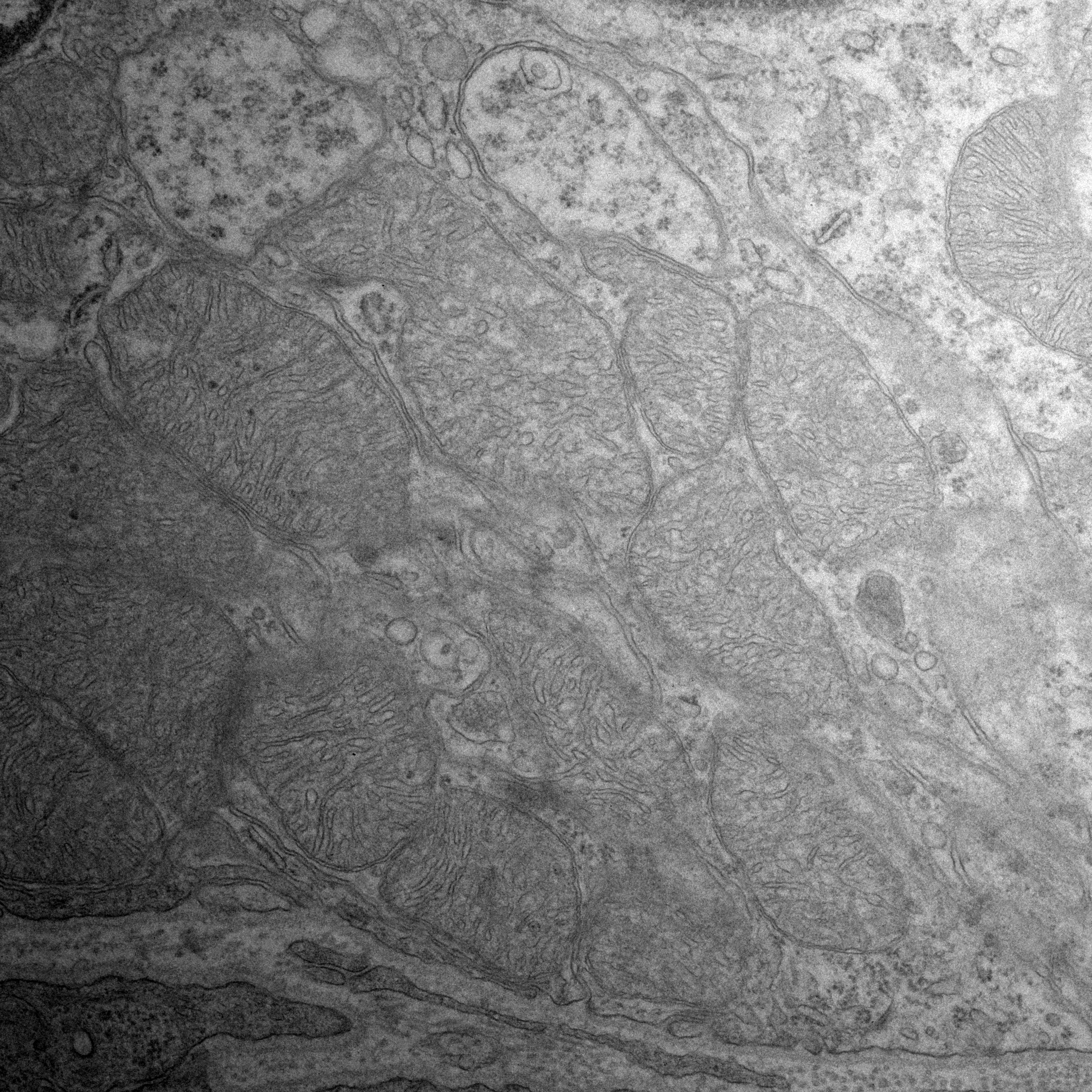

Supplement: Supplementary file 19 — Image files Extended Data Fig. 7a. [file 41590_2024_1902_MOESM19_ESM.zip › ED Fig 7a 6000X _80kV _Hom_15F.jpg]

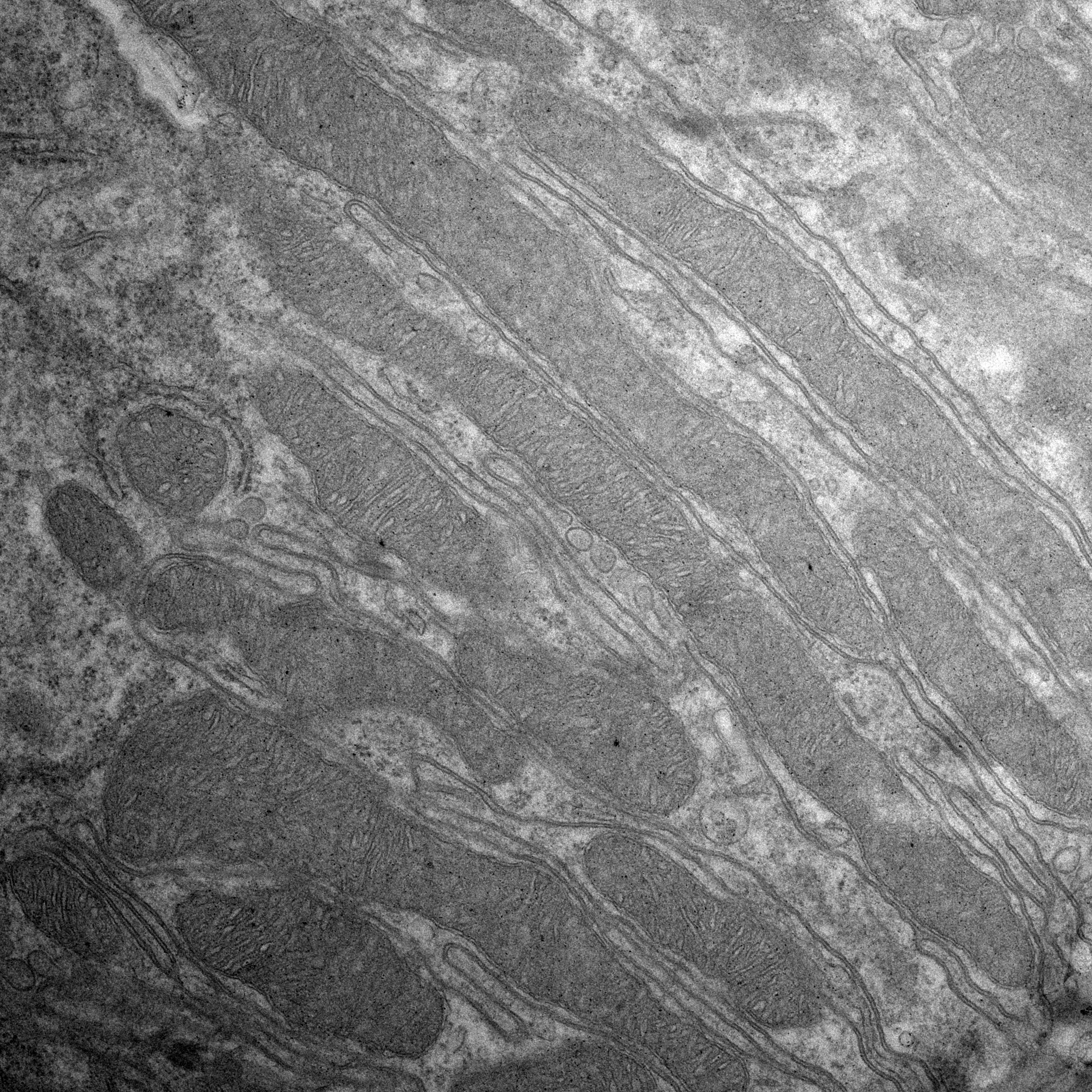

Supplement: Supplementary file 19 — Image files Extended Data Fig. 7a. [file 41590_2024_1902_MOESM19_ESM.zip › ED Fig 7a 6000X _80kV _WT_68F.jpg]
