# Supplementary material for: A TNIP1-driven systemic autoimmune disorder with elevated IgG4
Source: Nat Immunol. 2024 Jul 26;25(9):1678–91. doi: 10.1038/s41590-024-01902-0 (PMC11362012; doi:10.1038/s41590-024-01902-0)

Uncropped western blots for Fig. 5d

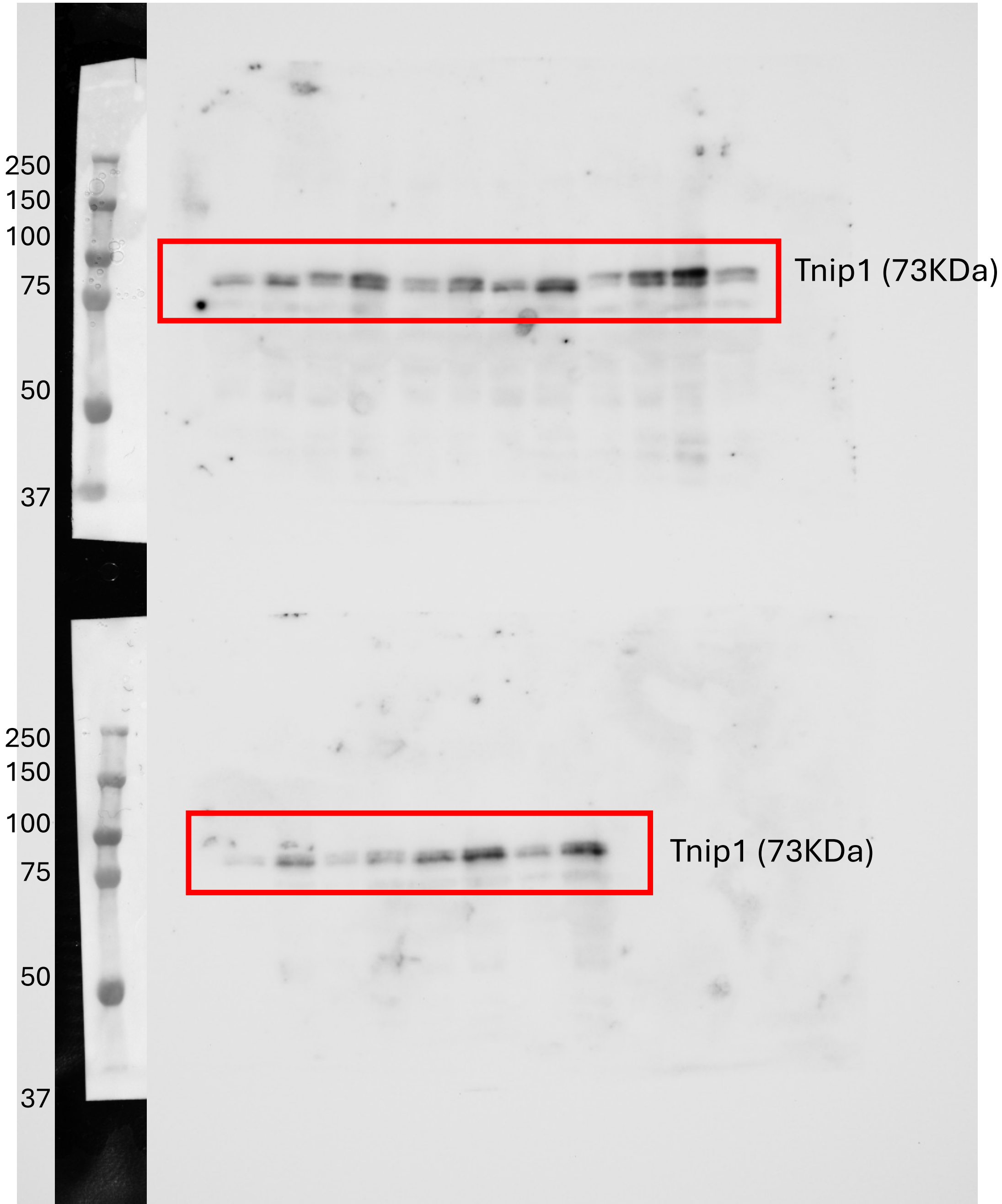

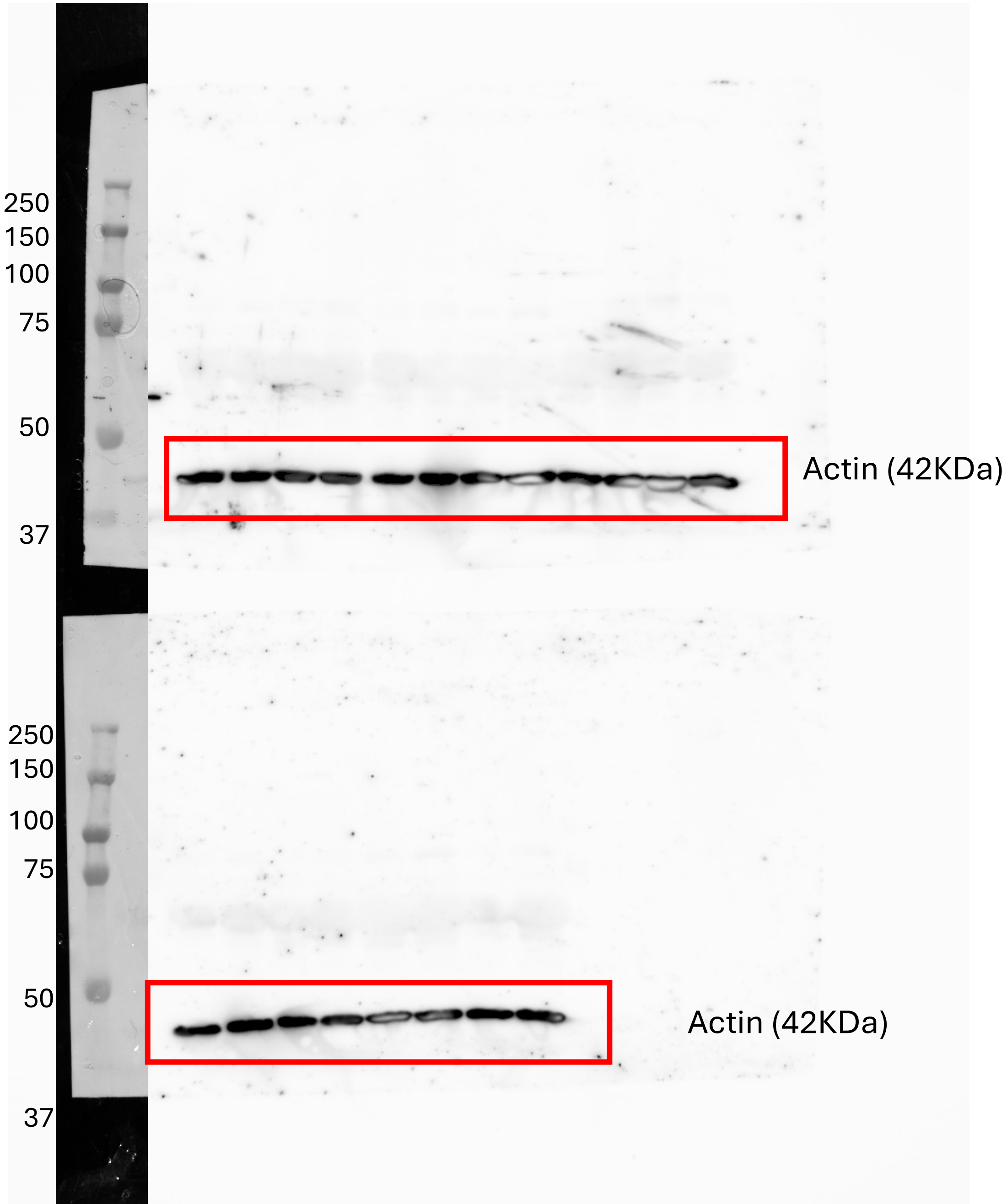

Uncropped western blots for Fig. 6e WCE

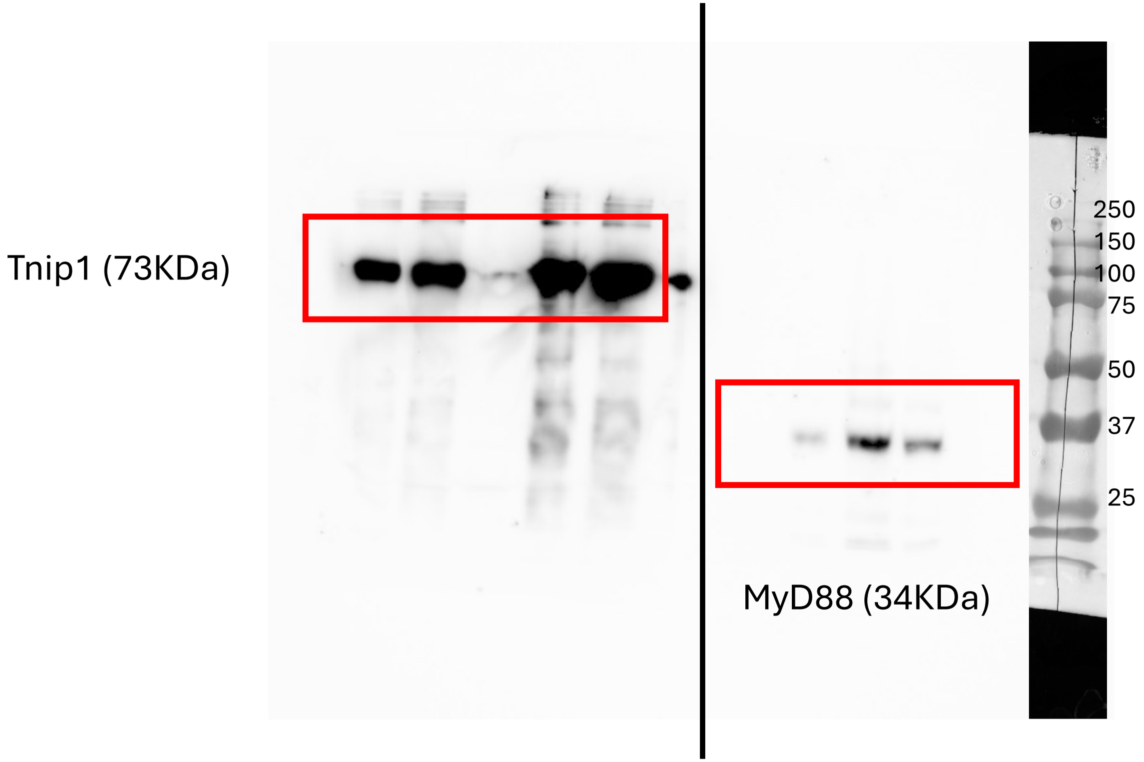

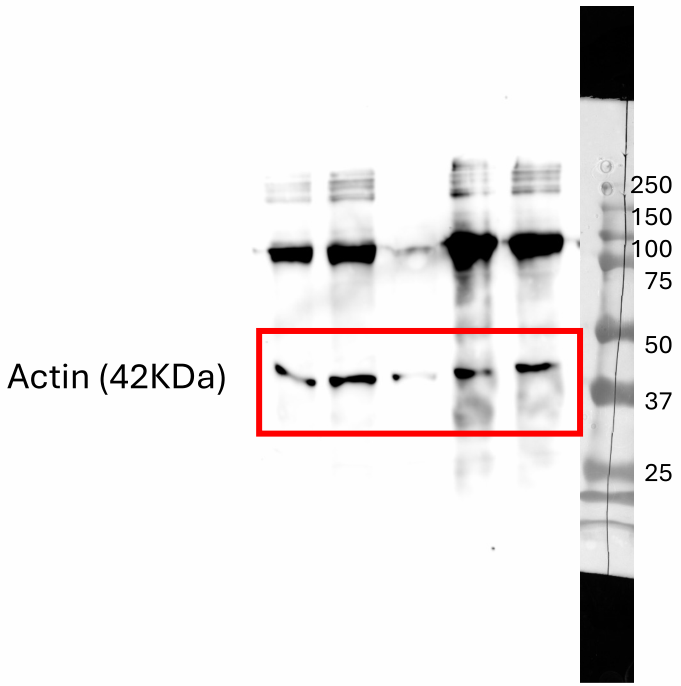

Uncropped western blots for Fig. 6e IP TNIP1/HA

Tnip1 (73KDa)

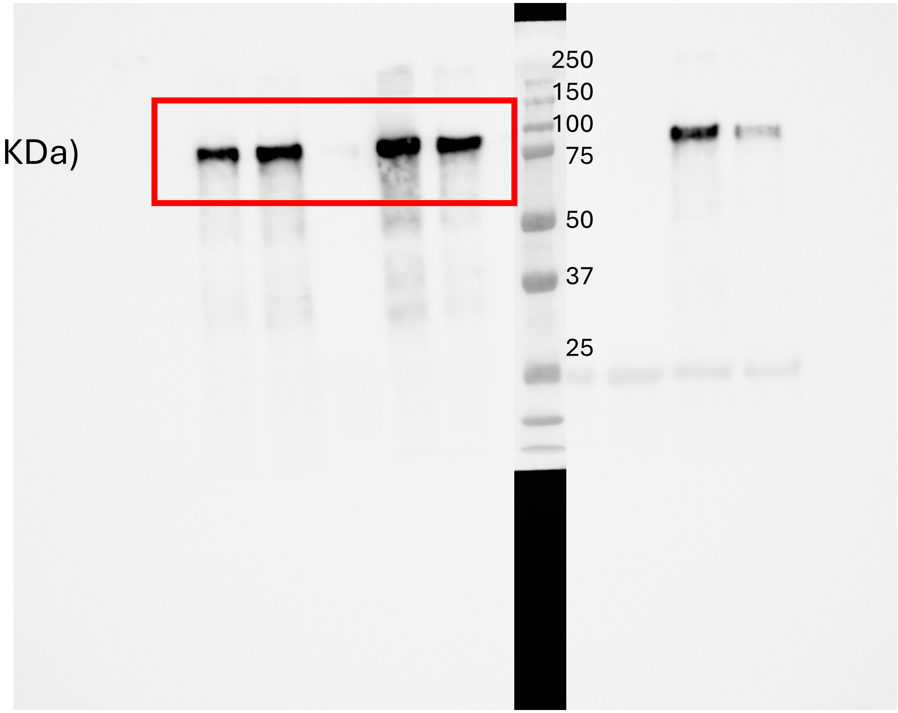

MyD88 (34KDa)

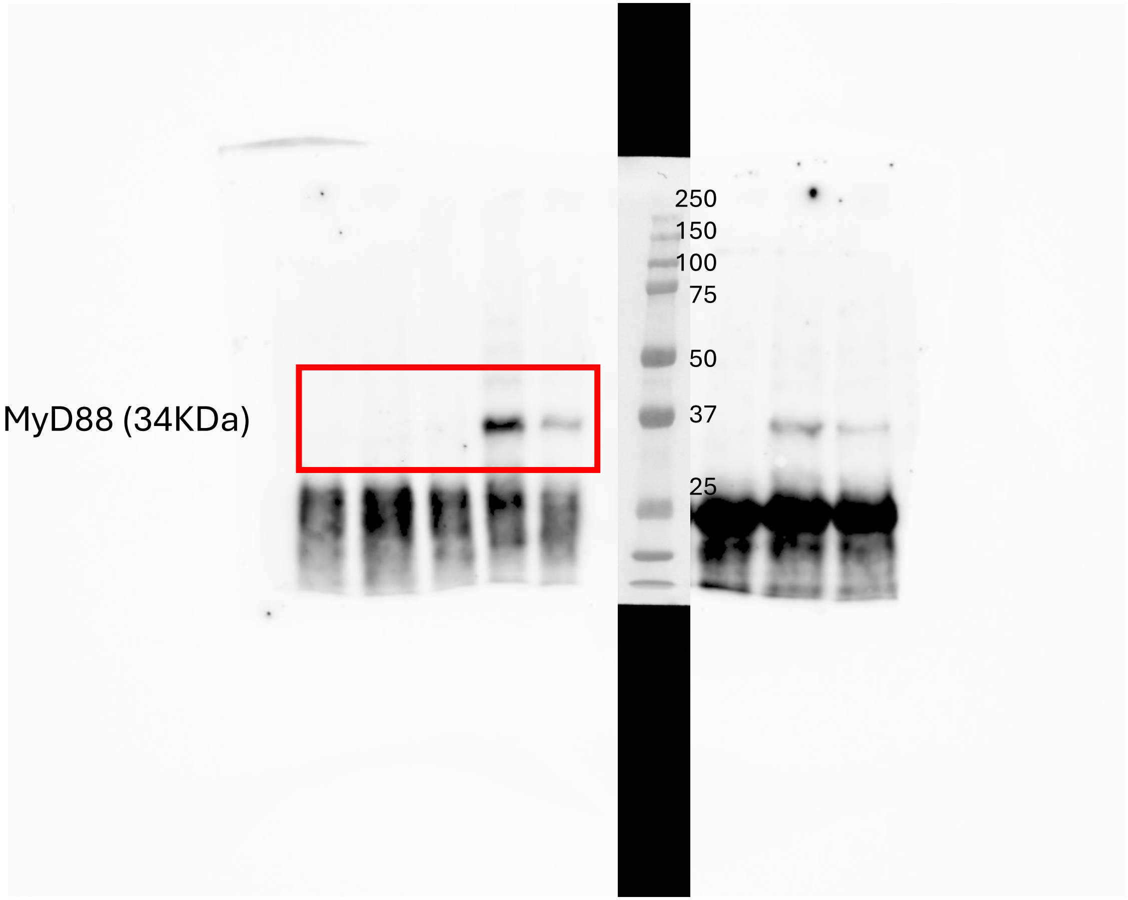

Uncropped western blots for Fig. 6e IP MyD88

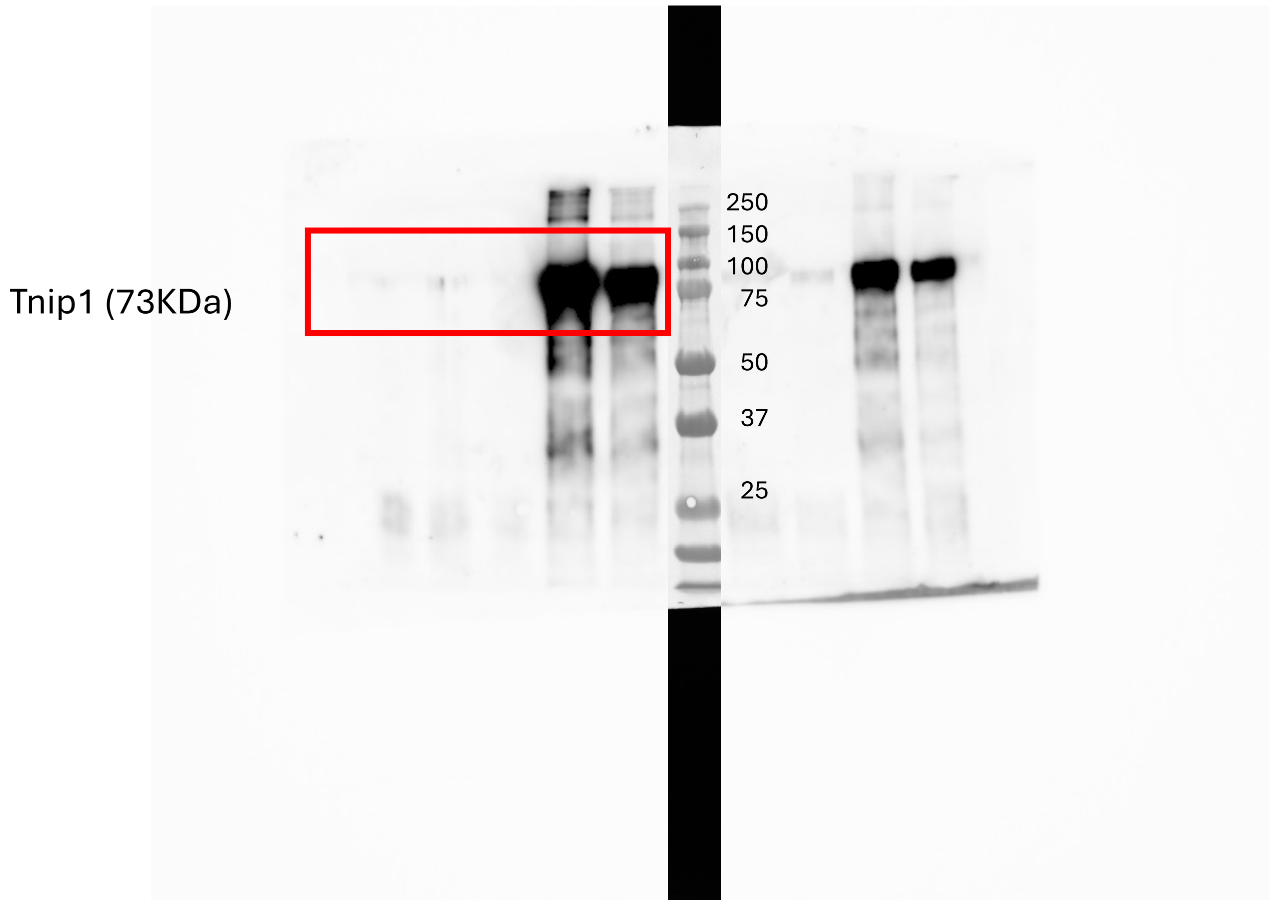

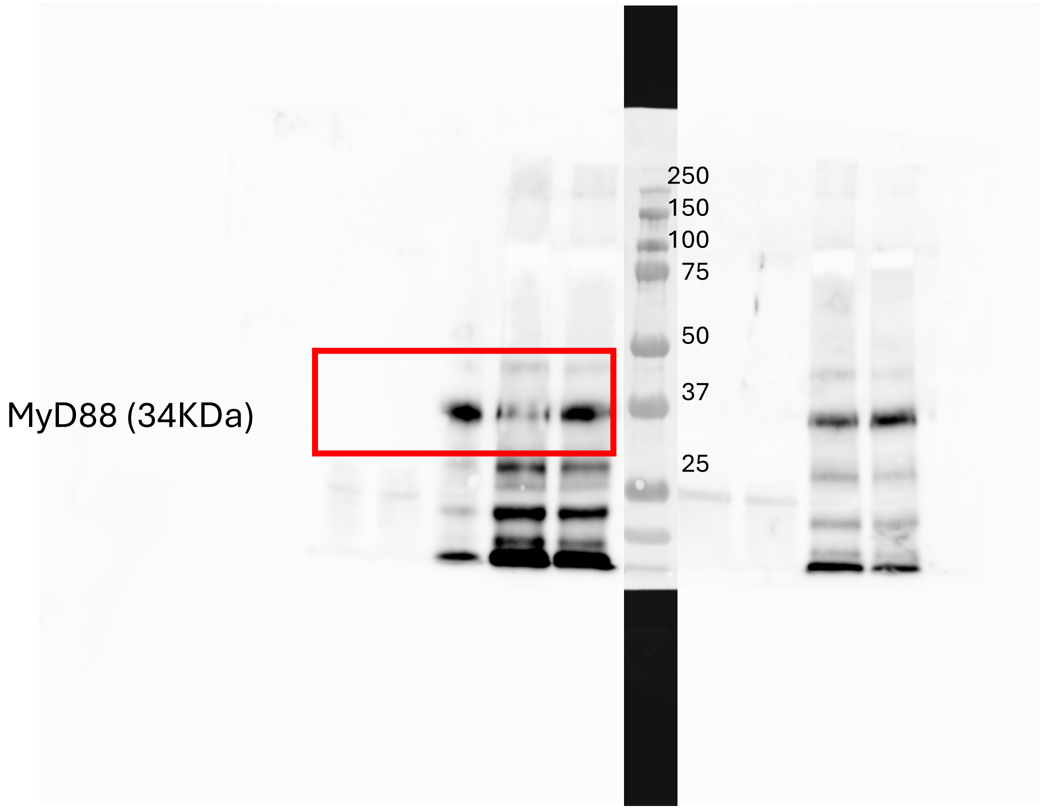

Uncropped western blots for Extended Data Fig 4a

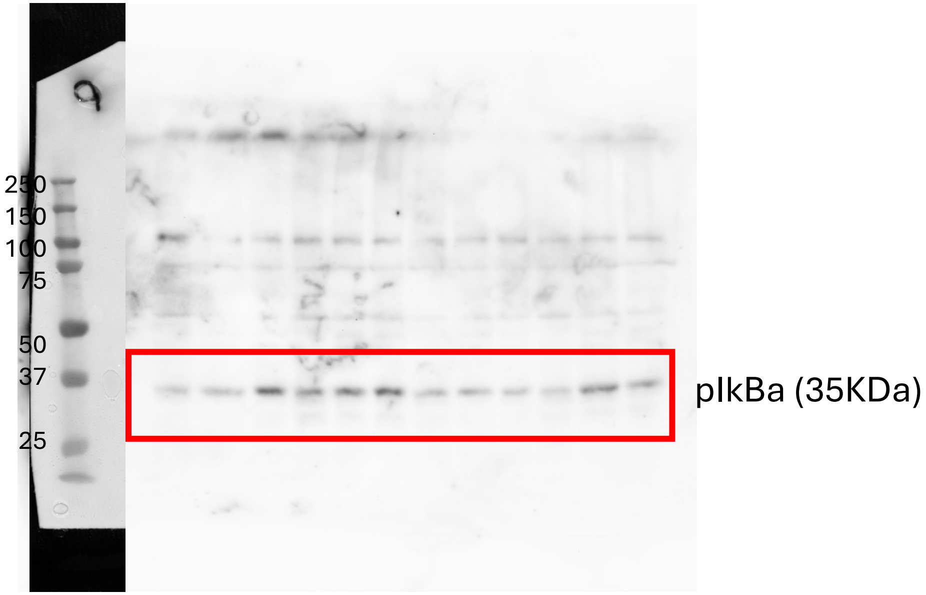

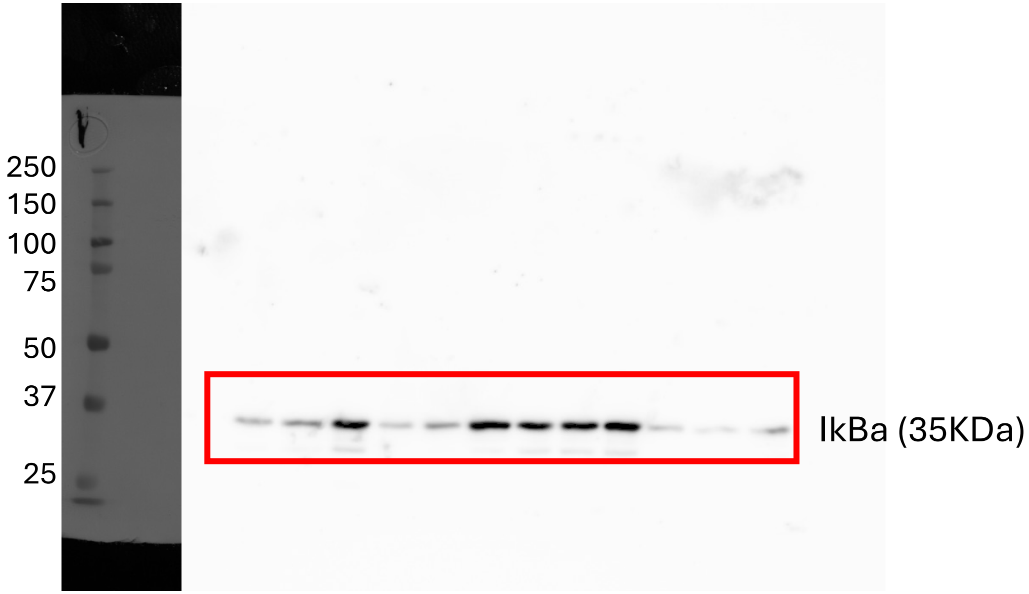

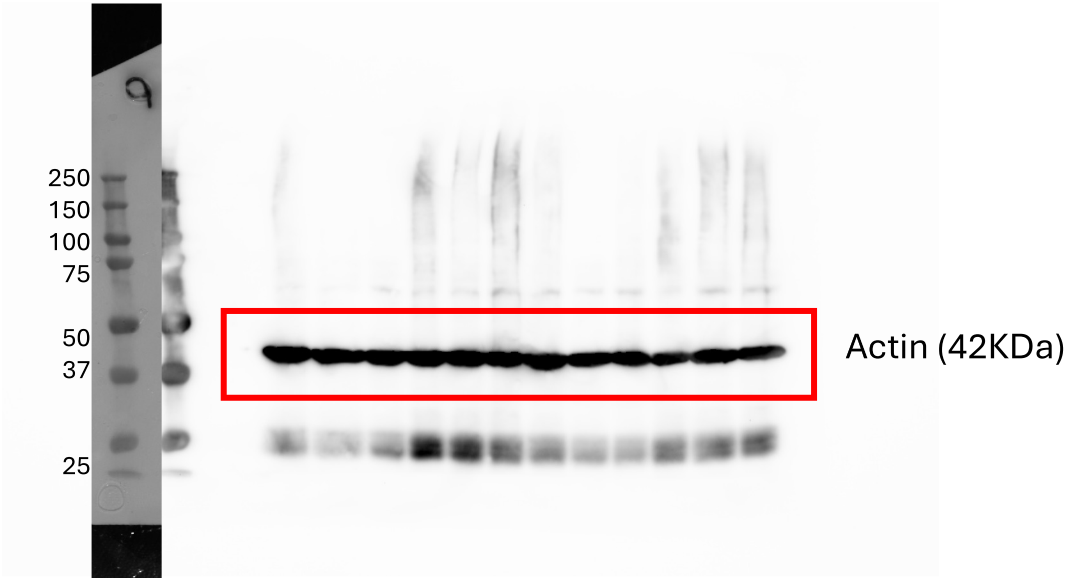

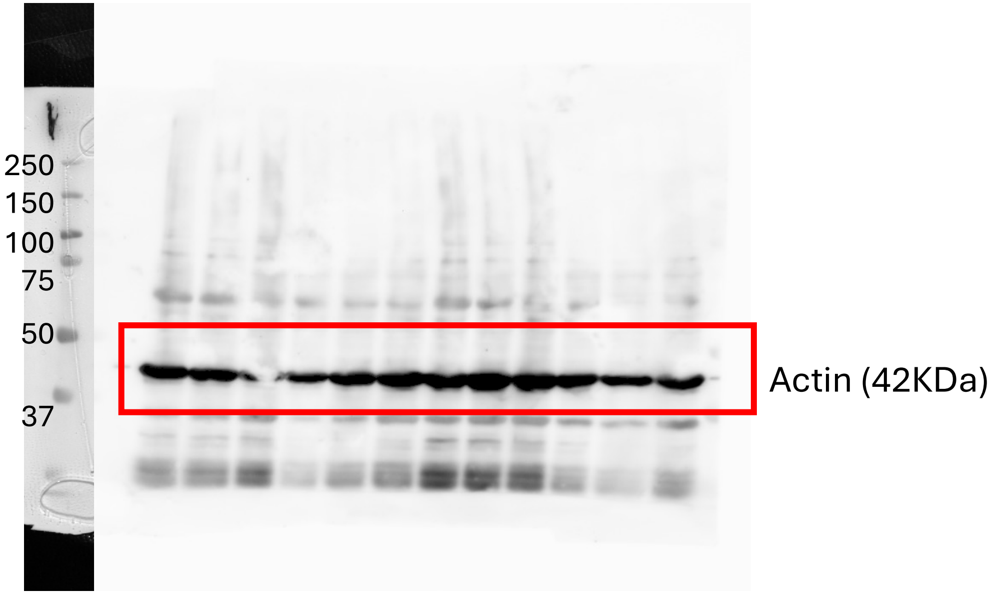

Uncropped western blots for Extended Data Fig 4b

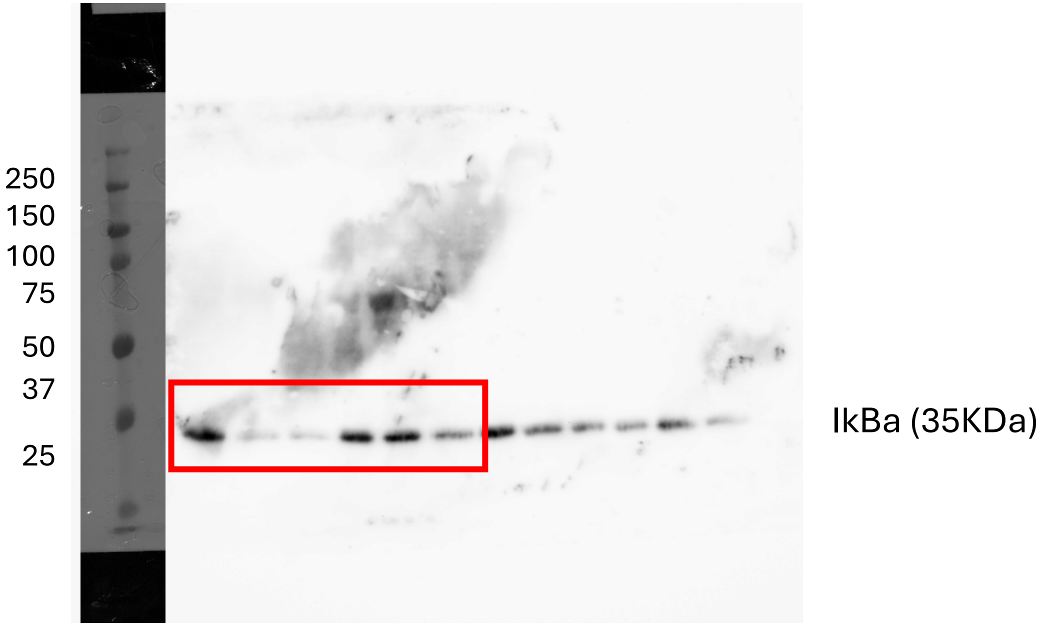

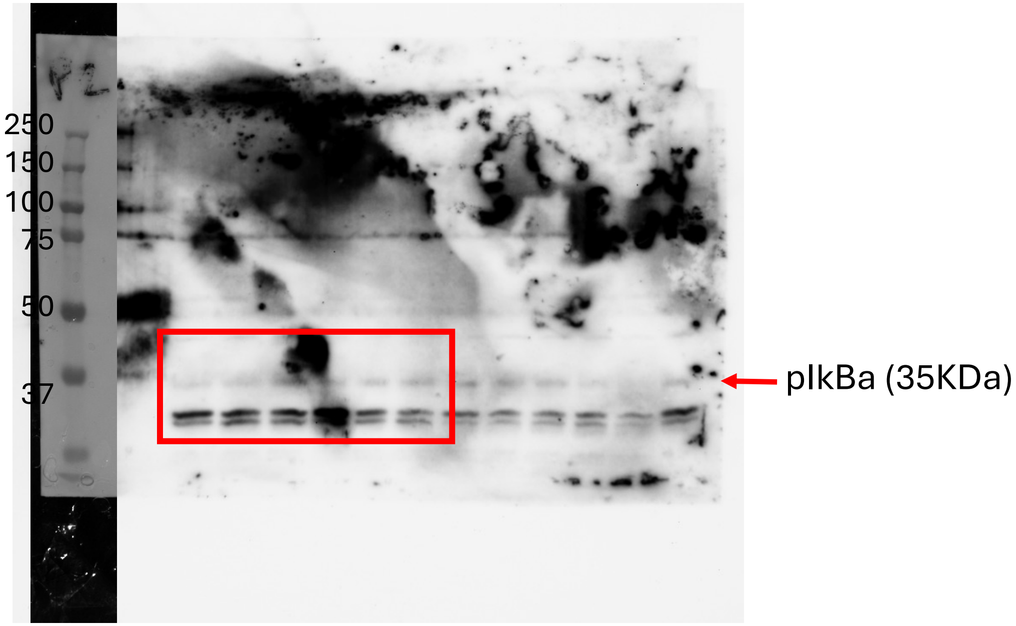

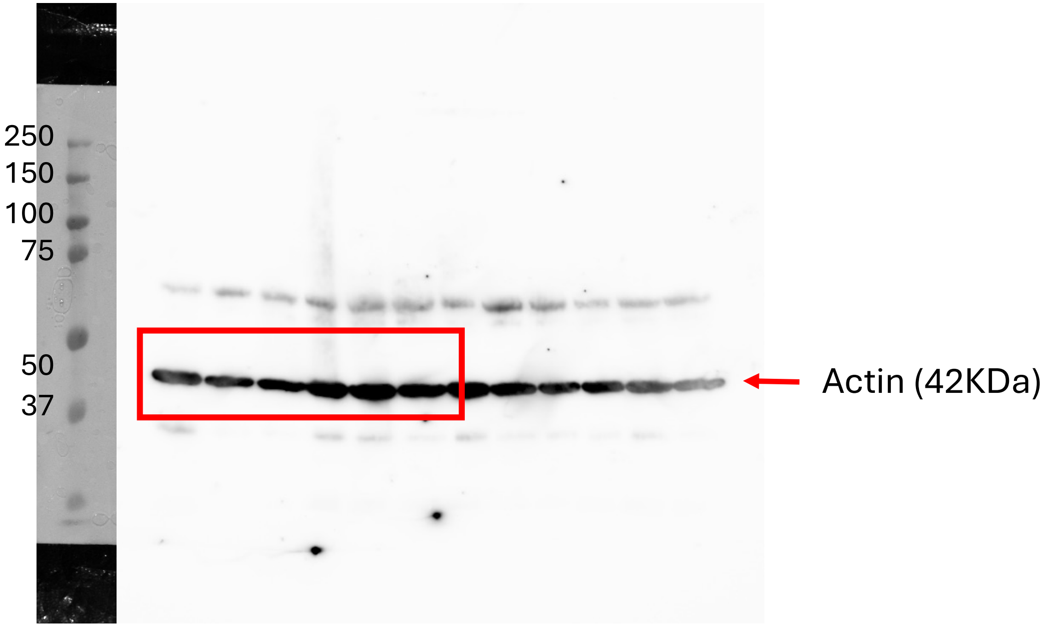

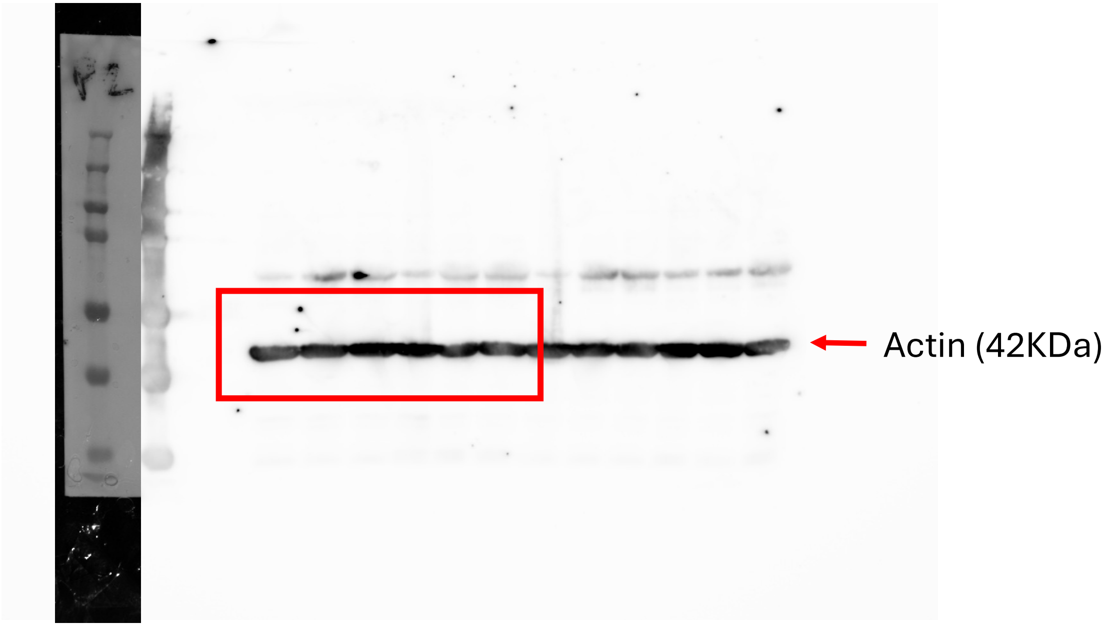

Uncropped western blots for Extended Data Fig 4c

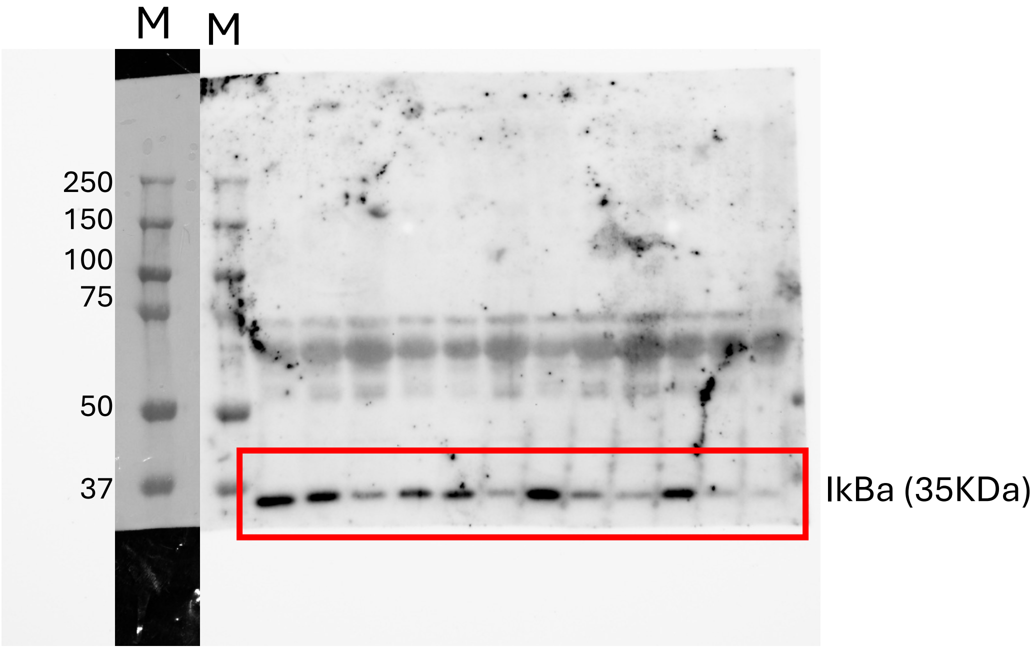

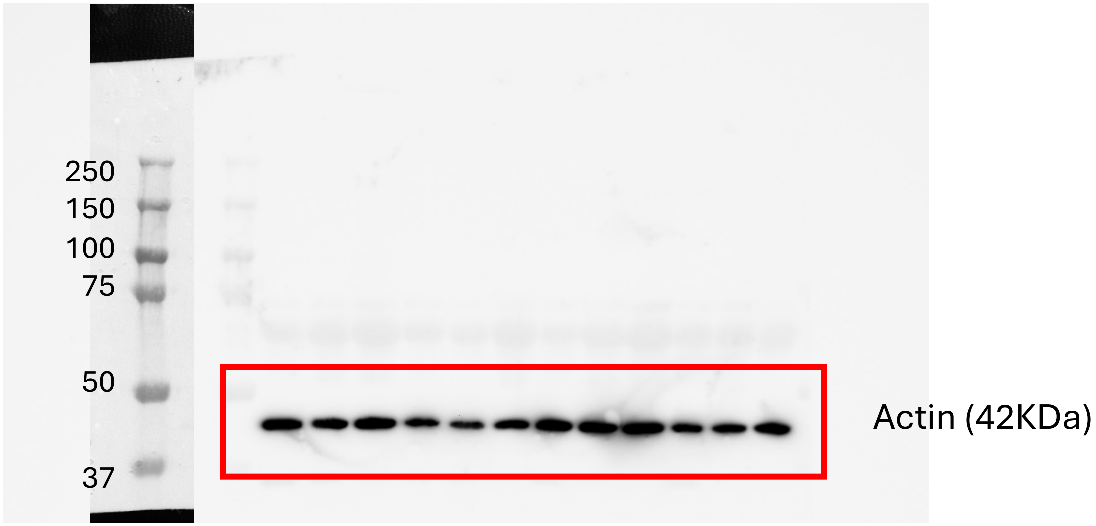

Uncropped western blots for Extended Data Fig 4d

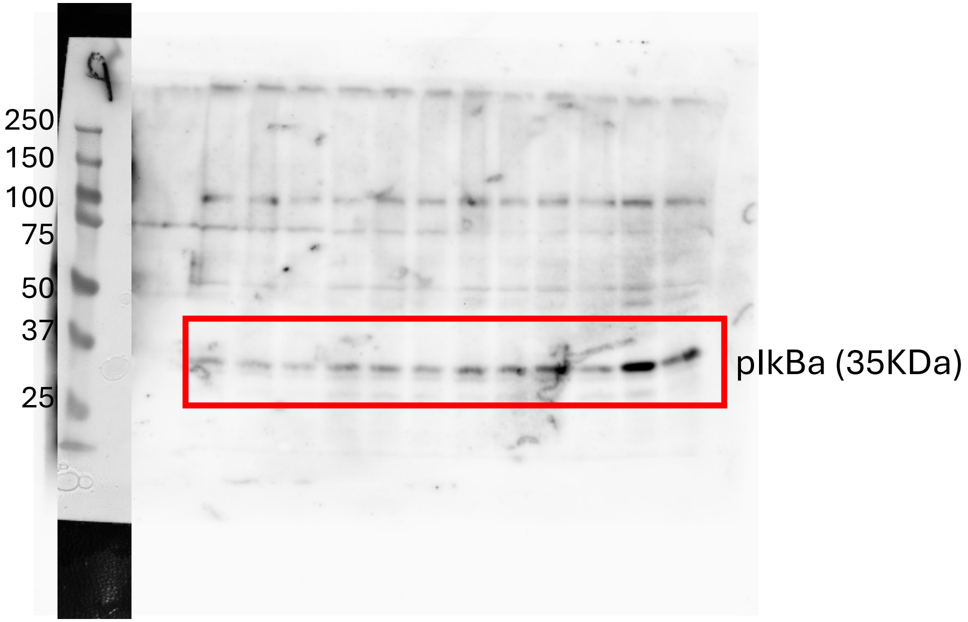

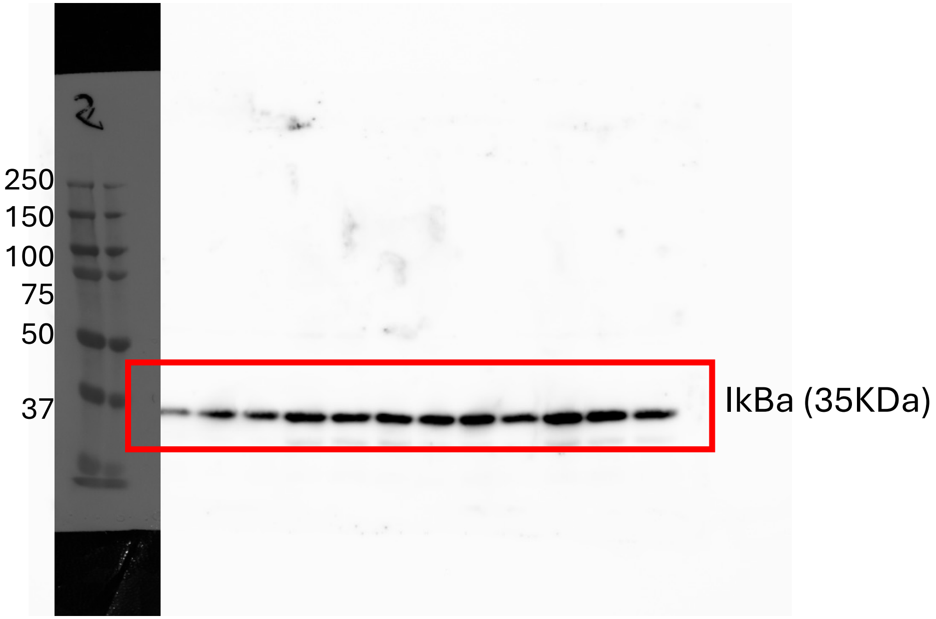

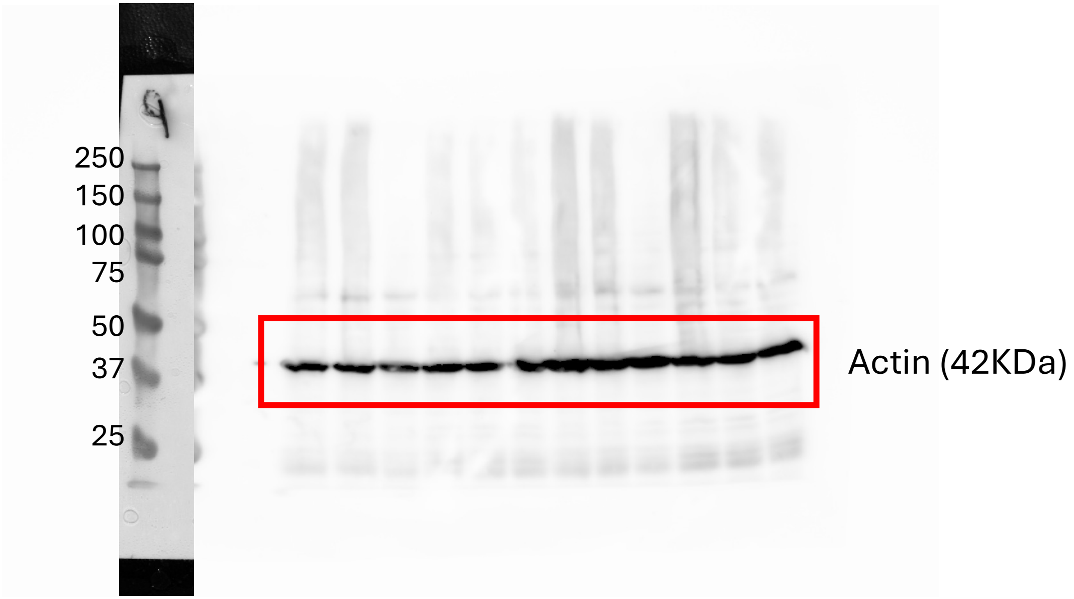

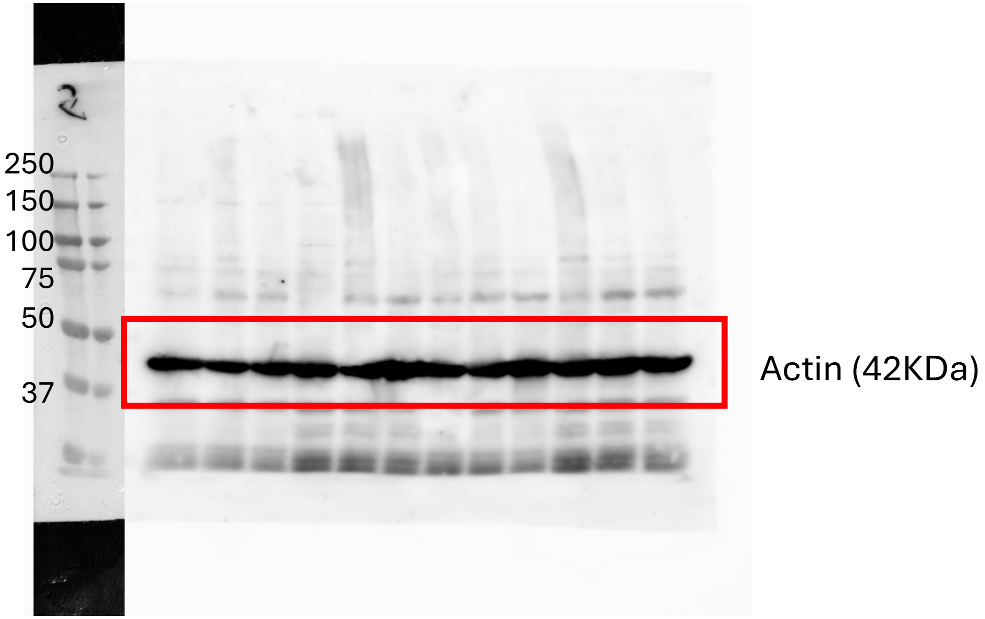

Uncropped western blots for Extended Data Fig 4e

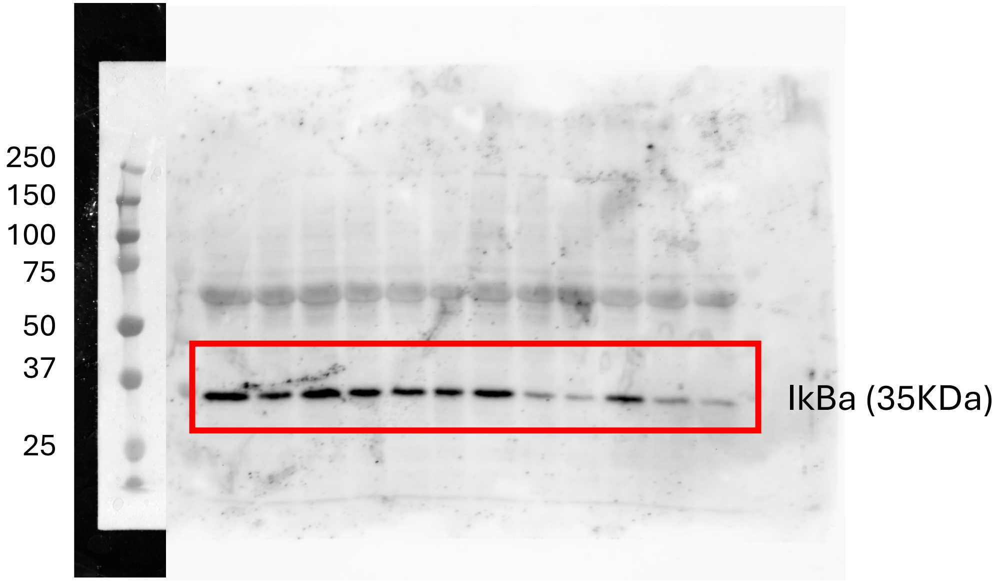

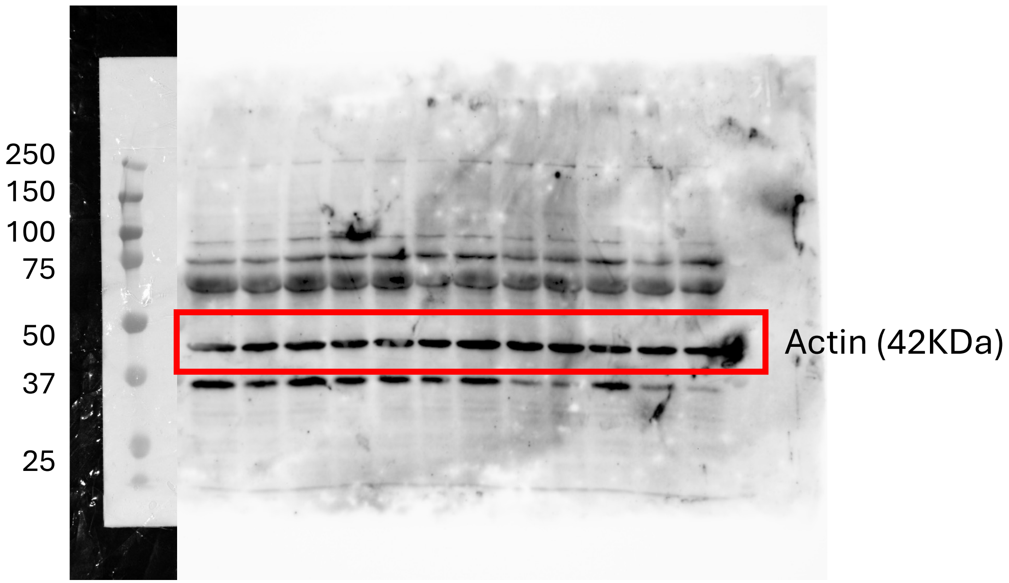

Uncropped western blots for Extended Data Fig 4j

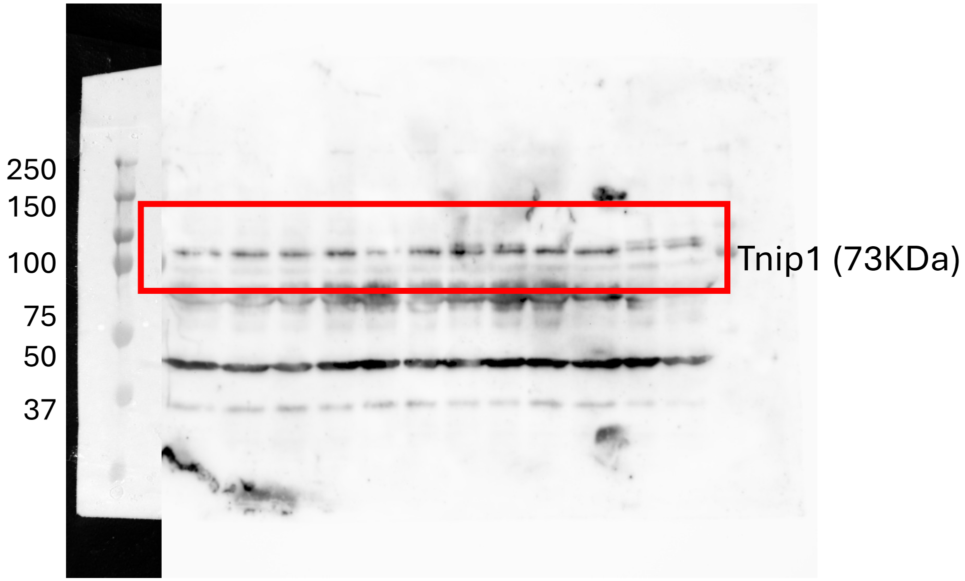

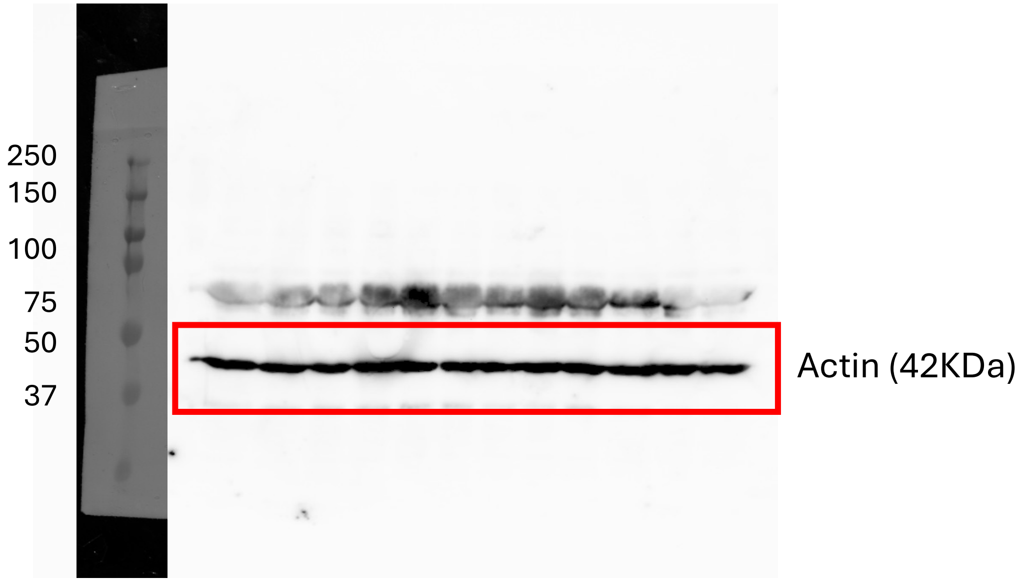

Uncropped western blots for Extended Data Fig 5i

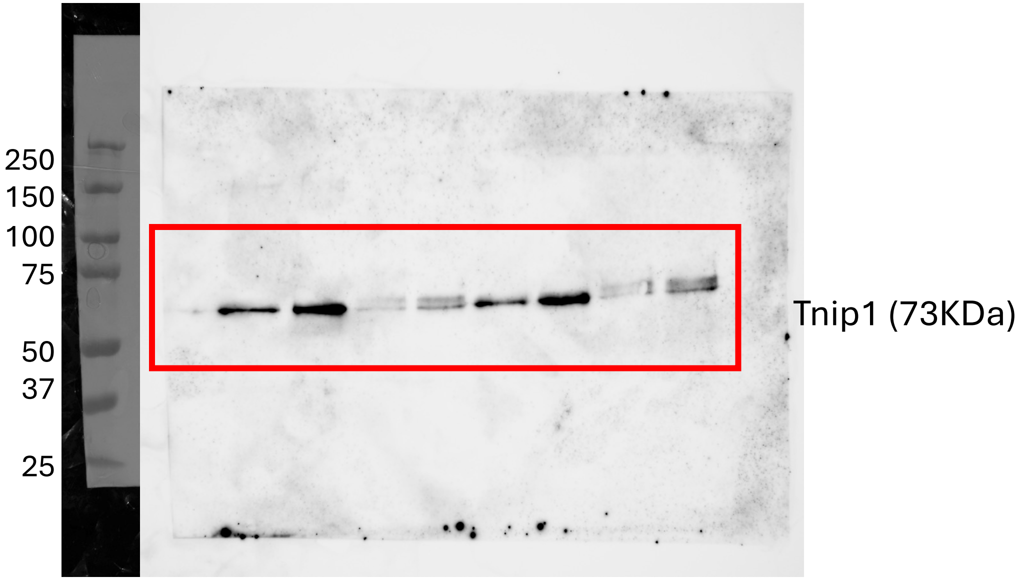

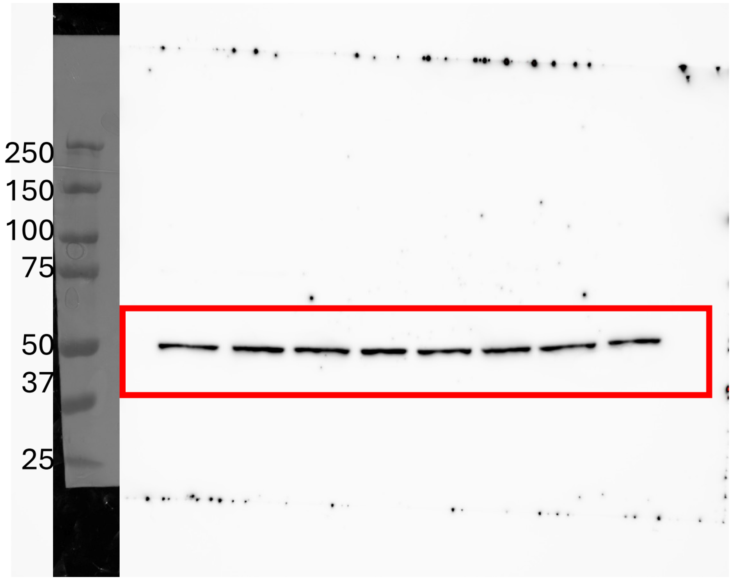

Uncropped western blots for Extended Data Fig 5j

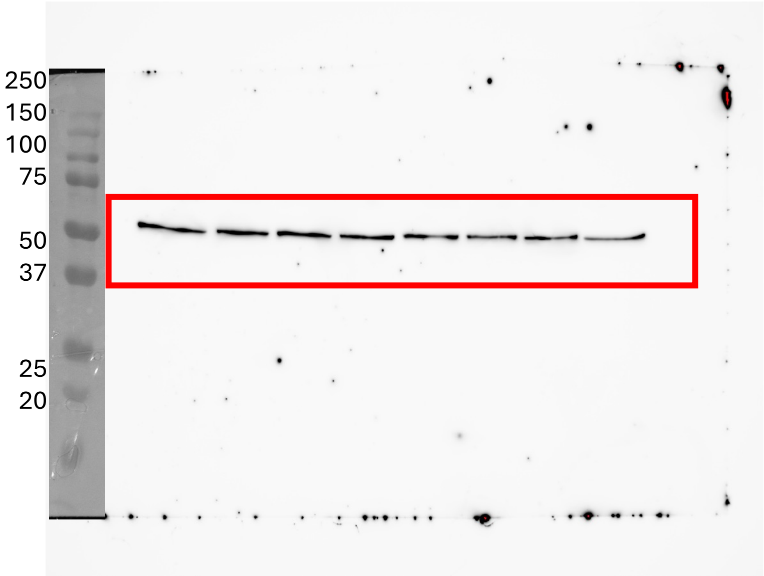

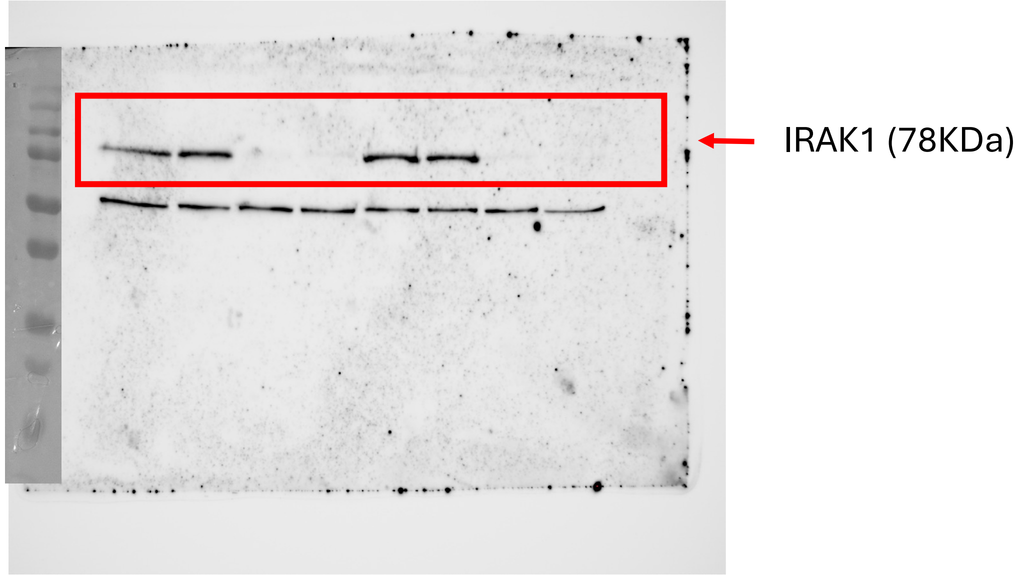

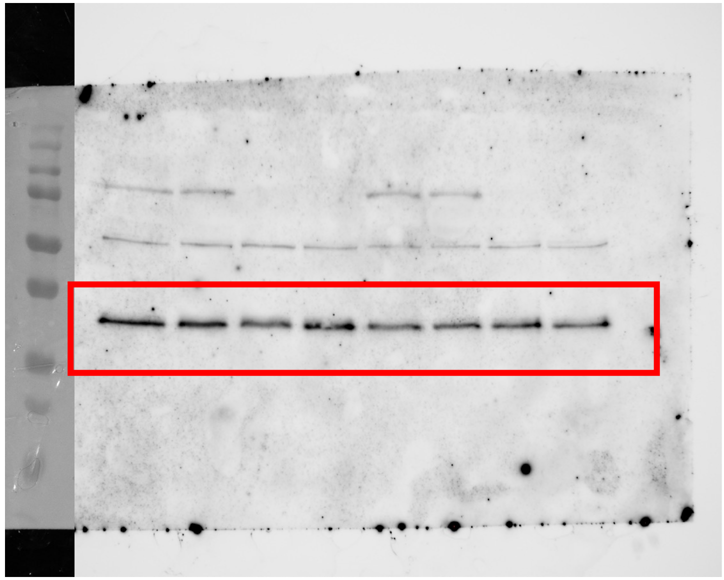

Supplement: Supplementary file 9 — Unprocessed immunoblots for Figs. 5d and 6e, and Extended Data Figs. 4a–e,j and 5i,j. [file 41590_2024_1902_MOESM9_ESM.pdf]
