# Supplementary figures and images for: A TNIP1-driven systemic autoimmune disorder with elevated IgG4 (part 2 of 2)
Source: Nat Immunol. 2024 Jul 26;25(9):1678–91. doi: 10.1038/s41590-024-01902-0 (PMC11362012; doi:10.1038/s41590-024-01902-0)

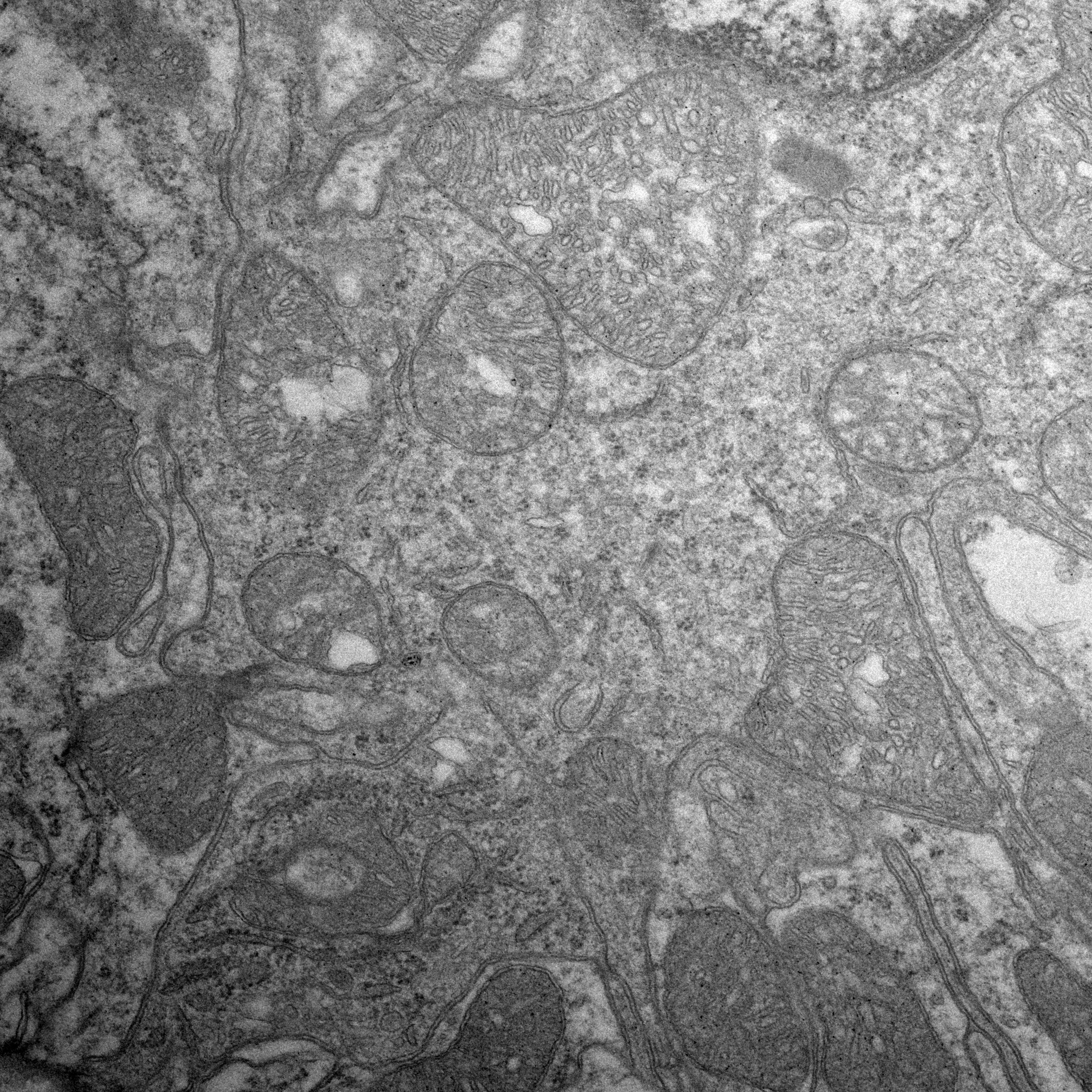

Supplement: Supplementary file 19 — Image files Extended Data Fig. 7a. [file 41590_2024_1902_MOESM19_ESM.zip › ED Fig 7a 6000X _80kV _Hom_14F.jpg]
